# Traditional use of medicinal plants in the boreal forest of Canada: review and perspectives

**DOI:** 10.1186/1746-4269-8-7

**Published:** 2012-01-30

**Authors:** Yadav Uprety, Hugo Asselin, Archana Dhakal, Nancy Julien

**Affiliations:** 1 Canada Research Chair in Aboriginal Forestry, Université du Québec en Abitibi-Témiscamingue, 445, boulevard de l'Université, Rouyn-Noranda, Québec, J9X 5E4, Canada; 2 Département des sciences de la santé, Université du Québec en Abitibi-Témiscamingue, 445, boulevard de l'Université, Rouyn-Noranda, Québec, J9X 5E4, Canada

**Keywords:** Medicinal plants, traditional knowledge, boreal forest, Aboriginal people, Algonquian, Athapaskan, conservation, management, policy

## Abstract

**Background:**

The boreal forest of Canada is home to several hundred thousands Aboriginal people who have been using medicinal plants in traditional health care systems for thousands of years. This knowledge, transmitted by oral tradition from generation to generation, has been eroding in recent decades due to rapid cultural change. Until now, published reviews about traditional uses of medicinal plants in boreal Canada have focused either on particular Aboriginal groups or on restricted regions. Here, we present a review of traditional uses of medicinal plants by the Aboriginal people of the entire Canadian boreal forest in order to provide comprehensive documentation, identify research gaps, and suggest perspectives for future research.

**Methods:**

A review of the literature published in scientific journals, books, theses and reports.

**Results:**

A total of 546 medicinal plant taxa used by the Aboriginal people of the Canadian boreal forest were reported in the reviewed literature. These plants were used to treat 28 disease and disorder categories, with the highest number of species being used for gastro-intestinal disorders, followed by musculoskeletal disorders. Herbs were the primary source of medicinal plants, followed by shrubs. The medicinal knowledge of Aboriginal peoples of the western Canadian boreal forest has been given considerably less attention by researchers. Canada is lacking comprehensive policy on harvesting, conservation and use of medicinal plants. This could be explained by the illusion of an infinite boreal forest, or by the fact that many boreal medicinal plant species are widely distributed.

**Conclusion:**

To our knowledge, this review is the most comprehensive to date to reveal the rich traditional medicinal knowledge of Aboriginal peoples of the Canadian boreal forest. Future ethnobotanical research endeavours should focus on documenting the knowledge held by Aboriginal groups that have so far received less attention, particularly those of the western boreal forest. In addition, several critical issues need to be addressed regarding the legal, ethical and cultural aspects of the conservation of medicinal plant species and the protection of the associated traditional knowledge.

## Background

Medicinal plants have been used in traditional health care systems since prehistoric times and are still the most important health care source for the vast majority of the population around the world [e.g. [1-6]]. It is estimated that 70-80% of people worldwide rely on traditional herbal medicine to meet their primary health care needs [7,8]. Globally, millions of people rely on medicinal plants not only for primary health care, but also for income generation and livelihood improvement [8]. Annual sales of herbal-based medicines range between 7.5 billion US$ and 108 billion US$ worldwide, the latter number representing sales of processed medicines [9]. In Canada annual market sales of medicinal plants reached 400 million US$ in 2001 [10], and are growing at a pace of 15% annually [11].

Through millennia of trial and error, indigenous people have gained substantial knowledge of medicinal plants which has been transmitted from generation to generation as part of oral traditions [12,13]. However, concerns are being raised about the loss of native knowledge and the possible extinction of medicinal plant resources due to disruptions to traditional ways of life induced by colonial forces [14-17]. Hence, proper documentation of traditional knowledge regarding plant use, along with conservation and sustainable management of key habitats, could contribute to safeguarding this heritage [18].

A few studies have attempted to review the use of medicinal plants by the Aboriginal people of Canada, but they focused either on particular Aboriginal groups [e.g. [19]], or on restricted regions [e.g. eastern Canada [20]]. Here we present a review of the traditional use of medicinal plants by the Aboriginal peoples of the Canadian boreal forest. Furthermore, we provide complementary information on conservation status of medicinal plant species, as well as on policies framing medicinal plant use and traditional knowledge. Finally, we identify gaps in knowledge, and suggest perspectives for future research.

### Canada's Aboriginal People and the Boreal Forest

**Figure 1:**
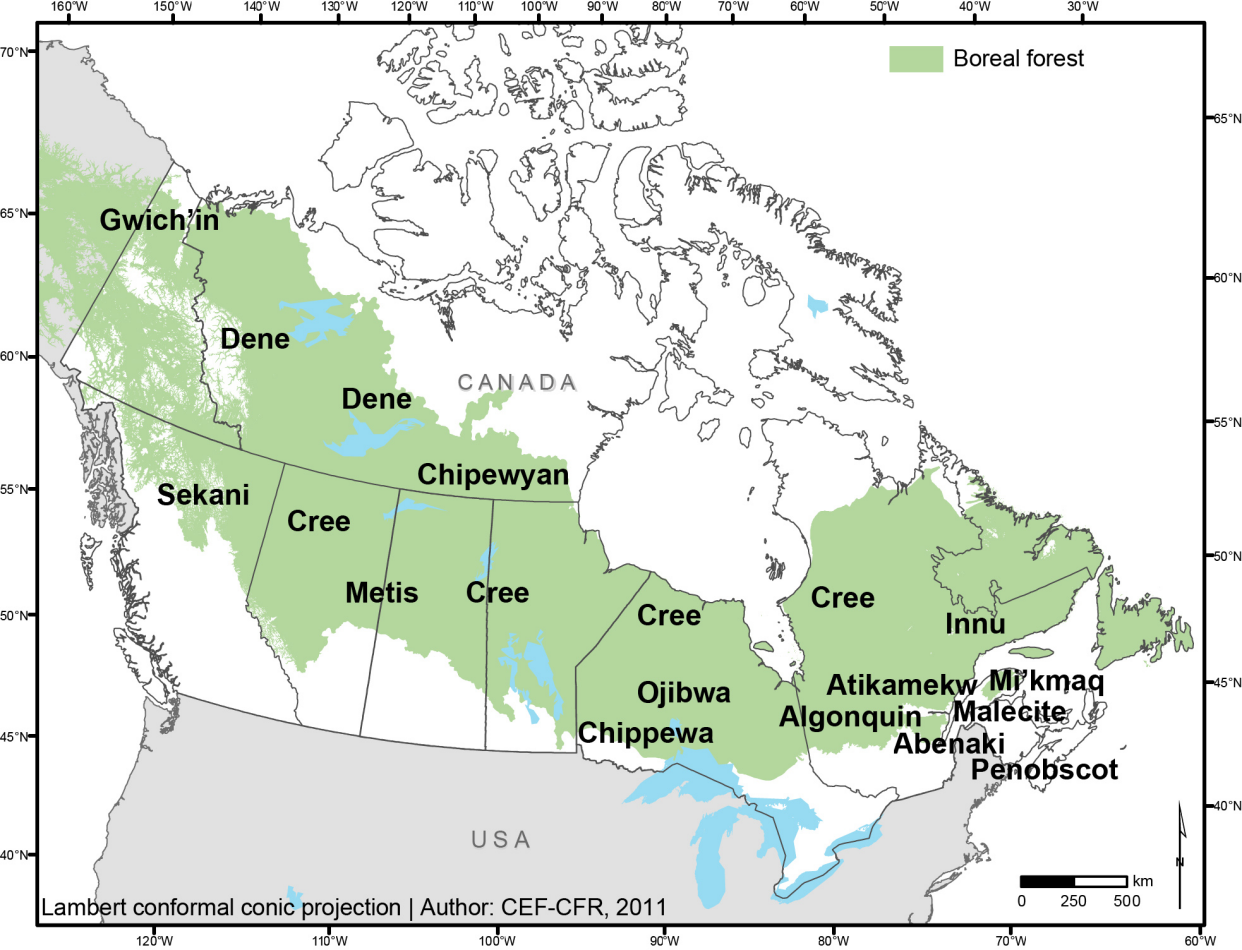
**The boreal forest of Canada, and names and approximate locations of Aboriginal peoples discussed in this review**. Note that although Metis communities are found in most of Canada, it is the communities from the central provinces that were reported in the studies included in this review.

Sometimes called the land of much geography and little history [21], Canada is blessed with an immense forest endowment [22]. The circumboreal forest is the most extensive terrestrial biome in the world, encompassing some 14 million km^2 ^and 32% of the Earth's forest cover. Thirty percent of this world resource is found in Canada, where it occupies 58% of the nation's land area comprising seven boreal and taiga ecozones [23]. Canada's boreal ecozones cover 5.8 million km^2^, of which 4.3 million km^2 ^are forested [24]. The boreal forest of Canada is a broad vegetation zone (Figure 1) that stretches from the northern Atlantic coast westward to the Rocky Mountains, across the southwestern Northwest Territories and Yukon. The southern edge borders the eastern hardwood forest, abuts the north shores of the Great Lakes, and in the west intergrades with the aspen parkland and prairie. The northern edge fades into the tundra.

According to the 2006 census [25], there were 1 172 790 Aboriginal people in Canada (including First Nations, Metis and Inuit), representing ca. 4% of the total population. The proportion of Aboriginal people is much higher in the boreal zone [26], reaching more than 30% of the total population in several areas [27]. Aboriginal peoples of the Canadian boreal forest are part of two major linguistic groups [28]: the central and eastern boreal forest is home to peoples of the Algonquian linguistic group (Mi'kmaq, Malecite, Abenaki, Penobscot, Innu/Montagnais, Cree, Atikamekw, Algonquin/Anishinabe, Ojibwa/Anishinabe, Chippewa), whereas the northwestern boreal forest is home to peoples of the Athapaskan linguistic group (Chipewyan, Dene, Gwich'in, Sekani)^i ^(Figure 1).

Canada's forests have long played an integral role in supporting the lives of Aboriginal people, meeting their physical, cultural, spiritual and material needs. Traditional knowledge related to medicinal plants has been instrumental in the survival and wellbeing of Aboriginal people for thousands of years [29]. Unfortunately, this type of knowledge has been seriously eroding over the past decades [14,29] indicating challenges for restoration. The concept of Aboriginal forestry, increasingly used in recent years [30-33], provides a means to keep indigenous cultures healthy and connected to the land [34-36]. Aboriginal communities possess their own traditional knowledge that contributes to a broader understanding of native plant species, many of which have yet to be studied by "western" scientists.

## Methods

We reviewed scientific studies published in journals, books, theses and reports. Pertinent literature was searched in different electronic databases (ISI Web of Science, MEDLINE, Science Direct, Scopus, and Google Scholar) using specific search terms such as "medicinal plants", "traditional", "Aboriginal OR First Nation OR Indigenous OR Indian", "boreal", and "Canada". We do not claim to have included every existing information source about traditional uses of medicinal plants, but we rather chose to focus on information easily accessible to researchers (available on the internet or through interlibrary loan). We are aware that several Aboriginal communities have endeavoured to record their members' traditional knowledge related to medicinal plants over the last few decades. However, in most cases, this information has yet to be made available to outsiders.

We reviewed a total of 49 publications that provided information about the use of medicinal plant species to treat various ailments. We only used publications presenting first-hand ethnobotanical information. Previously published reviews were consulted but were not included in the analyses. A master list was produced, showing name(s), part(s) used, use(s), and reference(s) for each species (Additional file 1).

Although we focused our search on traditional medicinal practices of Aboriginal people living in the Canadian boreal forest, some of the inventoried plants are distributed partly or entirely outside the boreal forest (e.g., in the temperate forest or in arctic or alpine areas). Large-scale trade networks between different nations are known to have existed in the past [37], allowing boreal peoples to obtain plants from contiguous areas in exchange for other goods.

The precision of botanical identification in this review depended on that from original sources. Latin names and native status (native vs. introduced) were verified in the Plant Database of the United States Department of Agriculture, Natural Resources Conservation Service [38], the Plant Database of the Missouri Botanical Garden [39], the Flora of North America [40], and the Canadian Vascular Plants Database (VASCAN) [41]. Whenever available, subspecies (ssp.) and variety (var.) names are also provided. The currently accepted name is followed by synonyms, when provided in the source references. In some cases, only the genus was provided in the literature [e.g. [42-44]] and we did not attempt to refine the information to the species level.

The Aboriginal plant names mentioned in this review were taken textually from the original sources, whenever they were reported. Since North American Aboriginal cultures were based on oral tradition prior to European contact, various spellings exist for the same word. No selection was done and all variants are provided. Most Aboriginal languages are descriptive and thus the name given to a plant often refers to its appearance or function rather than to genetic uniqueness. Identical names are thus sometimes given to different species, or different names to the same species. To some extent, the approach shows similarities with the concept of plant functional traits [45].

Traditional plant uses are provided with the name of the Aboriginal group whenever the information was available, and the reference from which the information was retrieved. We followed the method proposed by Cook [46] to classify plants according to the different ailment categories they help to cure. However, in some cases Cook's [46] categories were not precise enough and plants were assigned to additional ailment categories.

### Ethnomedicine of Boreal Canada

Traditional medicine among the Aboriginal peoples of the Canadian boreal forest is based on oral tradition transmitted through several generations [13,47]. It is a cultural phenomenon, dynamic and adaptive, like language and other cultural manifestations [13]. The holistic approach of Aboriginal healing systems involves spirituality and intimate connection with the natural environment [47,48]. It also involves strong community networks of people who participate in the process of healing, and who can direct community members to people who have the knowledge to facilitate healing.

Vogel [49] compiled the historical context of Aboriginal peoples' medicinal culture and discussed shamanistic and spiritual aspects. Moerman [50-52] published some of the most complete ethnobotanical compilations for North America, including boreal Canada. Shemluck [53] provided a review of the medicinal uses of species from the Asteraceae family by North American Aboriginal people. Andre et al. [54] compiled the medicinal knowledge of Arctic and Subarctic indigenous people.

#### Ethnomedicine of Eastern and Central Canada

Assiniwi [55] and Erichsen-Brown [56] reported uses of medicinal plants by eastern North American Aboriginal people. Foster and Duke [57] published a useful field guide to the medicinal plants of central and eastern North America. Arnason et al. [20] reported on some 400 medicinal plants used in traditional health care systems by Aboriginal people of eastern Canada. Only for the Maritimes, 128 medicinal plant species were reported to be used by Chandler et al. [58]. Medicinal plants knowledge of Aboriginal people from the Maritimes has also been explored by several other researchers [43,48,59-65]. Youngken [44,66] studied the medicinal knowledge of several groups from the northeastern United States and Canada and Rousseau [67] worked with the Abenaki. Speck [63] reported on Algonquian peoples' knowledge, whereas Marie-Victorin [68] and Black [69] specifically worked with the Algonquin. Tantaquidgeon [70], Clément [71] and Laurendeau [72] explored Montagnais (Innu) knowledge, Raymond [73] worked with the Atikamekw, whereas Holmes [42], Strath [74], Jenkins [75], Beardsley [76], Grandi [77], Williams and Glover [78], Iserhoff et al. [79], Marshall [80,81], Fraser [82] and Leduc et al. [83] focused on Cree territory. The Ojibwa knowledge of medicinal plants has long been a subject of great attention and it has therefore contributed a lot to the ethnobotany literature of the Central boreal region [47,84-89]. Some of these studies were reviewed and compiled by Meeker et al. [19] who provided detailed information about 384 plants used by the Ojibwa^ii^. Davidson-Hunt et al. [90] provided identification, classification and nomenclature systems for plants used by the Ojibwa. Beresford-Kroeger [91] provided some information about traditional medicinal uses of tree species found in northeastern North America.

#### Ethnomedicine of Western Canada

Marles et al. [13] described the traditional use of plants by Cree, Dene, and Métis peoples of Manitoba, Saskatchewan, and Alberta. This study also partly covered eastern Canada, and it is the only one to cover Métis medicinal culture. Marles [92] also worked with the Chipewyan of northern Saskatchewan. Siegfried [93] documented the ethnomedicinal knowledge of the Alberta Cree. Leighton [94,95] and Clavelle [96] studied the Crees of Saskatchewan and Smith [97] reported on medicinal plant uses by the Sekani of British Columbia. Studies were also conducted with First Nations from the Dene group in the western boreal region: the Fisherman Lake Slave [98] the Prophet River First Nation [17], the Gwich'in [99,100] and the Dogrib [101].

#### Taxonomic Diversity, Growth habit and Parts Used

**Figure 2:**
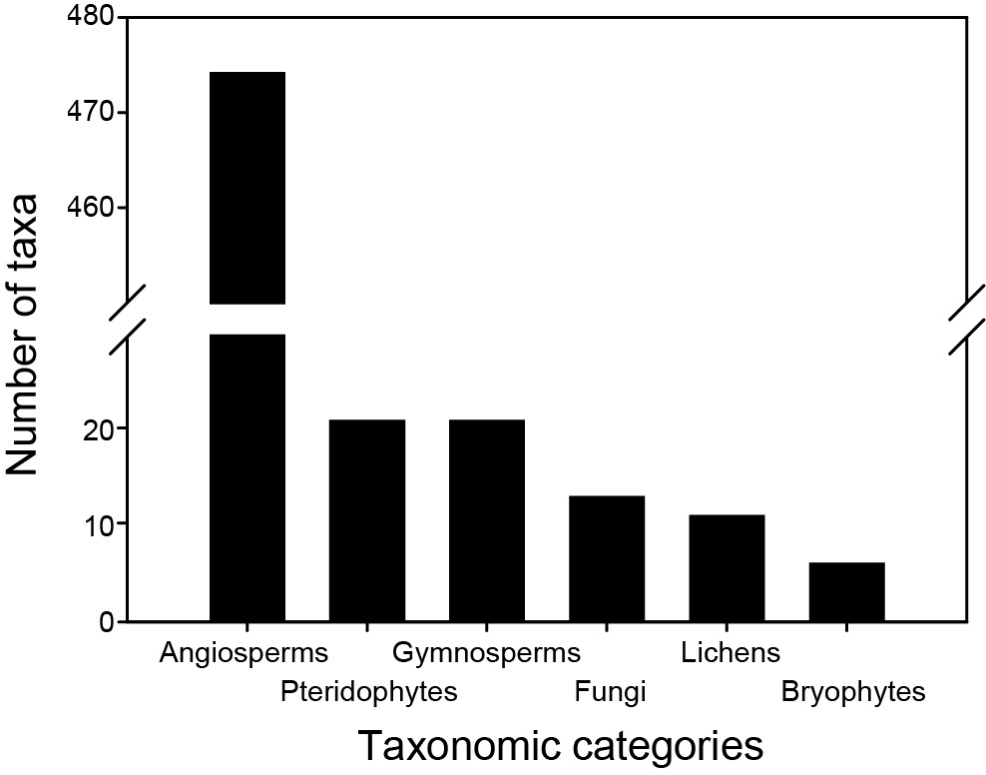
**Frequency of medicinal plant taxa in major taxonomic categories**.

**Figure 3:**
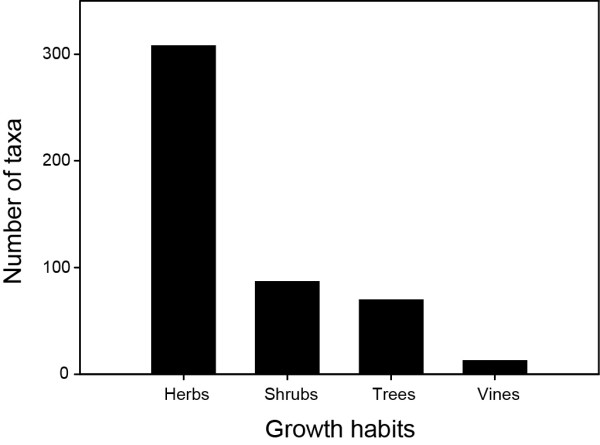
**Frequency of medicinal flowering plant taxa in different growth habits**.

We report on a total of 546 medicinal plant taxa used by Aboriginal peoples of the Canadian boreal forest (Additional file 1). Although most information was available at the species or even subspecies level, sometimes only the genera was provided. Among the most commonly used plants were: *Abies balsamea *(L.) Mill., *Achillea millefolium *L., *Acorus calamus *L., *Aralia nudicaulis *L., *Betula papyrifera *Marsh., *Cornus sericea *L., *Heracleum maximum *Bartram, *Juniperus communis *L., *Larix laricina *(Du Roi) K. Koch, *Menta arvensis *L., *Nuphar lutea *(L.) Sm, *Picea glauca *(Moench) Voss, *Picea mariana *(Mill.) BSP, *Populus balsamifera *L., *Populus tremuloides *Michx, *Rhododendron groenlandicum *(Oeder) K.A. Kron & W.S. Judd., *Salix *sp., *Sorbus americana *Marsh, and *Thuja occidentalis *L. Angiosperms were predominant, with 474 taxa belonging to 90 families, followed by Pteridophytes (21 taxa from 5 families), Gymnosperms (21 taxa from 3 families), Fungi (13 taxa from 7 families), Lichens (11 taxa from 4 families), and Bryophytes (6 taxa from 2 families) (Figure 2). Well represented Angiosperm families were Asteraceae (62 species), Rosaceae (48), Liliaceae (21), Ericaceae (18), Betulaceae (18), Caprifoliaceae (18), Ranunculaceae (16), Salicaceae (16), Polygonaceae (15) and Lamiaceae (13). The prevailing growth habit (*sensu *[38]) of angiosperms medicinal plant taxa was herb (307 species), most likely because they are more abundant (Figure 3). The more abundant a plant is, the more likely it is to be used. The next dominant growth habit of angiosperm taxa was shrub (86), followed by tree (69) and vine (12). Of the 21 gymnosperm taxa, 19 were trees and 2 were shrubs. All dominant species of angiosperms and gymnosperms of the boreal forest were being used as medicinal plants. Furthermore, forty-nine introduced species were part of the native pharmacopoeia (Additional file 1).

Almost all plant parts were used to prepare different remedies: roots, rhizomes, stem, bark, leaves, flowers, fruits, young shoots, and whole plants (Additional file 1). The most frequently used plant parts were roots, followed by leaves, whole plants, fruits, and rhizomes.

#### Ailments Treated and Preparation Methods

A total of 28 major ailment categories were treated with medicinal plants (Table 1). Gastro-intestinal disorders, musculoskeletal disorders, cold, cough and sore throat, injuries, respiratory system disorders, urinary system disorders, and dermatological infections were treated with the highest diversity of medicinal plant species (Table 1 Additional file 2).

**Table 1:** Major ailment categories and taxa reported.

Ailment category	Number of taxa*	Number of use reports
Blood system	39	17
Circulatory system	53	16
Cold, cough and sore throat	130	19
Dermatological	100	40
Diabetes	42	7
Ear	20	17
Fainting and fits	18	9
Fever	62	22
Gastro-intestinal system	214	26
General	89	26
Gynaecological	85	20
Haemorrhages	38	19
Hair	14	7
Headache	69	28
Injuries	119	36
Mental	18	9
Metabolic system	20	11
Musculoskeletal system	134	34
Nervous system	31	12
Nutritional	70	24
Odontological	44	23
Ophthalmological	57	27
Poisoning	41	16
Pregnancy/Birth/Puerperium	83	22
Respiratory system	118	32
Sexual dysfunction	3	2
Urinary system	109	33
Venereal	28	14

*Most taxa were reported in more than one ailment category (see Additional file 1).

Preparation methods included paste, poultice, juice, decoction, infusion, and chewing the raw plant (Table 2 Additional file 1). The majority of formulations were prepared as decoction or infusion (Additional file 1). Some formulations were prepared using combinations of different plants, sometimes as many as 20 substances being combined in one remedy [47]. Some formulations also included animal organs or fat [47,85,95]. Proper selection of species, parts, as well as preparation and administration methods are all very important in traditional health care systems [17,81]. Medicinal plant use should be carried out under the supervision of a knowledgeable person [81], usually an elder [102], as some plants might be poisonous (even lethal, e.g., *Cicuta, Taxus, Veratrum*), or could cause adverse reactions when taken in combination with other plants or with western medicine.

**Table 2:** Common forms of preparation methods for remedies made of medicinal plants.

Preparation method	Description
Paste	Fresh plant parts are crushed to obtain a paste used externally or internally.
Poultice	Plant parts are crushed to obtain a soft moist mass generally used externally to treat swellings, pain, inflamed or infected body parts.
Juice	Obtained by squeezing or crushing plant parts and filtering through cloth. Sometimes requires addition of freshwater or other liquid for dilution.
Powder	Obtained by crushing dried plant parts.
Chewing	Fresh plant parts are chewed without prior transformation.
Infusion	Plant parts are plunged in either hot or cold water for several minutes. If hot water is used infusion is taken as a tea. More than one plant species can be used in conjunction.
Decoction	Plant parts are boiled in water for several minutes and the extract is used. More than one plant species can be used in conjunction.

In some Aboriginal medicinal cultures the various processes of healing are connected with ceremonies and rites [47,70]. Those who possess the secret cures sometimes think that if they disclose them too freely the herbs will lose their potency [63]. When a plant is collected, it is important to leave a small offering (tobacco, matches, tea, rifle or shotgun shells, money, sugar or a prayer) in place of what is taken [17,20,99]. This ritual shows respect for the plant and increases the healing power of the medicine [20]. The season of collection and proper storage conditions are considered important for the effectiveness of remedies. Plants are usually gathered in late summer or early fall, when fully developed [47,58]. In some instances the fertile and sterile plants are considered separately [47]. When bark is used, it is sometimes collected from the eastern side of the tree [65,70]. When roots are used, the healing power is deemed stronger in certain portions than in others [47]. Medicinal plants harvesting practices are integral to the healing process, and crucial for resource preservation.

#### Phytochemical and Pharmacological Studies of Boreal Canadian Medicinal Plants

The most frequent approach to species selection for phytochemical, pharmacological or antimicrobial analysis is by reviewing the ethnobotanical literature. This highlights the importance of such studies in western pharmacognosy. Phytochemical and pharmacological studies investigating medicinal properties of North American plant species used to be lacking [20]. However, there was a marked increase in such studies in recent years, pinpointing the active principles of many plants used by Aboriginal peoples of Canada [12]. Examples of such studies are: Chandler and Hooper [103], Wat et al. [104], Chandler and Hooper [105], Hooper and Chandler [106,107], Bergeron et al. [108], Owen and Johns [109], Jones et al. [110], Lin et al. [111], McCune and Johns [112-114], Ficker et al. [115], Bafi-Yeboa et al. [116], Spoor et al. [117], Awad et al. [118], Tsao and Liu [119], Webster et al. [120], Marles [92] and Martineau et al. [121].

At the species level, Chandler et al. [122,123] highlighted the correspondence between traditional use and phytochemical and pharmacological properties of *Achillea millefolium *and *Tanacetum vulgare*, two of the most widely used medicinal plant species in boreal Canada (Additional file 1). Applequist and Moerman [124] also reviewed ethnobotany and bioactivity of *Achillea millefolium*. Dufour [125] and de Moor [126] respectively studied biological activity in *Rhododendron groenlandicum *and *Abies balsamea*. Saxena et al. [127] analyzed the antimicrobial activities of *Rhus glabra*, Kobaisy et al. [128] highlighted the antimycobacterial activity of *Oplopanax horridus*, Kitts et al. [129] and Vuksan and Sievenpiper [130] considered *Panax quinquefolius*, Murch et al. [131]*Hypericum perforatum*, Petzke et al. [132]* Taxus canadensis*, and Matsuo et al. [133]* Caulophyllum thalictroides*. Matsuura et al. [134] worked on *Empetrum nigrum*.

Several studies were conducted on the anti-diabetic properties of medicinal plants, as diabetes is a serious concern for Aboriginal people of Canada [83,117,135-138]. Most of the scientific research into the anti-diabetic properties of boreal medicinal plants has been done in partnership with Cree communities of northern Quebec.

### Conservation and Management of Medicinal Plants

Conservation and management of traditional medicinal plants is an important issue worldwide, mostly in developing countries where medicinal plants are primary forest products for rural communities. The nature of the interactions between traditional practices and local ecosystems can be both positive and negative depending on use intensity, intent (for local/personal use or for commercial use) and magnitude of exploitation [139]. Plants should only be collected in such a manner that ensures their continued presence, both in specific collection locations and across the landscape [19]. One of the biggest threats to the survival of medicinal plant species is habitat loss due to infrastructure development, mining, forestry, oil and gas exploitation, and hydro power generation projects [19]. Because many plant species have medicinal properties, their conservation could foster the preservation of important habitats for other species of plants and wildlife.

Conservation and management of medicinal plants has been given less attention in Canada than elsewhere, although it has been discussed recently [19,140-143]. Some management issues have been raised related to non-timber forest products (NTFPs), but without special focus on medicinal plants [144,145]. The most serious threats to boreal plants are habitat loss and fragmentation, climate change, and invasive species [143]. It is not known whether overexploitation is an issue in boreal Canada, as it is in several countries worldwide [18]. According to Westfall and Glickman [140], there is no formal system of accounting for medicinal plant harvesting in Canada, and thus little is known about which plants are being harvested, from where, and in what quantity. It could be too late to address conservation issues as the lack of a proper accounting system does not offer an up-to-date portrait of the status and scale of exploitation of medicinal plants. High pressure from the timber harvesting industry poses severe threats to medicinal plants in boreal Canada, especially to species associated to old-growth forests.

An integrated, collaborative approach for sustainable use, conservation and management of medicinal plants should be put into place and involve all stakeholders [146]. However, local peculiarities should be taken into account and the role of stakeholders might be different in different locations as the Aboriginal peoples of boreal Canada have different legal relationships with the federal and provincial governments. National level conservation policies do not necessarily apply within reserve boundaries. Furthermore, treaties and agreements between the governments and some First Nations have granted the latter different levels of control over natural resources [147].

Special care has to be given when attributing a legal protection status to a species because of over-harvesting by non Aboriginal people, as it will prevent Aboriginal people from using a resource they have been counting on for centuries. Furthermore, small-scale, aboriginal-led businesses based on *in situ *collection of medicinal plants provide interesting sustainable livelihood options. Special efforts have thus to be made to identify important plant collection areas [148]. Zoning tools could be especially helpful to decision making [149].

Of the 546 plant species included in this review, only six were listed as imperilled according to the Species at risk public registry [150]: *Betula lenta, Cornus florida, Panax quinquefolius, Juglans cinerea, Solidago speciosa *were endangered, whereas *Aletris farinosa *was threatened. Such a low percentage of species at risk could probably be explained by three factors: (1) most boreal plants have extensive distributions, (2) the boreal zone has been less impacted by human activities than more southern areas, and (3) the plants used by aboriginal people tend to be the more common ones.

### Challenges to Traditional Medicinal Plants Research

**Figure 4:**
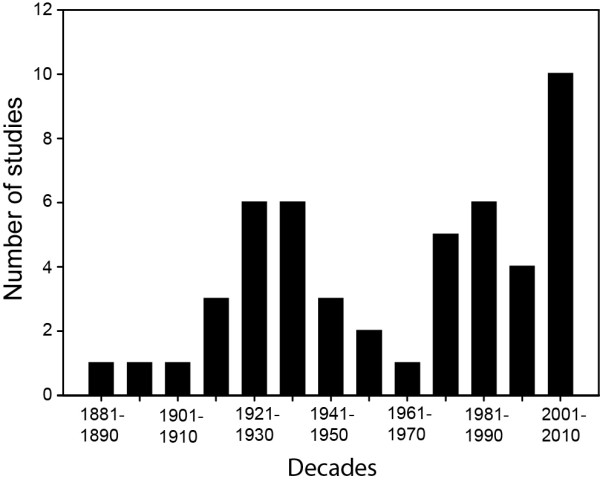
**Number of studies included in this review for each decade between 1881 and 2010**.

Aboriginal people of Canada are worried that their knowledge could be stolen by profit-seeking pharmaceutical companies without acknowledging or involving communities [143], and without proper compensation being given in return. Concerns about the respect of intellectual property rights thus render most Aboriginal people reluctant to disclose their knowledge to outsiders [151], especially as legal protection is insufficient. This could explain why published ethnobotanical studies were somewhat less numerous in boreal Canada between the 1950s and 1970s (Figure 4). Informal discussions with Cree and Algonquin communities from Northern Quebec indeed revealed that Aboriginal people are cautious in reaction to misguided research practices by academics and government agencies. The historical background where Aboriginal people have suffered more inconveniences than they have benefited from European settlement is doubtless contributing to this generalized mistrust [21]. Also important is that land claims have not yet been settled for most Aboriginal communities and nations [152,153]. Aboriginal people would probably be more open to share their knowledge if they had more power in land governance [154].

In such conditions ethnobotanists face important challenges related to trust building and safeguarding traditional people's intellectual property rights. Nevertheless, a trustful environment can be favoured by considering the following ethical principles [after CNR [142]]:

- respect Aboriginal culture and protect sensitive and confidential information;

- ensure that knowledge ownership and project leadership resides with Aboriginal people;

- share benefits with communities;

- contribute to capacity building in Aboriginal communities;

- take into account and protect the interrelationship between environment, health and culture;

- obtain clear and informed consent from research participants, pay careful attention to ethical and legal issues, and obtain ethical approval of research protocols;

- respect Aboriginal and treaty rights.

### Policy and Institutional Framework Related to Medicinal Plants

#### International Perspective

Since its adoption in 1992, the United Nations Convention on Biological Diversity (CBD) has strived to implement its three major goals: conservation of biological diversity, sustainable use of its components, and a fair and equitable sharing of the benefits from the use of genetic resources [155]. Although medicinal plants were not explicitly on the agenda of the various CBD meetings, all three goals of the Convention are fully applicable to medicinal plant resources [156]. According to CBD's Article 8 (j): Traditional Knowledge, Innovations and Practices, signatories agree to

*"respect, preserve and maintain knowledge, innovations and practices of indigenous and local communities embodying traditional lifestyles relevant for the conservation and sustainable use of biological diversity and promote their wider application with the approval and involvement of the holders of such knowledge, innovations and practices and encourage the equitable sharing of the benefits arising from the utilization of such knowledge innovations and practices"*.

Agenda 21 and Forest Principles adopted at the UN Conference on Environment and Development (UNCED) identified forest products other than wood (also called non-timber forest products or NTFPs) as an important area requiring increased attention and as a source of environmentally-sound and sustainable development [157]. Since the Johannesburg Earth Summit in 2002, much attention has been given to the possibility of combining biodiversity conservation and poverty alleviation [158]. Via their legislations, countries are obliged to implement these various policy measures to ensure that traditional knowledge and intellectual property rights are respected [159]. The Conference of the Parties to the Convention on Biological Diversity held in Nagoya (Japan) in 2010 discussed the access and benefit sharing issues of sustainable use of biodiversity [160]. The Agreement on Trade Related Aspects of Intellectual Property Rights mandates countries to safeguard intellectual property rights [159]. The UN Declaration on the Rights of Indigenous Peoples, Article 24 includes provisions for use of resources (including medicinal plants) and rights over territories [161]:

*"Indigenous peoples have the right to their traditional medicines and to maintain their health practices, including the conservation of their vital medicinal plants, animals and minerals. Indigenous individuals also have the right to access, without any discrimination, to all social and health services"*.

The World Health Organization (WHO) has drafted several guidelines and passed resolutions for the integration of traditional health care systems and remedies into national health policies and regulations [8,162-165]. The specific guidelines on conservation of medicinal plants are provided in WHO *et al*. (1993). Organizations like WHO, the World Wildlife Fund (WWF), the World Conservation Union (IUCN), the UN Food and Agriculture Organization (FAO), the wildlife trade monitoring network-TRAFFIC, the International Development Research Centre (IDRC), and Plant Life International have been involved in the medicinal plants sector for a long time [see [8,165,166]].

Various recommendations have been made on the use and conservation of medicinal plants, such as those associated with international conferences at Chiang Mai, Thailand, in 1988, and Bangalore, India, in 1998 and 2009 [18,167]. They included the need for co-ordinated conservation action, based on both *in situ *and *ex situ *strategies; inclusion of community and gender perspectives in the development of policies and programmes; the need for more information on medicinal plants trade; establishment of systems for inventorying and monitoring medicinal plants status; development of sustainable harvesting practices; encouragement of micro-enterprise development by indigenous and rural communities; and protection of traditional resources and intellectual property rights [18]. The recent International Healers' Conference on Promotion of Traditional Medicine for Sustainable Healthcare [167] called for the promotion of self-regulation of all traditional health professions, capacity building in local communities to develop biocultural protocols, integration of traditional medicine into national healthcare systems, and establishment of a Permanent Forum on Traditional Health Practices at the United Nations.

The regulation of existing markets by setting environmental standards for international trade is a traditional instrument advocated by international environmental policy [168]. Biodiversity conservation oriented trade policy measures are components of international agreements (e.g., the listing of species in CITES appendices and banning of certain species for trade on the international market). Likewise, the General Agreement on Tariffs and Trade (GATT) also regulates biodiversity trade and imposes certain restrictions on international trade of plant resources, including several species of medicinal plants. Existing guidelines for the sustainable collection of NTFPs provide useful models for medicinal plants, including the Forest Stewardship Council (FSC) sustainable forest management standard, the International Federation of Organic Agricultural Movements (IFOAM), and Fairtrade Labelling Organizations International (FLO) [2].

#### National Perspective

Comprehensive national policy, laws and regulations on traditional medicine do not exist in Canada [10]. Nevertheless, Aboriginal and treaty rights are protected by the constitution of Canada, and this is reflected in forest policy and forest management practices. Canada's National Forest Sector Strategy (1988, reviewed and revised in 1992, 1998, 2003 and 2008) included provisions for ensuring rights and participation of Aboriginal people and incorporating traditional knowledge, cultural values and practices in managing forest lands [22,169,170]. Involvement of Aboriginal people in developing non-timber forest products and the role they play in sustainable forest management have been recognized [22]. The economic development of NTFPs for diversification of the forest industry is one of the important aspects of sustainable management of Canada's forest [13]. Canada is also an active participant of multilateral and bilateral international treaties and conventions including Forest Principles and CBD adopted in 1992 in Rio, and CITES [22]; ensuring conservation and sustainable management of medicinal plants, as well as protection of indigenous knowledge. Canada has recently - although belatedly - ratified the UN Declaration on the Rights of Indigenous Peoples that reaffirms the country's commitment to promoting and protecting the rights of Indigenous peoples and their resources [171].

*Sustainable Forests: A Canadian Commitment *published in 1992 [172] was signed by governments, industry, non-governmental organizations, Aboriginal people, and communities. It responded to international initiatives and commitments, including the UNCED and Agenda 21. The Canadian government has implemented the Species at Risk Act in 2002 to protect endangered and threatened species [173]. Equivalent legislations also exist at the provincial level [140].

The Natural Health Products Directorate of Health Canada is a governing body for the regulation of plant remedies. The Canadian policy on "Natural Health Products Regulations" includes herbal medicines among other things and was implemented in 2004 by the Natural Health Products Directorate [174,175]. The program has identified indigenous medicinal plants and Aboriginal contributions and approaches to alternative health care as priority research areas [141].

#### Forest Certification and Aboriginal Medicinal Plants of Canada

Forest certification provides important benefits to forest communities and certified forests are increasing in proportion since the beginning of the 1990's. Canada is leading the world in terms of total area of certified forest and proportion of managed forests that have been certified [176]. Certification standards mandate forest companies to protect biodiversity and Aboriginal culture. Specific criteria protect the rights of Aboriginal people, ensure preservation of Aboriginal resources, traditional knowledge and land, and compensation of Indigenous people for the use of their traditional knowledge in forest management [176]. These provisions provided by forest certification standards could benefit Aboriginal people by providing opportunities for protecting not only timber, but also non-timber forest values, including medicinal plants [177]. Interestingly, certification has proven equally, or even more effective than legislation to ensure protection of species, habitats and culture, as pressure from the market is often stronger than from governments.

### Trends, Gaps and Future Directions

Ethnobotanical research in the Canadian boreal forest has so far focused on plant use by Aboriginal people from the eastern boreal zone. The Mi'kmaq and Malecite nations of the Maritimes are among the most studied groups [e.g. [43,48,60-65]], along with the Ojibwa/Chippewa [e.g. [47,84-89]. In Quebec, Cree and Innu cultures have been given more attention [e.g. [42,70-83]]. The northwestern zone of the boreal forest has received less attention [but see [13,17,54,92-99].

After the 1960s, there has been a shift from ethnobotanical studies to phytochemical, antimicrobial and pharmacological studies. Notwithstanding the importance of phytochemical, antimicrobial and pharmacological studies, ethnobotanical efforts should continue, especially in areas and within nations that have received less attention so far, or for which publicly available material is scarce. For example, studies should be conducted in the northwestern Canadian boreal forest, notably with the Metis and with peoples of the Athapaskan language family, as well as with the Naskapi from northeastern Quebec. To diversify the scope of ethnobotanical studies, new methods should be adopted, for field work as well as for data analysis [e.g. [83,178-182]].

Studies are needed to determine if, for the same active principle and at the same dosage, the efficiency is different for traditional and western remedies. Possible interactions between medicinal plants and western medicine or between different species of medicinal plants also need to be investigated [183-185]. Studies are also needed in pharmacognosy, i.e. the standardization, authentication and study of natural drugs [186]. The utilization of animal products (alone or in combination with plants) to treat ailments has been given far less attention than medicinal plants, despite major potential [187].

From a governance point of view, gender-based or intergenerational knowledge differences related to medicinal plant use should be better documented. Studies are also needed to determine how traditional medicine could be given a larger place in modern health care systems [188-192].

Policy issues about traditional medicinal practices have not yet been properly addressed in Canada [10]. The recent adoption of the UN Declaration on the Rights of Indigenous People could provide incentives in this regard. Canada is also lagging behind in terms of regulations about conservation and management of medicinal plants. As suggested by Westfall and Glickman [140], Canada requires an enforceable policy that protects wild medicinal plants, effective monitoring system for commercial harvesting, and policy incentives for the cultivation of medicinal plants in order to reduce harvesting pressure of wild plants. However, harvesting medicinal plants from cultivated fields instead of natural forests might affect spiritual and ceremonial aspects, possibly with reduced medicinal effects [156].

## Conclusion

Medicinal plants represent a significant contribution to human health and one of the most significant ways in which humans directly reap the benefits provided by biodiversity [7,17]. Use of medicinal plants by Aboriginal people from the Canadian boreal forest has a long history [11]. Here we reported on 546 medicinal plant species used in the traditional health care systems of Aboriginal people from the Canadian boreal forest. This is the most comprehensive review to date and it shows striking similarities between medicinal plant uses in different nations. Thus, by triangulation, it is probably still possible to document most of the knowledge, but research should continue, especially in areas or within nations that have received less attention.

## Endnotes

^i ^Only the names of the peoples mentioned in this review are provided. The Algonquian and Athapaskan language families include other peoples not listed here. The names are those that are currently in use and different names were sometimes provided in the older literature.

^ii ^Anishinabe is the correct name for this people and it is how they refer to themselves. Ojibwa and Chippewa are names given to them by other tribes or by non-aboriginal people [see [19]]. The Algonquin also call themselves Anishinabe (or Anicinape) [33].

## Competing interests

The authors declare that they have no competing interests.

## Authors' contributions

HA and NJ designed the study. HA supervised the work. YU, HA and AD carried out the literature search. YU and HA analyzed the data and wrote the manuscript. All authors approved the final version of the manuscript.

## Supplementary Material

Additional file 1 **Medicinal plants used by the Aboriginal people of boreal Canada**. Plants are sorted by scientific name. For each plant, family name, growth habit, vernacular name(s), part(s) used, use(s), and reference(s) are provided. Click here for file

Additional file 2 **Major disease categories and associated medicinal plants used by the Aboriginal people of boreal Canada**. Click here for file
