# Supplementary material for: Traditional use of medicinal plants in the boreal forest of Canada: review and perspectives
Source: J Ethnobiol Ethnomed. 2012 Jan 30;8:7. doi: 10.1186/1746-4269-8-7 (PMC3316145; doi:10.1186/1746-4269-8-7)
Supplement: Additional file 1 — Medicinal plants used by the Aboriginal people of boreal Canada. Plants are sorted by scientific name. For each plant, family name, growth habit, vernacular name(s), part(s) used, use(s), and reference(s) are provided. [file 1746-4269-8-7-S1.DOC]

**Additional file 1: Medicinal plants used by the Aboriginal people of boreal Canada**

| ***Latin name, Botanical family and Growth habit*** | ***Vernacular name(s)*** | ***Part(s) used, Use(s) and Reference(s)*** |
| --- | --- | --- |
| *Abies balsamea* (L.) Mill.  Pinaceae, Tree | balsam fir (Eng.); sapin baumier (Fr.); pikew-ahtik, nupukasik, pīkowāhtik, napakāsīt, nāpukasītuk, nāpukasī (Cree); sta'kwin (Malecite); a'ninandak', ini'nandŏk, ne-naig-wah-dayg, jîngo'b (Ojibwa); kokô^k^wank (Abenaki); cigubi, irimucit (Atikamekw); ilnasht, innâsht (Innu) | **Gum:** Applied over cuts, burns, impetigo, sores, scabs, abscesses, ulcers, grip, insect bites, boils, swellings, infections and cuts as a healer [44; Mi'kmaq: 60, 62; Ojibwa: 84, 87; Algonquians: 63; Algonquin: 68, 69; Atikamekw: 73; Malecite: 65; Innu: 72; Cree: 80; Dene: 17]. Used to stimulate appetite [Innu: 72] or for cough, cuts, constipation and to favour childbirth [Montagnais: 71]. Used as an antiseptic for itching or scabies [Abenaki: 67]. Used to treat sore eyes [Ojibwa: 87], or snow blindness [Cree: 80]. Boiled and drunk as a cough and cold medicine [Ojibwa: 86; Atikamekw: 73; Cree: 80]. Used as an emetic [Algonquin: 75], for menstrual irregularity, or skin problems [Cree: 95]. Burned and the fumes inhaled to treat convulsions [Ojibwa: 47]. Combined with bear grease as a hair ointment [Ojibwa: 47]. Combined with crushed bark to treat soreness of the chest resulting from cold [Algonquians: 63].  **Sap:** Used internally as a remedy for gonorrhea and for cold in the chest [Ojibwa: 44], or as a cure-all drink [Dene: 13]. Used for snow blindness [Cree: 81] or to treat skin disorders, cuts, burns and bee stings [Cree: 96]. Poultice used in arthritis and muscular pain [Cree: 96].  **Branches and needles:** Boiled and mixed with grease to be used against stomachache and constipation, sometimes along with *Lycopodium* [Montagnais: 71]. Dried and mixed with animal fat to be applied to burns [Cree: 81]. Boiled and steam used to treat flu [Dene: 13], cold and sore throat [Cree: 81]. Used as a pillow in sore throat and cough [Cree: 81]. Used for making poultices and as a source of laxative tea for women after childbirth [Algonquin: 69]. Placed upon live coals and the smoke inhaled for colds [Ojibwa: 87] or asthma [Cree: 13]. Tea from tops or combs to cure colic [Mi'kmaq: 43]. Applied to sore areas, or boiled, cooled and rubbed on chest and back to treat asthma [Cree: 81]. Pounded and put on the chest to relieve pain [Cree: 81].  **Cones:** Boiled and the liquid is used as a tea to fight nausea [Montagnais: 71]. Juice used as a laxative, or buds or young cones steeped with bark of young hemlock (*Tsuga canadensis*) and oak (*Quercus rubra*) taken as a cure for diarrhoea [Malecite: 65].  **Bark:** General medicine [Cree: 42], part of a compound applied topically for pain relief, and another compound preparation drunk after childbirth [Cree: 13]. Tea used as a "women's medicine" [Cree: 96]. Used to prepare a decoction to induce perspiration [Ojibwa: 44] or diaphoresis [Algonquians: 63]. Dried bark with pitch blisters ground and mixed with lard to make a poultice applied to infected wounds or for arthritis [Cree: 13], or grated with other plants and the resulting powder mixed with water to make a paste applied to infections and boils [Cree: 13]. Used against anorexia and shiver [Montagnais: 71]. Infusion used for tuberculosis [Cree: 95]. Bark steeped and given in initial stages of gonorrhoea [Algonquians: 63; Malecite: 65]. Boiled and mixed with Caribou wheat (*Eriophorum* sp.) for cancer treatment [Dene: 17]. Poultice used to treat facial paralysis, skin disorders, cuts, burns and bee stings [Cree: 96]. '  **Inner bark:** Eaten to treat stomach problems [Dene: 17]. Boiled to a jelly used for treating burns [Algonquin: 75; Cree: 80], sores and swellings [Algonquians: 63] or drunk as a general tonic for cold, influenza, etc. [Algonquin: 75; Cree, Dene, Metis: 13; Dene: 17]. Used in diabetes [Cree: 82, 83].  **Roots:** Used for heart disease [Algonquin: 69]. Decoction taken for chest cold, pain, and backache, as an emetic and against tuberculosis [Cree: 13]. Decoction sprinkled on hot stones and steam used to ease rheumatic joints [Ojibwa: 47].  **Buds:** Boiled and drunk against "inward disorders" [Cree: 78]. |
| *Abies* *lasiocarpa* (Hook.) Nutt.  Pinaceae, Tree | subalpine fir (Eng.); sapin subalpin (Fr.); tsutsi (Dene) | **Gum:** Poultice used for backpain [Dene: 98]. |
| *Acer negundo* L.  Aceraceae, Tree | box elder (Eng.); érable négondo, érable à Giguère (Fr.); adjagobi'mûk (Ojibwa) | **Inner bark:** Steeped and used as an emetic [Ojibwa: 87]. |
| *Acer nigrum* Michx. f.  Aceraceae, Tree | black maple (Eng.); érable noir (Fr.); ishig'omeaush' (Ojibwa) | **Inner bark:** Decoction used for diarrhoea [Ojibwa: 84]. |
| *Acer pensylvanicum* L.  Aceraceae, Tree | moosewood, striped maple (Eng.); érable de Pennsylvanie, bois barré, bois d'orignal (Fr.); mō^n^'zomĭsh' (Ojibwa); onsé'gak^uk^ (Abenaki) | Medicinal tea [Algonquin: 69].  **Bark:** Steeped and made into a poultice for swelling of the limbs [Algonquians: 63]. Used for gonorrhea, kidney troubles and for blood spitting [Mi'kmaq: 60]. Bark used for bronchial trouble [Abenaki: 67]. Decoction drunk as a laxative [91].  **Inner bark:** Boiled and taken as an emetic [Ojibwa: 84]. |
| *Acer rubrum* L.  Aceraceae, Tree | red maple (Eng.); érable rouge (Fr.); cicigîme'wîc (Ojibwa) | **Bark:** Tea used to wash and cure sore eyes [91, Ojibwa: 87]. |
| *Acer saccharinum* L.  Aceraceae, Tree | silver maple, soft maple, (Eng.); érable argenté (Fr.); innīnâ'tik, sigme-winš (Ojibwa) | **Bark:** Boiled and applied to sores [Ojibwa: 85]. Steeped and applied for chest pain [Mi'kmaq: 62].  **Inner bark:** Decoction used for diarrhoea, or mixed with a *Betula* decoction and used as a diuretic [Ojibwa: 84]. |
| *Acer* *saccharum* Marsh.  Aceraceae, Tree | sugar maple (Eng.); érable à sucre (Fr.); a'nina'tĭg (Ojibwa) | **Sap:** Used as a tonic [55]. Used with a decoction of *Caltha palustris* as a cough syrup [Ojibwa: 88].  **Bark:** Used as a tonic or physic [91]. |
| *Acer spicatum* Lam.  Aceraceae, Tree | mountain maple (Eng.); érable à épis (Fr.); malsuna'u (Malecite); cacagobi'mûk (Ojibwa); webanatuk (Atikamekw) | **Branches:** Pith put into the eye to remove foreign matter, or soaked in water to make a lotion for treating sore eyes [Ojibwa: 87].  **Bark:** Steeped in water and the water used to cure eyes [Malecite: 65].  **Roots:** Boiled and applied to wounds and abscess [Atikamekw: 73]. |
| *Achillea millefolium* L.  Asteraceae, Herb | yarrow, milfoil (Eng.); achillée millefeuille, herbe à dindes (Fr.); wapunewusk, wapanowask, wapānaskiy(h)k, astēskotawān, astawēskōtawan, miskigonimaski, oskānīmaskīgī, osgunīmasgigah, wīpōwānīwisikwān, kāwāpistikwānīkāpawik, āmowask (Cree); t'a'^n^chaydelgai (Chipewyan); sa'pagol (Malecite); a'djidamo'wano, adjidamo'anûk (Ojibwa); namahiba'go (Abenaki); teskwemaskiki (Atikamekw); kâuâpishtukuâniâshiti tshishiteu-nîpîsha (Montagnais); at''àn dagàii (Dene) | **Whole plant:** Used to stop bleeding [Dene: 13; Cree: 93]. Boiled until thick and used as a liniment [Malecite: 65]. Used for fever, cold and other respiratory disorders [Mi'kmaq: 43; Atikamekw: 73; Abenaki: 67; Algonquin: 69; Montagnais/Innu: 60, 71, 72]. Dried, mashed into a powder, and rubbed with leaves or bark on swelling, bruise, or sprain [Mi'kmaq: 43, 61]. Mixed with lard and applied to infected sores [Cree: 13]. Decoction used to treat diarrhoea [Cree: 13]. Soaking in a bath of hot water and *Achillae millefolium* helps treat arthritis or aching bones, and a twice-boiled decoction of the plant can be drunk after the bath [Cree: 13]. Boiled and wrapped on a sore body or used to wash the body to treat pain [Dene: 17]. Tea for cough and ulcers [Dene: 99] or stomach problems [Cree: 96]. Boiled and taken to treat diabetes [Cree: 93].  **Above-ground parts:** Poultice used to treat headaches including migraines, arthritis, muscular pain, sore back, or body pain [Cree: 13; 96]. Tea drunk as a painkiller [Cree: 13] as well as for sore throat, cough and cold [Cree: 96]. Decoction drunk to treat sore chest [Cree: 13]. Poultice or tea used to treat colds, headaches, to stop bleeding or to relieve a sore throat, itchiness and other skin problems [Dene: 100].  **Leaves:** Dried and boiled to make a decoction to treat diabetes [Cree: 13]. Crushed and used as a snuff for headaches, or boiled and the steam inhaled and the decoction drunk, or flower and leaves are burned and the smoke inhaled [Chippewa: 47; Cree: 13; Atikamekw: 73; Algonquin: 69]. Chewed and applied to burns, cuts or bee stings [Algonquin: 68; Cree: 13]. Infusion used as a wash for pimples, mosquito bites or other skin eruptions [Chippewa: 47; Dene: 13; Chipewyan: 92]. Poultice used in skin disorders, cuts, burns and bee stings [Cree: 96].  **Roots:** An ointment rubbed on sores, aching bones or swellings to relieve the pain, and a decoction of the same mix is drunk to help reduce swelling [Cree: 13]. Seeds and roots are boiled and the steam used to treat sore eyes [Cree: 13].  **Flowers:** Burned (sometimes with leaves) and the smoke used to fumigate a room in which someone is sick [Dene: 13]. Florets placed on a bed of coals and smoke inhaled to break a fever [Ojibwa: 87]. Fresh flower heads chewed and applied to bee stings, cuts, sores [Cree, Metis: 13] or placed in the nostril to stop a nosebleed [Cree: 13, 93]. Decoction drunk as a spring tonic, to regain lost appetite, to treat menstrual cramps or heavy menstruation, to aid in childbirth to relieve labor pains and to stop haemorrhaging [Cree, Dene: 13]. Used to treat sinus or chest congestion [Cree: 13]. Dried flower heads boiled and the decoction used as a wash for skin rashes or sores [Cree, Metis: 13]. Dried and smoked for headache [Chipewyan: 92]. Boiled and drunk to prevent nosebleed, the liquid can also be used to soothe infected skin and sunburns, or rashes, including eczema [Dene: 99]. Poultice used in skin disorders, cuts, burns and bee stings [Cree: 96]. Boiled and drunk to treat cough or liver ailments [Dene: 98]. |
| *Achillea millefolium* ssp. *lanulosa* (Nuttall) Piper var. *lanulosa*  **Syn.:** *A.* *millefolium* L. var. *borealis* (Bong.) Farw.; *A. borealis* Bong.  Asteraceae, Herb | boreal yarrow, Siberian yarrow (Eng.); achillée laineuse (Fr.); at''àn dagàu (Dene) | **Above-ground parts:** Poultice or tea used to treat cold, headache, to stop bleeding or to relieve sore throat, itchiness and other skin ailments [Dene: 100]. |
| *Achillea* *millefolium* L. var. *occidentalis* DC.  **Syn.**: *A. lanulosa* Nutt.  Asteraceae, Herb | western yarrow (Eng.); achillée laineuse (Fr.); wīcipōwānīwistikwān, kāwāpistikwānīkāpawik (Cree); wa'bîgwûn (Ojibwa) | **Whole plant:** Used in bruises, swellings and sprains [Mi'kmaq: 61].  **Roots:** Decoction taken for teething related sickness [Cree: 95].  **Flowers:** Crushed with *Mentha arvensis*, wrapped in a cloth and dipped in water to clean pus from the gums of a teething child [Cree: 95].  **Leaves:** Poultice to cure the bite of a spider [Ojibwa: 87]. |
| *Achillea sibirica* Ledeb.  Asteraceae, Herb | Siberian yarrow (Eng.); achillée de Sibérie (Fr.); wīcipōwānīwistikwān, kāwāpistikwānīkāpawik, āmowask (Cree) | **Roots:** Decoction with other herbs taken internally for sickness realted to teething, chewed root applied to sores on the gums [Cree: 95].  **Above-ground parts:** Tea used in sore throat, cough and cold [Cree: 96].  **Flowers:** Boiled and drunk to treat cough and liver ailments [Dene: 98]. |
| *Aconogonon alaskanum* (W. Wight ex Hultén) Soják var. *alaskanum* **Syn.:** *Polygonum* *alpinum* All.; *P. alaskanum* W. Wight ex Hultén  Polygonaceae, Herb | Alaska wild rhubarb (Eng.); renouée d'Alaska (Fr.)ts'u gyùu (Dene) | **Leaves:** Used on burns [Dene: 100]. |
| *Acorus* *americanus* (Raf.) Raf.  Araceae, Herb | American sweetflag (Eng.); acore d'Amérique (Fr.) | **Rhizome:** Poultice used to treat skin disorders, bee stings, cuts and burns, headache, arthritis, muscular aches and pains. Sucked or drunk as tea to treat sore throat, cough and cold. Tea used in diabetes, fever, heart problems and as a wash to treat facial paralysis [Cree: 96]. Boiled and taken to treat diarrhoea, high blood pressure, sore throat, flu, cold, headaches, and pain [Cree: 93]. |
| **Acorus calamus* L.  Araceae, Herb | sweet flag (Eng.); acorus roseau, belle-angélique (Fr.); wika (Chippewa); wachuskomechiwin, wachuskowmiytsuwin, wacaskōmīcowin, wacaskwatapih, wiy(h)kiyuw, wihkes, wīhkes, wihkis (Cree); dzëñni (Chipewyan); kiuhu'ziwazas (Malecite); wiikansh, na'bûgûck , wikĕn', na'bugûck' (Ojibwa); môskwas'wask (Abenaki) | Medicinal plant [Malecite: 59], componenent of many compound medicines used to treat venereal disease [Metis: 13], cough and cold [Cree: 42; Malecite: 65], heart trouble, headache, fever, infected wounds, muscle or joint pain including rheumatism [Cree: 13].  **Root:** Scalded (not boiled) and used as a physic [Chippewa: 47]. Used to cure a cold in the throat [Chippewa: 47; Ojibwa: 87; Algonquin: 69], fever, tootache, earache, headache [Chippewa: 47; Ojibwa: 89] or for stomach cramps [Ojibwa: 87]. Used to cure open wounds [Mi'kmaq: 43]. Combined with pepperroot (*Dentaria diphylla*) given for heart disease and for coughs combined with cherry (*Prunus virginiana*) [Algonquin: 69]. Tea given to women after childbirth and used to treat the symptoms of menopause, or combined with *Sorbus americana* and used as a tonic [Algonquin: 69]. Chewed and juice swallowed for cold, sore throat and chest congestion, or powder used to treat chest infection [Dene: 17].  **Rhizome:** Used as a tonic, to treat cholera, chewed against pharyngitis or for clearing the throat, an infusion is used by women in dysmenorrhea [Algonquians: 63; Chippewa: 88]. Chewed for cough [Mi'kmaq: 60]. Combined with the bark of *Xanthozylon americanum*, the bark of the roots of *Sassafras variifolium* and roots of *Asarum canadense* and steeped to make a remedy for cold, cough, and bronchial troubles [Chippewa: 85]. Carminative [Abenaki: 67], a decoction is drunk for fever and cold [44; Chippewa: 47; Cree, Dene, Metis: 13]. Pulverized and snuffed up nostrils in colds [Chippewa: 47]. Chewed and juice or decoction drunk to treat cold, cough including whooping cough, sore throat, sinus congestion, blood spitting, dry mouth, upset stomach, toothache, teething pain, rash, headache, rhumatism, muscle pain, chest pain, lower back pain, pounding heart, tonsillitis, pneumonia, diabetes, venereal disease or intestinal worms [44; Cree: 95; Cree, Dene, Metis: 13]. Chewed to treat tonsils, diabetes [Cree, Dene: 13], or to relieve fatigue on a long hike [Dene: 13]. Smoked, or chewed and the juice swallowed (or rarely boiled to make a drink) to treat cough and cold, sore throat, stomach ache, toothache, or pain. Mixed with stipe bases of *Dryopteris spinulosa*, roots of *Aster puniceus* and stems of *Sorbus scopulina*, boiled and drunk to relieve sore kidneys or other "internal pain". Combined with stipe bases of *Dryopteris spinulosa* and beaver castor and used as a heart medicine. Boiled with *Amanita muscaria* to make a wash for sore eyes [Chipewyan: 92]. Steam from boiling inhaled to relieve congestion, headache, or earache [Cree: 13]. Small piece softened in water and inserted into the ear or grinded, boiled, and mixed with flour to make a batter to make a compress placed over affected ear [Cree: 13]. Smoked to treat a cough or boiled to make a wash for sore eyes [Dene: 13]. Dried and grinded or freshly grated chewed and used as a poultice to treat headache and painful joints from arthritis, muscle cramps, a decoction is drunk to aid the healing [Cree: 13]. Powdered with other herbs (*Nuphar variegatum* or *Heracleum lanatum*) applied externally to treat headache, swelling of limbs, painful joints, muscle pain, and rheumatism, or chewed for cough, cuts, toothache, earache, bellyache, or facial paralysis [Cree: 95]. Cough medicine [Dene: 98]. |
| *Actaea* *pachypoda* Elliot  **Syn.:** *A. alba* (L.) Mill.  Ranunculaceae, Herb | white baneberry (Eng.); actée à gros pédicelles (Fr.); wapkadak (Ojibwa) | Used against menstrual disorders [Algonquin: 68].  **Roots:** Decoction given as a remedy for convulsions in both children and adults [Ojibwa: 85], or to slow excessive blood flow from menstruation, childbirth or wounds [Metis: 13]. |
| *Actaea* *racemosa* L. var. *racemosa*  **Syn.:** *Cimicifuga* *racemosa* (L.) Nutt.  Ranunculaceae, Herb | cohosh root (Eng.); cimicaire à grappes (Fr.) | **Roots:** Used for kidney troubles [Algonquians: 63]. |
| *Actaea* *rubra* (Aiton) Willd.  Ranunculaceae, Herb | red baneberry (Eng.); actée rouge, poison de couleuvre (Fr.); maskōminānātik (Cree); odzī'bĭkĕ^n^s', wi'cosidji'bĭk, wîckobidji'bîk (Ojibwa) | **Whole plant:** Used in a purgative tea [Cree: 42; Metis: 13].  **Roots:** Decoction made with 20 other plants and drunk to treat nose bleeding [Dene: 13]. Mixed with spruce branch tips in a decoction taken to treat stomach problems such as indigestion or constipation [Ojibwa: 87; Metis: 13]. Decoction for stomach pain [Ojibwa: 44, 84; Penobscot: 44]. Decoction or infusion given to slow heavy menstrual flow [Ojibwa: 47; Cree: 95]. Tea drunk after childbirth [Ojibwa: 87]. |
| *Adiantum pedatum* L.  Pteridaceae, Herb | maidenhair fern (Eng.); adiante pédalé, capillaire du Canada (Fr.) | Plant steeped for curing fits [Algonquians: 63]. |
| *Agastache foeniculum* (Pursh) Kuntze  **Syn.:** A.anethiodora (Nutt.) Britton  Lamiaceae, Herb | giant hyssop (Eng.); agastache fenouil (Fr.); kā-wīkīpakahk (Cree); weza'wûnûckwûk' (Ojibwa) | **Whole plant:** Dried and boiled with another herb to make a decoction drunk to treat stomachache [Cree: 13].  **Above-ground parts:** Tea used in stomach problems [Cree: 96].  **Stem and leaves:** Mixed with other plants to make an infusion or decoction against blood spitting [Cree: 95]. Used for cough [Dene: 13]. Poultice applied to burns [Ojibwa: 47].  **Flowers:** Chewed as a breath freshener [Cree: 95]. Poultice made with *Solidago altissima* and *Rudbeckia laciniata* applied to burns [Ojibwa: 47].  **Roots:** Infusion for cold, chest pain, and cough [Ojibwa: 47]. |
| *Agrimonia* *gryposepala* Wallr.  Rosaceae, Herb | agrimony (Eng.); aigremoine à sépales crochus (Fr.); saga'tîgans (Ojibwa) | **Roots:** Used for urinary troubles [Ojibwa: 87]. |
| *Aletris* *farinosa* L.  Liliaceae, Herb | unicorn root, colicroot (Eng.); alétris farineux (Fr.) | **Roots:** Decoction used as a tonic and emmenagogue, and for stomachache [44]. |
| **Alisma plantago-aquatica* L.  Alismataceae, Herb | broad-leaved water plantain (Eng.); alisma commun (Fr.); mitīhīmaskīhkīh (Cree) | **Stem:** Powdered stem base used as an ingredient in a many-herb remedy to treat various ailments [Cree: 95]. Dried stem base eaten directly or grated and taken in water for heart troubles, stomachache, cramps and stomach flu, constipation, prevent fainting during childbirth [Cree: 95]. |
| *Allium stellatum* Fraser ex Ker Gawl.  Liliaceae, Herb | wild onion (Eng.); ail étoilé (Fr.); mûckode'cigaga'wûnj (Ojibwa) | Boiled to obtain syrup taken to treat sore throat [55].  **Roots:** Decoction used for cold [Ojibwa: 47]. |
| *Allium* *tricoccum* Aiton  Liliaceae, Herb | wild leek (Eng.); ail des bois (Fr.); siga'gawûnj' (Ojibwa) | **Roots:** Decoction used as an emetic [Ojibwa: 47]. |
| Alnus incana (L.) Moench ssp. rugosa (Du Roi) R.T. Clausen  **Syn.:** A. rugosa (Du Roi) Spreng.  Betulaceae, Tree | speckled alder, mountain alder (Eng.); aulne rugueux (Fr.); wadûb', wado'bîn (Ojibwa); mithkwatō(h)spī, atōspī, oto'pé, wdopi (Abenaki); atûshpî (Montagnais) | **Bark:** Used as a laxative [Cree: 95], astringent and emetic [Ojibwa: 88]. Steeped with tamarack bark for anemia [Ojibwa: 85].Chewed in ulcerated mouth [Malecite: 65]. Shavings used as a poultice for sores or applied warm to swollen areas [Ojibwa: 85]. Decoction of shavings used as wash for skin cancer, drunk for leukemia together with *Salix* sp., or for anemia with *Prunus virginiana* and *Rubus idaeus* [Ojibwa: 85].  **Inner bark:** Tea used as an emetic, as a laxative, and in liver disorders [Algonquin: 69]. Decoction to wash sore eyes [Cree: 95]. Boiled to yield a red liquid used in skin troubles [Abenaki: 67; Montagnais: 71]. Decoction of *Alnus incana* and *Viburnum acerifolium* taken as an emetic [Ojibwa: 47]. Used in diabetes [Cree: 77, 82, 83].  **Twigs and stems:** Decoction of branches as wash for burns or sore mouth and decoction of stem used for ulcers or bleeding ulcers [Ojibwa: 85].  **Roots:** Hemostatic [Ojibwa: 87]. Decoction taken to ease labor in childbirth [Ojibwa: 47]. Decoction made from equal parts of roots of *Cornus alternifolia, Cornus sericea* and *Alnus incana* used as a wash or compress to treat sore eyes [Ojibwa: 47]. Tea used for blood stools as an astringent and coagulant [Ojibwa: 87]. Bark scraped off the root and mixed with molasses for toothache [Algonquin: 69]. |
| *Alnus rubra* Bong.  Betulaceae, Tree | red alder (Eng.); aulne rouge (Fr.) | **Bark:** Steeped and drunk to stop cholera [Montagnais: 70]. |
| *Alnus* sp.  Betulaceae, Tree | alder (Eng.); aulne (Fr.) | Boiled, mixed with porcupine fat, and taken as a physic [Mi'kmaq: 43].  **Bark:** Boiled, then water is used to stop cramps and retching [Algonquians: 63], whereas pulp is used as a poultice for sore eyes [Algonquin: 75]. Steeped in water and taken for fever, stomach cramps, kidney problems, and asthma [Mi'kmaq: 62]. Tea used in diphtheria [Mi'kmaq: 43].  **Leaves:** Used in fits [Mi'kmaq: 62].  **Bark and leaves:** Used to cover body to treat fever or festers [Mi'kmaq: 43].  **Twigs:** Boiled and drunk to purify blood [Mi'kmaq: 60]. |
| *Alnus* *viridis* (Chaix) DC.  Betulaceae, Tree | green alder (Eng.); atōspīah, atōsbīah, atōspī, māthatōspī, mithkwatōspi, miskwatōspi, mihkwatōspi (Cree) | Decoction used in a steam treatment to trigger menstruation [Cree: 95].  **Bark:** Used in dropsy [Cree: 42].  **Flowers:** Green female catkins boiled to make a medicinal tea for treating venereal disease in men [Dene: 13].  **Stem:** Boiled to make an emetic for treating an upset stomach [Dene: 13].  **Roots:** Decoction drunk to relieve menstrual cramps [Dene: 13], or used to treat scalding from boiling water [Cree: 13]. |
| *Alnus* *viridis* (Chaix) DC. ssp. *crispa* (Aiton) Turrill  **Syn:** *Alnus* *crispa* (Aiton) Pursh  Betulaceae, Tree | green alder (Eng.); aulne crispé (Fr.); k'áilisën (Chipewyan); māthatō(h)spī (Cree); shakâu tshîtshue (Montagnais); k'oh (Dene) | Decoction used in a steam treatment to trigger menstruation [Cree: 95]. Tea used to treat stomach pain [Dene: 100].  **Cones:** Boiled to make a tea to treat venereal disease in men [Chipewyan: 92].  **Stem:** Tea taken for stomach pain [Chipewyan: 92].  **Inner bark:** Tea used against diarrhoea [Montagnais: 71].  **Bark:** Boiled and drunk to treat stomach ache, or cooled and rubbed on skin to heal sores, scabs, eczema, insect bites, sunburns, rashes and aching joints [Dene: 99].  **Buds:** Chewed and juice swallowed for cold. Boiled and drunk for cold or applied to sores, or used for bathing to soothe eczema and rashes [Dene: 99].  **Roots:** Mashed and eaten to help with stomach ache [Dene: 99].  **Leaves:** Chewed or crushed and put on bee stings [Dene: 100]. |
| *Amanita muscaria* Fr.  Amanitaceae, Fungi | fly agaric (Eng.); amanite tue-mouche (Fr.) | Boiled with other plants to make eye-drops for sore eyes [Dene: 13]. |
| *Amelanchier alnifolia* (Nutt.) Nutt ex M. Roem.  Rosaceae, Shrub | Saskatoon serviceberry, juneberry (Eng.); amélanchier à feuilles d'aulne, petites poires (Fr.); misaskatoomena, misakwatōminatik, msāskwatuwmin, saskwatoomina, saskwatōmin (Cree); k'ęàjie (Dene) | **Roots and stem:** Decoction drunk to treat lung problems including tuberculosis [Cree: 13].  **Roots:** Decoction with other herbs given to children to stop diarrhoea [Cree: 13]. Decoction used in teething, chest pain, cough, and lung infection [Cree: 95]. Tea used in stomach problems [Cree: 96]. Herbal water used for back paralysis [Cree: 93].  **Buds:** Decoction drunk to slow diarrhoea [Cree: 13]. Tea used in stomach problems [Cree: 96].  **Stem:** Decoction mixed with *Symphoricarpos albus* against fever [Cree: 95]. Tea drunk to cure urinary problems [Dene: 101].  **Berries:** Tea drunk for whooping cough. Berry buds boiled and tea drunk to cure headache, stomach pain and diarrhoea [Dene: 101]. |
| *Amelanchier* *bartramiana* (Tausch) M. Roem.  Rosaceae, Tree | Bartram's shadbush (Eng.); amélanchier, de Bartram (Fr.); atûminânakashî (Montagnais) | **Bark:** Boiled and the preparation applied to wounds or taken as a tea to reduce cough [Montagnais: 71]. |
| *Amelanchier* *canadensis* (L.) Medik.  Rosaceae, Tree | shadbush, serviceberry (Eng.); amélanchier du Canada (Fr.); gizigwa'komĭnaga'wûnj (Ojibwa) | **Roots:** Steeped and taken to slow heavy menstrual flow [Ojibwa: 47]. Decoction combined with roots of cherry and young oak taken to treat dysentery; [Ojibwa: 47].  **Bark:** Decoction combined with pin cherry, choke cherry and wild cherry taken to treat "female diseases" [Ojibwa: 47].  **Inner bark:** Decoction used as a disinfectant [Ojibwa: 47]. |
| *Amelanchier* *laevis* Wiegand  Rosaceae, Tree | smooth juneberry, glabrous shadbush, (Eng.); amélanchier glabre, petites poires (Fr.); goziga'gominaga'wûnj (Ojibwa) | **Bark:** Tea for the expectant mother [Ojibwa: 87]. |
| *Amorpha* *canescens* Pursh  Fabaceae, Shrub | leadplant (Eng.); faux indigo (Fr.); we'abŏnag'kak (Ojibwa) | **Roots:** Decoction for stomachache [Ojibwa: 84]. |
| *Amphicarpaea* *bracteata* (L.) var. *comosa* (L.) Fernald  **Syn.:** *Falcata* *comosa* (L.) Kuntze  Fabaceae, Vine | hogpeanut (Eng.); amphicarpe bractéolée (Fr.) | **Roots:** Decoction combined with other plants taken as a physic [Ojibwa: 47]. |
| Anaphalis margaritacea (L.) Benth.  Asteraceae, Herb | pearly overlasting (Eng.); anaphale marguerite, immortelle (Fr.); wa'bigwûn, basi'bagûk (Ojibwa); kawapukanik (Atikamekw) | Plant decoction used for coughing and consumption [Algonquians: 63]. Poultice used against burns [Algonquin: 68].  **Flowers:** Decoction used in paralysis [Ojibwa: 47]. Powder sprinkled on live coals and inhaled by a person who has a stroke of paralysis [Ojibwa: 87].  **Leaves:** Boiled and applied to burns and skin problems [Atikamekw: 73]. |
| *Andromeda polifolia* L. var*. latifolia* Aiton  **Syn:** *Andromeda* *polifolia* L. var. *glaucophylla* (Link) DC.  Ericaceae, Shrub | glaucous-leaved bog rosemary (Eng.); andromède glauque (Fr.); kakouboushk (Cree) | **Branches:** Used to treat diabetes [Cree: 82].  **Stem and roots:** Boiled and drunk to treat stomach problems [Dene: 98]. |
| *Androsace* *septentrionalis* L.  Primulaceae, Herb | northern fairy-candelabra (Eng.); androsace septentrionale (Fr.) | **Whole plant:** Decoction used to wash hair or any body part to kill lice [Dene: 98]. |
| *Andropogon* *gerardii* Vitman  **Syn.:** *Andropogon* *furcatus* Muhl. ex Willd.  Poaceae, Herb | big bluestem (Eng.); barbon de Gérard (Fr.); mûckode'kanĕs (Ojibwa) | **Roots:** Decoction taken for stomach pain [Ojibwa: 47]. Decoction, alone or with *Symphoricarpos albus,* used to treat stoppage of urine [Ojibwa: 47]. |
| *Anemone canadensis* L.  Ranunculaceae, Herb | Canadian anemone (Eng.); anémone du Canada (Fr.); wabesgung, mîdewidji'bîk (Ojibwa) | **Roots:** Chewed slowly to a pulp and laid on a wound, serves as a styptic [Ojibwa: 85]. Steeped to make a wash for obstinate, scabby sores [Ojibwa: 85]. Eaten to clear the throat [Ojibwa: 87]. Decoction used for pains in the lumbar region [Ojibwa: 84].  **Leaves:** Bruised and placed in nostril to stop nasal hemorrhage, or used as a styptic [Ojibwa: 85]. |
| *Anemone* *cylindrica* A. Gray  Ranunculaceae, Herb | thimble-weed (Eng.); anémone cylindrique (Fr.); gande gwa'sonînke' (Ojibwa) | **Roots:** Tea used to relieve lung congestion and tuberculosis [Ojibwa: 87]. |
| Anemone multifida Poir.  **Syn.:** A.globosa (Torr. & A. Gray) Nutt. ex Pritz.  Ranunculaceae, Herb | wind-flower (Eng.); anémone multifide (Fr.) | Used against headache [Algonquin: 68]. |
| *Anemone* sp.  Ranunculaceae, Herb | anemone (Eng.); anémone (Fr.); wisŏg'ibŏk' (Ojibwa) | **Leaves:** Dried, powdered and used as an errhine, against headache [Ojibwa: 84]. |
| *Antennaria* *howellii* Greene ssp. *neodioica* (Greene) Bayer  **Syn.:** *A*.*neodioica* Greene  Asteraceae, Herb | lesser cat's-foot (Eng.); antennaire néodioïque, immortelle (Fr.); gagîge'bûg (Ojibwa) | **Whole plant:** Tea given to a mother after childbirth [Ojibwa: 87]. |
| Antennaria neglecta Greene  Asteraceae, Herb | lesser pussytoes (Eng.); antennaire négligée (Fr.); gagîge'bûg (Ojibwa) | Infusion given as a gynecological aid after childbirth [Ojibwa: 19]. |
| *Antennaria* *plantaginifolia* (L.) Richardson  Asteraceae, Herb | woman's tobacco (Eng.); antennaire à feuilles de plantain (Fr.) | Used for stomachache and as an expectorant [Ojibwa: 19]. |
| *Apocynum androsaemifolium* L.  Apocynaceae, Herb | spreading dogbane (Eng.), apocyn à feuilles d'androsème, herbe à puce, gobe-mouches (Fr.), tōtōsāpowask (Cree); ma'kwona'gĭc odji'bĭk, wesa'wûckwûn, sasa'bikwan (Ojibwa) | **Whole plant:** Tea given to increase lactation in nursing mothers, and cooled tea used as eyewash to treat sore eyes from smoke or snow blindness [Metis: 13; Cree: 95].  **Roots:** Dried, pulverized and snuffed up the nostrils, or put on hot stones and the fumes are inhaled, against headache. Powdered, moistened with lukewarm water and applied to incisions on the temples to treat headache. Decoction used to treat heart palpitation. Very weak decoction taken internally to treat cold. Cotton moistened with decoction used to stop nostril bleeding, or in severe cases mashed roots are used as a plug. Decoction poured into ear to treat soreness [Ojibwa: 47]. Placed upon live coals and the incense inhaled for throat trouble [Ojibwa: 87].  **Stalk and roots:** Steeped to make a tea for women to drink to keep the kidneys free during pregnancy [Ojibwa: 87]. |
| *Apocynum* *cannabinum* L.  **Syn.:** *A. hypericifolium* Aiton  Apocynaceae, Herb | Indian hemp (Eng.); apocyn chanvrin (Fr.) | Medicinal plant [Cree: 42]. Steeped in water and administered to expel worms [Algonquians: 63]. |
| *Aquilegia* *canadensis* L.  Ranunculaceae, Herb | wild columbine (Eng.); ancolie du Canada (Fr.) | **Roots:** Used for stomach troubles [Ojibwa: 87]. |
| *Aralia hispida* Vent.  Araliaceae, Herb | bristly sarsaparilla, dwarf-elder (Eng.); aralie hispide, salsepareille (Fr.); nishitshikâta (Montagnais) | **Roots:** Used in heart disease [Algonquin: 69].  **Root bark:** Scraped and dried before being used to prepare a tea for cough [Montagnais: 71]. |
| *Aralia nudicaulis* L.  Araliaceae, Herb | wild sarsaparilla (Eng.); aralie à tige nue, salsepareille (Fr.); ba-gwa-nan, wabos'odji'bĭk, bebamabi'k (Ojibwa); wāposōcēpīhk, wāposocīpihk, wāpōsogībī (Cree); gajíé (Chipewyan); wapacak (Atikamekw); saçat'sek, sasôgsek (Abenaki) | Tea used as a blood medicine, for fainting and fits [Ojibwa: 86], or as a tonic [Abenaki: 67]. Decoction (excluding fruit) used to treat pneumonia in children [Cree: 95].  **Leaves:** Boiled with stems of *Sorbus scopulina* and *Sarracenia purpurea* to make a tea taken to relieve chest pain [Chipewyan: 92].  **Roots:** Used as a diuretic and alterative [Cree: 74]. Dried and crushed to a powder and steeped with sweet flag for cough, steeped and taken in weakness. Boiled in a box with hot stones, and decoction taken internally for stomachache, or merely as a beverage [Algonquians: 63]. Chewed and inserted in an aching ear [Atikamekw: 73]. Pounded in a mortar, boiled in hot water and used for blood purification during pregnancy [Ojibwa: 87]. Used as stimulant [Ojibwa: 88]. Fresh root pounded and applied as a poultice to bring a boil to a head or to cure a carbuncle [Ojibwa: 87]. Decoction used as a remedy for blood problems, or applied to a sore. Dried and powdered, or fresh root chewed and inserted in nostril to stop nose bleeding. Decoction of stalk of *Ribes triste*, root of *Aralia racemosa* and root of *Aralia nudicaulis* taken for amenorrhoea [Ojibwa: 47]. Powdered, steeped in water and used to treat cold and influenza [Mi'kmaq: 62]. Powdered and used in many-herb remedy to treat various ailments. Decoction taken internally for teething [Cree: 95]. Poultice used to treat infected wounds [Cree: 13, 93]. Tea for kidney disorders [Algonquin: 69].  **Fruiting stalk:** Decoction used to stimulate lactation. [Cree: 95].  **Rhizomes:** Chewed or made into a tea to treat heart pain, chronic chest pain, upset stomach, liver problems [Dene: 13; Chipewyan: 92] and sore throat [Cree: 13]. Poultice used to treat skin disorders, bee stings, cuts and burns [Cree: 96]. |
| *Aralia racemosa* L.  Araliaceae, Herb | spikenard, petty morel, life-of-man (Eng.); aralie à grappes, grande salsepareille, anis sauvage (Fr.); či-kadak, o'kadak' (Ojibwa); skidjinawi'widjp'k' (Malecite) | Small quantity of this plant with small quantities of *Pyrola uliginosa, Baptisia tinctoria, Galium aperine, Streptopus amplexifolius, Acer pennsylvanicum* and *Eupatorium perfoliatum* used for gonorrhea, kidney troubles and for blood spitting [Algonquians: 63]. Boiled and applied to wounds [Mi'kmaq: 43].  **Roots:** Infusion used as medicine [Ojibwa: 86]. Used to make poultices for boils [Chippewa: 85]. Tea used to treat tuberculosis, or combined with *Euphorbia* spp. to treat diabetes [Algonquin: 69]. Mixed with *Cornus stolonifera* and smoked against headache. Steeped and taken in consumption, against gonorrhoea, or mixed with black snakeroot in kidney trouble [Malecite: 65]. Grinded and taken with water for cold, cough and sore throat [Mi'kmaq: 62]. Steeped in water and drunk for cold [Mi'kmaq: 62]. Decoction taken in cough Poultice applied to boils, fractures, sprain or strained muscles [Ojibwa: 47] or to the feet in general dropsy [44]. Decoction of stalk of *Ribes triste*, root of *Aralia racemosa* and root of *Aralia nudicaulis* taken for amenorrhoea [Ojibwa: 47]. Rhizome/root used as a carminative as well as an expectorant and antiseptic in cough, chest pain and mortification [44].  **Fruits:** Juice and oil of the seeds poured into the ears to treat deafness [44]. |
| **Arctium lappa* L.  Asteraceae, Herb | burdock (Eng.); bardane majeure (Fr.) | Used as blood medicine [Ojibwa: 86].  **Roots:** Crushed with buds of *Populus balsamifera* and applied to sores [Malecite: 65].  **Buds:** Steeped and used in chancre [Malecite: 65]. |
| **Arctium minus* Bernh.  Asteraceae, Herb | burdock (Eng.); bardane mineure (Fr.); wi'sûgibûg', gi'masan, wi'sûgibûg (Ojibwa); saga'dabohag, oné'bag (Abenaki) | For fever, headache and rheumatism [Abenaki: 67].  **Roots:** Mashed, heated and used as a poultice for boils and absceses [Algonquians: 63]. Used as a tonic or as part of a medicine for stomachache [Ojibwa: 87].  **Leaves:** Infusion taken in cough [Ojibwa: 47]. |
| *Arctostaphylos* *alpina* (L.) Spreng.  Ericaceae, Shrub | alpine bearberry (Eng.); busserole alpine (Fr.); sah-gah-go-me-nah-gah-shen, bi-gwa-dji-mi-squa-bimag (Ojibwa) | Infusion used as a wash for rheumatism and general illnesses [Ojibwa: 19].  **Bark:** Decoction used for blood problems [Ojibwa: 19].  **Leaves:** Smoked to induce intoxication [Ojibwa: 86]. |
| *Arctostaphylos uva-ursi* (L.) Spreng.  Ericaceae, Shrub | bearberry (Eng.); raisin d'ours (Fr.); kinnikinnick (Algonquian); āchiygasipuk, muskimina, muskominanatik, pithīkōmin, kinnikinick (Cree); dé(lh)ni (Chipewyan);  saga'komĭnagûnj', me-squah-be-mag (Ojibwa) | **Whole plant:** Mixed with that *Vaccinium myrtilloides* in a tea taken to trigger menstruation [Cree: 95].  **Stem:** Mixed with *Vaccinum myrtilloides* in a decoction taken to prevent miscarriage and speed up recovery after childbirth [Cree: 95].  **Stem, leaves and fruits:** Decoction drunk for pain in the back and sprained back [44]. **Leaves and fruits:** Tonic [Mi'kmaq: 62].  **Leaves:** Decoction drunk to treat bladder and kidney problems [Metis: 13]. Dried and pulverized and combined with tobacco or red willow and smoked with a pipe to treat headache [Chippewa: 47] or intoxication [Ojibwa: 86].  **Fruits:** Mixed with grease and taken against diarrhoea [Cree: 95].  **Roots:** Tea drunk to treat a persistent cough [Dene: 13; Chipewyan: 92]. Mixed with herbs and given in excessive menstrual bleeding [Cree: 95].  **Bark:** Tea with bark and root of *Salix discolour, Pinus strobus,* *Quercus rubra* and *Pinus banksiana* given in fainting and fits [Ojibwa: 86]. |
| *Arisaema triphyllum* (L.) Schott.  Araceae, Herb | Indian turnip, jack-in-the-pulpit (Eng.); petit prêcheur (Fr.); pogdjinsgwewiwazis (Malecite); sag-a-ba (Mi'kmaq); caca'gomîn (Ojibwa) | General medicine [Ojibwa: 86]. Steeped to make a liniment for external use [Mi'kmaq: 44; Algonquians: 63]. Scraped, pounded, moistened and applied as a poultice on abscesses and boils [Malecite: 65].  **Root bulbs:** Used to treat tuberculosis and as a general stomach medicine [Mi'kmaq: 62].  **Roots:** Decoction used to treat sore eyes [Ojibwa: 47, 86]. |
| *Aristolochia serpentaria* L.  Aristolochiaceae, Herb | virginia snake-root (Eng.); serpentaire de Virginie (Fr.) | **Roots:** Steeped and used for fits [Algonquians: 63]. |
| *Arnica angustifolia* Vahl  **Syn:** *Arnica* *alpina* (L.) Olin  Asteraceae, Herb | alpine arnica (Eng.); arnica à feuilles étroites (Fr.); at'an tsoo (Dene) | **Flowers:** Petals mixed with grease or oil to make an ointment used to rub aching muscles [Dene: 100].  **Above-ground parts:** Tea used to treat skin rash [Dene: 100]. |
| **Artemisia absinthium* L.  Asteraceae, Herb | wormwood (Eng.); armoise absinthe (Fr.); muse'odji'bĭk (Ojibwa) | Entire top of plant boiled and used as a warm compress to treat sprain of strained muscles [Ojibwa: 47]. |
| *Artemisia campestris* L.  Asteraceae, Herb | field sagewort (Eng.), armoise rouge, aurone sauvage (Fr.); denek'áze'eya(ha)naidíé (Chipewyan) | **Roots:** Chewed and juice swallowed as an emetic or to treat a sore throat [Dene: 13]. Tea drunk against constipation [Ojibwa: 19]. Chewed to cure sore throat or to induce vomiting [Chipewyan: 92]. |
| *Artemisia* *dracunculus* L.  **Syn.:** *A.dracunculoides* L.  Asteraceae, Herb | fuzzy weed, dragon wormwood (Eng.); estragon (Fr.); ba'sibûgûk', o'gima'wûck (Ojibwa) | **Leaves and flowers:** Dried, steeped in water and taken to treat heart palpitation. Chewed and used as a poultice to stop bleeding from wounds [Ojibwa: 47].  **Leaves:** Chewed to treat heart palpitation. Dried, steeped and taken to treat dysentery [Ojibwa: 47].  **Leaves and stalks:** Decoction taken in amenorrhoea [Ojibwa: 47].  **Leaves, stalks and roots:** Decoction taken to ease labor [Ojibwa: 47].  **Root:** Mixed with *Dirca palustris* in a decoction used as hair ointment. Decoction taken in amenorrhoea. Strong decoction used as a bath for strengthening children or elders [Ojibwa: 47]. |
| *Artemisia frigida* Willd.  Asteraceae, Herb | prairie sagebrush, wild sage, pasture sage, fringed sagewort (Eng.); armoise douce (Fr.); mostosowehkuskwa, moostooswehkuskwa, mōstōsowīkask (Cree); bi'jikiwĭn'gûck (Ojibwa) | **Leaves:** Rubbed on the skin as an insect repellant [Cree, Metis: 13]. Boiled leaves used as a poultice to treat skin problems, including promoting healing of burns with limited scarring [Cree: 13]. Tea drunk to relieve back pain caused by kidney trouble, to rid the body of intestinal worms, as a diuretic to treat bladder infections and other urinary disorders, to relieve the body of toxins, as a tonic to promote healing, as a gargle to treat a sore throat, as a wash for wounds and to clear blurred vision, and as a bath for sore feet, rheumatism, or arthritis [Metis: 13]. Chewed to freshen the breath [Cree: 13]. Decoction used in fever and headache [Cree: 95]. Dried, scrumbled, and placed on a hot stone, and fumes used as a disinfectant [Ojibwa: 47].  **Roots:** Mixed with *Rosa arkansana, Astragalus crassicarpus* and *Polygola senega* in a decoction used to stop bleeding from wounds, as a tonic, stimulant and antidote. Decoction used as an anti-convulsive [Ojibwa: 47]. |
| *Artemisia* *ludoviciana* Nutt. ssp. *ludoviciana*  **Syn.:** *A. gnaphalodes* Nutt.  Asteraceae, Herb | white mugwort (Eng.); armoise de Louisiane (Fr.); nokwe'jigûn (Ojibwa) | **Flowers:** Dried and placed on coals, fumes act as an antidote for "bad medicine" [Ojibwa: 47]. |
| *Artemisia norvegica* ssp. *saxatilis* (Besser) H.M. Hall & Clements **Syn:** *A.* *arctica* Less. ssp. *arctica*  Asteraceae, Herb | mountain sagewort, arctic wormwood (Eng.); armoise saxatile (Fr.) | **Roots:** Boiled and drunk to treat backpain [Dene: 98]. |
| *Artemisia* sp.  Asteraceae, Herb |  | Decoction used for stomach troubles, cold, worms, and other ailments [44]. |
| *Artemisia* *tilesii* Ledeb.  **Syn.:** *A.* *tilesii* Ledeb. ssp. *elatior* (Torr. & A. Gray) Hultén  Asteraceae, Herb | Tilesius wormwood (Eng.); armoise de Tilesius (Fr.); gyùu tsanh (Dene) | Tea used for cold and sore throat. Steam inhaled to clear nasal passages [Dene: 99]. Used to treat congested chest and to clear a stuffy head or nose [Dene: 100].  **Flowers:** Boiled and drunk to treat cough and liver ailments [Dene: 98]. |
| *Asarum canadense* L.  Aristolochiaceae, Herb | wild ginger (Eng.); asaret du Canada, gingembre sauvage (Fr.); dagmigwe-al (Malecite); agabwen, name'pîn (Ojibwa); alna'badipwa'beul^e^ (Abenaki) | **Roots:** Used as general medicine [Ojibwa: 85, 87]. Remedies for bruises and contusions [Algonquians: 63]. Tea drunk for fever and convulsions in infants [Algonquin: 69]. Steeped and given in cramps [Malecite: 65]. Used as cough medicine [Abenaki: 67]. Mixed with *Plantago major* to make a poultice used to treat inflamed skin. Dried, mashed and applied as a poultice on fractures [Ojibwa: 47].  **Rhizomes and roots:** Used as a carminative [Ojibwa: 88] or as a remedy for stomach troubles [44]. |
| *Asclepias syriaca* L.  Asclepiadaceae, Herb | milkweed (Eng.); asclépiade commune, petits cochons (Fr.); inĭ'nĭwûnj, cabo'sîkûn (Ojibwa) | **Roots:** Used as a "female remedy" [Ojibwa: 87]. Decoction used in confinement [Ojibwa: 47]. |
| *Asclepias tuberosa* L.  Asclepiadaceae, Herb | butterfly milkweed, pleurisy root (Eng.) asclépiade tubéreuse (Fr.) | **Roots:** Used as a diaphoretic and cold medicine [Penobscot: 44]. Eaten raw for pulmonary troubles, chewed and put on wounds or pulverized on wounds, and also applied as a remedy for old obstinate sores [44]. |
| *Aspidium cristatum* (L.) Sw.  Polypodiaceae, Herb | shield fern (Eng.); dryoptère à crêtes (Fr.); ana'ganûck (Ojibwa) | **Roots:** Tea taken in stomach troubles [Ojibwa: 87]. |
| *Astragalus* *americanus* (Hook.) M.E. Jones  Fabaceae, Herb | American milk-vetch, rattle-pod (Eng.); astragale d'Amérique (Fr.); kāsīsīkwānīpathisihk (Cree) | **Roots:** Chewed and juice swallowed to treat stomachache, cramps or stomach flu [Cree: 95]. |
| *Astragalus crassicarpus* Nutt.  Fabaceae, Herb | ground plum (Eng.); astragale graines-de-boeuf (Fr.); bi'jikiwi'bûgesan (Ojibwa) | **Roots:** Mixed with *Artemisia frigida, Rosa arkansana* and *Polygola senega* in a decoction applied to wounds to stop bleeding. Decoction taken for convulsions, and as a stimulant [Ojibwa: 47]. |
| *Athyrium* *filix-femina* (L.) Roth  Dryopteridaceae, Herb | common ladyfern (Eng.); athyrium fougère-femelle (Fr.); a'sawan, ana'ganûck (Ojibwa) | **Roots:** Mixed with *Urtica gracilis* in a decoction used to treat stoppage of urine [Ojibwa: 47]. Dried, made into a powder and used to heal sores. Tea used to ease milk flow in patients with caked breast [Ojibwa: 87]. |
| *Baptisia tinctoria* (L.) R. Br.  Fabaceae, Herb | black root, wild indigo (Eng.); baptisie des teinturiers (Fr.); chepatakwawutupe (Cree) | Medicinal plant [Ojibwa: 86].  **Roots:** Mixed with *Pyrola uliginosa, Galium aperine, Aralia racemosa, Streptopus amplexifolius*, *Acer pennsylvanicum*, and *Eupatorium perfoliatum* in small quantities, steeped and drunk in gonorrhea, kidney problems, and blood spitting [Algonquians: 63].  **Rhizomes:** Powder use as an emetic, cathartic, and on ulcers and syphilitic sores [Cree: 74]. |
| **Berberis vulgaris* L.  Berberidaceae, Shrub | barberry (Eng.); berbéris vulgaire, épine-vinette (Fr.) | **Roots or bark:** Mashed and applied to ulcerated gums and sore throat [Algonquians: 63]. |
| Betula alleghaniensis Britton  **Syn.:** *B. lutea* Michx. f.  Betulaceae, Tree | yellow birch (Eng.); bouleau jaune, merisier (Fr.); wilen (Innu) | **Bark:** Infusion taken for dysentery [Algonquin: 75]. Used in rheumatism, also chewed or steeped and used against diarrhoea, indigestion and stomach cramps [Mi'kmaq: 62]. Used to make plasters [Innu: 72]. |
| *Betula* *glandulosa* Michx.  Betulaceae, Shrub | glandular birch, dwarf birch (Eng.); bouleau glanduleux (Fr.); huu t'an (Dene); ínt'ánbandhaze (Chipewyan) | **Whole plant:** Tea taken to treat stomach problems [Dene: 100].  **Inner bark:** Used to treat stomach problems [Dene: 100].  **Leaves:** Used to treat insect bites [Dene: 100].  **Twigs:** Chewed and applied to deep cuts [Chipewyan: 92]. |
| *Betula lenta* L.  Betulaceae, Tree | black birch (Eng.), bouleau flexible, merisier rouge (Fr.); winsik, kade-wigwas | Medicinal plant [Algonquin: 69].  **Bark:** Mixed with beech (*Fagus grandifolia*) and red-osier dogwood (*Cornus* *stolonifera*) as a remedy for pulmonary trouble. Decoction used against pneumonia and diarrhoea [Chippewa: 85].  **Sap:** Drunk in tiredness [55]. |
| *Betula nana* L.  Betulaceae, Shrub | bog birch, dwarf birch, glandular birch (Eng.); boleau nain, bouleau de savane (Fr.) | **Twigs:** Fresh twigs chewed and put on a deepcut to stop bleeding [Dene: 13].  **Stem and leaves:** Boiled to make a weight loss tea [Dene: 13]. |
| *Betula* *nana* L. ssp. *exilis* (Sukaczev) Hultén  **Syn.:** *B. exilis* Sukaczev  Betulaceae, Shrub | dwafr birch (Eng.); bouleau grêle (Fr.); wi'umis'sik (Ojibwa) | **Inner bark:** Mixed with *Acer saccharinum* in a decoction taken as a diuretic [Ojibwa: 84]. |
| *Betula* *neoalaskana* Sarg.  Betulaceae, Shrub | Alaska paper birch, white birch (Eng.); bouleau d'Alaska (Fr.); k'i (Chipewyan) | **Leaves:** Chewed and plastered on wasp stings to extract the poison [Chipewyan: 92]. |
| *Betula* *nigra* L.  Betulaceae, Tree | black birch (Eng.); bouleau noir (Fr.) | **Bark:** Decoction taken in stomach pain [Ojibwa: 47]. |
| *Betula papyrifera* Marsh.  Betulaceae, Tree | paper birch, white birch (Eng.); bouleau à papier, bouleau blanc (Fr.); wuskwi-atik, wāskwayahtik, wasgwah, waskwaha, wāskwāh, waskway, owkimawa(h)tik (Cree); k'i (Chipewyan); maskwe'nos (Malecite); wi'gwasa'tĭg (Ojibway); uâshkuai (Montagnais); aat'oo, k' i (Dene) | Sapling used to reduce swelling caused by a bee sting [Dene: 101].  **Leaves:** Chewed and plastered on wasp stings to extract the poison [Dene: 13; Chipewyan: 92]. Used to treat insect bites [Dene: 100].  **Stem or branch:** Decoction taken in teething sickness [Cree: 95]. Dried twigs used to clean out a boil [Dene: 101]. Boiled and taken as a tea to treat stomach ailments [Dene: 99].  **Buds:** Mixed with lard and applied as an ointment [Cree: 93].  **Wood:** Dried, finely powdered rotten wood used as baby powder [Cree: 94, 95; Metis: 13]. Decoction used in back pain or to induce lactation [Cree: 95]. Boiled and water used as a topical cleanser [Cree: 93].  **Wood and inner bark:** Decoction used to treat "women's troubles" [Cree: 95].  **Inner bark:** Used to ease teething pain [Cree, Metis: 13]. Grated and eaten with balsam fir as a beneficial to diet [Montagnais: 60]. Steam from tea inhaled to treat asthma [Cree: 13], drunk for "women's troubles" [Metis: 13], or used as a gargle for tonsillitis, sore throat, and cold [Cree, Metis: 13]. Boiled and used as a poultice to treat burns and wounds [Cree, Metis: 13]. Scraping from the outside of the innermost bark layer put in water and applied to infected cuts [Malecite: 65]. Dried, ground and added to an ointment made from pitch and grease to treat persistent scabs and rashes, and boiled to make a wash to treat skin rash and other skin sores [Cree: 95]. Tea used to treat stomach problems [Dene: 100]. Boiled and tea smeared on burns, or drunk to cure menstrual cramps, cough and cold, back pain. Ashes smeared on abscesses [Dene: 101].  **Bark:** Mixed with another plant in a decoction used by women who cannot conceive a child. Powdery outer layer sprinkled on a sprained ankle before bandaging it [Cree: 13]. Used as a cast for a broken arm or leg, a sprained ankle, or swollen limbs [Cree: 13; Dene: 101]. Steamed and peeled to produce thin sheets suitable for bandages [Metis: 13; Cree: 95]. Dried and used as baby powder [Ojibway: 89]. Tea used for diarrhoea [Montagnais: 71]. White powder on the bark (lichens) used for diaper rash and other skin rashes [Algonquin: 69]. Compress used to treat abscesses, boiled and used against impetigo [Cree: 80].  **Roots:** Mixed with other plants in a decoction drunk to relieve menstrual cramps, a different mix is used as a heart medicine [Dene: 13]. Tea used to treat snow blindness [Dene: 99].  **Sap:** Used as a cough medicine, warmed and taken as a tea for general health [Dene: 17]. Tea drunk for cough and breathing problems [Dene: 101]. |
| **Betula* *pubescens* Ehrh.**Syn.:** *B. alba* L.  Betulaceae, Tree | downy birch (Eng.); bouleau pubescent (Fr.); wuskwi (Cree); wîgwas (Ojibwa) | **Wood:** White rotten wood boiled in a decoction of *Ledum latifolium*, dried, powdered and used in dermatological problems [Cree: 42].  **Buds:** Used in gonorrhoea [Cree: 74].  **Bark:** Mixed with *Tsuga canadensis* and pine bark in an infusion used in treatment of consumption and other lung troubles [Cree: 74]. Root bark and maple sugar cooked together to make a soothing syrup to alleviate stomach cramps [Ojibwa: 87]. |
| *Betula pumila* L. var. *glandulifera* Regel.  **Syn:** *B. glandulifera* (Regel) Butler  Betulaceae, Tree | low birch (Eng.); bouleau glandulifère (Fr.); bîne'mîcins (Ojibwa); ínt'ánbandhaze (Chipewyan) | **Cones:** Put on coals and used as an incense to cure catarrh. Tea drunk during menstruation and for strengthening during childbirth [Ojibwa: 87].  **Twigs:** Chewed and put on a deep cut [Chipewyan: 92]. |
| *Boschniakia* *rossica* (Cham. & Schltdl.) Fedtsch.  Orobanchaceae, Herb | northern groundcone (Eng.); boschniakie de Russie (Fr.); du'iinahshèe (Dene) | **Roots:** Boiled and drunk to cure stomach problems [Dene: 98]. White central part of roots or new plants referred to as "potatoes" boiled and eaten to increase appetite or relieve stomach ache [Dene: 99] or chewed as a general medicine. |
| *Botrychium virginianum* (L.) Sw.  Ophioglossaceae, Herb | rattlesnake fern, Virginia grape fern (Eng.); botryche de Virginie (Fr.); ozaga'tigŭm, gîckênsîne'namûkûk (Ojibwa) | Used for lung trouble and consumption [Ojibwa: 87], and chorea [Abenaki: 67].  **Roots:** Bruised and applied to cuts [Ojibwa: 44, 84]. Poultice applied to snake bite [Ojibwa: 47]. |
| *Brassica* sp.  Brassicaceae, Herb | mustard (Eng.) | Used in rheumatism [Mi'kmaq: 62]. |
| *Calla palustris* L.  Araceae, Herb | water arum (Eng.); calla des marais (Fr.); ōcicākokātask (Cree); ûshteshu (Montagnais) | Compress applied to burns [Montagnais: 71].  **Rhizomes:** Used topically to treat sore legs [Dene: 13]. Chewed to treat sore mouth, powder put on sores [Dene: 98].  **Stem:** Used to treat sore legs [Cree: 95]. |
| *Caltha palustris* L.  Ranunculaceae, Herb | marsh marigold (Eng.); populage des marais (Fr.); o'git'bûg, mi^n^gde-beguk (Ojibwa) | Mixed with sugar maple in a decoction used as a cough syrup [Ojibwa: 88].  **Roots:** Boiled and mashed to make a poultice for stubborn sores [Ojibwa: 85]. Boiled, strained, cooled and taken to treat colds [Ojibwa: 47]. Mashed and applied externally to treat scrofula. Mixed with *Sanicula canadensis* in a decoction taken in confinement [Ojibwa: 47].  **Leaves and stalks:** Mixed with leaves and stalks of *Ribes* sp. and taken to treat stoppage of urine [Ojibwa: 47]. |
| *Calvatia craniiformis* Schw.  Lycoperdaceae, Fungi | giant puffball (Eng.); vesse-de-loup crâniforme (Fr.); oskwe'tûk (Ojibwa) | Spores inhaled to stop nose bleeding [Ojibwa: 47, 87]. |
| *Campanula rotundifolia* L.  Campanulaceae, Herb | harebell, bluebell (Eng.); campanule à feuilles rondes (Fr.); kuskwasonapiskos, sewayonakunis, mitīhīmaskīhkīh (Cree); degaimaribet'á^n^chayé (Chipewyan); mekmi^n^swa^n^, zi'gĭnĭ'ce, adota'gons (Ojibwa) | **Roots:** Tea used to treat influenza, fever, lung trouble, or heart trouble [Dene, Metis: 13; ; Chipewyan: 92]. Infusion used as ear drops to treat ear soreness [Ojibwa: 47], and in a compound medicine for lung troubles [Ojibwa: 87]. Chewed for heart ailments [Cree: 95]. |
| **Capsella* *bursa-pastoris* (L.) Medik.  Brassicaceae, Herb | shepherd's purse (Eng.); capselle bourse-à-pasteur (Fr.); ĭ'ckode'wadji'bĭk (Ojibwa) | **Whole plant:** Decoction used in dysentery [Ojibwa: 47]. |
| Cardamine diphylla (Michx.) Alph. Wood **Syn.:** *Dentaria diphylla* Michx.  Brassicaceae, Herb | toothwort, pepper-root (Eng.); dentaire à deux feuilles, snicroûte, carcajou (Fr.); ka'djiwuk (Malecite) | **Roots:** Tea given for fever in children. Mixed with sweetflag in a tea used for heart disease [Algonquin: 69]. Chewed (green or dried) for hoarseness or to clear throat. Steeped and given as a tonic [Malecite: 65]. |
| *Carex* *aquatilis* Wahlenb.  Cyperaceae, Herb | sedge (Eng.); carex aquatique (Fr.); tlh'oghtsëné (Chipewyan) | **Roots:** Used to induce menstruation [Dene: 13; Chipewyan: 92] or as part of a compound tea for intestinal problems [Dene: 13]. |
| **Carum carvi* L.  Apiaceae, Herb | caraway (Eng.); carvi commun, anis, (Fr.); sīcisis, iskotawutupe (Cree) | Analgesic [Abenaki: 67]. Boiled with '*Petasites sagittatus* to treat chickenpox [Dene: 17].  **Rhizomes and seeds:** Used as a corrective and adjuvant, and to relieve colic [Cree: 74].  **Seeds:** Tea given to children to treat cough [Cree: 13]. |
| *Carya* *cordiformis* (Wangenh.) K. Koch  Juglandaceae, Tree | bitternut hickory (Eng.); caryer cordiforme, noyer amer (Fr.) | Oil used in rheumatism [91]. |
| *Carya* *laciniosa* (Michx. f.) G. Don  Juglandaceae, Tree | shellbark hickory (Eng.); caryer lacinié (Fr.) | Used for arthritis [91]. |
| *Carya* *ovata* (Mill.) K. Koch  **Syn:** *Hicoria* *alba* Britton  Juglandaceae, Tree | shagbark hickory (Eng.); caryer ovale, noyer tendre (Fr.) | Used for arthritis [91].  **Shoots:** Fresh shoots placed on hot stones and fumes inhaled in convulsions [Ojibwa: 47]. |
| *Castilleja* *coccinea* (L.) Spreng.  Scrophulariaceae, Herb | scarlet paintbrush, Indian paintbrush (Eng.); castilléjie écarlate (Fr.); wĭnabojo'noko'mĭs wi'nĭzĭsûn' (Ojibwa) | Used in "women diseases" [Ojibwa: 47].  **Flowers:** Decoction used in rheumatism [Ojibwa: 47]. |
| *Castilleja miniata* Dougl. Ex Hook.  Scrophulariaceae, Herb | great red paintbrush, Indian paintbrush, painted-cup (Eng.); castilléjie rougeâtre (Fr.) | **Flowers:** Dried heads added to wild chamomile flowers to make a tea to cure headaches and relax nerves [Metis: 13]. |
| *Caulophyllum* *thalictroides* (L.) Michx.  Berberidaceae, Herb | blue cohosh (Eng.); caulophylle faux-pigamon (Fr.); iskwawutupe (Cree); be'cigodji'bigûk, oci'gîmîc (Ojibwa) | Used in cramps [Ojibwa: 47].  **Rhizomes:** Powdered and used in uterine troubles, especially dysmenorrhea, metrorrhagia and post partum hemorrhage [Cree: 74]. Infusion given to render delivery rapid and painless. Used for rheumatism, dropsy, uterine inflammation, and colic [44].  **Roots:** Infusion used in amenorrhea [Cree: 74] or for painful menstruation [Ojibwa: 87]. Tea used as an emetic [Ojibwa: 87]. Decoction used in lung troubles. Mixed with *Rudbeckia laciniata* in a decoction taken in indigestion. Mixed with *Sanguinaria canadensis* in a decoction taken to treat stomach cramps. Scraped finely, tied in a cloth, squeezed in warm water taken as an emetic [Ojibwa: 47].  **Flowers:** Infusion used for rheumatism and sciatica [Cree: 74]. |
| *Ceanothus americanus* L.  Rhamnaceae, Shrub | New Jersey tea (Eng.); céanothus d'Amérique (Fr.); kadegimnedu, konjibik (Ojibwa) | **Roots:** Steeped and infusion drunk to cure constipation, bloating and shortness of breath. Mixed with roots or branches of wild grape, roots of *Hepatica*, bark of beech, and inner bark of sugar maple, black birch and red-osier dogwood, all steeped together to make a remedy for pulmonary troubles [Ojibwa: 85]. |
| *Ceanothus* *herbaceus* Raf.  **Syn.:** *C. ovatus* auct. non Desf.  Rhamnaceae, Shrub | New Jersey tea (Eng.); céanothus à feuilles ovées (Fr.); odiga'dimanido' (Ojibwa) | Used in lung troubles and as an emetic [Ojibwa: 47].  **Roots:** Decoction taken in cough [Ojibwa: 47]. |
| *Celastrus scandens* L.  Celastraceae, Vine | bitter-sweet (Eng.); célastre grimpant, bourreau des arbres (Fr.); bima'kwûd, manîdobima'kwit (Ojibwa) | Medicinal plant [Ojibwa: 86].  **Roots:** Cooked in animal fat, strained, and used as an ointment for cancer or obstinate sore [Chippewa: 85; Ojibwa: 87]. Decoction used to treat stoppage of urine [Chippewa: 47], or as a physic or diuretic.  **Fruits:** Used for sotmach troubles [Ojibwa: 87].  **Stalk:** Decoction used in skin eruptions [Ojibwa: 87]. |
| *Cetraria* *islandica* (L.) Ach.  Parmeliaceae, Lichens | lichen (Eng.); lichen d'Islande (Fr.) | Boiled and taken to treat tuberculosis [Dene: 98]. |
| *Chamaecyparis* *thyoides* (L.) BSP  **Syn.:** *Cupressus thyoides* L.  Cupressaceae, Tree | white cedar (Eng.); cèdre blanc de l'Atlantique (Fr.); gī'zhik (Ojibwa) | **Leaves:** Cushed and applied to relieve headache, boiled and inhaled to cure backache [Ojibwa: 84]. |
| *Chamaedaphne calyculata* (L.) Moench  **Syn.:** *Cassandra calyculata* (L.) D. Don.  Ericaceae, Shrub | leatherleaf (Eng.); faux bleuet, cassandre caliculé (Fr.); matshikîsha (Montagnais) | **Bark or twigs:** Steeped into water and used as a compress or tea for headache [Montagnais: 71]. |
| Chamerion angustifolium (L.) Holub ssp. angustifolium  **Syn.:** *Epilobium angustifolium* L.  Onagraceae, Herb | fireweed, willow herb (Eng.); épilobe à feuilles étroites, bouquets rouges (Fr.); hapaskwa, askapask, athkāpask, ākāpuskwah, liy(h)kāpusk (Cree); oja'cidji'bĭk (Ojibwa); kapamastak (Atikamekw); tlitl'echi (Dene); gó^n^dhi'elé (Chipewyan) | Applied to treat cuts [Algonquin: 75] or used in cough [Abenaki: 67].Young tops eaten to "strengthen the blood" [Metis: 13]. Tea used to relieve a sore stomach [Dene: 101].  **Whole plant:** Tea taken to treat intestinal worms [Dene: 13; Chipewyan: 92]. Boiled and the liquid rubbed on the skin to ease rashes [Dene: 99].  **Leaves:** Chewed and applied as a plaster on bruises [Metis: 13; Cree: 95]. Poultice from fresh or dried leaves applied to bruises [Chippewa: 47], burns, bee stings, aches and swelling caused by arthritis [Dene: 99]. Chewed and applied to bee stings and bites [Dene: 100].  **Roots:** Roasted in ashes, mashed, and applied to boils [Algonquin: 68]. Peeled, chewed or pounded, and applied as a poultice on boils, carbuncle, abscesses or open wounds to prevent infection [Metis: 13; Cree: 95; Ojibwa: 87]. Boiled and used for skin problems [Atikamekw: 73]. Herbal water taken to induce menses [Cree: 93]. |
| *Chamerion* *latifolium* (L.) Holub **Syn.:** *Epilobium* *latifolium* L.  Onagraceae, Herb | river beauty, dwarf fireweed (Eng.); épilobe à feuilles larges (Fr.); tlitl'echi (Dene) | Tea used to relieve a sore stomach [Dene: 101].  **Leaves:** Chewed and applied to bee stings and bites [Dene: 100]. |
| *Chelone glabra* L.  Scrophulariaceae, Herb | white turtle head (Eng.); tête de tortue, galane glâbre (Fr.); athozis wiwsnia'ginol (Malecite) | Steeped and given to prevent pregnancy [Malecite: 65].  **Roots:** Used with cedar bark to prepare a medicinal tea [Algonquin: 69]. |
| **Chenopodium album* L.  Chenopodiaceae, Herb | lamb's quarters (Eng.); chénopode blanc, chou gras (Fr.); wīthiniwpakwātik (Cree) | **Leaves:** Dipped in maple or birch sap and used to treat cold [55]. Decoction taken or applied to treat painful limbs [Cree: 95]. |
| **Chenopodium ambrosioides* L.  Chenopodiaceae, Herb | American wormseed (Eng.); chénopode fausse-ambroisie (Fr.) | **Whole plant:** Infusion taken to relieve painful menstruation [44]. |
| *Chimaphila* *umbellata* (L.) W. Bartram  Pyrolaceae, Herb | prince's pine, pipsissewa (Eng.); chimaphile à ombelles, herbe à peigne (Fr.); amiskwāthōwipak (Cree); k'agegiga'kil (Malecite); yaskopteg, ga'gîge'bûg (Ojibwa); jabak (Abenaki) | Mixed with other species to make a remedy for gonorrhoea and ulcers. Steeped and applied to blisters [Ojibwa: 85]. Applied to open sores and ingested as a tonic and diuretic [44]. Used as an ingredient in a decoction to treat backache or stabbing pain in the chest. Tea or decoction used in blood coughing [Cree: 95]. Steeped with common Juniper and taken in consumption. Steeped and taken to purify blood [Malecite: 65]. Used as a medicine for head colds [Abenaki: 67]. Used in tuberculosis and as a general stomach medicine [Mi'kmaq: 62; Ojibwa: 87]. Boiled and drunk to induce sweating [Algonquians: 63].  **Roots:** Decoction used to treat sore eyes [Ojibwa: 47]. Steeped with *Conioselium chinense, Tsuga canadensis,* and *Rumex crispus* and given in bladder problems [Mi'kmaq: 62]. |
| *Cicuta* *douglasii* (DC.) J.M. Coult. & Rose  Apiaceae, Herb | Douglas' water-hemlock, western water-hemlock (Eng.); cicutaire pourpre (Fr.) | **Roots:** Smoked to cure headache [Dene: 98]. |
| *Cicuta maculata* L.  Apiaceae, Herb | waterhemlock (Eng.); cicutaire maculée, carotte à Moreau (Fr.); maciskatask (Cree); apagwasî'gons (Ojibwa) | **Roots:** Medicinal [Ojibwa: 87]. Dried and powdered to make a liniment applied externally [Cree: 95]. |
| **Cirsium arvense* (L.) Scop.  Asteraceae, Herb | Canada thistle (Eng.); chardon des champs (Fr.); kaminakuse, kaweminukuse (Cree); masa'nûck (Ojibwa); kon'hizak (Abenaki) | Boiled for consumption [Algonquians: 63]. Tonic, diuretic and astringent [Chippewa: 47]. Used as a bowel tonic [Ojibwa: 87] or as a vermifuge for children [Abenaki: 67]. Used to reduce itching, particularly that caused by *Rhus radicans* (poison ivy) [Algonquin: 68].  **Roots:** Fleshy white root dried, powdered, and mixed with warm animal grease as a poultice to be applied to aching joints [Metis: 13]. |
| *Cirsium* *discolor* (Muhl. ex Willd.) Spreng.  Asteraceae, Herb | common thistle (Eng.); chardon discolore (Fr.); kee-poo-cus-i-cun (Cree) | Dried, powdered and mixed with water to make a paste applied to wounds [Cree: 76]. |
| *Cirsium* sp.  Asteraceae, Herb | thistle (Eng.); chardon (Fr.); ma'zana'tĭg (Ojibwa) | **Roots:** Mixed with *Populus balsamifera* in a decoction taken internally to treat "female weakness" and back pain. Steeped with *Taraxacum officinale* and taken in confinement [Ojibwa: 47]. |
| **Cirsium* *vulgare* (Savi) Ten.  **Syn.:** *C. lanceolatum* (L.) Hill  Asteraceae, Herb | common thistle (Eng.); chardon vulgaire, piqueux (Fr.); ji'masa'nûck (Ojibwa) | **Roots:** Used for alleviating stomach cramps [Ojibwa: 87]. |
| *Cladina* *stellaris* (Opiz) Brodo  **Syn.:** *C. alpestris* (L.) Rabenh.; Cladonia stellaris (Opiz) Pouzar & Vezda  Cladoniaceae, Lichen | reindeer lichen (Eng.); lichen à caribou, mousse à caribou (Fr.); wāpiskastaskamihk, atikōmīciwin (Cree) | Powdered and mixed with water to expel intestinal worms [Cree: 95]. |
| *Cladina* *rangiferina* (L.) Nyl.  Cladoniaceae, Lichen | greygreen reindeer lichen (Eng.); lichen à caribou, mousse à caribou (Fr.); whapskumuk, epshatuk (Cree) | Used to treat diabetes [Cree: 82]. |
| Clintonia borealis (Aiton) Raf.  Liliaceae, Herb | corn-lily, bluebead lily (Eng.); clintonie boréale (Fr.); gînose'wîbûg (Ojibwa); sestedomanuk, skaskataminask (Atikamekw) tshîtshue atâpukuat (Montagnais) | Pounded and applied to face and hands as a mosquito repellent [Atikamekw: 73]. Used to activate labour for childbirth [Ojibway: 73].  **Leaves:** Poultice used in infections and open wounds [Algonquin: 69; Atikamekw: 73]. Boiled before being applied to swelling and hemorroids [Montagnais: 71]. Applied to burns. Decoction applied to treat scrofula [Ojibwa: 47].  **Roots:** Tea used to help parturition. Put on a dog bite to prevent infection [Ojibwa: 87]. |
| *Clintonia* *uniflora* (Menzies ex Schult. & Schult. f.) Kunth  **Syn.:** *Smilacina* *borealis* (Aiton) Raf.  Liliaceae, Herb | single-flowered clintonia, Queens cup, bride's bonnet (Eng.); clintonie uniflore (Fr.) | **Roots:** Juice drunk in gravel [Algonquians: 63]. |
| *Comarum* *palustre* L.  **Syn.:** *Potentilla* *palustris* (L.) Scop.  Rosaceae, Herb | marsh five-finger (Eng.); potentille palustre (Fr.); beba'akwûndek, bĭne'bûg (Ojibwa) | Plant used to cure stomach cramps [Ojibwa: 87].  **Roots:** Decoction used to treat dysentery [Ojibwa: 47]. |
| Comptonia peregrina (L.) J.M. Coult.  **Syn.:** *Myrica asplenifolia* L.  Myricaceae, Shrub | sweet fern, ant wood (Eng.); comptonie voyageuse (Fr.); kba'agne-mins (Chippewa ); eninigsomo'zil (Malecite); kba'agne-minš, gibaime'nûna'gwûs (Ojibwa) | Used for catarrh. Steeped with yarrow and applied to sprain swelling [Malecite: 65]. Used to treat eczema, sores, cancer and poison ivy rash [Mi'kmaq: 61].  **Leaves:** Steeped and rubbed on the skin to cure poison ivy rash [Mi'kmaq: 60; Algonquians: 63]. Steeped with leaves of catnip (*Nepeta calaria*) to make an infusion to be used as a febrifuge [Chippewa: 85]. Tea used to make to cure the flux and stomach cramps [Ojibwa: 87]. Steeped and tea taken as a general tonic. Poultice used for rheumatism and external sores [Mi'kmaq: 62]. Crushed and the perfume inhaled or drunk as a tea for headache [Algonquin: 69]. |
| *Conioselinum* *chinense* (L.) Britton, Sterns & Poggenb.  Apiaceae, Herb | Chinese hemlockparsley (Eng.); coniosélinum de Genesee (Fr.) | **Roots:** Steeped with *Tsuga canadensis*, *Chimaphila umbellata* and *Rumex crispus* and given in bladder problems [Mi'kmaq: 62]. |
| *Coptis* *trifolia* (L.) Salisb.  **Syn.:** *C. groenlandica* (Oeder) Fernald  Ranunculaceae, Herb | golden thread (Eng.); coptide du Groenland, savoyane (Fr.); wizauke'skil (Malecite); wi'ca'uckomuk, uîshakâshkamuk^u^ (Montagnais/Innu); wijô'wapap'kôs (Abenaki); osawskamikwapi (Atikamekw); wesa wa'nikwe'ak , oza'widji'bĭg (Ojibwa) | Steeped, used to saturate a rag to be placed in sore mouth [Mi'kmaq: 61; Malecite: 65]. Used to treat frostbite or to stimulate appetite [Algonquin: 68].  **Stem:** Tea used as a wash for sore eyes, lips and interior of mouth [Montaganais: 70]. Chewed to allay canker or sores on the gums or in the mouth. Used to ease mouths irritated by excessive tobacco smoking [Algonquians: 63].  **Roots:** Used for sore eyes or steeped to make a stomach medicine [Mi'kmaq: 62]. Tea used for heart disease, toothache, diarrhoea and as an eyewash [Algonquin: 69; Atikamekw: 73]. Used for cough [Abenaki: 67; Atikamekw: 73]. Decoction used to soothe teething pain and heal gums, and as a mouth wash to treat sore mouth [Ojibwa: 87]. Decoction used for throat pain, cough and mouth infection [Montagnais: 71]. |
| *Cornus alternifolia* L. f.  Cornaceae, Tree | dogwood (Eng.); cornouiller à feuilles alternes (Fr.); mu^n^s-mins, moso'mîc, muj'omĭj' (Ojibwa) | **Bark:** Used as an eye bath [Chippewa: 85]. Decoction used as an emetic, in cough and fever [44].  **Inner bark:** Used as an emetic [Ojibwa: 87].  **Roots:** Alone or mixed with *Cornus sericea* and *Alnus incana* in a decoction used as a wash or compress to treat sore eyes [Ojibwa: 47]. |
| *Cornus canadensis* L.  Cornaceae, Shrub | bunch berry, dogwood, dwarf cornel, pigeonberry (Eng.); cornouiller du Canada, quatre-temps, rougets (Fr.); ode'imînîdji'bîk (Ojibwa); patkéna'houk (Abenaki); saguminan (Atikamekw); shâshâkuminânakashî (Montagnais); nekutsahemen (Innu) | Medicinal plant [Innu: 72].Used to heal sores and cancers [Mi'kmaq: 61]. Steeped and used for paralysis [Algonquians: 63]. Boiled with *Gaultheria procumbens* and used for cold or mixed with *Taxus canadensis* twigs and used against menstrual disorders [Atikamekw: 73]. Decoction used for "stich in the side" [Abenaki: 67]. Steeped and the liquid drunk in kidney problems [Mi'kmaq: 62].  **Whole plant:** Boiled and liquid drunk for rheumatism and other types of pain [Montagnais: 71].  **Leaves:** Used to prepare cathartic tea [Algonquin: 69].  **Roots, leaves and fruits:** Steeped and given against fits [Malecite: 65].  **Roots:** Tea used to cure colics [Ojibwa: 87]. |
| *Cornus florida* L.  Cornaceae, Tree | flowering dogwood (Eng.); cornouiller fleuri (Fr.); nemwatik (Chippewa) | Used for fever and cold [44].  **Inner bark:** Mixed with other plants (*Sanguinaria, Asarum* and *Ostrya*) to make a cough remedy [Chippewa: 85]. |
| *Cornus* *foemina* Mill.  **Syn.:** *C.* *stricta* Lam.  Cornaceae, Shrub | stiff dogwood (Eng.) | **Bark:** Dried and mixed with tobacco for smoking [Algonquians: 63]. |
| *Cornus* *racemosa* Lam.  **Syn.:** *C.* *paniculata* L'Hér.  Cornaceae, Shrub | panicled dogwood (Eng.); cornouiller à grappes (Fr.); meskwabi'mîc (Ojibwa) | **Bark:** Tea used for flux. Inserted into the anus as a treatment for piles [Ojibwa: 87]. |
| *Cornus sericea* L.  **Syn.:** *C.* *stolonifera* Michx  Cornaceae, Shrub | red-osier dogwood, red willow (Eng.); cornouiller stolonifère, hart rouge (Fr.); mehkwa pemakwa, mikwapamuk, mīhkwa pēmakwa, mīkōbīmāka, mikwanbimaka, mikwapimakwah, mithkwāpīmak, miskwāpīmak, mi(h)kwapiymak, mikwa piskaw, nipsiy wasaskwetow, pīmīhkwāhtik, mīhkwanīpisīya, mikobimuk (Cree); k'áik'ozé (Chipewyan); meskwabi-mins, mĭs'kwabi'mĭc (Ojibwa); nespipam'k' (Malecite); mamkawa'kousek (Abenaki); mîkuâpemuk^u^ (Montagnais) | Medicinal plant [Chippewa: 86]. Chewed and placed on fresh cuts to stop bleeding [Mi'kmaq: 43]. Steeped and used for diarrhoea or to make a wash to cure eruptions caused by poison ivy or by other poisons [Chippewa: 85]. Chewed and applied to sore eyes. Steeped and used as a gargle in sore throat. Used in catarrh [Malecite: 65].  **Bark:** Decoction from young bark used as an emetic in cold, cough and fever [Cree: 42]. Peeled, mixed with another plant, boiled, and a cloth soaked in the solution is applied to sore eyes [Cree: 13]. Applied to wounds to stop bleeding or drunk as a tea for cold [Algonquin: 69]. Smoked [Ojibwa: 86]. Infusion used to treat eye problems [Abenaki: 67]. Boiled and used in a mixture with barks of poplar, birch and spruce to treat cough and cold [Dene: 17]. Herbal water taken for diarrhoea and to induce vomiting [Cree: 93].  **Stems:** Tea taken for chest trouble and to cure stoppage of urine [Dene: 13; Chipewyan: 92].  **Roots:** Tea drunk to treat dizziness [Dene: 13; Chipewyan: 92] or stomach problems [Cree: 96]. Mixed with other herbs in a decoction given to children to stop diarrhoea [Cree: 13]. Mixed with *Cornus alternifolia* and *Alnus incana* in a decoction used as a wash or compress to treat sore eyes [Ojibwa: 47]. Scraped and mixed with *Aralia racemosa* and smoked to treat headache [Malecite: 65]. Used to treat aching muscles and soreness [Dene: 17].  **Fruits:** Boiled and taken to treat tuberculosis [Dene: 98].  **Fruits or pith:** Used to make a wash to treat snow blindness [Cree: 95].  **Pith:** Used in cataracts [Cree: 95].  **Bark and twigs:** Decoction used for skin or eye problems [Montagnais: 71].  **Leaves:** Crushed and used to treat burns, bee stings and insect bites [Dene: 100]. |
| *Cornus* sp.  Cornaceae, Shrub | willow, dogwood (Eng.); cornouiller (Fr.) | **Bark:** Minced, boiled and applied as a poultice in sprains [Algonquin: 75]. |
| *Corydalis aurea* Willd. Fumariaceae, Herb | golden corydalis (Eng.); corydale dorée (Fr.); tîpotîe'kwason (Ojibwa) | **Roots:** Placed on coals and smoke inhaled to clear the head and as a tonic [Ojibwa: 87]. |
| *Corylus americana* Walt.  Betulaceae, Shrub | American hazelnut (Eng.); noisetier d'Amérique (Fr.); mûkwobaga'nak (Ojibwa) | **Bark:** Boiled and used as a poultice to heal cuts [Ojibwa: 87].  **Stalk:** Burned and charcoal combined with bear gall pricked into the temples with needles to treat convulsions [Ojibwa: 47]. |
| *Corylus cornuta* Marsh.  **Syn :** *C. rostrata* Ait.  Betulaceae, Tree | beaked hazelnut (Eng.); noisetier à long bec (Fr.); bagan', baga'nak (Ojibwa); pakanatuk (Atikamekw) | Hairs of the husk used to expel worms [Ojibwa: 87].  **Branches and leaves:** Tea used in heart trouble and intestinal disorders [Atikamekw: 73; Algonquin: 69]. |
| *Crataegus* sp.  Rosaceae, Tree | hawthorn (Eng.); aubépine (Fr.); minesga^n^-wins, mînesaga'wûnj (Chippewa) | **Bark:** Medicinal [Chippewa: 85, 87].  **Roots:** Medicinal [Chippewa: 85, 87]. Mixed with other plants in a decoction given to treat "female weakness" and back pain [Ojibwa: 47]. |
| **Cucurbita* *maxima* Duchesne  Cucurbitaceae, Vine | squash (Eng.); courge (Fr.); ogwîssi'maun o'wasokwûne'k (Ojibwa) | **Seeds:** Tea drunk as a diuretic [Ojibwa: 87]. |
| *Cynoglossum* *virginianum* L. var. *boreale* (Fernald) Cooperr.  **Syn.:** *C. boreale* Fernald  Boraginaceae, Herb | hound's tongue (Eng.); cynoglosse boréal (Fr.); masa'n (Ojibwa) | Smoked to cure headache [Ojibwa: 87]. |
| *Cypripedium acaule* Aiton  Orchidaceae, Herb | lady'slipper, moccasin flower, nerve root (Eng.); cypripède acaule, sabot de la vierge (Fr.); kamidjetotapi (Atikamekw); ago'biso'wĭn (Ojibwa) | **Roots:** Steeped and used for nervousness [Algonquians: 63; Mi'kmaq: 60]. Used in venereal disease and menstrual disorders [Algonquin: 69]. Boiled and used against stomachache, urinary tract disorders and kidney problems for children [Atikamekw: 73]. Powdered, moistened and used to treat toothache. Steeped and taken in indigestion. Poultice applied to skin inflammation [Chippewa: 47].  **Flowers:** Powdered, moistened and used to treat toothache [Chippewa: 88]. |
| *Cypripedium* *parviflorum* Salisb.  Orchidaceae, Herb | ladyslipper (Eng.); cypripède jaune (Fr.); ma'kasîn (Ojibwa) | **Rhizomes:** Used as a sedative, antispasmodic or depressant [Cree: 74].  **Roots:** Used for "female troubles" [Ojibwa: 87]. |
| *Cypripedium* *reginae* Walter  Orchidaceae, Herb | showy lady's slipper (Eng.); cypripède royal (Fr.) | **Roots:** Used as a sedative in nervous disorders [Penosbcot: 44]. |
| *Dactylorhiza* *viridis* (L.) R.M. Bateman, A.M. Pridgeon & M.W. Chase  **Syn.:** *Habenaria* *bracteata* (Muhl. ex Willd.) R. Br.  Orchidaceae, Herb | rein orchis (Eng.); habénaire à longues bractées (Fr.); goko'cgûnda mînêskwe'mîn (Ojibwa) | Aphrodisiac [Ojibwa: 87]. |
| *Dalea purpurea* Vent.  **Syn.:** Petalostemon purpureus (Vent.) Rydb.  Fabaceae, Herb | purple prairie-clover (Eng.); dalée violette (Fr.); ba'sibûgûk' (Ojibwa) | **Leaves and flowers:** Decoction taken for heart trouble [Ojibwa: 47]. |
| *Dasiphora* *fruticosa* (L.) Rydb. ssp. *floribunda* (Pursh) Kartesz **Syn.:** *Potentilla* *fruticosa* auct. non L.  Rosaceae, Shrub | shrubby cinquefoil (Eng.); potentille frutescente (Fr.) | **Whole plant:** Decoction taken as a tea to treat fever [Dene: 98]. |
| *Delphinium* *glaucum* S. Watson  Ranunculaceae, Herb | tall larkspur (Eng.); pied-d'alouette glauque (Fr.) | **Whole plant:** Tea used to wash hair and kill lice [Dene: 100]. |
| *Diervilla lonicera* Mill.  Caprifoliaceae, Shrub | bush honeysuckle (Eng.); dièreville chèvrefeuille, herbe bleue (Fr.); wežauškwagmik, osawa'skanet (Ojibwa); maskōcīpihk (Cree); asasunatuk (Atikamekw) | **Bark:** Infusion used as an eye wash and against constipation [Ojibwa: 85].  **Roots or stem:** Infusion or decoction cooled and applied to sore eyes [Cree: 95].  **Roots:** Tea used to induce lactation at the time of childbirth [Cree: 95]. Mixed with other plants and used for urinary problems [Ojibwa: 87].  **Leaves:** Diuretic when used with *Cornus canadensis* [Atikamekw: 73]. Decoction taken in stomach pain [Ojibwa: 47]. |
| *Dirca palustris* L.  Thymelaceae, Shrub | moosewood, leatherwood, wicopy, rope-bark (Eng.); dirca des marais, bois de plomb (Fr.); djibe'gûb (Ojibwa) | **Roots:** Steeped to make a drink for pulmonary troubles [Ojibwa: 85]. Mixed with *Artemisia dracunculus* in a decoction used as a hair ointment [Ojibwa: 47].  **Bark:** Tea used as a diuretic [Ojibwa: 87].  **Inner bark:** Tea used as a laxative [Algonquin: 69].  **Stalk:** Dreid, pulverized and steeped in water and taken as a physic; green stalk chewed as a physic [Ojibwa: 47]. |
| *Dryopteris carthusiana* (Vill.) H. P. Fuchs  Dryopteridaceae, Herb | spinulose shield fern (Eng.); dryoptère spinuleuse (Fr.); ku(h)kuguwpuk (Cree) | **Frond:** Mixed with other plants in a decoction drunk for kidney pain. A different mix is used as a wash for skin diseases, and yet another mix is smoked to treat insanity [Dene: 13]. |
| *Dryopteris* *expansa* (C. Presl) Fraser-Jenkins & Jermy  Dryopteridaceae, Herb | spreading wood fern (Eng.); dryoptère dressée (Fr.) | **Young shoots:** Boiled and eaten to stimulate appetite [Cree: 93].  **Roots:** Cancer medicine [Cree: 93]. |
| *Dryopteris* *marginalis* (L.) A. Gray  Dryopteridaceae, Herb | marginal shield fern (Eng.); dryoptère à sores marginaux, dryoptéride marginale (Fr.) | **Rhizomes:** Infusion drunk as a vermifugal [Cree: 74]. |
| *Dryopteris* sp.  Dryopteridaceae, Herb | fern (Eng.); dryoptère, dryoptéride (Fr.); uîshakâtshâkuat (Montagnais) | **Leaves:** Dried, steeped into hot water and applied to swells and hemorroids [Montagnais: 71]. |
| *Echinocystis* *lobata* (Michx.) Torr. & A. Gray  Cucurbitaceae, Vine | wild balsam-apple (Eng.); échinocystis lobé, concombre sauvage (Fr.); nîgîtîni'gûnûk (Ojibwa) | **Roots:** Tea used as a tonic and as a bitter medicine for stomach troubles [Ojibwa: 87]. |
| *Echinodontium tinctorium* (Ellis & Ev.) Ellis & Ev.  [Echinodontiaceae](http://en.wikipedia.org/wiki/Echinodontiaceae), Fungi | Indian paint fungus (Eng.); meah-kis-igun (Cree) | Powdered and mixed with water to make a paste applied to wounds or given internally as an emetic [Cree: 76]. |
| *Empetrum nigrum* L.  Empetraceae, Shrub | crowberry, curlewberry (Eng.); camarine noire, graines noires (Fr.); askīmināsiht, ebshjimend (Cree); dineech'ùh (Dene) | **Whole plant:** Tea drunk for diarrhoea [Dene: 100].  **Berries:** Used to treat diabetes [Cree: 82].  **Roots:** Mixed with other plants in a decoction drunk to treat cough [Dene: 13; Chipewyan: 92].  **Twigs:** Used as a diuretic [Cree: 95]. Boiled with spruce cones and tea applied to sores, or drunk to cure mouth infections and sore throats [Dene: 101].  **Roots, berries and stems:** Tea drunk for stomach ache and cold [Dene: 99].  **Branches and roots:** Boiled and tea drunk to cure mouth infections [Dene: 101].  **Branches and berries:** Boiled and tea drunk to ease menstrual pain, pain related to child birth, and to cure mouth infections. Also applied to rashes [Dene: 101]. |
| *Epigaea repens* L.  Ericaceae, Shrub | trailing arbutus, mayflower (Eng.); épigée rampante, fleur de mai (Fr.) | **Leaves:** Tea drunk for kidney disorders [Algonquin: 69]. |
| *Equisetum arvense* L.  Equisetaceae, Herb | horsetail (Eng.), prêle des champs, queue de renard (Fr.); mistatimosoy, okotāwask, enskowusk, kiychiwiykusk (Cree); jasibonskok, gîji'bînûsk (Ojibwa); kheh dyè' (Dene) | Tea used to treat kidney problems, bladder infections or urinary track problems [Dene: 100].  **Whole plant:** Tea used to cure dropsy [Ojibwa: 87].  **Above-ground fertile shoots:** Decoction used as a diuretic or in kidney troubles [Cree: 95].  **Stem and leaves:** Burned and the ashes applied to running sores [Metis: 13]. Steamed for nasal congestion, cold and stomach ailments [Dene: 99].  **Stem:** Decoction drunk as a remedy for dysuria [Ojibwa: 85]. Herbal water used to treat kidney problems [Cree: 93].  **Roots:** Heated and applied to aching teeth [Metis: 13]. |
| *Equisetum hyemale* L.  Equisetaceae, Herb | scouring rush, horsetail (Eng.); prêle d'hiver (Fr.); gijib'inûskon' (Ojibwa) | **Leaves:** Burned and used as a disinfectant [Ojibwa: 47].  **Above-ground parts:** Tea taken in kidney problems [Cree: 96]. |
| *Equisetum* *palustre* L.  Equisetaceae, Herb | marsh horsetail (Eng.); prêle des marais (Fr.) | Mixed with *Monarda punctata* in a tea used for constipation or stomach trouble [Ojibwa: 19]. |
| *Equisetum pratense* Ehrh  Equisetaceae, Herb | common horsetail (Eng.); prêle des prés (Fr.); wishgobidjibik (Ojibwa) | Mixed with horse-mint in a tea used in stomach trouble [Ojibwa: 86].  **Roots:** Decoction taken as a diuretic [Dene: 98]. Boiled with the roots of Rubus idaeus ssp. strigosus and taken in stomach problems [Dene: 98]. |
| *Equisetum sylvaticum* L.  Equisetaceae, Herb | horsetail (Eng.); prêle des bois (Fr.); mistatimosoy, okotāwask, enskowusk, kiychiwiykusk (Cree); siba'mûckûn (Ojibwa) | **Rhizomes:** Mixed with other plants and boiled to make a wash for skin diseases [Dene: 13].  **Whole plant:** Tea used to cure kidney trouble and dropsy [Ojibwa: 87]. |
| *Erigeron canadensis* L.  Asteraceae, Herb | Canadian horseweed (Eng.); érigéron du Canada (Fr.); gababi'kwûna'tĭg (Ojibwa) | Used in diarrhoea [Cree: 42].  **Whole plant:** Steeped to treat "female weaknesses" [Ojibwa: 47].  **Leaves and roots:** Decoction taken in stomach pain [Ojibwa: 47]. |
| *Erigeron* *philadelphicus* L.  Asteraceae, Herb | Philadelphia fleabane (Eng.); érigéron de Philadelphie (Fr.); mîcaogacan (Ojibwa) | **Flowers:** Dried, smoked and inhaled to cure cold, or tea drunk to break fever [Ojibwa: 87]. |
| *Erigeron* sp.  Asteraceae, Herb | Canada fleabane (Eng.) | Decoction used for diarrhoea [44]. |
| *Erigeron* *strigosus* Muhl. ex Willd.  **Syn.:** *E. ramosus* (Walt.) BSP  Asteraceae, Herb | daisy fleahane (Eng.); érigéron hispide (Fr.); nokwe'sîgûn (Ojibwa) | Perfume inhaled to cure headache [Ojibwa: 87]. |
| *Eriophorum* sp*.*  Cyperaceae, Herb | cotton grass (Eng.); linaigrette (Fr.) | Boiled and drunk for stomach problems [Dene: 17].  **Roots:** Used in a mixture with spruce bark or spruce tips (new growth) to treat cancer [Dene: 17]. |
| **Eryngium* *aquaticum* L.  Apiaceae, Herb | button snakeroot (Eng.); panicaut aquatique (Fr.) | Remedy for snake bite. Mixed with *Iris versicolor* and used as a febrifuge or diuretic [44]. |
| **Erysimum cheiranthoides* L.  Cruciferea, Herb | wormseed mustard (Eng.); vélar giroflée, herbe au chantre (Fr.); o'zawa'bigwûn (Ojibwa) | **Roots:** Decoction used to treat skin eruptions [Ojibwa: 47]. |
| *Eupatorium maculatum* L.  Asteraceae, Herb | joe-pye-weed (Eng.); eupatoire maculée (Fr.); me'skwana'k bû'giso'wĭn (Ojibwa) | For menstrual disorders and venereal diseases, or to facilitate childbirth [Algonquin: 69].  **Roots:** Decoction used as a wash for inflamed joints [Ojibwa: 47]. |
| *Eupatorium perfoliatum* L.  Asteraceae, Herb | thoroughwort, common boneset (Eng.); eupatoire perfoliée, herbe à souder (Fr.); siabuksing, sasabwaksing, niya'wibûkûk' (Ojibwa); maladag'kwinbisoun (Abenaki) | Used as general medicine [Mi'kmaq: 62]. Steeped with other plants (*Pyrola uliginosa*, *Baptisia tinctoria*, *Galium aperine*, *Aralia racemosa*, *Streptopus amplesifolius*, *Acer pennsylvanicum*) and used for gonorrhea, kidney troubles and blood spitting [Algonquians: 63]. Used to strengthen the bones [Abenaki: 67]. Decoction used for fever and cold [44]. Chewed and bound on a rattle-snake bite as a poultice to draw out the poison. Boiled with wild bergamot (*Monarda fistulosa*) to make a fomentation to be applied for rheumatism [Ojibwa: 85].  **Roots:** Used for menstrual disorders [Ojibwa: 85]. Steeped and liquid taken to treat ulcers [Mi'kmaq: 62]. |
| *Eupatorium purpureum* L.  Asteraceae, Herb | joe-pye weed (Eng.); eupatoire pourpre (Fr.); biaskagemesek (Ojibwa) | Boiled and the vapors inhaled to treat cold. Mixed with other species and used to counteract the effects of a miscarriage [Ojibwa: 85]. |
| *Euphorbia corollata* L.  Euphorbiaceae, Herb | flowering spurge (Eng.); euphorbe pétaloïde (Fr.); cabosî'kûn, (Ojibwa) | **Roots:** Pounded and taken as an infusion before eating as a physic [Ojibwa: 87]. |
| *Euphorbia* sp.  Euphorbiaceae, Herb | spurge (Eng.); euphorbe (Fr.) | **Leaves:** Tea used to treat diabetes [Algonquin: 69]. |
| *Eurybia* *macrophylla* (L.) Cass.  **Syn.:** *Aster* *macrophyllus* L.  Asteraceae, Herb | large-leaved aster (Eng.); aster à grandes feuilles, pétouane (Fr.); naskosi'îcûs (Ojibwa) | **Roots:** Tea from young roots used to bathe the head for headache [Ojibwa: 87]. |
| *Euthamia* *graminifolia* (L.) Nutt.  **Syn.:** *Solidago* *graminifolia* (L.) Salisb.  Asteraceae, Herb | grass leaf goldenrod (Eng.); verge d'or graminifoliée (Fr.); wasa'waskwûne'k (Ojibwa) | **Flowers:** Infusion used for chest pain [Ojibwa: 87].  **Roots:** Decoction used in lung trouble [Ojibwa: 47]. |
| *Evernia mesomorpha* Nyl.  Parmeliaceae, Lichen | spruce moss (Eng.); k'itsa^n^jú (Chipewyan) | Cooled decoction used as eye drops to treat snow blindness [Dene: 13; Chipewyan: 92]. |
| *Fagus grandifolia* Ehrh.  Fagaceae, Tree | beech (Eng.), hêtre à grandes feuilles (Fr.); šewe-minš (Ojibwa); miki'kwimus (Malecite) | **Bark:** Used as a general medicine [Mi'kmaq: 62]. Mixed with bark of *Betula lenta* and *Cornus sericea* and used for pulmonary trouble [Ojibwa: 85].  **Leaves:** Applied to sores [Malecite: 65]. Soothing to the nerves and the stomach and help stimulate appetite [Mi'kmaq: 62].  **Bark and leaves:** Mixed with water and used as a tonic, antiseptic, or to heal ulcers, liver, kidney or bladder [Mi'kmaq: 62]. |
| *Fomes fomentarius* (L. Ex Fries) J. Kickx f.  Polyporaceae, Fungi | tinder fungus (Eng.); amadouvier (Fr.); wāsāsukwītwī, wāsaskwītoy, posākan (Cree). | Fruiting body burned to repell mosquitoes and flies [Metis: 13). Dry, spore producing layer cut into matchstick shaped pieces and placed on the skin and burned to produce counter-irritation to treat arthritis [Cree: 95]. |
| *Fomes officinalis*  Polyporaceae, Fungi | agarikon (Eng.); polypore officinal (Fr.); wah-pah-toos (Cree) | Pulverized and applied to frostbite [Cree: 76]. |
| *Fomes pinicola* (Swartz) Cooke  Polyporaceae, Fungi | polypore marginé, amadouvier des pins (Fr.); mech-quah-too (Cree) | Powdered and mixed with water to make a paste applied to wounds or taken internally as an emetic [Cree: 76]. Smoked to treat headache [Dene: 98]. |
| *Fragaria* *vesca* L. ssp. *bracteata* (A. Heller) Staudt  **Syn.:** *F. bracteata* A. Heller  Rosaceae, Herb | woodland strawberry (Eng.); fraisier (Fr.) | **Leaves, roots and fruits:** Mixed with boiling water and used as a blood purifier and blood-building agent [Mi'kmaq: 62].  **Leaves or roots:** Tea given as a gargle for gum problems, or used in diarrhoea or dysentery, weakness of the intestines, infections of urinary organs, or to prevent night sweats [Mi'kmaq: 62].  **Fruits:** Heart medicine [Dene: 17].  **Runners:** Boiled with '*Petasites sagittatus* and '*Pyrola asarifolia* and drunk as a medicine [Dene: 17]. |
| *Fragaria virginiana* Duchesne.  Rosaceae, Herb | wild strawberry (Eng.); fraisier des champs (Fr.); ode'imĭnĭdji'bĭk (Ojibwa); otehimina, otīhīminah, okdeamena, owtiyhiymin, otīhīminipukwah (Cree); | Steeped with *Rubus triflorus* and given in irregular menstruation [Malecite: 65].  **Whole plant:** Decoction drunk to treat heart problems [Cree: 13]. Boiled and the liquid drunk as a treatment for diarrhoea [Metis: 13; Cree: 93].  **Leaves:** Used as an astringent [Ojibwa: 88].  **Roots:** Used as a diuretic [Ojibwa: 88]. Decoction used in cholera [Ojibwa: 47] and tea in stomach ache [Ojibwa: 87]. Herbal water taken for heart problems [Cree: 93].  **Fruits:** Heart medicine [Dene: 17].  **Runners:** Boiled with '*Petasites sagittatus* and '*Pyrola asarifolia* and drunk as a medicine [Dene: 17]. |
| *Fragaria* *virginiana* Duchesne ssp. *glauca* (S. Watson) Staudt  Rosaceae, Herb | smooth wild strawberry, Virginia strawberry (Eng.); fraisier glauque (Fr.) | **Roots:** Burned and the ash mixed with water and placed on open sores [Dene: 98]. |
| *Fraxinus americana* L.  Oleaceae, Tree | yellow ash (Eng.); frêne d'Amérique, frêne blanc (Fr.); ôgmakw (Abenaki) | Used as an emmenagogue [Abenaki: 67].  **Leaves:** Strong decoction given as a cleanser after delivery [Algonquians: 63; Mi'kmaq: 60].  **Wood:** Smoke used to treat earache [Algonquin: 68]. |
| *Fraxinus nigra* Marsh.  Oleaceae, Tree | black or water ash (Eng.); frêne noir, frêne gras (Fr.); a'gimak' (Ojibwa) | **Inner bark:** Soaked in water and the liquid applied to sore eyes [Ojibwa: 84]. |
| *Fraxinus* *pennsylvanica* Marsh.  Oleaceae, Tree | red ash (Eng.); frêne de Pennsylvanie, frêne rouge (Fr.); a'gîma'k (Ojibwa) | **Inner bark:** Mixed with other plants and used as a tonic [Ojibwa: 87]. Tea used against weariness and depression [Atikamekw: 73]. |
| *Fraxinus* sp.  Oleaceae, Tree | ash (Eng.); frêne (Fr.); a'gimak' (Ojibwa) | **Log or branch:** Placed in fire, sap collected from the opposite end and used to treat earache [Algonquin: 69].  **Inner bark:** Decoction taken internally as a tonic and stimulant [Ojibwa: 47]. |
| **Galeopsis tetrahit* L.  Lamiaceae, Herb | hemp nettle (Eng.); galéopside à tige carrée (Fr.); amisko wehkuswa (Cree) | Tea drunk to calm nerves, reduce hyperactivity in children, as a spring tonic, to restore appetite, and to treat bad breath [Dene: 13].  **Leaves:** Chewed to treat severe hiccups [Dene: 13]. |
| *Galium aparine* L.  Rubiaceae, Herb | goose grass (Eng.); gaillet gratteron (Fr.); sakate'bwi (Ojibwa) | Used for gonorrhea, kidney troubles and for blood spitting [Algonquians: 63].  **Whole plant:** Tea used as a diuretic, in kidney trouble, gravel, stoppage of urine, and allied ailments [Ojibwa: 87].  **Stem:** Dipped in cold water and rubbed on rashes or other skin troubles [Algonquians: 63]. |
| *Galium boreale* L.  Rubiaceae, Herb | northern bedstraw (Eng.); gaillet boréal (Fr.) | Used as a diuretic [Cree: 42]. Flowering plant boiled and drunk to treat stomach ache [Dene: 17]. |
| *Galium* *tinctorium* (L.) Scop.  Rubiaceae, Herb | small cleaver (Eng.); gaillet des teinturiers (Fr.); waboskîki'mînûn (Ojibwa) | **Whole plant:** Tea used for beneficial effects upon the respiratory organs [Ojibwa: 87]. |
| *Galium* *trifidum* L.  Rubiaceae, Vine | small bedstraw (Eng.); gaillet trifide (Fr.); Ojîbwe'owe'cûwûn (Ojibwa) | Tea used for skin diseases such as eczema, ringworm and scrofula [Ojibwa: 87]. |
| Gaultheria hispidula (L.) Muhl. ex Bigelow  Ericaceae, Shrub | creeping snowberry, moxieplum (Eng.); petit thé (Fr.); wabos'obûgons' (Ojibwa) | **Leaves:** Tea used as a tonic [Algonquin: 69]. Used on cuts, burns, against tapeworms and to treat venereal diseases [Ojibwa: 19]. Boiled and taken for congested chest [Cree: 80]. Herbal water taken for fever and high blood pressure. Boiled and given to babies as a soother during teething [Cree: 93].  **Fruits:** Used in diabetes [Cree: 82, 83]. |
| *Gaultheria procumbens* L.  Ericaceae, Shrub | teaberry, checkerberry, wintergreen (Eng.); thé des bois, gaulthérie couchée (Fr.); pileuminan (Innu); pirebow (Atikamekw); winsibog, wînîsi'bûgûd (Ojibwa) | Mixed with mayapple, wild sarsaparilla, wild spikenard, burdock, *Taraxacum officinale*, prickly ash, black berry, sassafras, prince's pine, and black birch in a decoction used as a "spring and fall medicine" [Ojibwa: 85]. Steeped and taken as a blood flow regulator to prevent heart attacks [Mi'kmaq: 62]. Tea used in colds, headaches, and general discomforts [Atikamekw: 73; Algonquin: 69].  **Leaves:** Used for colds, chewed to improve breathing [Algonquin: 55]. Tea used to cure rheumatism and as a tonic [Ojibwa: 87]. Used to make a chest plaster to treat cold [Atikamekw: 73].  **Fruits:** Used as a stimulant and diuretic [Algonquin: 55] |
| *Geocaulon* *lividum* (Richardson) Fernald  **Syn.:** Comandra lividum Richardson  Santalaceae, Herb | northern comandra, false toadflax (Eng.); comandre livide (Fr.); sasjie (Chipewyan) | Medicinal plant [Cree: 42].  **Fruits:** Used for persistent chest trouble [Dene: 13; Chipewyan: 92]. |
| *Geranium maculatum* L.  Geraniaceae, Herb | wild geranium, cranesbill (Eng.); géranium maculé (Fr.); be'cigodji'bigûk, pesigunk, o'sawaskwîni's (Ojibwa) | **Roots:** Steeped and used for diarrhoea [Ojibwa: 85]. Powdered and put into sore mouth [Ojibwa: 47, 87]. Used to treat flux [Ojibwa: 87].  **Rhizomes:** Used as an intestinal astringent [Ojibwa: 44]. |
| *Geum* *aleppicum* Jacq.  **Syn.:** *G. strictum* Aiton  Rosaceae, Herb | Alep avens (Eng.); benoîte d'Alep (Fr.); makwanimiga'kil (Maletice); kākwīthitamōwask (Cree); ne'bone'ankwe'âk (Ojibwa) | **Roots:** Steeped and used for cough and cold [Maletice: 65]. Weak decoction taken internally for cough and soreness in the chest [Ojibwa: 84]. Decoction alone or mixed with other plants given to treat teething pain. Decoction used for sore tooth, sore throat, or to induce sweating [Cree: 95]. |
| *Geum* *canadense* Jacq.  Rosaceae, Herb | white avens, Canada avens (Eng.); benoîte du Canada (Fr.) | **Roots:** Used in "female weakness" [Ojibwa: 47]. |
| *Geum macrophyllum* Willd.  Rosaceae, Herb | large-leaved avens (Eng.); benoîte à grande feuilles (Fr.); kākwīthitamōwask, saw-gee-too-wusk (Cree); wica'wasa'konek (Ojibwa) | Used as a "female remedy" [Ojibwa: 87] or as component of a compound medicine [Metis: 13].  **Roots:** Decoction used in combination with other plants to treat teething pain [Cree: 95]. |
| *Geum rivale* L.  Rosaceae, Herb | water avens, chocolate root (Eng.); benoîte des ruisseaux (Fr.); kinipagwusk (Cree); egwitkil (Malecite); mikotcepik (Atikamekw) | **Roots:** Strong decoction given in dysentery, cough and cold, particularly for children [Algonquians: 63]. Steeped and given in diarrhoea [Malecite: 65]. Boiled four times and the fourth water is used to treat blood spitting [Atikamekw: 73]. Tea used to facilitate childbirth [Cree, Metis: 13]. |
| *Geum* *triflorum* Pursh  **Syn.:** *Sieversia* *ciliata* (Pursh) G. Don  Rosaceae, Herb | prairie smoke (Eng.); benoîte à trois fleurs (Fr.); ne'baneya'nekweäg' (Ojibwa) | **Roots:** Used as a tonic after childbirth [Ojibwa: 19]. Decoction taken in indigestion. Dried and chewed as a tonic and stimulant [Ojibwa: 47]. |
| *Glyceria* *canadensis* (Michx.) Trin.  Poaceae, Herb | rattlesnake grass, Canada managrass (Eng.); glycérie du Canada (Fr.); anagon'wûck (Ojibwa) | **Roots:** Used as a "female remedy" [Ojibwa: 87]. |
| *Gnaphalium* sp.  Asteraceae, Herb | gnaphale (Fr.) | Decoction used for coughing and consumption [Algonquians: 63]. |
| *Grindelia squarrosa* (Pursh) Dunal.  Asteraceae, Herb | curly-cup gumweed (Eng.); herbe à gomme commune (Fr.); kāpasakwāk maskīhkīh, kah pus kun askik (Cree) | Used in gonorrhoea and to prevent pregnancy [Cree: 76].  **Flowers:** Tea used to treat migraines and venereal diseases [Metis: 13]. |
| Gymnocarpium disjunctum (Rupr.) Chin  **Syn.:** Dryopteris disjuncta (Rupr.) Morton  Dryopteridaceae, Herb | oak fern (Eng.); dryoptéride disjointe (Fr.) | Used to treat cholera [Abenaki: 67]. |
| *Gymnocarpium dryopteris* (L.) Newman  **Syn.:** *Dryopteris* *linnaeana* C. Chr.  Dryopteridaceae, Herb | western oak fern (Eng.); gymnocarpe du chêne (Fr.); oraganiockokuk (Atikamekw) | **Leaves:** Crushed and rubbed on face to prevent or to sooth mosquito bite [Atikamekw: 73]. |
| *Hamamelis virginiana* L.  Hamamelidaceae, Tree | witch hazel (Eng.); hamamélis de Virginie, café du diable (Fr.); nsakemižins (Ojibwa) | **Inner bark:** Used as an emetic, especially in cases of poisoning. Steeped and strained as a lotion for skin trouble, and as a wash for sore eyes [Ojibwa: 85].  **Bark:** Infusion used for bruises, piles and hemorrhages [44]. |
| *Hedysarum* *alpinum* L. **Syn.:** *H. alpinum* L. ssp. *americanum* (Michx.) Fedtsch.  Fabaceae, Herb | alpine hedysarum, bear root, Indian carrots (Eng.); sainfoin alpin (Fr.); treh (Dene) | **Roots:** Eaten raw or with duck or fish oil to increase appetite or to relieve diarrhoea [Dene: 99]. Burned and smoke used to relieve sore eyes [Dene: 98]. |
| *Helenium autumnale* L.  Asteraceae, Herb  **Syn:** *Helenium* *autumnale* L. var. *montanum* (Nutt.) Fernald  Asteraceae, Herb | sneezeweed, swamp sunflower (Eng.); hélénie automnale (Fr.); cācāmōsikan (Cree) | **Flowers:** Dried, crumbled and inhaled to trigger sneezing in order to treat a headache [Cree: 13, 96]. |
| *Helianthus* *occidentalis* Riddell  Asteraceae, Herb | sunflower (Eng.); pŭkite'wŭkbŏku^n^s' (Ojibwa) | **Roots:** Crushed and applied to bruises and contusions [Ojibwa: 84]. |
| Heliopsis helianthoides (L.) Sweet var. scabra (Dunal) Fernald  **Syn.:** H. scabra Dunal  Asteraceae, Herb | ox-eye-daisy, false sunflower (Eng.); héliopsis faux-hélianthe (Fr.); gi'zĭso'bûgons' (Ojibwa) | **Roots:** Used as a tonic [Ojibwa: 47]. |
| *Hepatica* *nobilis* Schreb. var. *obtusa* (Pursh) Steyerm.  **Syn.:** *H.* *americana* (DC.) Ker Gawl.  Ranunculaceae, Herb | hepatica, American liverleaf (Eng.); hépatique d'Amérique (Fr.); gabisan'ikeäg' (Ojibwa) | **Roots:** Decoction taken internally in convulsions [Ojibwa: 47]. |
| *Hepatica triloba* Chaix.  Ranunculaceae, Herb | hepatica (Eng.); hépatique trilobée (Fr.); pne-uzidi^n^, pne^n^-obogo^n^s (Ojibwa) | Used as an ingredient in poultices for inflamations and bruises, and also as a remedy for liver ailments [Ojibwa: 85].  **Roots:** Mixed with sweet cicely (*Osmorhiza longistylis*) and tall anemone (*Anemone virginiana*) and boiled to make a remedy for amenorrhoea [Ojibwa: 85]. |
| Heracleum maximum Bartram  **Syn.:** *H. lanatum* Michx.  Apiaceae, Herb | cow parsnip, indian rhubarb (Eng.), berce très grande (Fr.); pukwanatik, pakwānāhtik, askīskatask, askīwīskātask, ōskātaskwistikwān, piygwanā(h)tik, pick quan ah tick (Cree); sewapo'kil (Malecite); bi'bîgwe'wûnûck (Ojibwa); uîpîtakâshk^u^ tshîtshue(Montagnais) | Steeped with *Calmus* and given in cholera [Malecite: 65]. Dried, chopped, and rubbed on aching or sore body parts. Cooled decoction used to bathe sore body parts, and the decoction can be drunk for colds [Cree: 13]. Used to keep sickness away and in combating cold and/or influenza [Mi'kmaq: 62].  **Roots:** Fresh or dried root applied to an aching tooth [Cree: 13, 76, 93]. Chewed and juice swallowed to treat tooth ache [Cree: 96]. Part of a cancer medicine. Grated and mixed with warm water to make a paste applied to swollen legs. [Metis: 13]. Tea used for throat pain [Montagnais: 71]. Fresh root pounded and applied to sores as a poultice [Ojibwa: 87]. Powdered and made into a paste applied to boils, swellings and chancre [Cree: 76]. Mixed with other plants (*Nuphar variegatum, Acorus calamus*) to make a poultice applied to painful limbs or used for headache [Cree: 95]. Steeped and taken as a tea for blood purification, to prevent and cure smallpox, and in consumption [Malecite: 65]. Chewed dry to treat sore throat, decoction used to gargle, and poultice applied to boils and sores [Ojibwa: 47, 88]. Decoction used for arthritis [Cree: 13, 80]. Boiled and used to clean skin infections [Cree: 80]. Decoction drunk as a cough medicine [Dene: 98].  **Roots and leaves:** Steam from boiling inhaled to purify the body [Cree: 13].  **Roots and flowers:** Dried, pounded and made into a poultice used in boils [Ojibwa: 47]. |
| *Heuchera richardsonii* R. Br.  Saxifragaceae, Herb | Richardson's alumroot (Eng.); heuchère de Richardson (Fr.); pithīkōcīpihk (Cree); ciwade'imĭn'ĭbûg (Ojibwa) | **Roots:** Chewed or made into a decoction taken in diarrhoea. Infusion used to wash sore eyes [Cree: 95]. Dried, chewed and the juice swallowed in stomach pain. Chewed in sore mouth. Decoction used to treat sore eyes [Ojibwa: 47]. |
| *Hierochloe odorata* (L.) Beauv.  Poaceae, Herb | sweet-grass, vanilla grass (Eng.); hiérochloé odorante, foin d'odeur, herbe sainte (Fr.); wehkuskwa, wekus (Cree) | Decoction given to facilitate childbirth for young mothers [Cree: 13]. |
| *Hordeum jubatum* L.  Poaceae, Herb | squirrel-tail (Eng.); orge agréable (Fr.); a'djidamo'wano (Ojibwa) | **Roots:** Dried, pounded, put in a moist cloth with warm water and sopped on the eyes to treat sty or inflammation of lids [Ojibwa: 47]. |
| *Hudsonia tomentosa* Nutt.  Cestaceae, Shrub | ground berry, woolly hudsonia (Eng.); hudsonie tomenteuse (Fr.) | **Whole plant:** Boiled and liquid drunk to purge women blood [Algonquians: 63]. |
| *Humulus* *lupulus* L.  Cannabaceae, Vine | hop (Eng.); houblon commun (Fr.); jiwî'cgoni'bûg (Ojibwa) | Tea acts like saleratus on the system, increasing the excresence of urine and reducing its acidity [Ojibwa: 87]. |
| *Hydrophyllum virginianum* L.  Hydrophyllaceae, Herb | eastern waterleaf (Eng.); hydrophylle de Virginie (Fr.); hu^n^kite'wagūŭs', ne'bîneankwe'ûk (Ojibwa) | **Roots:** Boiled and taken for chest or back pain [Ojibwa: 84; 44]. Used to keep flux in check [Ojibwa: 87]. |
| **Hylotelephium* *telephium* (L.) H. Ohba ssp. *telephium*  **Syn.:** *Sedum* *telephium* L.  Crassulaceae, Herb | garden stonecrop, live-forever (Eng.); orpin pourpre (Fr.); mediawigagil (Malecite) | **Leaves:** Poultice used in boils [Malecite: 65]. |
| *Hymenoxys richardsonii* (Hook.) Cockerell.  Asteraceae, Herb | Richardon's bitterweed, Colorado rubber-plant (Eng.); hymenoxys de Richardson (Fr.) | **Roots:** Ground into a powder and used as a snuff to induce sneezing to clear head and relieve head colds and headaches [Cree: 13]. |
| **Hypericum perforatum* L.  Clusiaceae, Herb | St. John's wort (Eng.); millepertuis commun (Fr.) | Boiled to make a cough medicine [Algonquians: 63]. |
| *Ilex* *mucronata* (L.) Powell, Savolainen & Andrews  **Syn.:** Nemopanthus mucronatus (L.) Loes.  Aquifoliaceae, Tree | mountain holly (Eng.); faux houx, némopanthe mucroné (Fr.); mîckimînû'nîmîc (Ojibwa); wudjiga'nimus (Malecite); kâmatshakâshit shakâu (Montagnais) | **Fruits:** Medicinal [Ojibwa: 86].  **Roots:** Steeped and taken in gravel [Malecite: 65].  **Leaves:** Used to restore lost appetite and to strengthen the stomach [Cree: 44].  **Inner bark:** Scraped in water, boiled and paste applied to head to reduce pain [Montagnais: 71]. |
| Ilex verticillata (L.) Gray  Aquifoliaceae, Tree | winterberry (Eng.); houx verticillé (Fr.); awe'nîsibûg (Ojibwa) | **Bark:** Used in diarrhoea [Ojibwa: 86]. |
| Impatiens capensis Meerb.  **Syn.:** *I. biflora* Walter  Balsaminaceae, Herb | spotted jewelweed, Cape touch-me-not (Eng.); impatiente du Cap (Fr.); pesgide'skil (Malecite); wesa'wûs ga'skonêk (Ojibwa) | Steeped and taken to cure jaundice [Malecite: 65]. Fresh juice rubbed on head to cure headache [Ojibwa: 87].  **Stems:** Bruised and applied to cure rash or other skin troubles [Chippewa: 85].  **Leaves:** Steeped to make a medicinal tea [Ojibwa: 87]. |
| *Impatiens pallida* Nutt.  Balsaminaceae, Herb | pale jewelweed, pale touch-me-not (Eng.); impatiente pâle (Fr.) | **Stems:** Bruised and applied to cure rash or other skin troubles [Ojibwa: 85]. |
| *Inonotus obliquus* (Ach. Ex Pers.) Pil.  [Hymenochaetaceae](http://en.wikipedia.org/wiki/Hymenochaetaceae), Fungi | tinder fungus (Eng.); polypore oblique (Fr.); pōsākan, wīsakīchak omīkī, wīsakecak omikīh (Cree) | Decoction drunk to treat heart conditions or mixed with other plants in a medicinal tea for high blood pressure. Soaked overnight and given to help an underweight child gain weight [Cree: 13]. Placed on the skin and burned to produce a counter-irritation in the treatment of arthritis [Cree: 95]. |
| **Inula helenium* L.  Asteraceae, Herb | elecampane (Eng.); inule aulnée, elecampane (Fr.) minsisimatek (Malecite); | **Roots:** Powdered and snuffed to treat headache. Steeped and taken in heart trouble [Malecite: 65]. |
| *Iris versicolor* L.  Iridaceae, Herb | muskrat, blue flag, (Eng.); iris versicolore (Fr.); na'bûkûck (Ojibwa); wa-dusk-skwamuk (Atikamekw) | General medicine [Mi'kmaq: 62]. Used as a purgative [Cree: 42]. Remedy for gastric disturbances [44]. Steepped with *Scirpus rubrotinctus* and gargle in sore throat [Malecite: 65].  **Whole plant:** Steamed to keep diseases away. Crushed and mixed with flour to make a poultice placed on painful body parts [Algonquians: 63].  **Roots:** Pounded to a pulp laid on a platain leaf and applied to serofulous sores [Chippewa: 85]. Steeped and used for cholera [Algonquians: 63; Chippewa: 85]. Powdered, mixed with warm water and given to infants to relieve stomach cramps, belching or indigestion [Mi'kmaq: 62]. Pounded and applied to burns and wounds [Atikamekw: 73]. Poultice applied to swellings [Ojibwa: 47]. Boiled in water and taken as physic and emetic [Ojibwa: 87]. |
| *Juglans cinerea* L.  Juglandaceae, Tree | butternut (Eng.); noyer cendré (Fr.); p^c^ga^c^nmus (Malecite) | **Sap:** Boiled down until thick and waxy, mixed with cornmeal, and taken as a cathartic [Chippewa: 85].  **Bark:** Steeped and taken before breakfast as purgative [Malecite: 65]. |
| *Juniperus communis* L.  Cupressaceae, Tree | juniper (Eng.); genévrier commun, genièvre (Fr.); kawins (Chippewa), kahkakew-mina, kahkakewatik, ahaseminanatik, kakakìmìniatik, kākākīmīnātik, kākākīwīmināhtik, māsakiys (Cree); datsá^n^jíé (Chipewyan); tuna'liguk'(malecite); ga'gawan'dagisĭd (Ojibwa); kâkâtshiminânakashî (Montagnais); deetrèe jàk (Dene) | Steeped with Prince's pine and taken in consumption [Malecite: 65].  **Above-ground parts:** Tea used for cold or stomach ache [Dene: 100].  **Fruits:** Inner bark and juice of berries used to treat ulcers [Mi'kmaq: 60]. Stewed and strained and the liquid given for cold, bladder problems, and as a diuretic [Mi'kmaq: 43]. Boiled and taken for chest pains, bad colds, coughs and congestion [Dene: 99]. Used to treat diabetes related symptoms [Cree: 82]. Tea drunk to ease back pain [Dene: 101].  **Roots:** Decoction used in calculus, cystitis and Bright's disease [Mi'kmaq: 43]. Boiled with other plants to make a decoction drunk to treat menstrual cramps [Dene: 13]. Used to treat diabetes [Cree: 82].  **Leaves:** Dried, powdered and dusted on psoriasis and eczema [Mi'kmaq: 43]. Used to treat diabetes [Cree: 82].  **Bark:** Boiled until a jelly forms, used to treat boils [Algonquin: 75]. Mixed with another plant and soaked in warm water but not boiled, then the infusion can be drunk lukewarm to treat aches and pain [Cree: 13].  **Inner bark:** Used in diabetes [Cree: 77]. Softened in water and used as a poultice on wounds [Metis: 13; Cree: 42].  **Twigs/stem:** Tea given to ease bladder pain [Montagnais: 71]. Steeped and used in hair wash or taken as a tonic [Malecite: 65]. Stem debarked and used to make a tea to treat diarrhoea [Metis: 13]. Decoction of a barked procumbent stem or branch used to treat diarrhoea and sore chest. Mixed with other plants to make decoctions to treat "women troubles", teething pain, sickness after childbirth, fever, and cough [Cree: 95].  **Twigs and leaves:** Boiled to make a drink for asthma [Chippewa: 85]. Used to treat diabetes [Cree: 82]. Boiled and steamed to hasten release of the placenta [Dene: 101].  **Cones:** Green cones boiled to make a diuretic remedy for kidney trouble. Can be smoked in a pipe to treat asthma [Metis: 13]. Berry-like cones eaten as a cure-all medicine [Chipewyan: 92].  **Gum:** Applied to wounds. Mixed with brandy and skunk cabbage and taken in tuberculosis. Applied to sprains, and to relieve soreness and pain [Mi'kmaq: 43]. |
| *Juniperus horizontalis* Moench.  Cupressaceae, Shrub | creeping juniper, rocky mountain juniper (Eng.); genévrier horizontal (Fr.); ahaseminanatik, masekesh, masikeskatik (Cree) | **Stems:** Boiled with other plants to make a tea for treating cold and teething, and for use as a general system cleaner [Cree: 13].  **Leaves:** Burned and the smoke inhaled to clear sinuses plugged by a head cold [Metis: 13].  **Berries:** Used for back pain, boiled and taken in urinary tract problems [Cree: 81]. Tea drunk to ease back pain [Dene: 101].  **Branches:** Boiled and steamed to hasten release of the placenta [Dene: 101]. |
| *Juniperus virginiana* L.  Cupressaceae, Tree | red cedar (Eng.); genévrier de Virginie (Fr.); miskwa'wak (Ojibwa) | **Leaves and twigs:** Used as a diuretic [Cree: 42].  **Leaves and fruits:** Bruised and used internally to treat headache [Ojibwa: 84].  **Twigs:** Boiled with little twigs of *Taxux canadensis* and taken internally to treat rheumatism [Ojibwa: 47]. |
| *Kalmia angustifolia* L.  Ericaceae, Shrub | lambkill, sheep laurel, (Eng.); kalmia à feuilles étroites, crevard de moutons (Fr.); mikwëwa'lwql (Malecite); wesajebo (Atikamekw); jabak (Abenaki); uschipikwh (Cree); uishetshipuk, uîshatshipukua (Innu) | Steeped in hot water and soaked in a woolen pad or rubbed on the limbs or body to relieve pain and stiffness [Montagnais: 70]. Used for head colds [Abenaki: 67]. Tea used for cold and as a tonic [Innu: 72]. Mixed with *Sorbus americana* in a compress used on cuts and wounds [Innu: 72].  **Bark:** Tea used as an emetic [Algonquin: 75]. Steeped and used externally for rheumatism, sore legs and feet [Mi'kmaq: 62].  **Leaves:** Although the leaves are known to be poisonous, a very small quantity can be steeped and drunk for cold, headache and backache. A hot water infusion (very poisonous if very strong) can also be drunk for stomach pain. Poultice applied to head to cure headache [Atikamekw: 73; Algonquians: 63]. Used in diabetes [Cree: 77; 83]. Singed by fire, crushed and used in colds [Algonquin: 69]. Salve prepared from fresh parts and applied to swelling or sprain [Malecite: 65]. Tea used for throat pain [Montagnais: 71]. Applied to swells, arthritis or knee pain [Montagnais: 71]. Used to treat diabetes [Cree: 82].  **Twigs, leaves and flowers:** Used in bowel complaints and as a tonic [Cree: 42].  **Twigs:** Used to prepare a decoction for throat pain and mouth infections [Montagnais: 71].  **Roots:** Used to treat diabetes [Cree: 82]. |
| *Kalmia* *latifolia* L.  Ericaceae, Tree | mountain laurel (Eng.); kalmia à feuilles larges (Fr.) | Used in diarrhoea [Cree: 42]. |
| *Kalmia* *polifolia* Wangenh.  Ericaceae, Shrub | pale bog laurel (Eng.); kalmia à feuilles d'andromède (Fr.); nágodhts'ëlé (Chipewyan) | **Leaves:** Chewed or drunk as tea to treat diarrhoea [Chipewyan: 92]. |
| *Lactuca* *biennis* (Moench) Fernald  **Syn.:** *L. spicata* (Lam.) Hitchc.  Asteraceae, Herb | tall lettuce, blue lettuce (Eng.); laitue bisannuelle (Fr.); dadoca'bo (Ojibwa) | Tea from plant given to women with caked breasts to induce lactation [Ojibwa: 87]. |
| *Lactuca canadensis* L.  Asteraceae, Herb | wild lettuce, Canada lettuce (Eng.); laitue du Canada (Fr.); odjici'gomĭn (Ojibwa) | Juice used in skin warts [Chippewa: 47]. |
| *Laportea* *canadensis* (L.) Weddell  Urticaceae, Herb | wood nettle, Canada nettle (Eng.); laportéa du Canada, ortie du Canada (Fr.); masa'natîk (Ojibwa) | **Roots:** Tea used as a diuretic [Ojibwa: 87]. |
| **Lappula* *squarrosa* (Retz.) Dumort.  Boraginaceae, Herb | stickweed (Eng.); bardanette (Fr.); ozaga'tĭgomĕ^n^s (Ojibwa) | **Roots:** Powdered and sniffed or fumes inhaled for headache [Ojibwa: 84]. |
| *Larix* *laricina* (Du Roi) K. Koch  Pinaceae, Tree | tamarack, larch (Eng), mélèze laricin, épinette rouge (Fr.); wakinakum, wakinakun, wakinākin, wāginagun, waachinaakin (Cree); poka'mus (Malecite); mŏsh'kīkiwa'dik, mû'ckîgwa'tîg (Ojibwa); mackigwatuk (Atikamekw); uatshinakan (Innu/Montagnais); oblanda'gasouk, pôbnôdageso (Abenaki); ts'iiteenjùh, diweh (Dene); nídhe (Chipewyan) | Used to stop vomiting [Cree: 95]. Boiled with crowberry and drunk to cure cold [Dene: 101]. Used on boils [Cree: 80].  **Branches:** Decoction used as a diuretic [Algonquians: 63]. Tea used for stomach pain, cold, fatigue, or for general health [Dene: 99]. Tea from fresh branches used to treat stomach problems [Dene: 101].  **Bark:** Used in suppurating wounds [Mi'kmaq: 60]. Steeped with alder bark (*Alnus incana*) to make an infusion for anemia [Chippewa: 85]. Steeped with spruce and fir bark and given in gonorrhoea [Melacite: 65]. Used to make a cough medicine [Abenaki: 67]. Used in chronic bronchitis, in chronic inflammation of the urinary passages and in phases of hemorrage [Ojibwa: 87]. Applied as a compress for eye problems, wounds and swelling [Montagnais: 71]. Boiled, cooled and taken as a cough syrup, for sore throat or mouth. Broth taken for stomach ache [Cree: 81].  **Inner bark:** Used as a poultice or boiled to make a wash for burns, boils [Dene: 13; Chipewyan: 92], frostbite [Cree: 13], hemorrhoids, infected wounds [Cree, Metis: 13], or cuts [Cree: 13]. Grinded finely, mixed with animal fat and used for skin sores and burns [Cree: 81]. Tea drunk to treat depression, used as an eye wash or for ear irritation [Cree: 13]. Green strips applied to burns and used to make a tea to treat sore threat [Algonquin: 75]. Chopped (fresh or dried) and applied to burns [Ojibwa: 47]. Used in diabetes [Cree: 77, 83]. Boiled and given for sores and swelling [Algonquians: 63]. Chewed raw or boiled and taken to treat sore throat [Cree: 81]. Boiled and tea used to wash a wound. Tea drunk for pain relief, stomach problems, mouth infections, sore throat, fever, cold, flu and bleeding. Boiled with spruce cones and applied to wounds [Dene: 101]. Used to treat diabetes [Cree: 82]. Herbal water taken for heart problems [Cree: 93].  **Needles and inner bark:** Used for cough and to prepare poultices for treating infections [Atikamekw: 69; Innu: 72].  **Inner bark and wood:** Poultice applied to frostbite and deep cuts [Cree: 95].  **Gum:** Chewed to relieve indigestion [Cree: 13]. Used in deep cuts, wounds and burns [Cree: 81]. Applied fresh or boiled on cuts [Cree: 80]. Amber applied to wounds, or boiled and rubbed to cure sore mouth [Cree: 81].  **Sap:** Placed in eyes to treat snow blindness [Cree: 80].  **Leaves:** Used as inhalant and fumigator [Ojibwa: 87]. Tea used as a laxative [Atikamekw: 73].  **Cones**: Used to prepare a decoction for jaundice [Montagnais: 71]. Tea used to soothe cold and relieve headache [Dene: 99].  **Twigs and gum:** Used for cough [Montagnais: 71].  **Leaves and bark:** Crushed and used in headache [Ojibwa: 84].  **Roots and bark:** Tea used as a general medicine [Ojibwa: 86]. Mixed with another plant in a decoction drunk daily to treat arthritis, pain, or cold [Cree: 13].  **Pulp:** Boiled and used to treat impetigo, or used to wash leg sores [Cree: 81]. Wrapped in cloths and placed on burns. Boiled, strained and taken in cough and cold [Cree: 80].  **Wood:** Chewed to treat sore throat [Cree: 81].  **Bark, boughs and roots:** Tea used to treat cold [Dene: 100].  **Roots:** Boiled and liquid used to heal wounds [Dene: 98]. |
| *Lasallia* *papulosa* (Ach.) Llano  **Syn.:** Umbilicaria papulosa (Ach.) Nyl.  Umbilicariaceae, Lichen | tripes-de-roches (Fr.); uâkuanâpishk^u^ (Montagnais) | Tea used for urinary problems [Montagnais: 71]. |
| *Lathyrus ochroleucus* Hook.  Fabaceae, Vine | creamy vetchling, pale vetchling (Eng.); gesse jaunâtre (Fr.); bûgwa'dj ûk pîni'k mîne'bûg (Ojibwa) | Used for stomach trouble [Ojibwa: 87]. |
| *Lathyrus* *venosus* Muhl. ex Willd.  Fabaceae, Herb | veiny vetchling, wild pea (Eng.); gesse veinée (Fr.); mĭ'nĭsĭno'wûck (Ojibwa) | **Roots:** Decoction applied to bleeding wounds or taken internally as a tonic and stimulant. Decoction (alone or mixed with roots of *Apocynum androsaemifolium*) taken internally to treat convulsions [Ojibwa: 47]. |
| *Lepidium virginicum* L.  Brassicaceae, Herb | wild peppergrass (Eng.); lépidie de Virginie (Fr.) | General medicinal plant [Ojibwa: 86]. |
| *Leucanthemum vulgare Lam.  **Syn.:** Chrysanthemum leucanthemum L.  Asteraceae, Herb | ox-eye daisy (Eng.); chrysanthème leucanthème, marguerite (Fr.); iskwew owehowina (Cree) | **Flowers and leaves:** Mixed with other plants in a tea that soothes nerves in adults and hyperactivity in children [Metis: 13]. |
| *Leymus* *mollis* (Trin.) Pilg.  Poaceae, Herb | sea limegrass, American dunegrass (Eng.); seigle de mer (Fr.); kawabash (Cree) | Used to treat diabetes [Cree: 82]. |
| *Lilium canadense* L.  Liliaceae, Herb | Canada lily, wild yellow lily (Eng.); lis du Canada (Fr.); walbatweka.'gil (Malecite); wĭnabojo'bikwûk' (Ojibwa) | **Roots:** Used in stomach disorders [Algonquin: 69]. Given with *Viburum lantago* in irregular menstruation [Malecite: 65]. Decoction used externally to cure bites of poisonous reptiles [Ojibwa: 47]. |
| *Lilium philadelphicum* L.  Liliaceae, Herb | orange-red lily, Philadelphia lily (Eng.); lis de Philadelphie (Fr.); wapayoominusk, wākican, wākiychān (Cree); miškodé-pin, miskode-pin (Ojibwa) | **Bulbs:** Boiled to make a poultice to be applied to wounds and contusions [Chippewa: 85].  Mixed with the root of *nibnatugu^n^s,* boiled and applied as a poultice on the bite of a dog [Chippewa: 85]. Boiled into a soup and taken to treat appendicitis. Tubers sticked into cavities to treat toothache [Cree: 93].  **Roots:** Part of a compound medicine used for heart problems [Dene: 13]. Used in stomach disorders [Algonquin: 69]. Grinded, soaked in warm water and applied as a poultice to swelling or bruise. Mixed with staghorn sumach, blackberry root, mountain holly and mountain raspberry root and used in consumption, cough, and fever [Malecite: 65]. |
| *Limonium* *carolinianum* (Walter) Britton  Plumbaginaceae, Herb | sea lavender (Eng.); limonium de Caroline (Fr.) | **Roots:** Grinded and taken with water before each meal for consumption with hemorrhage [Mi'kmaq: 62]. |
| **Linaria* *vulgaris* Mill.  Scrophulariaceae, Herb | butter and eggs (Eng.); linaire vulgaire (Fr.); owacawa'skwûneg (Ojibwa) | **Whole plant:** Dried and used with other plants in the sweat lodge as a bronchial inhalant [Ojibwa: 87]. |
| *Linnaea borealis* L.  Caprifoliaceae, Herb | twin flower (Eng.), linnée boréale (Fr.); pâshpâshtshu (Montagnais) | Paste used to cure inflamation of the limbs [Algonquians: 63]. Tea used to fight chest pain [Montagnais: 71].  **Whole plant:** Tea used in menstrual difficulties, and to ensure good health of pregnant women's babies [Algonquin: 69]. |
| *Lobelia inflata* L.  Campanulaceae, Herb | Indian tobacco (Eng.); lobélie gonflée (Fr.) | **Leaves:** Dried and smoked as an emetic [44] and for general health [Mi'kmaq: 62]. Smoke blown into ear to treat earache [Mi'kmaq: 60]. |
| *Lobelia kalmii* L.  Campanulaceae, Herb | Ontario lobelia, Kalm's lobelia (Eng.); lobélie de Kalm (Fr.) | Used as an emetic [Cree: 42]. |
| *Lonicera* *canadensis* Bartram ex Marsh.  Caprifoliaceae, Shrub | American fly honeysuckle (Eng.); chèvrefeuille du Canada (Fr.) | **Vines:** Steeped for urinary troubles [Algonquians: 63]. |
| *Lonicera dioica* L. **Syn.:** *L. dioica* L. var. *glaucescens* (Rydb.) Butters  Caprifoliaceae, Vine | glaucous honeysuckle, limber honeysuckle (Eng.); chèvrefeuille dioïque (Fr.); šabankuk (Ojibwa); Sīpāhtik, sīpaminukusīatik, sīpaminitasīatik, sīpāminakasīwā(h)tik, gāganōnskīwaskwah, gagīnawonskiwaskwah, pay(h)payā(h)tik (Cree) | Soaked in water and used to wash hair to make it grow longer [Cree: 13].  **Stems:** Mixed with *Cornus sericea* and *neakmižinš* and used as a diuretic [Ojibwa: 85]. Nodes cut off and discarded and the internodes boiled to make a drink taken as a diuretic [Cree: 13]. Decoction used to treat blood clotting after childbirth, and venereal diseases [Cree: 95]. Tea taken in kidney problems [Cree: 96]. Boiled and taken as a diuretic [Dene: 98]. Herbal water taken in heart ailments [Cree: 93].  **Bark:** Mixed with other species in an infusion used instead of drinking water in case of dysuria [Ojibwa: 85]. Tea used in menstrual difficulties, to treat kidney stones, or as a cathartic [Algonquin: 69].  **Inner bark:** Infusion used as a diuretic [Cree: 95].  **Roots:** Decoction used in lung trouble [Ojibwa: 47]. Decoction drunk to treat chronic bladder problems [Cree: 13] or to relieve constipation [Dene: 98]. |
| *Lonicera* *involucrata* (Richardson) Banks ex Spreng.  Caprifoliaceae, Shrub | black twinberry; twinflower, honeysuckle (Eng.); chèvrefeuille involucré (Fr.); payipahtik, pipahtikwa (Cree) | **Stem:** Dried and boiled to make a decoction drunk to treat venereal diseases [Metis: 13]. |
| *Lycoperdon perlatum* Pers.  [Agaricaceae](http://en.wikipedia.org/wiki/Agaricaceae)*,* Fungi | puffball fungus (Eng.); vesse-de-loup perlée (Fr.); wathaman, pesohkan, bībōgīthāmin, bīpōgīthamin, pissoskum, kōkōsiwathaman, kāpwīpocīpathisit, kāpikiy(h)tiypayta (Cree); datsántsíé (Chipewyan) | To stop bleeding from a cut, the mature puffball can be turned open and the inside surface of the skin, with its adhering cottony mass applied to the wound. Spores may also be squirted from the puffball onto the cut and into the nose to stop nosebleeding [Cree, Dene, Metis: 13; Chipewyan: 92]. Spores can also be used as baby powder to prevent chafing [Dene: 13] or to treat weeping sores or burns [Dene: 98, 100]. |
| *Lycoperdon* sp.  [Agaricaceae](http://en.wikipedia.org/wiki/Agaricaceae)*,* Fungi | puffball (Eng.); vesse-de-loup (Fr.); kâpîputepishiti (Montagnais) | Spores inhaled to stop nosebleeding [Dene: 101; Montagnais: 71; Cree: 93, 95] or applied to cuts to stop bleeding [Dene: 17] or to relieve skin rash [Dene: 101]. |
| *Lycopodium clavatum* L.  Lycopodiaceae, Herb | stag's horn, common club-moss (Eng.); lycopode claviforme, courants verts (Fr.) | **Whole plant:** Brewed and used for weakness and fever [Algonquians: 63]. Used in diabetes [Cree: 77, 82].  **Shoots:** Used in diabetes [Cree: 83]. |
| *Lycopodium* *dendroideum* Michx.  Lycopodiaceae, Herb | round-branched tree-clubmoss, ground pine, bear paw (Eng.); lycopode dendroïde (Fr.) | General medicinal plant. Boiled and drunk as a purgative in case of biliousness [Algonquians: 63]. Mixed with *Diervilla lonicera* and used as a diuretic [Ojibwa: 87]. |
| *Lycopodium* *obscurum* L.  Lycopodiaceae, Herb | ground pine (Eng.); lycopode foncé (Fr.) | Mixed with twigs of *Picea glauca* and inner part of wood of *Ostrya virginiana* and used for steaming stiff joints in rheumatism [Ojibwa: 47]. |
| *Lycopodium* sp.  Lycopodiaceae, Herb | club-moss, ground pine (Eng.); lycopode (Fr.); pâshitshinâkuana (Montagnais) | **Whole plant:** Tea for inducing labor and making childbirth easier, also used in treatments of bladder trouble in children [Algonquin: 69]. Weak runnings as antipyretics [Algonquians: 63; Mi'kmaq: 60]. Steeped in hot water with seal oil or lard, along with balsam fir and used as a compress against diarrhoea for children [Montagnais: 71]. |
| Lysichiton americanus Hultén & H. St. John  **Syn.:** *L. camtschatcensis* auct. non (L.) Schott  Araceae, Herb | yellow skunk cabbage (Eng.); lysichiton d'Amérique (Fr.) | Used as a general medicine [Malecite: 59, Mi'kmaq: 62]. Small piece steeped in a cupful of water and taken in diabetes [Mi'kmaq: 62]. |
| *Maianthemum canadense* Desf.  Liliaceae, Herb | wild lily-of-the-valley, squirrel berry (Eng.); maïanthème du Canada, petit muguet (Fr.); sōsōwīpukōsak, sōskōpukwagōh (Cree); agongosî'mînûn (Ojibwa) | Used to "keep the kidneys open" during pregnancy, to cure sore throat and headache [Ojibwa: 87]. Tea drunk for headache [Algonquians: 63].  **Leaves:** Cleaned and applied directly on a cut as a bandage or a cooled decoction is used to soak a cut. Poultice used to treat swellings of the limbs [Cree: 13]. |
| *Maianthemum* *racemosum* (L.) Link ssp. *racemosum*  **Syn.:** *Smilacina* *racemosa* (L.) Desf.; *Vagnera* *racemosa* (L.) Morong  Liliaceae, Herb | false spikenard, false Solomon's seal (Eng.); smilacine à grappes (Fr.); amwiminigak' (Maletice); onkôk'akak, onkôk'skak (Abenaki); kinē'wigwŏshk, agongo'simînûn', cigona'gan (Ojibwa) | Used in bleeding from the mouth [Abenaki: 67].Tea for sore back [Algonquin: 69].  **Roots:** Powdered, placed on a hot stone and fomentation applied to painful body parts or fume inhaled to cure headache or catarrh [Chippewa: 85; Ojibwa: 66, 84]. Decoction taken to treat back pain and other diseases in women [Ojibwa: 47]. Mixed with *Apocynum androsaemifolium* and used to "keep the kidneys open during pregnancy", to cure sore throat and headache [Ojibwa: 87].  **Leaves:** Warm decoction used as a bath to ease rash or itch [Maletice: 65; Ojibwa: 84]. Crushed and applied to cuts to stop bleeding [Ojibwa: 84]. |
| *Maianthemum* *stellatum* (L.) Link  **Syn.:** *Smilacina* *stellata* (L.) Desf.  Liliaceae, Herb | star-flowered solomon's seal (Eng.); smilacine étoilée (Fr.); anungokauh (Ojibwa) | Plants used as a stimulant and system cleanser [Ojibwa: 19]. |
| *Malaxis* *unifolia* Michx.  **Syn.:** *Microstylis* *unifolia* (Michx.) BSP  Orchidaceae, Herb | green Adder's mouth (Eng.); malaxis unifolié (Fr.); aîa'nîkotci'mîn (Ojibwa) | **Roots:** Used with *Diervilla lonicera* as a diuretic [Ojibwa: 87]. |
| **Matricaria discoidea* DC.**Syn.:** *Matricaria* *matricarioides* auct. non (Less.) Porter  Asteraceae, Herb | pineapple weed, chamomile (Eng.), matricaire odorante, herbe à crapaud (Fr.) | Used for kidney problems [Cree: 93]. Tea drunk to treat insomnia or to calm the nerves, can be sweetened with honey and given in a bottle to calm a fretting baby [Metis: 13]. Tea drunk as a relaxant, also given to new mothers to induce milk flow [Dene: 100].  **Flowers:** Used to treat eye infections by squeezing the fresh juice directly into the eye [Cree: 13]. |
| *Matteuccia struthiopteris* (L.) Todaro.  Dryopteridaceae, Herb | fiddlehead fern, ostrich fern (Eng.); matteucie fougère-à-l'autruche (Fr.); kākākīwīkot (Cree) | **Fronds:** Base of the frond boiled with other herbs to make a decoction drunk to slow a pounding heart, also used to treat stomach pain [Dene: 13]. Decoction made with the base of the stipe of the sterile frond used for back pain and to speed expulsion ot the afterbirth [Cree: 95].  **Rhizomes:** Stipe buds on rhizome used to treat cancer and to help gain weight [Metis: 13]. |
| **Medicago sativa* L.  Fabaceae, Herb | alfalfa, lucerne (Eng.); luzerne, lentine (Fr.) | **Above-ground parts:** Steeped to make a drink for arthritis or ulcers [Cree: 13]. Tea used in stomach problems, arthritis, and muscular pain [Cree: 96]. |
| *Melampyrum* *lineare* Desr.  Scrophulariaceae, Herb | narrow-leaved cow-wheat (Eng.); mélampyre linéaire (Fr.); agongasi'mînûk (Ojibwa) | **Whole plant:** Tea taken as an eye medicine [Ojibwa: 87]. |
| *Melilotus officinalis (L.) Lam.  **Syn.:** *M. albus* Medik.  Fabaceae, Herb | yellow sweet-clover (Eng.); mélilot jaune, mélilot officinal, trèfle d'odeur jaune (Fr.) | **Whole plant:** Boiled with barley seeds to make a decoction drunk as a general tonic [Cree: 13]. Tea used as a tonic and blood cleanser [Cree: 96]. |
| *Menispermum canadense* L.  Menispermaceae, Vine | Canada moonseed (Eng.); ménisperme du Canada (Fr.); bîma'kwît wa'bîgons (Ojibwa) | **Rhizomes:** Used for scrofula [44]. |
| **Mentha* *aquatica* L.  **Syn.:** *M. piperita* L.  Lamiaceae, Herb | peppermint (Eng.); menthe poivrée (Fr.) | Used for sore throat and for kidney troubles in combination with beaver castor [Algonquin: 75].  **Leaves:** Steeped and used for headache [Algonquians: 63]. |
| *Mentha arvensis* L.  **Syn.:** *M. canadensis* L.  Lamiaceae, Herb | wild mint (Eng.); mente sauvage, baume (Fr.); amisko wehkuskwa, amiskōwīkask, āmskuwiy(h)kusk, wīkask, wīkaskwah, wakaskwah (Cree); pse'skil (Melatice); name'wûckons (Ojibwa); kaakawakamicik (Atikamekw); kâuîpâpinamânapukâshiti nîpîsha (Montagnais); tsátlh'oghtsëné (Chipewyan) | Used as a carminative [Chippewa: 85] or to treat stomachache [Cree: 42, Ojibwa: 86]. Steeped and given at frequent intervals to make a child quieter [Malecite: 65]. Tea drunk to favor sleep [Cree, Metis: 13 ], as wash for sores, as a part of compound medicines for treating cancer or diabetes [Metis: 13], to treat cough or cold [Cree, Dene: 13; Dene: 17], fever [Atikamekw: 73; Montagnais: 71; Cree: 13], congestion, chills, menstrual cramps, to soothe teething babies' gums or as a tonic, to treat weariness or fatigue, to treat children diarrhoea, or pain [Cree: 13]. Smelled or boiled and the steam inhaled to clear the nasal passages in cold [Cree: 13]. Tea used to prevent the onset of cold or to cure prolonged cold and coughing up blood [Cree: 95].  **Whole plant:** Tea taken as a blood remedy and against fever [Ojibwa: 87]. Tea used to treat stomach disorders and fever [Cree: 96].  **Above-ground parts:** Tea used to treat arthritis, muscular pain, sore throats, cough and cold. Steam inhaled for sore throat, cough and cold. Tea used as "women's medicine" [Cree: 96]. Tea used to treat cough [Chipewyan: 92]. Herbal water used for fever and high blood pressure [Cree: 93].  **Flowers:** Ground with yarrow flowers, placed in a cloth, moistened and rubbed on infected gums to remove pus. Crushed with flowers of *Achillea* *millefolium* var. *occidentalis*, wrapped in a cloth and dipped in water to clean pus from the gums of a teething child [Cree: 95].  **Leaves or leafy stem:** Ground and applied to the gums to relieve toothache [Cree: 95].  **Leafy stem and flowers:** Inserted into the nostril to stop nosebleed [Cree: 95]. |
| **Mentha* *spicata* L.  **Syn.:** *M. viridis* L.  Lamiaceae, Herb | spearmint (Eng.); menthe à épis, baume (Fr.); wekemakowemina (Cree) | **Leaves:** Infusion given in colic [Cree: 74]. |
| *Menyanthes* *trifoliata* L.  Menyanthaceae, Herb | buckbean (Eng.); ményanthe trifolié, herbe à canards (Fr.) | **Roots:** Very strong decoction drunk as a medicine [Algonquians: 63]. |
| *Mertensia* *paniculata* (Aiton) G. Don  Boraginaceae, Herb | lungwort, tall bluebells (Eng.); mertensie paniculée (Fr.); ogu-malask (Cree) | Part of a compound medicine used to treat heart trouble [Metis: 13]. |
| *Mirabilis* *nyctaginea* (Michx.) MacMill.  **Syn.:** *Oxybaphus* *nyctagineus* (Michx.) Sweet; Allionia nyctaginea Michx.  Nyctaginaceae, Herb | heart-leaved four-o'clock, heart-leaved umbrellawort (Eng.); nyctage parasol (Fr.); goko'coadji'bîk, be'dukadak'igisĭn (Ojibwa) | **Roots:** Poultice made with a decoction of dried roots or with fresh roots used in sprain or strained muscles [Ojibwa: 47].Used to reduce sprains and swellings [Ojibwa: 87]. |
| *Mitchella repens* L.  Rubiaceae, Herb | two-eyed Berry, creeper partridge-berry (Eng.); mitchella rampant, pain de perdrix (Fr.); papatigwibagassiwil (Abenaki) | **Fruits:** Used to treat swellings [Abenaki: 67]. Cooked into a jelly and used for fever [Algonquians: 63]. |
| *Mitella nuda* L.  Saxifragaceae, Herb | bishop's cap, naked mitrewort (Eng.); mitrelle nue (Fr.); amiskōcawakayipak, ōcawakāyipak (Cree) | **Leaves:** Crushed and wrapped in cloth and inserted in the ear to treat earache [Cree: 95]. |
| *Monarda fistulosa* L.  Lamiaceae, Herb | wild bergamot, horse mint (Eng.); monarde fistuleuse, menthe de cheval (Fr.); mostoswīkask, kapiskotānāskīhk (Cree); moshkōs'wa^n^owi^n^s', weca'wûs wackwî'nek, bibi'gwûnûkûk'  wabino'wuck (Ojibwa) | Tops dried and used as a sternutatory for cold relief, or boiled with *Eupatorium perfoliatum* to make a fomentation to be applied for rheumatism [Chippewa: 85].  **Whole plant:** Dried, boiled to obtain the volatile oil and inhaled to cure catarrh and bronchial affections [Ojibwa: 87]. Boiled and the decoction drunk by women after childbirth. Mixed with other plants to make a drink for treating menstrual cramps, stomachache, headache and fever [Cree: 13]. Tea used to treat fever, headache, and also used as "women's medicine" [Cree: 96].  **Above-ground parts:** Tea used to treat stomach disorders [Cree: 96].  **Roots:** Decoction used to ease stomach and intestinal pain [Ojibwa: 84].  **Roots and flowers:** Decoction used against intestinal worms [Ojibwa: 47].  **Flowers and leaves:** Steeped and used in skin eruptions and burns [Ojibwa: 47].  **Leaves:** Chewed and placed in the nostrils to relieve headache [Chippewa: 85]. |
| *Monarda punctata* L.  Lamiaceae, Herb | spotted beebalm (Eng.); monarde ponctuée (Fr.); ka-bi-sani-gwe-iag (Ojibwa) | Used as rubbing and smelling medicine, and as a remedy for stomach trouble [Ojibwa: 86]. |
| *Moneses* *uniflora* (L.) A. Gray  Pyrolaceae, Herb | single delight, one-flowered wintergreen (Eng.); monésès uniflore (Fr.) | Steeped to make a medicine for paralysis [Algonquians: 63]. |
| *Monotropa uniflora* L.  Monotropaceae, Herb | Indian pipe (Eng.); monotrope uniflore (Fr.); mīpitahmaskīhkīh (Cree) | Juice mixed with water and applied to sore eyes [44].  **Flower:** Chewed to relieve toothache [Cree: 95]. |
| *Morella* *pensylvanica* (Mirb.) Kartesz  Myricaceae, Tree | northern bayberry (Eng.); myrique de Pennsylvanie (Fr.) | **Roots:** Powdered, steeped in water, and applied for arthritis and rheumatism, or to treat kidney problems [Mi'kmaq: 62].  **Leaves:** Steeped in water and the liquid used as a mouth wash for sore mouth [Mi'kmaq: 62]. |
| *Myrica gale* L.  Myricaceae, Shrub | sweet gale (Eng.); myrique baumier (Fr.); tehgoo (Dene) | Used for sore eyes. Boiled and tea applied to skin rash and sores, or drunk to cure mouth infections and sore throat [Dene: 101].  **Stem:** Boiled with leaves and fruits and taken to treat tuberculosis [Dene: 98]. |
| *Myriosclerotinia caricis-ampullaceae* (Nyberg) Buch.  **Syn.:** *Sclerotinia caricis-ampullaceae* Nyberg  [Sclerotiniaceae](http://www.eol.org/pages/5643), Fungi | mwākōkot (Cree) | **Sclerotium:** Powdered and taken in water to facilitate labor during childbirth, also used in menstrual irregularity [Cree: 95]. |
| **Nepeta cataria* L.  Lamiaceae, Herb | Catnip (Eng.); népéta cataire, herbe à chats (Fr.); gajugĕns'ibûg, tci'name'wûck (Ojibwa) | **Leaves:** Alone or in combination with *Pycnanthemum virginianum* in a decoction given in fever. Steeped with an equal amount of *Tanacetum vulgare* and given in fever [Ojibwa: 47]. Tea as a blood purifier [Ojibwa: 87]. |
| **Nicotiana tabacum* L.  Solanaceae, Shrub | tobacco (Eng.); tabac (Fr.) | **Leaves:** Topical coagulant [Mi'kmaq: 61]. Used for tootache [Cree: 81]. Smoke blown into the ear to cure earache [Algonquians: 63, Mi'kmaq: 60, Cree: 81] or into the nostrils for faints [Cree: 81]. |
| *Nuphar lutea* (L.) Sm  **Syn.:** *N.variegata* Durand  Nymphaeaceae, Herb | variegated pond-lily, cow lily, yellow pond lily (Eng.); nénuphar à fleurs panachées, grand nénuphar jaune, pied-de-cheval (Fr.); pwakumosikum, oskotamo, waskātamow, wāskātamo, waskutamo, ōskītīpak, waskītīpak (Cree); uckicteabu (Atikamekw); egh'ke (Dene); teghaizé (Chipewyan) | **Rhizomes:** Powdered, used in a compound remedy to treat various ailments [Cree: 95]. Cut into thin slices, dried and chewed or made into a tea drunk to treat arthritis or used to bathe affected joints [Dene: 13; Chipewyan: 92] or to treat swellings [Cree: 13]. Sliced, grated and used as a poultice on boils [Cree, Dene: 13; Chipewyan: 92], diabetic skin ulcers [Metis: 13], infected wounds [Cree, Dene, Metis: 13], or mixed with other plants and used as a poultice to treat sore back or legs, foot pain [Cree: 13], sore joints, arthritis, or headache [Metis: 13]. Used in compound decoctions as a cough medicine, to facilitate childbirth [Dene: 13], to help recovery after childbirth [Cree: 13], or as a heart medicine [Cree, Dene: 13]. Poultice used in swellings and infections [Algonquin: 69]. Ground with *Acorus calamus* and *Heracleum lanatum*, moistened with water or grease and used as a poultice in headache, sore joints, swellings, and painful limbs. Fresh or rehydrated dried slices applied to infected skin [Cree: 95]. Soaked or boiled in water and used as a wash to treat swellings, infections and bee stings [Dene: 17]. Poultice used to treat facial paralysis, skin disorders, bee stings, cuts, burns, arthritis, and muscular pain. Tea used in heart problems and as a "women's medicine" [Cree: 96]. Boiled with stems of *Sorbus scopulina* and leaves of *Sarracenia purpurea* to make a cough remedy [Chipewyan: 92].  **Roots:** Steeped or worn around the neck as a general medicine [Mi'kmaq: 62]. Used to treat cold and sore back [Dene: 100].  **Whole plant:** Used in diabetes [Cree: 77].  **Stems:** Leafy stems sucked to prevent thirst [Atikamekw: 73]. Tea drunk to cure stomach pain, shortness of breath, heart problems, and urinary tract problems [Dene: 101]. |
| *Nuphar* *lutea* (L.) Sm. ssp. *advena* (Aiton) Kartesz & Gandhi  **Syn.:** *N. advena* (Aiton) W.T. Aiton  Nymphaeaceae, Herb | cow lily (Eng.); nénuphar à feuilles émergentes (Fr.) | Pounded into a pulp used in a poultice to heal swollen limbs [Mi'kmaq: 62].  **Leaves:** Mashed and used in swellings [Mi'kmaq: 60; Algonquians: 63].  **Roots:** Bruised and used in swellings and bruises [Algonquians: 63].  **Rhizomes:** Used as an astringent [Chippewa: 47]. |
| *Nuphar* *lutea* (L.) Sm. ssp. *polysepala* (Engelm.) E.O. Beal  **Syn.:** *N. polysepala* (Engelm.) Greene, *N. advena* Aiton  Nymphaeaceae, Herb | rocky mountain pond-lily, yellow water lily (Eng.); nénuphar à sépales nombreux (Fr.); oga'da mûn (Ojibwa); egh'ke (Dene) | **Roots:** Grated to make a poultice for sores, or dried, powdered and used to heal cuts and swellings [Ojibwa: 87].  **Stems:** Tea drunk to cure sore stomach, shortness of breath, heart problems and urinary tract problems [Dene: 101]. |
| *Nuphar* sp.  Nymphaeaceae, Herb | nénuphar (Fr.); ^m^ska'ta^wé^ (Abenaki) | Used to allay sexual irritability and in spermatorrhea [Abenaki: 67]. |
| *Nymphaea* *odorata* Aiton  **Syn.:** *Castalia* *odorata* (Aiton) Alph. Wood  Nymphaeaceae, Herb | American white water lily, sweet scented water lily (Eng.); nymphéa odorant, nénuphar blanc, lis d'eau (Fr.); odîte'abûg wa'bîgwûn (Ojibwa) | Plant pounded into a pulp and used as a poultice to heal swollen limbs [Mi'kmaq: 62]. **Leaves:** Paste used for swellings [Mi'kmaq: 60; Algonquians: 63].  **Roots:** Juice drunk in coughs. Boiled and used as a poultice for swellings [Algonquians: 63]. Used as a cough medicine or to treat tuberculosis [Ojibwa: 87]. Powdered root put in the mouth to treat sore mouth [Ojibwa: 47].  **Rhizomes:** Decoction used for leucorrhoea. Poultice used for suppurating glands [44]. |
| *Oclemena* *nemoralis* (Aiton) Greene  **Syn.:** *Aster* *nemoralis* Aiton  Asteraceae, Herb | bog aster (Eng.); aster des bois (Fr.); winĭsĭkĕns (Ojibwa) | **Roots:** Decoction drops used in ears to treat soreness [Ojibwa: 47]. |
| *Oenothera biennis* L.  Onagraceae, Herb | mink plant (Eng.); onagre (Fr.) | **Whole plant:** Soaked in warm water to make a poultice to heal bruises [Ojibwa: 87].  **Bark:** Strong infusion drunk in small doses for pain in the bowels [Montagnais: 70]. |
| *Oligoneuron* *rigidum* (L.) Small var. *rigidum*  **Syn:** *Solidago* *rigida* L.  Asteraceae, Herb | goldenrod (Eng.) | Used as an astringent and styptic [Chippewa: 47].  **Roots:** Decoction used to treat stoppage of urine [Chippewa: 47]. |
| *Onoclea* *sensibilis* L.  Dryopteridaceae, Herb | sensitive fern (Eng.); onoclée sensible (Fr.); a'nana'ganûck (Ojibwa) | **Roots:** Powdered and used to make a tea given to stimulate milk flow [Ojibwa: 87]. |
| *Oplopanax* *horridus* (Sm.) Miq.  **Syn.:** *Echinopanax* *horridus* (Sm.) Decne. & Planch. ex Harms  Araliaceae, Shrub | devil's club (Eng.); bois piquant (Fr.) | Used for colds and tuberculosis [Dene: 17].  **Roots:** Mixed with other plants in a decoction used as a diuretic, to treat diabetes, or to prevent birth [Cree, Metis: 13]. Boiled and taken as a general medicine. Used to wash sores [Dene: 17]. |
| *Orthilia* *secunda* (L.) House **Syn.**: *Pyrola* *secunda* L.  Pyrolaceae, Herb | one-sided wintergreen (Eng.); pyrole unilatérale (Fr.); sasdzaghé (Chipewyan) | **Leaves:** Mashed with lard and put on a cut to stop bleeding and help healing. Chewed to relieve toothache [Chipewyan: 92]. |
| *Osmorhiza* *claytonii* (Michx.) C.B. Clarke  Apiaceae, Herb | hairy sweet cicely (Eng.); osmorhize de Clayton (Fr.); osaga'tigom' (Ojibwa) | **Roots:** Dried, pounded and moistened in warm water and applied to skin ulcers. Dried and chewed or a decoction used to gargle to treat sore throat [Ojibwa: 47]. |
| Osmorhiza longistylis (Torr.) DC.  Apiaceae, Herb | long-styled sweet cicely (Eng.); osmorhize à long style (Fr.); segedebwens, osaga'tîkûm (Ojibwa) | **Roots:** Infusion taken for amenorrhea. Decoction used to wash nostrils of a hunting dog whit dulled sense of scent. [Ojibwa: 85]. Tea drunk for sore throat or to facilitate parturition [Ojibwa: 87]. |
| *Ostrya* *virginiana* (Mill.) K. Koch  Betulaceae, Tree | hophornbeam (Eng.); ostryer de virginie, bois de fer (Fr.); ma'nanons' (Ojibwa) | **Wood:** Decoction used for rheumatic pain or tuberculosis [Ojibwa: 91]. Decoction boiled with *Thuja occidentalis* and used as a cough syrup [Ojibwa: 47, 91]. Wood from the heart of branches boiled and decoction taken in kidney troubles. Mixed with *Lycopodium* *obscurum* and twigs of *Picea glauca* in a decoction used for steaming stiff joints in rheumatism [Ojibwa: 47]. |
| *Oxalis* *montana* Raf.  Oxalidaceae*,* Herb | wood sorrel (Eng.); oxalide de montagne (Fr.) | Aphrodisiac [55]. |
| *Packera* *aurea* (L.) A. Löve & D. Löve  **Syn.:** *Senecio* *aureus* L.  Asteraceae, Herb | golden ragwort, golden senecio (Eng.); séneçon doré (Fr.) | Used as a remedy for hemorrhages, bruises and in case of abortion [66]. |
| *Panax* *quinquefolius* L.  Araliaceae, Herb | ginseng (Eng., Fr.); ginseng à cinq folioles (Fr.); | **Roots:** Steeped and taken time to time to increase fertility in women [Algonquians: 63]. |
| *Panax trifolius* L.  **Syn.:** *Aralia trifolia* Gray  Araliaceae, Herb | dwarf ginseng (Eng.); ginseng à trois folioles, petit ginseng (Fr.); nesō'wakŏk (Ojibwa) | **Roots:** Chewed and applied to cuts [Ojibwa: 84]. |
| *Parmelia sulcata* Taylor.  Parmeliaceae, Lichen | shield lichen (Eng.) | Rubbed on the gums of teething babies to relieve discomfort [Metis: 13]. |
| **Pastinaca* *sativa* L.  Apiaceae, Herb | wild parsnip (Eng.); panais (Fr.); pigwe'wûnûsk (Ojibwa) | Very small quantity mixed with four other kinds of roots to make a tea for "female troubles" [Ojibwa: 87]. |
| *Pedicularis canadensis* L.  Scrophulariaceae, Herb | wood beteny, Canada pedicularis (Eng.); pédiculaire du Canada (Fr.); mandamî'nîodji'bîkîns (Ojibwa) | **Roots:** Finely cut and used as an aphrodisiac [Ojibwa: 87]. Used as a remedy for anemia [Chippewa: 85]. |
| *Pedicularis* *lanata* Cham. & Schltdl.  Scrophulariaceae, Herb | woolly lousewort (Eng.); pédiculaire laineuse (Fr.) | **Roots:** Mixed with tobacco and smoked to relieve headache [Dene: 98]. |
| *Penstemon* *confertus* Douglas ex Lindl.  Scrophulariaceae, Herb | lesser yellow beardtongue (Eng.); penstémon à fleurs jaunes (Fr.) | Used as a cough medicine [Chippewa: 47].  **Roots:** Decoction drunk as a purgative [Chippewa: 47].  **Outer bark:** Decoction used as a remedy for stomach troubles [Chippewa: 47]. |
| *Petasites* *frigidus* (L.) Fr. var. *palmatus* (Aiton) Cronquist **Syn.** : *P. palmatus* (Aiton) A. Gray  Asteraceae, Herb | palmate coltsfoot (Eng.); pétasite palmé (Fr.) | Steam taken to treat asthma, congested chest or cold [Dene: 100]. |
| *Petasites* *frigidus* (L.) Fr. var. *sagittatus* (Banks ex Pursh) Cherniawsky  **Syn.:** *P. sagittatus* (Banks ex Pursh) A. Gray  Asteraceae, Herb | arrow-leaved coltsfoot (Eng.); pétasite sagitté (Fr.); piskehte puskwa, nigutinepia, mōsōtawakayipak, yuwskiy(h)tiypuk (Cree) | Cooked with *Pyrola asarifolia* and the mixture used as a wash to treat chickenpox [Dene: 17]. Steam taken to treat asthma, congested chest or cold [Dene: 100].  **Leaves:** Used as a poultice on skin sores and burns [Metis: 13]. Applied to worms eating the flesh and to itchy skin [Cree: 95]. Put all over the body to treat chickenpox. Poultice wrapped to treat sore knee [Dene: 17]. Wraped on affected area to heal sores and draw out infections [Cree: 93].  **Leaves and roots:** Boiled together and put on measles for healing, decoction drunk "to make babies" [Dene: 17]. |
| *Phryma leptostachya* L.  Verbenaceae, Herb | lopseed (Eng.); phryma à épis grêles (Fr.); waia'bishkĕno'kŏk (Ojibwa) | **Roots:** Decoction taken for rheumatic pain in the legs [Ojibwa: 84]. Chewed dry or a decoction used as a gargle to treat sore throat [Ojibwa: 47]. |
| *Physocarpus opulifolius* (L.) Maxim.  Rosaceae, Shrub | ninebark (Eng.); physocarpus à feuilles d'obier, bois à sept écorces (Fr.); miskwazi-wušk (Ojibwa) | **Roots:** Infusion used as an emetic [Chippewa: 85]. |
| *Phytolacca americana* L.  Phytolaccaceae, Herb | American pokeweed, pigeon berry (Eng.); phytolaque d'Amérique (Fr.) | **Leaves:** Chewed and applied to wounds to stop bleeding [Mi'kmaq: 43]. |
| Phytolacca americana L. var. americana **Syn.:** *P. decandra* L.  Phytolaccaceae, Herb | common pokeweed, poke berry (Eng.); phytolaque d'Amérique (Fr.) | **Roots:** Used as an emetic and in rheumatism [44]. |
| Picea glauca (Moench) Voss  **Syn.:** *P. canadensis* (Mill.) BSP.  Pinaceae, Tree | white spruce (Eng.); épinette blanche (Fr.); wapiskimnahik, eyinatik, minuhik, mīnahik, sī(h)ta (Cree); me-naig, gawa'ndag (Chippewa); mzazesso, mskask (Abenaki); minaik^u^ (Montagnais), ts'iivii, ts'iwa (Dene); ts'uchogh (Chipewyan) | **Twigs:** Tea used as a general medicine [Algonquians: 63]. Steeped to make an antiscorbutic tea [Mi'kmaq: 60]. Tips of branches used to brew a tea to "heal the inside". Tea used in sudatory by women after childbirth [Algonquin: 69]. Boiled and the vapour taken to fight fever and eye problems [Montagnais: 71], to relieve cold [Dene: 99] or to ease headache [Dene: 101]. Tea used for fever [Montagnais: 71]. Mixed with *Lycopodium* *obscurum* and inner part of *Ostrya virginiana* wood in a decoction used for steaming stiff joints in rheumatism [Ojibwa: 47]. Young twigs chewed to relieve itchy throat [Dene: 99]. Used to treat diabetes [Cree: 82]. Juice from young tree boughs used for sore eyes. Towels soaked in tea and wrapped around a woman's waist to speed up delivery, to ease afterbirth pain, and to loosen retained placenta [Dene: 101]. Herbal water taken for colds [Cree: 93].  **Inner bark:** Used as a poultice or boiled to make a wash for decayed teeth, skin sores, and burns [Dene: 17; Chipewyan: 92]. Chewed or drunk as a tea for cough [Algonquin: 69; Montagnais: 71]. Decoction used in a compound arthritis remedy [Cree: 95]. Used in diabetes [Cree: 83]. Chewed to relieve cold or maintain good health, or placed on a wound [Dene: 99]. Used to stop bleeding from cuts [Cree: 81]. Used to treat diabetes [Cree: 82]. Mixed with caribou grease to make a salve for burns [Chipewyan: 92].  **Bark:** Tea used as a salve [Mi'kmaq: 43]. Steeped and given in tuberculosis [Mi'kmaq: 62]. Used as a splint and to heal broken limbs [Dene: 100].  **Sap:** Used to soothe irritated skin, applied to cuts and mouth infections [Dene: 17, 99]. Applied to chest to prevent cold and tuberculosis. Tea taken to soothe sore throat [Dene: 99]. Applied to stop bleeding from cuts [Dene: 13; 99; Cree: 81]. Boiled and taken in cough. Heated and pressed on blisters [Cree: 80]. Herbal water taken for colds. Heated, mixed with lard, cooled and applied to wound infections [Cree: 93].  **Bark and branches:** Mixed with red willow bark to treat cold [Dene: 17].  **Gum:** Used alone or most commonly mixed with rendered fat from a bear, otter, or beaver, or lard or petroleum jelly, and applied as a salve for skin infections, cuts, rashes, burns, persistent sores, and chapped or craked skin [Cree, Dene, Metis: 13; Algonquin: 69]. Chewed and the juice swallowed to treat a sore throat [Metis: 13]. Mixed with grease to make an ointment to treat skin rashes, scabies, persistent scabs and a growing boil [Cree: 95]. Chewed as a laxative [Algonquin: 69]. Applied to throat against cough or on back for pain relief [Montagnais: 71]. Used to treat diabetes related symptoms [Cree: 82]. Applied in cuts and skin infections [Cree: 80, 81]. Used to treat gum disease, cuts, skin rashes, tea drunk for stomach ailments [Dene: 100]. Smeared on painful areas of the body, boiled and gargled to cure toothaches, used as an ointment for sores and placed on a wound to stop bleeding, boiled and tea drunk for colds and coughing [Dene: 101]. Warm and applied to cuts, boils or sores [Cree: 79]. Poultice used to treat skin disorders, bee stings, cuts and burns [Cree: 96]. Warmed up and put in infections [Dene: 13]. Used to treat sore throat [Dene: 100].  **Rotten wood:** Powdered and used as baby powder or to treat skin rashes [Cree: 94, 95].  **Cones:** Used to make a medicine for excessive urination [Abenaki: 67]. Used to make a jelly to treat headache and stomachache [Algonquin: 75]. Boiled and water used to wash itchy skin, or drunk against intestinal problems [Montagnais: 71]. Used to treat diabetes related symptoms [Cree: 82].  **Leaves:** Used as an inhalant or fumigator [Ojibwa: 87]. Used to treat diabetes related symptoms [Cree: 82]. Powder used as a compress for aches and pains [Cree: 80, 81]. Cushed, placed on cloth and put in throat to treat pain and burns [Cree: 80].  **Roots and bark:** Tea used for stomach pain, fainting and fits [Ojibwa: 86].  **Gum and bark:** Used for cough [Montagnais: 71].  **Cones and branches:** Tea used for cough and colds, sore throat and chest pain [Dene: 99].  **Roots:** Pounded and boiled to make a liquid medicine [Dene: 99].  **Young tips, cones and branches:** Boiled and taken to treat itchy throat [Dene: 99]. Steam used to treat congested head and chest [Dene: 100].  **Wood:** Inside strips put on burns, boiled without bark and taken for sore throat [Cree: 79]. |
| *Picea* *mariana* (Mill.) BSP.  Pinaceae, Tree | black spruce (Eng.); épinette noire (Fr.); ithināhtik, minahik, setakwunatik, pekewatik, mīthawapakōnuk, mistikōpikī (Cree); kaawaataak (Ojibway); Say-say-gah-dag , jingwûp (Chippewa); a'mikwan'dŏk (Ojibwa); ushkâtuk^u^ (Montagnais); ts'iivii, ędzò (Dene); el (Chipewyan) | Used for a sudatory after childbirth [Algonquin: 69].  **Twigs:** Boiled to make a broth for cough [Algonquin: 63] or to produce steam that relieves cold [Dene: 99]. Tips or buds peeled and eaten to prevent breathing shortness, heart problems, and high blood pressure [Cree: 13]. Decoction used to treat sore eyes [Dene: 101; Dene, Metis: 13]. Tips used to make a tea for "healing the inside" [Algonquin: 69]. Tea used against fever [Montagnais: 71]. Yound twigs chewed to relieve itchy throat [Dene: 99]. Used to treat diabetes [Cree: 82]. Boiled and steamed to cure congestion or cold and to ease headache. Towels soaked in tea and wrapped around a woman's waist to speed up delivery, to ease afterbirth pain, and to loosen retained placenta [Dene: 101].  **Gum:** Applied as a salve for skin infections, cuts, boils, rashes, burns, sores, and chapped or craked skin [Dene: 13, 100; Cree: 13, 79, 80; Algonquin: 69]. Mixed with grease to make an ointment to treat skin rashes, scabies, persistent scabs and bad burns [Cree: 95]. Chewed to prevent breathing shortness, also good for the heart [Cree: 13]. Soaked in hot water and used to treat infected cuts and wounds [Cree: 13]. Chewed and applied to a sore ear or to treat stomach ache [Cree: 13]. Boiled until it turns pink and then cooled to make an ointment to draw the infection out of cysts [Cree: 13] or to treat infected wounds, rashes, scabs on the head or chiken pox [Cree: 13]. Used as a laxative [Algonquin: 69]. Used for cuts and back pain [Montagnais: 71]. Used to treat diabetes [Cree: 82]. Used to treat gum disease, tea drunk for stomach ailments [Dene: 100]. Smeared on painful areas of the body, boiled and gargled to cure toothache, used as an ointment for sores and placed on a wound to stop bleeding. Boiled and tea drunk for cough and cold [Dene: 101]. Used to treat sore throat [Dene: 100].  **Sap:** Blood medicine, used to soothe irritated skin, applied to cuts and mouth infections [Dene: 13; 99]. Applied in chest to prevent colds and tuberculosis, tea taken to soothe sore throats [Dene: 99]. Applied in cuts and sore breasts [Cree: 81].  **Bark and branches:** Mixed with red willow bark to treat cold [Dene: 13].  **Wood:** Powdered and used as baby powder [Dene: 13]. Inside strips put on burns, or boiled without bark and taken for sore throats [Cree: 79].  **Charcoal:** Used as a baby powder [Dene: 13].  **Cones:** Young cones boiled to make a mouth wash for mouth infection, toothache or sore throat or to clear phlegm from the throat [Dene, Metis: 13; Chipewyan: 92]. Chewed to relieve toothache or sore mouth. Mixed with other plants in a decoction to treat venereal disease [Cree: 95]. Made into a jelly used to treat headache and stomachache [Algonquin: 75]. Decoction used to gargle a sore throat or mouth infection [Cree: 95; Dene: 101]. Used to treat diabetes [Cree: 77; 83], diarrhoea [Ojibway: 89; Cree: 95] and stomach ache [Montagnais: 71]. Applied to stop bleeding. Boiled and tea used to wash broken legs, to easeskin rash [Dene: 101].  **Leaves and bark:** Decoction used for headache [Ojibwa: 66].  **Leaves:** Used as a reviver [Ojibwa: 87] or to treat diabetes [Cree: 82]. Crushed and used to treat pain. Crushed, placed on cloth and put in throat to treat pain and burns [Cree: 80].  **Bark and roots:** Tea used for stomach pain, fainting and fits [Ojibwa: 86].  **Inner bark:** Chewed for laryngitis [Mi'kmaq: 62]. Chewed to relieve colds or maintain good health, placed on a wound [Dene: 99]. Used to treat diabetes [Cree: 82].  **Bark and gum:** Used against cough [Montagnais: 71].  **Cones and branches:** Tea used for cough and colds, sore throat and chest pain [Dene: 99].  **Roots:** Pounded and boiled to make a liquid medicine [Dene: 99].  **Young tips, cones and branches:** Boiled and taken to treat itchy throat [Dene: 99]. Steam used to treat congested head and chest [Dene: 100].  **Bark:** Used as a splint and to heal broken limbs [Dene: 100]. Boiled and taken in cough Heated and pressed on blisters [Cree: 80]. |
| *Picea* *rubens* Sarg.  Pinaceae, Tree | red spruce (Eng.); épinette rouge (Fr.) | **Bark:** Boiled with roots of sour grass and the liquid drunk for lung and throat trouble [Algonquians: 63]. |
| *Picea* *sitchensis* (Bong.) Carrière  Pinaceae, Tree | Sitka spruce, tideland spruce (Eng.); épinette de Sitka (Fr.) | **Cones:** Boiled and decoction taken internally for pain in the chest [Sikani: 97].  **Inside bark:** Scraped and chewed for cough [Sikani: 97].  **Needles:** Chewed and saliva applied to external sores [Sikani: 97].  **Gum:** Applied with a stick to the white spots on the eye [Sikani: 97]. |
| *Picea* sp.  Pinaceae, Tree | spruce (Eng.); épinette (Fr.); cigubi (Atikamekw) sĕ'ssēgân'dŏk (Ojibwa) | **Gum:** Used as a poultice on wounds [Atikamekw: 73], boils and abscesses [Algonquians: 63].  **Roots:** Used for sewing [Ojibwa: 84; Atikamekw: 73]. |
| *Pinus banksiana* Lamb.  Pinaceae, Tree | jack pine (Eng.); pin gris (Fr.); ōskāhtak, ivashick (Cree); Ookiik, gîga'ndag (Ojibway); ushtshishk (Montagnais); goo (Dene) | **Inner bark:** Used to make a poultice to heal a deep cut [Cree: 95] or to treat diabetes [Cree: 82].  **Cones and inner bark:** Used in diabetes [Cree: 77, 83].  **Bark:** Mixed with bark and root of *Salix discolor, Pinus strobus, Quercus rubra* and *Arctostaphylos uva-ursi* in a tea given in fainting and fits [Ojibwa: 86]. Rubbed on a sore body part or boiled and tea drunk to cure shortness of breath [Dene: 101].  **Sap:** Used for burns [Ojibway: 89] and cuts [Dene: 17]. Chewed to treat cold [Cree: 93].  **Leaves:** Used as a reviver [Ojibwa: 87].  **Twigs:** Crushed and mixed with lard and applied for allergies and itching [Montagnais: 71]. Tea mixed with sugar and put into sore eyes. Tea drunk to ease pain, cold, and as a general remedy. Tea also applied to a sore body part [Dene: 101]. |
| *Pinus* *contorta* Douglas ex Louden  Pinaceae, Tree | lodgepole pine (Eng.); pin tordu (Fr.); ōskāhtak, oskatik (Cree) | **Inner bark:** Softened in water and used as a poultice to heal a deep wound [Metis: 13].  **Gum:** Chewed and saliva swallowed for cough and cold [Sikani: 97].  **Roots:** Boiled and liquid used to clean wounds [Dene: 98]. |
| *Pinus resinosa* Aiton  Pinaceae, Tree | red pine (Eng.); pin rouge (Fr.); pōkgwĕ'nagēmŏk, abakwanûg i'mûg (Ojibwa) | **Leaves:** Crushed and applied to relieve headache. Vapour or fumes from heating inhaled to cure headache [Ojibwa: 84]. Crushed with *Pinus strobus* leaves, boiled and applied to relieve headache [Ojibwa: 66]. |
| *Pinus* sp.  Pinaceae, Tree | pine (Eng.); pin (Fr.); cigawk (Atikamekw) | Inner part of the tree boiled and pulp applied to cuts [Cree: 80].  **Inner bark:** Steeped in boiling water and applied to the chest for cold [Atikamekw: 73]. |
| *Pinus strobus* L.  Pinaceae, Tree | white pine (Eng.); pin blanc (Fr.); zhingwâk' (Ojibwa); kah-be-sah-dah-ge-set, jîngwak kweseskwe'tûk (Chippewa); kohah'sis (Abenaki). | Tea used in kidney or urinary trouble [Mi'kmaq: 60].  **Bark, needles and twigs:** Tea used in cold and cough [Mi'kmaq: 43, 60].  **Bark:** Boiled, mixed with grease and applied to wounds. Tea used in kidney troubles [Mi'kmaq: 43]. Steeped and used to treat cough [Abenaki: 67] and cold [Mi'kmaq: 62]. Mixed with bark and root of *Salix discolor, Pinus strobus, Quercus rubra* and *Arctostaphylos uva-ursi* in a tea given in fainting and fits [Ojibwa: 86].  **Inner bark:** Boiled and given for sores, swellings [Algonquians: 63].  **Pitch:** Mixed with deer tallow and used as a poultice for felons and similar inflammations [Chippewa: 85].  **Needles:** Crushed and applied to relieve headache. Boiled, and the vapour inhaled to cure backache. Fume produced by heating inhaled to cure headache [Ojibwa: 84]. Crushed with *Pinus resinosa* needles, boiled and applied to relieve headache [Ojibwa: 66]. Powdered and used as a reviver or inhalant [Ojibwa: 87].  **Gum:** Boiled and drunk for sore throat, cold and consumption [Algonquians: 63].  **Wood:** Mixed with inner bark of *Prunus serotina* and *Prunus americana* in a decoction used to treat cuts and wounds [Ojibwa: 47]. |
| **Plantago major* L.  Plantaginaceae, Herb | common plantain, whiteman's foot (Eng.); plantain majeur, grand plantain queue de rat (Fr.); muchikwanas, paswēpak (Cree); ceca'gûski'bûge sink , gine'biwûck (Ojibwa); magaskisitan (Atikamekw); ondiba'go (Abenaki) | **Stem:** Base boiled and drunk to relieve fainting spells [Metis: 13].  **Leaves:** Chewed into a paste applied to burns and scalds. Juice drunk for toothache and earache or for internal hemorrhage or injury [Cree: 74]. Soaked in warm water and applied to bruises, sprains or sores as a poultice, also used to sooth burns, scalds, bee stings, and snake bites [Algonquin: 69; Ojibwa: 87]. Applied fresh on wounds, bruises, burns and haematoma [Atikamekw: 73]. Infusion drunk to treat heart trouble [Cree: 13, 96], cramps, stomachache, stomach flu, or constipation [Metis: 13]. Used for rheumatism, swellings and as an analgesic [Abenaki: 67]. Used as a poultice to remove poison and heal wounds [Mi'kmaq: 62]. Mixed with grease and applied to inflamed skin. Mixed with roots of *Asarum canadense* to treat inflamed skin. Decoction applied to rheumatic joints [Ojibwa: 47]. Poultice used as a painkiller for cuts and bruises [Dene: 100]. Fresh leaves heated slightly and put on the wound to draw out infection [Cree: 93].  **Leaves and root:** Chopped finely and applied to bites of poisonous reptiles, or applied as a poultice on inflamed skin [Ojibwa: 47].  **Above-ground parts:** Tea used to soothe burns [Dene: 100]. |
| *Platanthera* *dilatata* (Pursh) Lindl. ex Beck var. *dilatata*  Orchidaceae, Herb | tall white bog orchid, scentbottle (Eng.); platanthère dilatée (Fr.) | **Roots:** Juice drunk in gravel [Algonquians: 63]. |
| *Platanthera* *dilatata* (Pursh) Lindl. ex Beck var. *leucostachys* (Lindl.) Luer  **Syn.:** *Habenaria* *leucostachys* (Lindl.) S. Watson  Orchidaceae, Herb | Sierra bog orchid (Eng.); platanthère à tige pâle (Fr.) | Used as a cough medicine. Decoction used as a body wash. W/hen heated used as a bath for rheumatism [Ojibwa: 47]. |
| *Platanthera* *orbiculata* (Pursh) Lindl.  **Syn.:** *Habenaria* *orbiculata* (Pursh) Torr.  Orchidaceae, Herb | roundleaved orchis (Eng.); habénaire à feuilles orbiculaires (Fr.) | **Leaves:** Applied to the hands or feet to cure blisters [Algonquians: 63]. |
| *Poa palustris* L.  Poaceae, Herb | swamp meadow-grass, fowl bluegrass (Eng.); pâturin palustre (Fr.) | **Seed heads:** Boiled to make a rinse used to make the hair grow thicker and longer [Dene: 13]. |
| *Podophyllum peltatum* L.  Berberidaceae, Herb | mayapple (Eng.); podophylle pelté, pomme de mai (Fr.) | **Roots:** Used as cathartic [44]. Powdered and used for liver troubles [Cree: 74]. |
| *Polygala* *polygama* Walter  Polygalaceae*,* Herb | milkwort, pink polygala (Eng.); polygala polygame (Fr.) | Boiled and used as a cough medicine [Algonquians: 63]. |
| *Polygala senega* L.  Polygalaceae, Herb | seneca snakeroot (Eng.); sénéca (Fr.); menisehkes, mesisikas, ominisihkes, sīkōtākanīsīkan, wenisikas, wīnsīkas, wīncīkēs (Cree); winis'sikē^n^s', bi'jikiwûck' (Ojibwa) | Used as a tonic [Ojibwa: 47] or as a general medicine [Ojibwa: 86].  **Roots:** Powdered and added to other plants and used to treat many ailments [Cree: 95]. Chewed and the juice swallowed to treat a sore throat. Crushed and mixed with another plant to make a poultice applied to cuts [Cree: 13]. Mixed with other plants and used as a heart medicine and blood purifier [Metis: 13]. Roots crushed or chewed to treat toothache or sore mouth [Cree: 13, 95]. Dried and chewed for cold [Malecite: 65]. Decoction for cough and cold [Ojibwa: 84]. Mixed with *Artemisia frigida, Astragalus crassicarpus,* and *Rosa arkansana* in a decoction taken internally for convulsions or applied to wounds to stop bleeding [Ojibwa: 47]. Sucked and juice swallowed to treat tooth ache, sore throat, cough and cold. Poultice used to treat skin disorders, bee stings, cuts and burns [Cree: 96].  **Leaves:** Infusion taken for sore throat [Ojibwa: 84]. |
| *Polygonatum* *biflorum* (Walter) Elliot  Liliaceae, Herb | giant Solomon's seal (Eng.); sceau-de-Salomon géant (Fr.); nanîbîte'ode'kîn (Ojibwa) | **Roots:** Used as a physic. Tea used for cough [Ojibwa: 87]. |
| *Polygonatum* *biflorum* (Walter) Elliot var. *commutatum* (Schult. & Schult. f.) Morong  **Syn.:** P. commutatum (Schult. & Schult. f.) A. Dietr.  Liliaceae, Herb | giant Solomon's seal (Eng.); sceau-de-Salomon géant (Fr.); nebnegodek (Ojibwa) | **Roots:** Used to prevent measles [Ojibwa: 85]. Decoction sprinkled on hot stones and the smoke inhaled to treat hadache [Ojibwa: 47]. |
| *Polygonatum pubescens* (Willd.) Pursh.  Liliaceae, Herb | hairy Solomon's seal (Eng.); sceau-de-Salomon pubescent (Fr.); onkôk'skak (Abenaki) | Used to stop bleeding from mouth in women [Abenaki: 67]. |
| *Polygonum amphibium* L.  Polygonaceae, Herb | water smartweed, amphibious knot-weed (Eng.); renouée amphibie (Fr.); kāmithkwacōāhtik, kistōtīwīcīpihk (Cree) | **Roots:** Applied fresh on mouth blisters. Powdered with other plants to treat various ailments [Cree: 95]. |
| **Polygonum hydropiper*  L.  Polygonaceae, Herb | marshpepper knotweed, water pepper (Eng.); renouée poivre-d'eau (Fr.) | **Leaves:** Dried and used to make a tea for dropsy [Malecite: 65]. |
| *Polygonum muhlenbergii* (Meisn.) Wats.  Polygonaceae, Herb | long-root smartweed, swamp persicaria (Eng.); renouée émergée (Fr.); agongo'simînûn (Ojibwa) | Tea used to cure stomachache [Ojibwa: 87]. |
| *Polygonum pensylvanicum* L.  Polygonaceae, Herb | Pennsylvania smartweed (Eng.); renouée de Pennsylvanie (Fr.); pebigumskike (Ojibwa) | Tops steeped and the infusion taken as a cure for epilepsy [Ojibwa: 85]. |
| **Polygonum persicaria* L.  Polygonaceae, Herb | lady's thumb (Eng.); renouée persicaire (Fr.) | **Flowers and leaves:** Decoction taken in stomach pain [Ojibwa: 47]. |
| *Polygonum punctatum* Elliot  Polygonaceae, Herb | interrupted smartweed (Eng.); renouée ponctuée (Fr.); ojig'imĭn (Ojibwa) | **Flowers and leaves:** Decoction taken in stomach pain [Ojibwa: 47]. |
| *Polypodium* *virginianum* L.  **Syn.:** P. vulgare auct. non L. p.p.  Polypodiaceae, Herb | rock polypod, parasitic sword fern (Eng.); polypode de Virginie, tripe de roche (Fr.); tcetcgwi'zek' (Malecite); kākākīwīkoc, kāthīthīkipakākī, kinīpikōtīthanīwīpak (Cree); otatakwakanak (Atikamekw); Achiniu thistabi (Innu) | Used for stomach troubles [Abenaki: 67], or against kidney and neuralgic diseases [Atikamekw: 73]. Tea used to treat digestive tract problems [Algonquin: 68], heart diseases [Algonquin: 69], as well as lung, liver and kidney diseases [Innu: 72].  **Roots:** Steeped and given in pleurisy [Malecite: 65].  **Leaves:** Decoction taken as a tuberculosis cure [Cree: 95]. |
| *Polyporus resinosus* (Schrad.) Fr.  Polyporaceae, Fungi | fungi (Eng.); polypore (Fr.) | Conks boiled and used as a cough medicine [Dene: 98]. |
| *Polyporus* sp.  Polyporaceae, Fungi | polypore (Fr.) | Used for "female troubles" [Algonquians: 63]. |
| *Polystichum* *acrostichoides* (Michx.) Schott  Dryopteridaceae, Herb | christmas fern (Eng.); polystic faux-acrostic (Fr.) ktciutcetckuk (Malecite) | **Roots:** Chewed in hoarseness [Malecite: 65].  **Fronds:** Soaked in cold water and pounded to treat body pain [Algonquians: 63]. |
| *Pontederia cordata* L.  Pontederiaceae, Herb | pickerel weed, lake herb (Eng.); pontédérie cordée (Fr.); waladawi'ga (Malecite) | Brewed and taken in general illnesses [Algonquians: 63]. Steeped and used to prevent pregnancy [Malecite: 65]. |
| **Populus alba* L.  Salicaceae, Tree | white poplar (Eng.); peuplier blanc (Fr.); ah-sah-te (Ojibwa) | **Bark and roots:** Tea used for internal blood diseases [Ojibwa: 86]. |
| *Populus balsamifera* L.  Salicaceae, Tree | balsam poplar, black poplar (Eng.); peuplier baumier, peuplier noir, liard (Fr.); ma-saté, mah-nah-sah-te (Chippewa); metos, mayi metos, mayi-mītos, maymiytos, māthamītos, osīmisk (Cree); ewebibu'k (Malecite); man'asa'dĭ, asa'di (Ojibwa); t'oo, t'ooladzé (Dene) | Used for internal blood diseases [Ojibwa: 86].  **Buds:** Gathered unopened in early spring and boiled to extract the balsam, skimmed off and then boiled with bear fat to make a salve applied to frost-bitten members and on sores and inflamed wounds [Chippewa: 85]. Gathered in the spring and steeped in very hot bathwater until a layer of extract forms on the surface and the water is cool enough to bathe in, and also rubbed on afflicted areas to treat skin diseases such as eczema or psoriasis [Cree, Metis: 13]. Collected in the spring to make a tea taken for cold [Dene: 99]. Rubbed on the gums of a teething baby, or a cooled infusion used to rinse the baby's mouth [Dene: 13]. Rubbed on a sore tooth [Cree: 13]. Decoction drunk to treat heart problems [Cree: 13]. Boiled for 41 minutes with trembling aspen branch bark and the decoction given to treat diabetes [Cree: 13]. Gathered in the spring to make salve [Algonquin: 69]. Applied directly to the nostril to stop nosebleed [Cree: 95]. Smashed with roots of *Arctium lappa* and applied to sores [Malecite: 65]. Seeped before opening and used as a poultice in sprain or strained muscles, or boiled in grease, strained and kept for use when needed [Ojibwa: 47]. Cooked in lard or bear fat, cooled and used as a salve on cuts, wounds or bruises, also rubbed inside nostrils to clear congestion from cold, catarrh or bronchitis [Ojibwa: 87]. Steam used to treat nasal and chest congestion caused by cold [Dene: 100].  **Sap:** Drunk to treat diabetes and high blood pressure [Cree, Metis: 13]. Used on cuts [Dene: 99]. Boiled and tea drunk to ease chest pain Smeared on a painful body part [Dene: 101].  **Bark and sap:** Used to make a tea for asthma in children [Cree, Metis: 13].  **Bark:** Mixed with another plant in a decoction given in seizures [Cree: 13].  **Leaves:** Applied fresh to a sore [Cree: 95]. Chewed and applied to insect stings and bites [Dene: 100].  **Roots:** Scrapings steeped in hot water and used as a poultice on open sores and infected wounds [Algonquin: 69]. Decoction made with equal portion of roots of *Cirsium* sp. taken internally to treat weakness and back pain in women. Mixed with roots of *Populus tremuloides*, steeped and given to slow heavy menstrual flow [Ojibwa: 47].  **Inner bark:** Used in diabetes [Cree: 77]. Boiled and taken to treat stomach problems [Dene: 98].  **Catkins:** Boiled and rubbed on swollen areas. Tea drunk for breathing problems [Dene: 101].  **Rotten wood:** Powdered and used for skin rash [Dene: 101]. |
| *Populus* *deltoides* Bartram ex Marsh. ssp. *monilifera* (Aiton) Eckenwalder  **Syn.:** *P. monilifera* Aiton  Salicaceae, Tree | cottonwood (Eng.); peuplier deltoïde (Fr.); mâ'nâsâ'ti (Ojibwa) | "Cotton" applied to open sores as an absorbent [Ojibwa: 84]. |
| *Populus* *grandidentata* Michx.  Salicaceae, Tree | poplar (Eng.); peuplier à grandes dents (Fr.); mutoos (Cree); asadi (Ojibwa) | Used to prevent pregnancy [Cree: 76].  **Young roots:** Tea used as a hemostatic [Ojibwa: 87].  **Bark:** Infusion given to ease and lessen the menses [Cree: 76]. |
| *Populus* sp.  Salicaceae, Tree | poplar (Eng.); peuplier (Fr.) | Burned and ashes put on boils and abscesses [Cree: 80, 81].  **Bark:** Decoction used in cough [Cree: 42]. Dried, boiled and drunk for ringworm [Cree: 80].  **Inner bark:** Eaten in the spring, considered a mild purgative [Cree: 42]. Used in diabetes [Cree: 83].  **Dry wood:** Ash mixed with boiled water and taken for urinary infections. Also used as poultice [Cree: 79].  **Gum:** Drops put into eyes to treat snow blindness [Cree: 80]. |
| *Populus tremuloides* Michx.  Salicaceae, Tree | quaking aspen, trembling aspen (Eng.); peuplier faux-tremble, tremble (Fr.); mi'tuc, mitesh (Montagnais); asa'dĭ (Ojibwa); metos, miytos, wapisk-mītos, wasī-mītos, mistik, mītos, mitosinipiah (Cree); asawdeobuk (Atikamekw); os'sagakwé, wawabibagw (Abenaki); t'ooladzé (Dene); k'es (Chipewyan) | Tea used for rheumatism, cold and stomachache [Innu: 72].  **Sap:** Drunk for worms [Algonquin: 68; Montagnais: 70]. Boiled and drunk as a blood medicine [Dene: 17].  **Bark:** Used to treat venereal disease [Cree: 95]. Decoction or tea drunk as a vermifuge [Abenaki: 67; Algonquians: 63; Algonquin: 68; Montagnais: 70], to treat stomach disorders, or diabetes [Cree: 96]. Steeped and used for cold [Algonquians: 63; Mi'kmaq: 60]. Small squares placed under the tongue to treat a stomachache or against blood spitting [Cree: 13]. A strip about the length of the human heart cut at heart height on the south side of a mature tree is chewed and the juice swallowed as a heart medicine [Cree: 13]. Small strip of fresh green bark chewed and swallowed for relief from food poisoning or diarrhoea [Metis: 13]. Infusion drunk to treat cancer and diabetes [Cree: 13]. Decoction drunk to treat stomachache, diarrhoea [Cree: 13], fever and cough [Dene: 13]. Mixed with buds of *Populus balsamifera* in a decoction drunk to treat diabetes [Cree: 13]. White "dust" on the bark (crustose lichens and dead tree periderm) applied to cuts and wounds to stop bleeding [Dene: 13] or used as a styptic [Cree: 95]. Boiled and used in boils [Algonquin: 75]. Chewed and applied to cuts as a poultice [Chippewa: 47; Ojibwa: 87].  **Inner bark:** Used in diabetes [Cree: 77], as a laxative or cough remedy [Cree: 66].Boiled and used as a tonic [55]. Poultice used on sore arm or leg [Ojibwa: 87]. Chewed and juice swallowed to treat heart problems [Cree: 96]. Used to cover wounds to stop bleeding [Cree: 93].  **Leaves:** Chewed or crushed fresh and applied to bee or wasp stings [Cree: 95; Cree, Dene: 13; Chipewyan: 92], or on mosquito bites or cuts [Cree: 13]. Boiled and drunk as a medicine [Dene: 17].  **Buds:** Used for toothache [Cree: 13].  **Seeds:** Eaten to cause an abortion [Dene: 13; Chipewyan: 92].  **Roots:** Steeped with roots of *Populus balsamifera* and given to slow heavy menstrual flow [Chippewa: 47]. Grated and boiled into a sirup for rheumatisms and numb articulations [Atikamekw: 73].  **Rotten wood:** Powder used for skin rash [Dene: 101]. |
| *Potentilla* *anserina* L. **Syn.:** *Argentina* *anserina* (L.) Rydb.  Rosaceae, Herb | common silverweed (Eng.); potentille ansérine (Fr.) | **Whole plant:** Tea drunk as a coagulant, to heal cuts and sores [Dene: 100]. |
| *Potentilla arguta* Pursh  **Syn.:** Drymocallis arguta (Pursh) Rydb.  Rosaceae, Herb | tall cinquefoil (Eng.); potentille âcre (Fr.); gĭ'tciöde'imĭnĭdji'bĭk (Ojibwa) | **Roots:** Decoction used for dysentery. Powdered and pricked into the temples or placed in the nostrils to relieve convulsions. Dried, moistened, pulverized and applied to cuts [Ojibwa: 47]. |
| Potentilla norvegica L. ssp. monspeliensis (L.) Asch. & Graebn.  **Syn.:** P. monspeliensis L.  Rosaceae, Herb | Norwegian cinquefoil (Eng.); potentille de Norvège (Fr.); tcode'imînaga'wûnj (Ojibwa) | Used as a physic [Ojibwa: 87].  **Roots and stalk:** Chewed or a decoction used to treat sore throat [Ojibwa: 47]. |
| *Prenanthes* *alba* L.  Asteraceae, Herb | Lion's foot, white lettuce (Eng.); prenanthe blanche (Fr.); dado'cabodji'bĭk, weca'wûs wa'ckwînêsk (Ojibwa) | "Milk" used as a diuretic [Ojibwa: 87].  **Roots:** Powdered and put in a broth used in confinement [Ojibwa: 47]. Used as a "female remedy" [Ojibwa: 87]. |
| Prosartes trachycarpa S. Watson  **Syn.:** *Disporum trachycarpum* (S. Wats.) Benth. & Hook. f.  Liliaceae, Herb | rough-fruited fairybells (Eng.); prosartès à fruits rugueux (Fr.) | **Whole plant:** Mixed with another plant and drunk as a spring tonic [Metis: 13]. |
| *Prunella vulgaris* L.  Lamiaceae, Herb | selfheal, heal-all (Eng.); prunelle vulgaire, brunelle, herbe au charpentier (Fr.); name'wûskons', basi'bûgûk (Ojibwa) | Used in sore throat [Cree: 42]. "Women medicine" [Ojibwa: 47].  **Leaves:** Tea used for fever [Algonquin: 69].  **Roots:** Used in compound medicine as a "female remedy" [Ojibwa: 87]. Decoction combined with catnip and taken as a physic [Ojibwa: 47]. |
| *Prunus* *americana* Marsh.  Rosaceae, Tree | wild plum (Eng.); prunier d'Amérique (Fr.); bogēsanōk, bû'gesana'tig (Ojibwa) | **Bark and roots:** Crushed and boiled with the roots of other plants as a remedy for diarrhoea [Ojibwa: 84].  **Bark:** Used as a disinfectant [Ojibwa: 47].  **Roots:** Decoction used against intestinal worms [Ojibwa: 47].  **Inner bark:** Mixed with the trunk of a young *Pinus strobus* and the inner bark of *Prunus serotina* in a decoction used to treat cuts and wounds [Ojibwa: 47]. |
| *Prunus nigra* Aiton  Rosaceae, Tree | Canada plum (Eng.); prunier noir, prunier sauvage (Fr.) | **Inner bark:** Tea drunk in cough [Algonquin: 69]. |
| *Prunus pensylvanica* L.f.  Rosaceae, Tree | pine cherry, bird cherry, fire cherry (Eng.); cerisier de Pennsylvanie, petit merisier (Fr.); pusawemina, pasisāwimin, pāsuwiymayātik (Cree); maskwetzi'minak (Maletice); kusigwa'kumi'nŏk, bae'wimînûn (Ojibwa); wesagatuk (Atikamekw); apueiminanatuk, apueiminânakashî (Montagnais) | Mixed with *Taxus canadensis* in a tea used for rheumatism [Algonquin: 69].  **Bark, fruits, leaves, and roots:** Tea taken in fever [Metis: 13].  **Bark:** Tea taken to treat bronchitis, cough and blood poisoning [Algonquin, Cree: 69; Innu: 72]. Steeped and taken in erysipelas [Malecite: 65]. Compress used on swellings and sprain [Montagnais: 71].  **Bark and branches:** Paste, sometimes mixed with *Sorbus americana* used for problems in shoulders, joints, nerves and heart [Montagnais: 71].  **Inner bark:** Infusion to treat sore eyes [Cree: 95]. Used on cuts, a tea drunk for colds, and boiled to a jelly used to treat burns [Algonquin: 75]. Used for cough [Ojibwa: 87; Montagnais: 71].  **Wood:** Dried without bark and used in prickly heat or chafed skin [Malecite: 65].  **Roots:** Decoction given for stomach pain or disorders [66; Ojibwa: 84]. Grated and boiled in water to make a syrup applied to umbilical cord [Atikamekw: 73]. Herbal water to treat whooping cough [Cree: 93].  **Fruits:** Eaten raw to fight cough [Montagnais: 71].  **Leaves or twigs:** Used to fight cough [Montagnais: 71]. |
| *Prunus serotina* Ehrh.  Rosaceae, Tree | rum cherry, black cherry (Eng.); cersier tardif (Fr.); we'kwanum (Malecite); ikwe'mîc (Ojibwa) | **Bark:** Steeped and drunk for cough [Algonquians: 63, Mi'kmaq: 60]. Used in cough and cold [Mi'kmaq: 61]. Steeped in water, added to beaver castor and a little gin and given in cough and cold, and consumption in men [Maletice: 65]. Tea as a remedy for cough and cold [Mi'kmaq: 62; Ojibwa: 87]. Part of a compound medicine for small pox [Mi'kmaq: 43].  **Fruits:** Steeped to make a bitter tonic [Algonquians: 63, Mi'kmaq: 60, 62].  **Inner bark:** Applied to external sores, an infusion is also given to relieve pain and soreness of the chest [44; Ojibwa: 84]. Mixed with the trunk of a young *Pinus strobus* and the inner bark of a young *Prunus americana* in a decoction used to treat cuts and wounds. Boiled and the water used as a wash to treat scrofula. Decoction used as a disinfectant [Ojibwa: 47].  **Roots:** Decoction against intestinal worms and cholera [Ojibwa: 47]. Powdered and applied to skin ulcers. Mashed fresh and used as a poultice [Ojibwa: 47]. |
| *Prunus* sp.  Rosaceae, Tree | red cherry (Eng.) | **Bark:** Steeped and the liquid drunk to prevent high blood pressure [Mi'kmaq: 62].  **Roots:** Decoction applied to skin ulcers or on painful women breasts [Ojibwa: 47]. |
| *Prunus virginiana* L.  Rosaceae, Tree | chokeberry (Eng.); cerisier de virginie, cerisier à grappes (Fr.); sisa^n^'wewi'nakâ^n^sh', a'sasawe'mînaga'wûnj (Ojibwa); takwahīmināna, takwēhiminān, tākwuhiymin (Cree); Pisheshueminatuk (Innu) | **Bark:** Steeped and drunk in diarrhoea [Algonquians: 63, Mi'kmaq: 60, Cree: 42]. Boiled to make an emetic drink to relieve stomach ache [Cree: 13]. Decoction used as a hair ointment, or mixed with roots of *Veronicastrum virginicum* and taken before breakfast to treat scrofula [Ojibwa: 47]. Tea used as a purgative and emetic [Cree: 96]. Herbal water taken for cold [Cree: 93].  **Inner bark:** Steeped with roots of *Corylus* sp*.* and white oak, and the heart of *Ostrya virginiana* wood and taken to treat lung hemmorhages. Decoction used for sore throat, stomach cramps [Ojibwa: 47], or diarrhoea [66]. Tea used for lung trouble [Ojibwa: 87].  **Leaves, stem, bark, and roots:** Tea used for colds, fevers, pneumonia [Cree, Metis: 13; Innu: 72], to clear phlegm from the throat, or for high blood pressure or heart problems [Cree: 13].  **Branchlets:** Used to make a drink taken during gestation [Ojibwa: 84].  **Roots:** Part of a compound medicine for diarrhoea in children [Cree: 13]. Mixed with *Acorus calamus* and given for cough or blood poisoning [Algonquin: 69]. Herbal water taken against flu [Cree: 93].  **Fruits:** Used as an astringent [Ojibwa: 47] or to treat diarrhoea [66]. |
| *Prunus virginiana* var. *demissa* (Nuttall) Torrey  **Syn:** *Prunus* *virginiana* L. var. *melanocarpa* (A. Nelson) Sarg.  Rosaceae, Shrub | western chokecherry, black chokecherry (Eng.); cerisier du Pacifique (Fr.) | **Roots:** Tea used to treat stomach disorders [Cree: 96]. |
| *Pteris aquilina* L.  Pteridaceae, Herb | brake (Eng.); fougère-aigle (Fr.); ana'ganûck (Ojibwa) | **Roots:** Tea used to alleviate cramps in the stomach [Ojibwa: 87].  **Leaves:** Dried and smoked upon live coals to relieve headache [Ojibwa: 87]. |
| *Pteris* sp.  Pteridaceae, Herb | brake (Eng.) fougère (Fr.) | **Fronds:** Used to make a bed for weak babies or old people [Mi'kmaq: 60; Algonquians: 63]. |
| *Pulsatilla* *patens* (L.) Mill. ssp. *multifida* (Pritz.) Zamels  **Syn.:** Anemone patens L., *P. hirsutissima* (Pursh) Britton  Ranunculaceae, Herb | prairie pasqueflower, cutleaf anemone (Eng.); pulsatille multifide (Fr.); gogeda'djibûg (Ojibwa) | **Leaves:** Powdered and smelled to treat headache [Ojibwa: 47]. Used as a counter-irritant in rheumatism and neuralgia [44]. |
| *Pycnanthemum* *virginianum* (L.) T. Dur. & B.D. Jacks. ex B.L. Rob. & Fernald  **Syn.:** *Koellia* *virginiana* (L.) MacMill.  Lamiaceae, Herb | Virginia mountainmint (Eng.); pycnanthème verticillé (Fr.); name'wûckon' (Ojibwa) | **Leaves:** Decoction made with equal parts of *Nepeta cataria* given in fever [Ojibwa: 47].  **Roots:** Powdered and made into a decoction for amenorrhoea [Ojibwa: 47]. |
| *Pyrola asarifolia* Michx.  Pyrolaceae, Herb | asarum-leaved wintergreen (Eng.); pyrole à feuilles d'asaret (Fr.); sasdzaghé (Chipewyan); amiskowehtawakewuskos, amiskōtawakayipak, miskīsikōmaskī(h)kīh, wāwipak (Cree) | Decoction taken to stop blood coughing [Cree: 95].  **Leaves:** Mashed with lard and put on a cut to stop bleeding and promote healing [Dene: 13; Chipewyan: 92]. Chewed to relieve toothache [Dene: 13; Chipewyan: 92]. Decoction used as a diuretic to treat severe back pain from kidney trouble including kidney stones and urinary tract blockage or scanty urination [Dene: 13]. Infusion used to treat sore eyes [Cree: 95]. |
| *Pyrola* *asarifolia* Michx. ssp. *asarifolia*  **Syn.:** *P. uliginosa* Torr. & A. Gray ex Torr.  Pyrolaceae, Shrub | wintergreen (Eng.); pyrole des marais (Fr.) | Cooked with *Petasites sagittatus* and the mixture used as a wash to treat chickenpox [Dene: 17].  **Leaves:** Used for gonorrhea, kidney trouble and for blood spitting. Decoction drunk for several ailments [Algonquians: 63]. |
| *Pyrola* *elliptica* Nutt.  Pyrolaceae, Herb | waxflower shinleaf (Eng.); pyrole elliptique (Fr.) | **Roots:** Boiled and drunk for weakness [Algonquians: 63]. |
| *Pyrola* *grandiflora* Radius  Pyrolaceae, Herb | arctic pyrola, arctic wintergreen (Eng.); pyrole à grandes fleurs (Fr.) | **Leaves:** Crushed and a warm poultice used to relieve pain caused by burns or for arthritic or joint pain or pain from cuts [Dene: 100]. |
| *Quercus alba* L.  Fagaceae, Tree | white oak (Eng.); chêne blanc (Fr.); mītig'ōmish' (Ojibwa) | **Acorns:** Used to promote thirst [Mi'kmaq: 62, 60] or as a cure for ulcers [Algonquians: 63].  **Bark:** Steeped and drunk for bleeding piles [Algonquians: 63]. Root bark and inner bark of the trunk of this and other oaks used in a decoction for diarrhoea [44; Ojibwa: 84]. |
| *Quercus* *macrocarpa* Michx.  Fagaceae, Tree | bur oak (Eng.); chêne à gros fruits (Fr.); mî'tîgo'mîc (Ojibwa) | **Inner bark:** Extract used in lung trouble and chest pain [Chippewa: 91]. Mixed with inner bark of *Quercus rubra* and *Populus tremuloides*, root, bud and blossom of *Populus balsamifera* and root of *Polygala senaga* in a decoction used for heart troubles [Ojibwa: 47].  **Bark:** Used as an astringent, to bandage a broken foot or leg [Ojibwa: 87]. Decoction used as a laxative [91].  **Roots:** Decoction taken to treat cramps [Ojibwa: 47]. |
| *Quercus rubra* L.  Fagaceae, Tree | red oak (Eng.); chêne rouge (Fr.); wabeik emkwanimozi (Malecite); wisug'emitig'omish', me-te-ko-mesh, mîtîgo'mîc , wi'sugi'mĭtĭgo'mĭc (Ojibwa) | **Bark:** Steeped in water with buds or young cones of *Abies balsamea* and *Tsuga canadensis* and given in diarrhoea [Malecite: 65]. Tea prepared with bark and root of *Salix discolor, Pinus strobus, Pinus banksiana* and *Arctostaphylos uva-ursi* given in fainting and fits [Ojibwa: 86]. Used for heart troubles and bronchial affections [Ojibwa: 87]. |
| **Ranunculus acris* L.  Ranunculaceae, Herb | buttercup, tall buttercup (Eng.); renoncule âcre, bouton d'or (Fr.) | Used against cancer [Mi'kmaq: 62].  **Leaves,** **flowers or fruits:** Crushed and inhaled for headache [Abenaki: 67; Algonquin: 68; Montagnais: 60; Mi'kmaq: 60]. |
| *Rhexia virginica* L*.*  Melastomaceae, Herb | Virginia meadow beauty, vigenar wood (Eng.); rhéxie de Virginie (Fr.) | **Leaves and stems:** Made into a brew to clean the throat and used as a sour drink [Algonquians: 63, Mi'kmaq: 60]. |
| *Rhinanthus minor* L.  Scrophulariaceae, Herb | little yellow rattle (Eng.); petit rhinanthe (Fr.) | Used to treat fits and epilepsy [Mi'kmaq: 62]. |
| *Rhododendron groenlandicum* (Oeder) K.A. Kron & W.S. Judd **Syn.:** *Ledum* *groenlandicum* Oeder  Ericaceae, Shrub | Labrador tea; Hudson's Bay tea (Eng.); thé du Labrador (Fr.); muskeegobug, muckig'obûg (Ojibway); muskekopukwa, muskekopakwa, muskakopukwu, maskīkowāpoy, maskēkopakwa, maskēkopakwatī, tīmaskīk (Cree); pusipga'skill (Malecite); jabak (Abenaki); îkûta (Montagnais); miluepuk (Innu); lidu masgit, gots'ago (Dene); nágodhi (Chipewyan) | Medicinal tea [Cree: 94; Innu: 72]. Used for head colds [Abenaki: 67]. Diuretic. Decoction used to treat pneumonia and, mixed with *Acorus calamus*, whooping cough [Cree: 95]. Tea drunk by women to facilitate childbirth [Algonquin: 68]. Used in nosebleeds [Ojibway: 89]. Decoction used to prevent hair loss or to treat eye infections [Cree: 13]. Steeped and used for kidney troubles [Malecite: 65]. Tea drunk in rheumatic complaints, to strengthen the stomach, relieve headhache and promote perspiration. Powder applied to gangrenes, contusions, and excoriations [Cree: 78]. Used in arthritis, or muscle pain. Tea drops put on abscesses. Broth applied in sore mouth. Tea taken as an energy drink [Cree: 81]. Used to treat cold [Dene: 100]. Boiled with fireweed and the tea drunk to speed up child birth [Dene: 101].  **Whole plant:** Mixed with another plant in a decoction drunk to treat cold or chest pain [Cree: 13]. Boiled and liquid used as a disinfectant for wounds and skin sores. Tea used for urinary tract problems [Cree: 80, 81].  **Leaves:** Decoction or infusion taken as a diuretic [Cree: 76; Algonquians: 63]. Steeped to make a tonic tea [Algonquians: 63]. Fresh leaves chewed and applied to wounds [Cree: 42]. Either chewed and the juice swallowed or made into a tea and drunk to treat stomach flu and diarrhoea [Dene: 13; Chipewyan: 92], chills and bad breath [Cree: 13]. Tea with other herbs taken to treat cold, infants teething pain, as a system cleanser [Cree: 13]. Tea given to nervous people to relieve tension, and powdered leaves applied to burns, or wet eczema [Dene: 13]. Decoction drunk and leaves wrapped in a cloth and applied to the head to treat migraine. Decoction drunk to treat a burning sensation during urination. Applied to wounds [Cree: 13]. Steeped and drunk as a general medicine, as a tonic, or against kidney problems [Mi'kmaq: 62]. Used as a tonic [Chippewa: 88]. Used in diabetes [Cree: 77, 82, 83]. Poultice applied to burns and scalds. Grated and used against headache [Algonquin: 68]. Mixed with grease or pitch to make an ointment applied to burns, itchy skin, sores on hands, and chapped skin including cracked nipple to which a leaf is applied directly. Mixed with fish oil and applied to the umbilical scab to promote healing. Powdered and applied directly to a baby's skin to treat rashes in the skin folds [Cree: 94, 95]. Chewed and juice swallowed to treat cold and sore throat Boiled and compress used to treat fever [Cree: 81]. Tea drunk in diarrhoea, arthritis, chest pain and back ache [Cree: 80]. Tea used to treat headache [Dene: 98]. Herbal water taken for heart and kidney problems. Dried, crushed, mixed with lard and applied to burns. Boiled and used to soak affected parts to treat arthritis [Cree: 93].  **Leaves and twigs:** Steeped and drunk for chill or to purify blood [Algonquians: 63]. Tea used as a tonic, in cold and headache [Algonquin: 69]. Macerated or prepared into a decoction to fight urinary tract problems. Dried and put into a hot wet towel and used as a compress for children urinary tract problems [Montagnais: 71]. Decoction cooled and used to soak joints affected by arthritis [Cree: 13]. Tea taken for stomach pain, diarrhoea, headache and cough [Cree: 81].  **Roots:** Decoction used to treat cold and clean the stomach [Cree: 13]. Tea drunk to treat chest pain [Dene: 101]. |
| Rhododendron tomentosum Harmaja **Syn.:** *Ledum* *palustre* L. ssp. *decumbens* (Aiton) Hultén  Ericaceae, Shrub | northern Labrador tea (Eng.); petit thé du Labrador (Fr.); lidu masgit, gots'ago (Dene); nágodhi (Chipewyan) | Tea taken for cold [Dene: 100].  **Leaves:** Boiled and drunk as a tea for stomach problems [Dene: 17; Chipewyan: 92] or headache [Dene: 98]. Used to treat diabetes [Cree: 82].  **Leaves, stem and flowers:** Tea used for good health. Steam from tea used to clear congested nasal passages [Dene: 99].  **Roots:** Tea drunk to cure chest pain [Dene: 101]. |
| *Rhus aromatica* Aiton  Anacardiaceae, Shrub | white sumac, fragrant sumac (Eng.); sumac aromatique (Fr.); bŏkkwan'ībŏk (Ojibwa) | **Roots:** Decoction taken to cure diarrhoea [Objiwa: 84].  **Bark and fruits:** Used in medicinal ceremonies [Ojibwa: 86]. |
| *Rhus copallinum* L.  Anacardiaceae, Tree | shining sumac, winged sumac (Eng.); sumac brillant (Fr.) | **Fruits and bark:** Medicinal [Mi'kmaq: 61]. |
| *Rhus glabra* L.  Anacardiaceae, Tree | smooth sumac, dwarf sumac (Eng.); sumac glabre (Fr.); pakwan-mins, bakwa'nak, maki'bûg (Ojibwa) | Medicinal [Ojibwa: 86]. Boiled and poured into the ear to treat earache [Mi'kmaq: 43]. Decoction used in dysentery [Ojibwa: 47].  **Bark:** Tea used as a hemostatic [Ojibwa: 86].  **Inner bark:** Used in combination with other planst as an astringent [Ojibwa: 86].  **Flowers:** Steeped and used for sore eyes [Ojibwa: 86]. Chewed to cure sore mouth. Drink taken as a remedy for asthma [Ojibwa: 85].  **Leaves:** Used in poultices [Ojibwa: 86] or to wash sore mouth [Ojibwa: 85].  **Fruits:** Used as a throat cleanser [Ojibwa: 86].  **Roots:** Steeped to make a drink to treat cold or used as an emetic [Ojibwa: 85]. |
| *Rhus typhina* L.  **Syn.:** R. hirta (L.) Sudw.  Anacardiaceae, Tree | staghorn sumac (Eng.); sumac vinaigrier (Fr.); sla'nimus (Malecite); bakwanâtig, bakwana'tîg (Ojibwa) | Used in sore throat [Mi'kmaq: 43].  **Fruits and leaves:** Crushed and prepared as a gargle for sore throats [55].  **Roots:** Used to stop haemorrhaging [Ojibwa: 86], or mixed with other plants for rheumatism [Algonquin: 69].  **Flowers:** Decoction taken to treat stomach pain [Ojibwa: 47].  **Roots and fruits:** Steeped and taken as a blood purifier, or mixed with blackberry root, mountain holly, orange-red lily root and mountain raspberry root and used for consumption or for cough and fever [Malecite: 65]. |
| *Ribes* *americanum* Mill.  Grossulariaceae, Shrub | wild black currant, American black currant (Eng.); gadellier d'Amérique (Fr.) | **Whole plant:** Herbal water taken to induce menses and to treat diabetes [Cree: 93]. |
| *Ribes* *glandulosum* Grauer  Grossulariaceae, Shrub | skunk currant (Eng.); gadellier glanduleux (Fr.); mīthicīmin (Cree); wabos'odji'bĭk (Ojibwa) | **Stems:** Used alone or with *Rubus idaeus* to make a decoction given to prevent blood clotting after birth [Cree: 95].  **Roots:** Decoction taken in back pain and "women diseases" [Ojibwa: 47]. |
| Ribes hudsonianum Richardson  Grossulariaceae Shrub | northern black currant (Eng.); gadellier de la Baie d'Hudson (Fr.) | **Roots:** Boiled with other plants to make a drink for cold [Dene: 13].  **Stems:** Mixed with *Ribes oxycanthoides* in a decoction used to treat sickness after childbirth [Cree: 95]. Decoction with leaves and flowers taken as a cough medicine [Dene: 98].  **Roots and bark:** Medicinal [Ojibwa: 86]. |
| *Ribes lacustre* (Pers.) Poir.  Grossulariaceae, Shrub | swamp gooseberry (Eng.); gadellier lacustre (Fr.); kâkuminânakashî (Montagnais) | **Bark:** Tea drunk as a treatment for diarrhoea and cold [Metis: 13].  **Leaves:** Tea drunk to prevent miscarriages [Metis: 13].  **Roots:** Tea used against diarrhoea or blood in feces [Montagnais: 71]. |
| *Ribes oxyacanthoides* L.  Grossulariaceae, Shrub | Canada gooseberry (Eng.); groseiller du Nord, groseillier hérissé (Fr.); sapoominak, sāpōmin, sābuwmin (Cree); cabo'mĭnaga'wûnj (Ojibwa); daghochį (Dene); daghósjíé (Chipewyan) | **Roots:** Decoction drunk to stimulate menstruations [Dene: 13; Chipewyan: 92].  **Stems:** Steeped in boiling water and the tea drunk by mothers after childbirth to stop excessive bleeding [Metis: 13]. Mixed with *Ribes hudsonianum* in a decoction used to treat sickness after childbirth [Cree: 95]. Boiled and tea drunk to cure mouth infections, sore stomach and to relieve shortness of breath. Solution also used for sore eyes [Dene: 101].  **Fruits:** Decoction used to treat weakness and back pain in women [Ojibwa: 47]. |
| *Ribes* *triste* Pall.  Grossulariaceae, Shrub | wild red currant (Eng.); gadellier amer (Fr.); mîci'tcimînûk, cigagwa'tĭgon (Ojibwa); eneeyù' (Dene) | **Whole plant:** Tea used for stomach problems [Dene: 99].  **Leaves:** Used to prepare "female remedy" [Ojibwa: 87]. Applied to burns [Dene: 100].  **Roots and stalk**: Decoction used to treat gravel [Ojibwa: 47].  **Stalk:** Mixed with roots of *Aralia racemosa* and *Aralia nudicaulis* in a decoction taken in amenorrhoea [Ojibwa: 47].  **Bark:** Herbal water used to induce menses [Cree: 93]. |
| *Rosa acicularis* Lindl.  Rosaceae, Shrub | prickly rose (Eng.); églantier, rosier aciculaire (Fr.); kaminakuse, okīnīak, okwāminalwasiāhtik, okinīwapīgwīnīwa, ogiminakasiatik, ōginīatik, owkiniy, owkāmnekusiy, ōkinī (Cree); dabhà (Dene); í^n^tsólé (Chipewyan) | **Branches:** Boiled to make a decoction drunk to relieve excessive menstruation [Cree: 13].  **Roots:** Decoction given to children to treat diarrhoea, used as eye drops to treat soreness such as from snow blindness [Cree, Dene: 13; Chipewyan: 92]. Infusion used to treat sore eyes [Cree: 95, 96]. Boiled and taken as a cough medicine [Cree: 95; Dene: 98]. Herbal water taken to correct irregular menses and to treat chest cold [Cree: 93].  **Flowers:** Eaten raw to prevent or treat cold and fever, petals used as a heart tonic [Dene: 13]. Petals boiled, strained and used as eye drops or as an eye wash to remove dirt and infection [Dene: 99, 100]. Also used for heat rash and cuts [Dene: 99]. Boiled and tea drunk to cure mouth infections, sore stomach, shortness of breath and cough [Dene: 101]. Petals chewed and applied to bee stings [Dene: 98].  **Leaves:** Chewed and put on bee stings to prevent swelling and release poison [Dene: 17].  **Fruits:** Boiled and drunk as a tea to treat cold and to loosen up the chest [Dene: 17]. Eaten fresh to treat summer cold Also good for the heart [Dene: 99]. |
| *Rosa* *arkansana* Porter  Rosaceae, Shrub | wild prairie rose (Eng.); rosier des prairies (Fr.); bi'jikiwi'ginĭg (Ojibwa) | **Roots:** Mixed with the roots of other plants (*Solidago juncea, Polygala senega, Artemisia frigida,* and *Astragalus crassicarpus*) in a decoction taken internally for convulsions. Mixed with *Artemisia frigida, Astragalus crassicarpus* and *Polygola senega* in a decoction used on wounds to stop bleeding [Ojibwa: 47]. |
| *Rosa* *blanda* Aiton  Rosaceae, Shrub | wild rose (Eng.); rosier inerme, rosier sauvage, églantier (Fr.); o'ginīk (Ojibwa) | Used to prevent scurvy [55].  **Roots:** Infusion in lukewarm water applied to inflamed eyes [Ojibwa: 84].  **Fruits:** Skin used for stomach trouble and indigestion [Ojibwa: 87].  **Flowers:** Powdered and used to relieve heart-burn [Ojibwa: 87]. |
| *Rosa* sp.  Rosaceae, Shrub | wild rose (Eng.); rosier sauvage (Fr.); kenukatia-mins, ogĭni'mĭnaga'wûnj (Ojibwa) | **Roots:** Mixed with the roots of blackberry and blueberry in an infusion taken for diarrhoea [Ojibwa: 85]. Crushed, steeped in water, and the decoction used as an eye-wash [44; Sikani: 97].  **Inner bark:** Soaked in warm water and squeezed over the eyes [Ojibwa: 47]. |
| *Rosa* *virginiana* Mill.  Rosaceae, Shrub | Virginia rose, dwarf wild rose (Eng.); rosier de Virginie (Fr.); oginī'minaga^n^'mŏs, oki-ni-mi-nah-gash (Ojibwa) | **Roots:** Infusion in hot water applied to sore eyes [Ojibwa: 84].  **Roots and bark:** Medicinal [Ojibwa: 86]. |
| *Rosa* *woodsii* Lindl.  Rosaceae, Shrub | woods' rose (Eng.) | **Leaves:** Chewed and put on bee stings to prevent swelling and release poison [Dene: 17].  **Fruits:** Boiled and drunk as a tea to treat cold and to loosen up the chest [Dene: 17]. |
| *Rubus allegheniensis* Porter  Rosaceae, Shrub | wild blackberry (Eng.); mûrier, ronce alléghanienne (Fr.); tetéga-min, o'dataga'gomic (Ojibwa) | **Roots:** Mixed with roots of blueberry and a species of wild rose, steeped in water to make a tea as a remedy for diarrhoea, also steeped to make a medicine to correct the condition of a pregnant woman threatened with miscarriage because of overexertion or a similar cause [Chippewa: 85]. Mixed with staghorn sumach, mountain holly, orange-red lily root and mountain respberry and used in consumption, cough and fever [Malecite: 65]. Tea for arresting flux [Ojibwa: 87].  **Stem:** Tea used as a diuretic [Ojibwa: 87]. |
| *Rubus chamaemorus* L.  Rosaceae, Herb | cloud berry (Eng.); ronce petit-mûrier, plaquebière, chicoutai (Fr.); nakàl, gors'okà (Dene) | Mixed with staghorn sumach, blackberry root, mountain holly, orange-red lily root and used in consumption, cough and fever [Malecite: 65].  **Leaves:** Crushed and applied to burns, insect bites, and bee stings [Dene: 100].  **Flowers:** Boiled and tea applied to sore eyes [Dene: 101].  **Berries:** Chewed and placed on wounds and sores [Dene: 101]. |
| *Rubus* *frondosus* Bigelow  Rosaceae, Herb | black berry (Eng.); ronce de Brainerd (Fr.); oda'tagago'mĭnaga'wûnj (Ojibwa) | **Roots:** Decoction taken in amenorrhoea, or mixed with inner bark of *Quercus macrocarpa* in a decoction taken for lung trouble [Ojibwa: 47]. |
| *Rubus idaeus* L.  Rosaceae, Herb | raspberry (Eng.); framboisier, ronce du mont Ida (Fr.); anosh'kanek, ayooskunak, ayuwskun, uyooskan, ayōsikan, athōskunatikwah, athōskan (Cree); meskwa'mînaga wûnj (Ojibwa); mikominatuk, miskiwimin (Atikamekw); alushikanatuk (Innu); ts'eenakal (Dene); tthekálhjíé (Chipewyan) | **Berries:** Taken as a heart medicine [Dene: 17].  **Leaves:** Tea used to give strength to women giving birth and to aid the process [Metis: 13].  **Stem:** Dried and boiled to make a decoction for treating fever [Cree: 13].  **Roots:** Cooled decoction used as eye drops to treat soreness such as from snow blindness [Dene: 13; Chipewyan: 92]. Tea from root bark used to heal sore eyes [Ojibwa: 87]. Boiled with birch inner bark and the vapor inhaled to treat asthma [Cree: 13]. Tea used against diarrhoea [Algonquin: 69; Innu: 72]. Boiled and used to treat bloody urine [Atikamekw: 73].  **Runners:** Steeped and the liquid taken for stomach problems [Mi'kmaq: 62].  **Roots and stem:** Mixed with other plants in a decoction used as a drink for children with diarrhoea, or used to wash skin infections [Cree, Dene: 13]. Decoction used to treat teething pain, to help women recover after childbirth, and to slow menstrual bleeding [Cree: 95].  **Leaves and fruits:** Used as a tonic and diarrhoea remedy [Mi'kmaq: 62].  **Stem and leaves:** Tea drunk to treat diarrhoea [Dene: 100]. |
| *Rubus* *idaeus* L. ssp. *strigosus* [Michx.) Focke  **Syn.:** *R. strigosus* Michx. *R. idaeus* L. var. *melanolasius* (Dieck) R.J. Davis  Rosaceae, Shrub | wild red raspberry (Eng.); framboisier sauvage (Fr.); mĭs'komĭnaga'wûnj (Ojibwa) | **Leaves:** Infusion given in cholera and dysentery as an astringent and tonic [Cree: 74].  **Roots and stem:** Boiled and used for measles [Ojibwa: 85].  **Roots:** Decoction used in dysentery. Inner bark of roots soaked in water and squeezed over eyes to treat eye problems [Ojibwa: 47]. Tea used for stomach problems [Cree: 96]. Boiled with the roots of *Equisetum pratense* and taken for stomach problems [Dene: 98]. |
| *Rubus occidentalis* L.  Rosaceae, Herb | wild black raspberry (Eng.); ronce occidentale, mûrier (Fr.); kadem-sku-min, makadē'wĭskwi'minŏk , oda'tagago'mĭnaga'wûnj (Ojibwa) | **Roots:** Boiled and used as an eye wash for sore eyes [Ojibwa: 85]. Decoction taken in stomach pain, *Rubus idaeus* sometimes used as a substitute [Ojibwa: 84]. Decoction used for stomach and bowel troubles [Ojibwa, Penobscot: 66]. Decoction given in back pain and "women diseases" [Ojibwa: 47]. |
| *Rubus* *pubescens* Raf. var. *pubescens*  **Syn.:** *R. triflorus* Richardson  Rosaceae, Herb | dwarf raspberry (Eng.); ronce pubescente, catherinettes (Fr.); wabimi'ndgak atho'sis wimi'nol (Malecite) | Steeped with wild strawberry and given in irregular menstruation [Malecite: 65]. |
| *Rubus* sp.  Rosaceae, Herb | blackberry (Eng.); mûre (Fr.); saptewemi'nus (Malecite); assasawemin, otatakahomin (Atikamekw) | Steeped and given at frequent intervals to treat diarrhoea [Malecite: 65].  **Runners:** Steeped and given for stomach problems [Mi'kmaq: 62].  **Twigs:** Scraped to make a tea taken against bronchitis [Atikamekw: 73]. |
| *Rudbeckia laciniata* L.  Asteraceae, Herb | cutleaf coneflower (Eng.); rudbeckie laciniée (Fr.); gi'zûswe'bigwa'ĭs (Ojibwa) | **Roots:** Mixed with equal amounts of *Caulophyllum thalictroides* roots in a decoction taken in indigestion [Ojibwa: 47].  **Flowers:** Mixed with *Agastache anethiodora* and *Solidago altissima* to make a poultice applied to burns [Ojibwa: 47]. |
| *Rumex* *altissimus* Alph. Wood  Polygonaceae, Herb | tall dock, pale dock (Eng.); patience élevée (Fr.) | Medicinal [Ojibwa: 86]. |
| *Rumex aquaticus* L.  Polygonaceae, Herb | western dock, yellow dock (Eng.); patience occidentale, doche (Fr.); osaw ochepihk, pikwataskōpīwāhtik (Cree) | **Whole plant:** Decoction used as a wash to treat joint pain [Metis: 13].  **Roots:** Bruised or crushed and used in sores and abrasions [Ojibwa: 44]. Root bark chewed and applied to serious wounds to stop bleeding and promote healing [Metis: 13]. Used to treat high blood pressure and heart ailments [Dene: 13]. |
| *Rumex* *arcticus* Trautv.  Polygonaceae, Herb | arctic dock (Eng.); patience arctique (Fr.) | **Leaves:** Tea used as a skin wash [Dene: 100].  **Roots:** Tea used as a skin wash [Dene: 100]. |
| **Rumex crispus* L.  Polygonaceae, Herb | curled dock (Eng.); patience crépue, rumex crépu (Fr.); o'zabetshi'wĭk, ci'obûg , oza'widji'bĭk (Ojibwa) | **Roots:** Steeped with *Conioselium chinense, Chimaphila umbellate* and *Tsuga canadensis* and given in bladder problems [Mi'kmaq: 62]. Bruised or crushed and applied to abrasions and sores [Ojibwa: 84]. Powdered, moistened, spread on a cloth and applied as a poultice for skin itching and eruptions. Pounded and applied to cuts and skin ulcers. Poultice applied to swellings [Ojibwa: 47]. Used to heal cuts [Ojibwa: 87]. |
| **Rumex* *obtusifolius* L.  Polygonaceae, Herb | bitter dock (Eng.); patience à feuilles obtuses, rumex à feuilles obtuses (Fr.); oza'widji'bĭk (Ojibwa) | Used on cuts and ulcers [Ojibwa: 47].  **Roots:** Steeped and applied to skin eruptions [Ojibwa: 47]. |
| *Rumex orbiculatus* A*.* Gray  Polygonaceae, Herb | water-dock (Eng.); patience orbiculaire, rumex orbiculaire (Fr.); pikwataskōpīwāhtik (Cree) | **Whole plant:** Decoction applied to painful joints [Cree: 95]. |
| *Rumex* *salicifolius* Weinm. var. *mexicanus* (Meisn.) C.L. Hitchc.  **Syn.:** R. mexicanus Meisn.  Polygonaceae, Herb | narrow-leaved dock (Eng.); rumex mexicain (Fr.); pikwataskōpīwāhtik (Cree) | **Whole plant:** Decoction applied to painful joints [Cree: 95]. |
| *Rumex* sp.  Polygonaceae, Herb | dock weed (Eng.); patience, rumex (Fr.) | **Rhizomes:** Used as a laxative [Cree: 74]. |
| *Sagittaria cuneata* Sheldon.  **Syn.:** *S. arifolia* Nutt. ex J.G. Sm.  Alismataceae, Herb | arrowhead (Eng.); sagittaire cunéaire (Fr.); wapato (Algonquin); wabasi (Ojibwa); deníeke (Chipewyan) | Remedy for indigestion [Ojibwa: 87].  **Leaves:** Applied fresh as a poultice to inflamed skin caused by scrofula [Cree: 13]. Poultice used in skin disorders, cuts, burns and bee stings [Cree: 96].  **Roots:** Used against tuberculosis [Algonquin: 69]. Medicinal [Chipewyan: 92]. |
| *Sagittaria* *latifolia* Willd.  Alismataceae, Herb | arrowhead (Eng.); sagittaire latifoliée (Fr.); muj'ota'bûk (Ojibwa) | **Roots:** Steeped and taken in indigestion [Ojibwa: 47]. |
| *Salix bebbiana* Sarg.  Salicaceae, Shrub | willow (Eng.); saule de Bebb (Fr.); nepise, nepiseatik, wekope, atikwupamuk, nīpīsīs, nīpīsī, nīpīsīah, nīpīsīgībī, nīpīstakwah (Cree) | **Inner bark:** Decoction alone or mixed with another plant to treat diarrhoea and stomachache [Cree: 13]. Chewed and applied to deep cuts [Cree: 95].  **Twigs:** Decoction taken to treat a toothache [Cree: 13].  **Roots:** Peeled, boiled, and the decoction drunk to relieve fatigue and provide strength. Applied to aching teeth [Cree: 13]. |
| *Salix* *candida* Flueggé ex Willd.  Salicaceae, Shrub | hoary willow (Eng.); saule tomenteux (Fr.); sisi'gewe'mĭsh (Ojibwa) | **Roots:** Decoction made with thick inner bark of roots taken for cough [66; Ojibwa: 84]. |
| *Salix cordata* Michx.  Salicaceae, Shrub | willow (Eng.); saule à tête laineuse (Fr.); kinoze's (Maletice) | **Bark:** Placed in hot water, removed, greased, and applied to blister. [Malecite: 65]. |
| *Salix discolor* Muhl.  Salicaceae, Shrub | pussy willow (Eng.); saule discolor, chatons (Fr.); nīpisī (Cree); wigubi (Atikamekw) | **Inner bark:** Infusion taken to cure diarrhoea [Cree: 95]. Powdered and mixed with boiling water to obtain a paste applied to sore throat [Atikamekw: 73].  **Bark and roots:** Tea prepared with bark and root of *Pinus banksiana*, *Pinus strobus*, *Quercus rubra* and *Arctostaphylos uva-ursi* given in fainting and fits [Ojibwa: 86].  **Roots:** Tea given in stomach troubles, fainting and trembling [Ojibwa: 86]. Boiled until thick and used to treat skin cancer, taken internally to treat kidney ailments [Mi'kmaq: 62]. **Twigs:** Infusion used to activate lactation after childbirth [Atikamekw: 73]. |
| *Salix* *fragilis* L.  Salicaceae, Tree | crack willow (Eng.); saule fragile (Fr.); sizigo'bamîc (Ojibwa) | **Bark:** Used as an astringent, as a styptic and poultice for sores [Ojibwa: 87]. |
| *Salix lucida* Muhl.  Salicaceae, Tree | squaw bush, red willow, shining willow (Eng.); saule brilliant (Fr.); mûckigo'bamîc, zigo'bamîc (Ojibwa) | **Bark:** Infusion drunk to stop vomiting and remove bile from the stomach [Montagnais: 70]. Smoked to relieve asthma. Steeped to make a mash to be put in a bandage on the head for headache. Poultice used to heal sores and stop bleeding from cuts [Ojibwa: 87]. Used to treat mouth sores, sores around the eyes and chest colds [Cree: 80]. |
| *Salix* *nigra* Marsh.  Salicaceae, Tree | black willow (Eng.); saule noir (Fr.); sepastikoos (Cree) | **Bark:** Used fresh as an astringent, haemostatic, tonic, or diuretic [Cree: 74].  **Roots:** Poultice used in bruises, sprains, and broken bones [Algonquians: 63].  **Leaves:** Bruised in hot water and used in sprains and bruises [Algonquians: 63]. |
| *Salix* *planifolia* Pursh  **Syn:** *Salix* *planifolia* Pursh ssp. *planifolia*  Salicaceae, Shrub | tea-leaved willow, diamond-leaved willow, flat-leaved willow (Eng.); saule à feuilles planes (Fr.); waskayabaduk (Cree) | **Bark:** Used to treat diabetes [Cree: 82]. |
| *Salix* *pedicellaris* Pursh  Salicaceae, Shrub | bog willow (Eng.); saule pédicellé (Fr.); sizigo'bamîc (Ojiwe) | **Bark:** Used to treat stomach troubles [Ojibwa: 87]. |
| *Salix* sp.  Salicaceae, Tree | willow, osier (Eng.); saule (Fr.); uapineumitshimatuk (Innu); uâpineu-mîtshima (Montagnais); ozĭ'sĭgo'bimĭc (Ojibwa); k'aii, k'ak (Dene) | Chewed and the saliva applied to external sores. White powdery tops chewed for cough [Sikani: 97]. Used in eye diseases [Abenaki: 67]. Compress used to soothe toothache [Innu: 72]. Heated and used for toothache. Dry willow split into halves and rolled on painful arthritic joints. Tea from dry willow placed in sore eyes and inhaled to cure headache [Dene: 101].  **Bark:** Steeped and drunk in quantities for cold [Algonquians: 63]. Used to treat cuts [Algonquin: 75]. Boiled until it turns to a thick paste, then placed in a towel and used locally as a compress for swells, back pain and other types of body pain (e.g. sprains). Tea used in cough [Montagnais: 71]. Bark from young shoots wrapped around a cut like a bandage [Dene: 99]. Tea drunk to cure urinary tract and stomach problems [Dene: 101]. Boiled and applied to back to treat back paralysis [Cree: 93].  **Inner bark:** Used in diabetes [Cree: 77, 82, 83]. Decoction taken in indigestion [Ojibwa: 47]. Inner bark from young shoots made into a poultice and used as a pain-killer on wounds [Dene: 99]. Tea used in stomach problems [Cree: 96].  **Roots:** Poultice used in bruises, sprains, and broken bones [Algonquians: 63]. Used in dysentry [Ojibwa: 47].  **Leaves:** Bruised in hot water and used in sprains and bruises [Algonquians: 63]. Decoction applied to arthritis and swells [Montagnais: 71]. Crushed or chewed and applied to bee stings and other insect bites, burns, rashes, cuts, and toothache [Dene: 99].  **Bark and leaves:** Poultice used to treat pain or to relieve insect bites [Dene: 100].  **Branches:** Decoction taken as a cough medicine [Dene: 98]. |
| *Sambucus* *nigra* L. ssp. *canadensis* (L.) R. Bolli  **Syn.:** *S. canadensis* L.  Caprifoliaceae, Shrub | common elder, elderberry (Eng.); sureau du Canada, sureau blanc (Fr.); pipigwe-minan (Chippewa) | **Bark:** Used as an emetic and physic [Algonquians: 63; Mi'kmaq: 60]. Tea used as an emetic and laxative [Algonquin: 69].  **Roots:** Steeped and taken as an emetic and physic [Chippewa: 85].  **Flowers:** Dried and used to make a tea drunk as a diaphoretic [66]. |
| *Sambucus racemosa* L.  Caprifoliaceae, Tree | red-fruited elder, red elderberry (Eng.); sureau rouge, sureau pubescent (Fr.); peskigdjila'nimus (Malecite); papaskatcîksi'gana'tig (Ojibwa); mishtukusha (Montagnais) | **Bark:** Decoction taken internally as a purgative [Sikani: 97]. Steeped in water with round wood and given as an emetic [Malecite: 65]. Used as a purgative or emetic [Ojibwa: 87].  **Roots:** Used to make a medicinal tea [Ojibwa: 86].  **Inner bark:** Boiled and the liquid drunk for constipation [Ojibwa: 87].  **Trunk:** Boiled to obtain a decoction used for urinary problems [Montagnais: 71]. |
| *Sanguinaria canadensis* L.  Papaveraceae, Herb | bloodroot (Eng.); sanguinaire du Canada, sang-dragon (Fr.); pe'kniasuk' (Maletice); meskwi-jibik, mĭs'kodji'bĭk (Ojibwa); pabakan'hilan^gn^, papagakanilhôk (Abenaki) | Used as an abortive [Abenaki: 67] or as a blood medicine [Ojibwa: 86]. Juice used in wound infection [Mi'kmaq: 61].  **Rhozomes:** Used as a tonic [Algonquin: 69].  **Roots:** Used to cure sore throat [Ojibwa: 87] or prevent bleeding [Algonquians: 63; Mi'kmaq: 60]. Chewed for heart trouble [Algonquin: 69]. Steeped in water and given in consumption, applied to cuts, and taken for bleeding piles [Malecite: 65]. Steeped and given for tuberculosis [Mi'kmaq: 62]. Mixed with *Caulophyllum thalictroides* in a decoction taken to treat stomach cramps [Ojibwa: 47].  **Rhizomes and roots:** Used as an irritant, narcotic, nauseant and emetic [Ojibwa: 88]. |
| *Sanguisorba canadensis* L.  Rosaceae, Herb | American great burnet (Eng.); sanguisorbe du Canada (Fr.); nishtshikâta (Montagnais) | **Root bark:** Dried and used to prepare a tea for cough [Montagnais: 71]. |
| *Sanicula canadensis* L.  Apiaceae, Herb | Canada sanicle, Canada black snakeroot (Eng.); sanicle du Canada (Fr.); mûkûd'widji'bĭk (Ojibwa) | **Roots:** Powdered, used to make a decoction taken for amenorrhoea. Mixed with *Caltha palustris* in a decoction taken in confinement [Ojibwa: 47]. |
| *Sanicula marilandica* L.  Apiaceae, Herb | black snakeroot (Eng.); sanicle du Maryland (Fr.); midwiminigak atho'sis wimi'nol (Malecite); masan (Ojibwa) | **Roots:** Steeped and given in irregular menstruation [Malecite: 65]. Used as a poultice on cure snake bites. Tea used to cure fevers [Ojibwa: 87]. |
| *Sanicula* *odorata* (Raf.) K.M. Pryer & L.R. Phillippe  **Syn.:** *S.* *gregaria* E.P. Bicknell  Apiaceae, Herb | black snakeroot (Eng.); sanicle grégaire (Fr.); wabimi'negakathosiswimi'nal (Malecite) | **Roots:** Steeped with spikenard and given in kidney trouble [Malecite: 65]. Used for rheumatism, irregular menstruation and slow parturition; also used against snake bites [44]. |
| *Sarracenia purpurea* L.  Sarraceniaceae, Herb | pitcher plant (Eng.); sarracénie pourpre, sabot, oreille de cochon, petits cochons, herbe crapaud (Fr.); alk tsotaco', alicotache, anîtshikâta (Montagnais); ayekitas, ayikitās, ayīkicās, athīkacās (Cree); omakakiwidass (Ojibwa); arikitcakotepik (Atikamekw); makikiotache (Algonquin); ts'ëlitili (Chipewyan) | Steeped and drunk for blood spitting and kidney troubles [Mi'kmaq: 60]. Used against tuberculosis [Mi'kmaq: 62] or lower back pain [Cree: 95]. Decoction used to treat various wounds [Algonquin: 68].  **Whole plant:** Steeped and taken in consumption [Malecite: 65]. Used in diabetes [Cree: 77, 82, 83]. Boiled and water used to wash burns and skin infections [Montagnais: 71].  **Leaves:** Boiled to make a tea used in urinary difficulties [Algonquin: 69] or as a wash for sores and for children's rashes, the same ailments can also be treated by applying a split leaf on the affected parts [Montagnais: 70]. Steeped and used in smallpox [Algonquians: 63; Montagnais: 70]. Used for chronic chest trouble, cough [Dene: 13] and "women's ailments" [Cree, Dene: 13]. Part of a compound medicinal tea taken to ease childbirth [Algonquin: 69; Cree: 13]. Decoction or infusion taken to remedy sickness associated with amenorrhoea [Cree: 95]. Tea medicinal [Chipewyan: 92]. Herbal water taken in fever and urinary tract problems. Crushed and sniffed for headaches [Cree: 93].  **Roots:** Steeped and drunk for blood spitting, sore throat, kidney problems, and pulmonary complaints [Algonquians: 63]. Used as a poultice on cuts [Cree: 13]. Boiled to make a tea used in urinary difficulties [Algonquin: 69]. Decoction given to women to prevent sickness after childbirth, and mixed with other plants in a decoction taken to help expel the afterbirth. Decoction taken in venereal diseases [Cree: 95]. Steeped in water and liquid taken internally to relieve indigestion [Mi'kmaq: 62]. Used in diabetes [Cree: 77]. Tea used to facilitate parturition [Ojibwa: 87]. Used as a diuretic and, mixed with beaver kidney, used to cure urinary tract diseases [Atikamekw: 73]. Poultice used in skin disorders, cuts, burns and bee stings [Cree: 96].  **Rhizomes:** Infusion used in the treatment of smallpox [66; Montagnais: 71]. |
| *Sassafras* *albidum* (Nutt.) Nees  **Syn.:** *S. variifolium* (Salisb.) Ktze.  Lauraceae, Tree | sassafras (Eng.); sassafras officinal (Fr.); menagwakemins (Chippewa) | **Bark roots:** Infusion taken as a springtime medicine to thin the blood [Chippewa: 85]. |
| *Saururus cernuus* L.  Saururaceae, Herb | Indian pepper, lizard's tail (Eng.); saurure penché (Fr.); we-ne-se-bah-gon (Ojibwa) | Stomach medicine [Ojibwa: 86]. |
| *Schoenoplectus* *acutus* (Muhl. ex Bigelow) A. Löve & D. Löve  **Syn.:** *Scirpus* *acutus* Muhl. ex Bigelow  Cyperaceae, Herb | bulrush, tule (Eng.); scirpe aigu, grand jonc (Fr.); kichekumewusk, kiychiykāmiyuwusk, ōkīhcīkamīwask, wechahkamewuskwa, mwaskosīwan (Cree) | **Stem:** Boiled to make a medicine for cough and fever [Metis: 13]. |
| *Scirpus* *microcarpus* J. Presl & C. Presl  **Syn.:** *S. rubrotinctus* Fernald  Cyperaceae, Herb | bulrush (Eng.); scirpe à graines rouges (Fr.); kagskinio'kis (Malecite) | **Roots:** Poultice used in abscesses. Steeped with *Iris versicolor* and used to gargle a sore throat [Malecite: 65]. |
| *Scutellaria galericulata* L.  Lamiaceae, Herb | marsh skullcap (Eng.); toque, tertianaire (Fr.); tcatcabonû'ksîk (Ojibwa) | Used in heart troubles [Ojibwa: 87].  **Leaves and flowers:** Tea and used to treat ulcer [Metis: 13] and fever [Cree: 13]. |
| *Scutellaria* *lateriflora* L.  Lamiaceae, Herb | mad-dog scullcap (Eng.); scutellaire latériflore (Fr.) | Mixed with *Scutellaria pilosa*, *Hypericum punctatum* and *Stylosanthes elatior* in a decoction drunk by women to promote menstruation. Also drunk for diarrhoea and used with other plants for painful breasts [44]. |
| *Shepherdia canadensis* (L.) Nutt.  **Syn.:** *Lepargyrea* *canadensis* (L.) Greene  Elaeagnaceae, Shrub | soapberry, buffalo-berry (Eng.); shepherdie du Canada, graines de boeuf (Fr.); kinipikomina, kinèpikōminānahtik, kinīpikōminā(h)tik emskuwmnā(h)tik (Cree); dinjik jàk (Dene) | Decoction applied externally to treat aching limbs, arthritis, and sore head and face [Cree: 95].  **Whole plant:** Tea used as a tonic [Dene: 100].  **Leaves and stem:** Decoction drunk as a purgative and emetic [Cree: 13, 96], to relieve constipation, tuberculosis [Metis: 13], and used as a wash for cuts, swellings, and skin sores due to impetigo [Metis: 13]. **Shoots:** Tea from new shoots drunk to prevent miscarriages and used as a wash for arthritis [Cree: 95; Metis: 13]. Taken to treat venereal diseases and blood coughing [Metis: 13].  **Stem:** Decoction used for venereal disease [Cree: 95].  **Roots:** Used in heart medicine [Dene: 13; Chipewyan: 92]. Boiled and put on sore or swollen knee [Dene: 17]. Infusion used for blood coughing [Cree: 95]. Boiled with juniper berries and used as a laxative [Dene: 99]. Boiled and used as a rinse for sore lips and mouth [Dene: 98].  **Bark:** Softened in hot water with the bark of pin cherry to make a plaster or bandage for broken bones [Algonquin: 69].  **Inner bark:** Infusion used as a laxative [Cree: 95].  **Berries:** Eaten raw or boiled for heartburns and diabetes. Mixed with water and drunk as a medicinal tea [Dene: 17]. Eaten raw or drunk as a tea for cold or sore throat [Dene: 99].  **Stem and roots:** Tea used in stomach ache and diarrhoea [Dene: 99]. Decoction used in fever [Dene: 98]. |
| **Silene* *latifolia* Poir. ssp. *alba* (Mill.) Greuter & Burdet  **Syn.:** *Lychnis* *alba* Mill.  Caryophyllaceae, Herb | white campion (Eng.); lychnis blanc (Fr.); basi'bûgûk (Ojibwa) | **Roots:** Tea used as a physic [Ojibwa: 87]. |
| *Silphium perfoliatum* L.  Asteraceae, Herb | cup-plant (Eng.); silphe perfolié (Fr.); akûn'damo, asasa'weskûk (Ojibwa) | Used for stomach trouble, and hemorrhage [Ojibwa: 87].  **Roots:** Decoction taken in lung troubles and in amenorrhoea. Dried, pounded and used as a moist compress to stop bleeding from wounds [Chippewa: 47]. Tea used in rheumatic pains [Ojibwa: 87]. |
| *Sium suave* Walter  Apiaceae, Herb | water parsnip (Eng.); berle douce (Fr.); sīwaskātask, sīwaskacāskwos, ōskātask, kowchuskowitoy (Cree) | Smoked to relieve headache [Dene: 98].  **Roots:** Used as a tonic. Eaten raw or boiled with other plants and the decoction drunk to treat chest congestion. Part of a compound decoction used to treat heart trouble, headache, and fever [Cree: 13]. Eaten raw or prepared as a tea for sore throat, cough and cold. Tea also used to treat fever, heart problems and headache. Eaten raw as a tonic and blood cleanser [Cree: 96]. Cancer medicine [Cree: 93]. |
| *Smilax herbacea* L.  Smilacaceae, Vine | carrion flower (Eng.); smilax herbacé, raisin de couleuvre (Fr.); ma'kodji'bĭk, bîgomînaga'wûnj (Ojibwa) | **Roots:** Decoction used as a physic and in kidney troubles [Ojibwa: 47]. Used in lung troubles [Ojibwa: 87]. |
| **Solanum dulcamara* L.  Solanaceae, Herb | bittersweet (Eng.); morelle douce-amère (Fr.); wizagapo'kl (Malecite) | Tea used to treat nausea [Mi'kmaq: 61; Malecite: 65]. |
| **Solanum nigrum* L.  Solanaceae, Herb | nightshade (Eng.); morelle noire (Fr.) | Medicinal [Ojibwa: 86]. |
| *Solidago* *altissima* L.  Asteraceae, Herb | goldenrod (Eng.); verge d'or très élevée (Fr.); a'djidamo'wano (Ojibwa) | **Roots:** Powdered, moistened and applied as a poultice in boils [Ojibwa: 47].  **Flowers:** Dried, moistened with cold water and applied to ulcers. Mixed with flowers of *Agastache anethiodora* and *Rudbeckia laciniata* to make a poultice applied to burns [Ojibwa: 47]. |
| *Solidago canadensis* L.  Asteraceae, Herb | goldenrod (Eng.); verge d'or du Canada (Fr.); chachamos kakew (Cree) | Tea used for heart disease [Algonquin: 69] and fever [Algonquin: 68].  **Leaves and stems:** Boiled and the decoction drunk to treat kidney and bladder problems, or constipation or cold [Dene: 13].  **Roots:** Decoction taken to treat stomach cramps [Ojibwa: 47]. |
| *Solidago* *flexicaulis* L.  Asteraceae, Herb | goldenrod (Eng.); verge d'or à tige zigzaguante (Fr.); a'djidamo'wano (Ojibwa) | Used in "women diseases" [Ojibwa: 47].  **Roots:** Dried and chewed to treat sore throat [Ojibwa: 47]. |
| *Solidago juncea* Aiton  Asteraceae, Herb | early goldenrod (Eng.); verge d'or jonciforme (Fr.); a'djidamo'wano (Ojibwa) | **Roots:** Decoction taken internally in convulsions and in "women complaints" [Ojibwa: 47]. |
| *Solidago multiradiata* Aiton  **Syn.:** *S. virgaurea* var. *multiradiata* (Aiton) Torrey & A. Gray  Asteraceae, Herb | multi-rayed goldenrod, mountain goldenrod (Eng.); verge d'or à rayons nombreux (Fr.) | Used as a tonic [Cree: 42]. |
| *Solidago* *speciosa* Nutt. var. *rigidiuscula* Torr. & A. Gray  **Syn.:** *S. rigidiuscula* (Torr. & A. Gray) Porter  Asteraceae, Herb | showy goldenrod (Eng.); verge d'or voyante (Fr.); o'zawa'bigwûn (Ojibwa) | Astringent and styptic [Ojibwa: 47].  **Roots:** Mixed with roots of *Pulsatilla hirsutissima* in a decoction taken in lung trouble. Decoction taken to treat lung hemmorhage. Combined with bear fat and used as a hair ointment. Decoction used for mouth haemorrhage, as a tonic, and to treat cuts and wounds. Infusion taken to ease labor during childbirth. Boiled and used as a warm compress to treat sprain of strained muscles [Ojibwa: 47].  **Roots and stem:** Decoction taken as a tonic and stimulant [Ojibwa: 47].  **Leaves:** Dried and used to make a decoction given in fever [Ojibwa: 47].  **Stem:** Combined with bear fat and used as a hair ointment. Boiled and used as a warm compress to treat sprain of strained muscles [Ojibwa: 47]. |
| *Sorbus americana* Marsh.  **Syn.:** *Pyrus americana* (Marsh.) DC.  Rosaceae, Tree | American mountain ash, round wood, bear berries (Eng.); sorbier d'Amérique, cormier (Fr.); maskōminānātik, esniywachiywa(h)tik (Cree); mina'kwimus, wik's (Malecite); ah-o-je-mahg (Chippewa); makoomiinaan (Ojibway); maskominanatuk (Atikamekw); mashkumenan, mashkuminânakashî (Innu) | Emetic [Algonquians: 63]. Mixed with *Populus tremuloides* in a tea used to treat constipation [Innu: 72]. Mixed with *Picea mariana*, *Picea glauca*, *Gaultheria procumbens*, *Sambucus nigra*, and wine in a tea used to fortify blood [Algonquin: 68].  **Leaves:** Dried and chewed for throat pain [Montagnais: 71].  **Bark:** Steeped in hot water and given to babies to drink for general disorders and especially for cholera. Decoction drunk to treat general pains in the body and considered good for the bones [Cree: 13], also drunk to stimulate appetite and to purify the blood [Algonquians: 63]. Burned and applied to boil as a poultice. Steeped and given to mothers after childbirth, also used as a tonic [Malecite: 65]. Boiled and used to make a plaster applied to the lower back of pregnant women to facilitate childbirth [Atikamekw: 73]. Boiled and used as a paste to be applied locally on painful body parts, on arthritis or rheumatism. Ground, boiled and the decoction taken for cough. Tea used for heart problems or tootache [Montagnais: 71]. Tea used to treat arthritis and muscular pain [Cree: 96].  **Roots and bark:** Medicinal [Ojibwa: 86].  **Inner bark:** Placed in a hot wet towel and applied to swells as a compress [Montagnais: 71].  **Inner bark and buds:** Tea used to cure weariness and depression [Atikamekw: 73], given for cold, or combined with *Acorus calamus* and used as a tonic [Algonquin: 69].  **Roots:** Infusion drunk for colic [Algonquians: 63]. Extracts used to stimulate appetite [Algonquin: 68]. Tea drunk to treat sore throat, cold and cough [Cree: 96].  **Stem:** Medicinal [Ojibway: 89]. Chewed and also boiled to make a decoction drunk to treat cold, cough, rheumatism, hemorrhaging, headache, heart trouble, sore chest, kidney pain, or to facilitate labor during childbirth [Dene: 13; Innu: 72]. Boiled and the steam inhaled for headache and sore chest [Dene: 13]. |
| *Sorbus* *decora* (Sarg.) C.K. Schneid.  Rosaceae, Tree | northern mountain ash (Eng.); sorbier plaisant (Fr.); maskōminānātik (Cree) | **Branches:** Decoction drunk for the treatment of back pain [Cree: 95]. Used to treat diabetes [Cree: 82].  **Inner bark and branches:** Used in diabetes [Cree: 77].  **Leaves:** Used to treat diabetes [Cree: 82].  **Bark:** Used to treat diabetes [Cree: 82].  **Inner bark and cambium:** Used to treat diabetes [Cree: 82]. |
| *Sorbus* *scopulina* Greene  Rosaceae, Shrub | Greene's mountain-ash (Eng.); sorbier de Greene (Fr.); naidídechëné (Chipewyan) | Used as a compress for pain or rubbed on chest, back and throat for cough and sore throat [Cree: 81]. Tea taken to relieve cold symptoms, headaches, sore chest, or for "blood in the body". Tea also drunk by women giving birth to make labor easier [Chipewyan: 92].  **Roots:** Boiled and used to treat tuberculosis or bathe in to relieve soreness [Dene: 17]. Decoction taken as a cough medicine [Dene: 98]. Herbal tea taken for back paralysis, diabetes and cancer [Cree: 93].  **Stem:** Decoction taken as a cough medicine [Dene: 98]. Boiled and steam inhaled to relieve headache or sore chest [Chipewyan: 92].  **Twigs:** Herbal tea taken for general ache and heart problems [Cree: 93].  **Fruits:** Decoction taken as a cough medicine [Dene: 98].  **Stem and roots:** Boiled and taken to treat tuberculosis [Dene: 98]. |
| *Sorbus* *sitchensis* M. Roem.  Rosaceae, Shrub | Sitka mountain-ash, western mountain-ash (Eng.); sorbier de Sitka (Fr.) | **Roots:** Boiled and used to treat tuberculosis or bathe in to relieve soreness [Dene: 17]. |
| Sphagnum capillifolium (Ehrh.) Hedw.  Sphagnaceae, Moss | Moss (Eng.); askīya (Cree) | Used to treat diaper rash, and to wash baby at birth [Cree: 95]. Used to treat diaper rash, toothache, urinary tract problems. Ssteam generated by putting moss in the heated stones used to treat body pain, muscle pain, to bring out fever [Cree: 80, 81]. |
| *Sphagnum fuscum* (Schimp.) Klinggr.  Sphagnaceae, Moss | peat moss (Eng.); sphaigne brune (Fr.); uske, muskak, askīyāh, mīkaskwahkawow, āsāskumkwa, eskiya, awasistche(Cree) | **Whole plant:** Applied to cuts or skin infections before bandaging affected areas [Cree: 13]. Used to treat diaper rash, and as a baby wash at birth [Cree: 95]. Antiseptic [Dene: 17]. Used to treat diabetes [Cree: 82]. |
| *Spiraea* *alba* Du Roi  Rosaceae, Shrub | white meadow-sweet (Eng.); spirée blanche (Fr.) | **Leaves and stem:** Used to make a medicinal tea [Algonquin: 69]. |
| *Spiraea tomentosa* L.  Rosaceae, Shrub | hardhack, steeple-bush (Eng.); spirée tomenteuse, thé du Canada (Fr.); memîsgwû'nagûg (Ojibwa) | **Leaves and stem:** Used to make a medicinal tea [Algonquin: 69].  **Leaves and flowers:** Tea given for pregnancy sickness and to ease labor during childbirth [Ojibwa: 87]. |
| *Sporobolus* *heterolepis* (A. Gray) A. Gray  Poaceae, Herb | prairie dropseed (Eng.); sporobole à glumes inégales (Fr.); napŏ'gûshkū^n^s' (Ojibwa) | **Roots:** Crushed by pounding or chewing and applied as a poultice to sores. Boiled and the decoction taken to induce emesis, "to remove bile" [Ojibwa: 84]. |
| **Stachys palustris* L.  Lamiaceae, Herb | woundwort (Eng.); épiaire des marais (Fr.); ande'gobûg (Ojibwa) | **Leaves:** Fresh or dried and used to make an infusion taken to treat colic [Ojibwa: 47]. |
| **Stellaria* *media* (L.) Vill.  Caryophyllaceae, Herb | common chickweed (Eng.); stellaire moyenne, mouron des oiseaux (Fr.); wi'nibĭdja'bibaga'no (Ojibwa) | **Leaves:** Infusion used as a wash for sore eyes [Ojibwa: 47]. |
| *Stereocaulon* *paschale* (L.) Hoffm.  Stereocaulaceae, Lichen | snow lichen (Eng.) | Used to treat diabetes [Cree: 82]. |
| *Streptopus amplexifolius* L. (DC.)  Liliaceae, Herb | solomon's seal, eel berry (Eng.); streptope amplexicaule (Fr.) | **Fruits:** Used for gonorrhea, kidney troubles and for blood spitting [Algonquians: 63].  **Fruits and stem:** Steeped and taken in general sickness [Algonquians: 63]. |
| *Streptopus* *lanceolatus* (Aiton) Reveal var. *roseus* (Michx.) Reveal  **Syn.:** *S. roseus* Michx.  Liliaceae, Herb | twisted-stalk (Eng.); streptope rose, rognons de coq (Fr.); agwĭn'gûsibûg', nanibîte'ode'kîn (Ojibwa) | Used for physic or to make a tea for cough [Ojibwa: 87].  **Flowers:** Steeped to make a medicine to produce a sweat [Algonquians: 63].  **Roots:** Steeped and used as a poultice to treat sty [Ojibwa: 47]. |
| Symphoricarpos albus (L.) S.F. Blake var. albus  **Syn.:** *S. racemosus* Michx.  Caprifoliaceae, Shrub | snowberry, wax berry, wolfberry (Eng.); symphorine blanche, graine d'hiver, graine de loup (Fr.); mahekun menes, mahekunimin, māyikaniminanahtik, mahikanimin (Cree); maĭn'gamûna'tĭg, anîgomiji'mînaga'wûnj (Ojibwa) | **Fruits:** Crushed or boiled to make a wash for sore eyes [Dene: 13].  **Roots and stem:** Decoction used to a treat teething pain and venereal disease [Cree: 95; Dene: 13]. Used as an eye wash and drunk to treat skin rashes or mixed with another plant to make a remedy for venereal deseases [Dene: 13].  **Branches and leaves:** Decoction used as a diuretic [Cree: 13].  **Stem:** Decoction given for kidney problems [Cree: 13].  **Fruits:** Infusion used to treat sore eyes [Cree: 95].  **Whole plant:** Infusion applied externally to treat a skin rash [Cree: 95].  **Roots:** Decoction used as a diuretic. Infusion given after childbirth [Ojibwa: 47]. Decoction taken as a physic, or mixed with *Andropogon* *gerardii* and used to treat stoppage of urine [Ojibwa: 47]. Tea used to clear up the afterbirth, and enable quicker convalescence after childbirth [Ojibwa: 87]. |
| *Symphoricarpos* *occidentalis* Hook.  Caprifoliaceae, Shrub | western snowberry (Eng.); symphorine de l'Ouest (Fr.) | **Leafy stems and berries:** Tea taken in kidney problems [Cree: 96]. |
| **Symphoricarpos* *orbiculatus* Moench  Caprifoliaceae, Shrub | Indian currant (Eng.); symphorine à feuilles rondes (Fr.); gus'sigwaka'mĭsh (Ojibwa) | **Roots:** Cooled decoction made with inner bark of roots applied to sore eyes [Ojibwa: 84]. |
| *Symphyotrichum* *ciliolatum* (Lindl.) A. Löve & D. Löve  **Syn.:** *Aster ciliolatus* Lindl.  Asteraceae, Herb | lindley's aster (Eng.); aster ciliolé (Fr.); mistaskewusk, amowusk (Cree) | **Roots:** Tea used to treat pink eye (conjunctivitis). Ground and applied topically to stop bleeding from cuts [Metis: 13]. |
| Symphyotrichum laeve (L.) A. Löve & D. Löve  **Syn.:** *Aster laevis* L.  Asteraceae, Herb | smooth aster (Eng.); aster lisse (Fr.); mistahisakwiwask (Cree) | **Roots:** Chewed to treat toothache and teething pain. Tea used in fevers and to aid recover after childbirth [Cree: 13]. |
| Symphyotrichum puniceum (L.) A. Löve & D. Löve  **Syn.:** *Aster puniceus* L.  Asteraceae, Herb | purple-stemmed aster (Eng.); aster ponceau (Fr.); mistasakewusk, mistahīsakwīwask, mstahiysāgiywusk, pāwistiko(h)maskīhkīh, bigonbimaskgigiah, pikōnbīmaskīgīah, pikwanpīmāskīgah (Cree); denek'áze'eya(ha)naidíé (Chipewyan) | Mixed with another plant in a decoction drunk to treat short breath [Cree: 13].  **Above-ground parts:** Dried, boiled and decoction drunk repeatedly to treat kidney problems, chills, and cold sweats [Cree: 13]. Used for headaches [Chipewyan: 92].  **Flower:** Dried, decoction used to treat headache [Dene: 13].  **Roots:** Dried, mixed with tobacco and smoked, or powdered and inhaled to treat headache. Used as a heart medicine, a diuretic and emetic tea, and as a medicine for sore kidneys. Mixed with other plants and smoked to treat "insanity". Burned on hot rocks in a sweat lodge to ease breathing [Dene: 13]. Dried and used combined with other plants in a decoction used to relax and aid sleep [Metis: 13], and in a different combination to facilitate childbirth [Cree: 13]. Decoction used in fever, after childbirth, teething sickness, and amenorrhoea. Chewed for toothache. Used in facial paralysis [Cree: 95]. Dried, mixed with tobacco and smoked, or powdered and inhaled to treat headache. Used as a heart medicine [Chipewyan: 92]. Herbal water taken for kidney problems [Cree: 93]. |
| *Symplocarpus foetidus* (L.) Nutt.  Araceae, Herb | skunk cabbage (Eng.); symplocarpe fétide, tabac du diable, chou puant (Fr.); sikag-buk (Chippewa) | Strong medicine [Algonquin: 69]. Smelled to treat headache [Mi'kmaq: 43]. Used with *Juniper* gum in tubercolosis [Mi'kmaq: 43].  **Roots:** Steeped to make a cough medicine [Chippewa: 85], also used for swellings [Abenaki: 67]. |
| **Tanacetum vulgare* L.  Asteraceae, Herb | common tansy (Eng.); tanaisie vulgaire (Fr.); o'ckinigi'kweäni'bĭc , muckiki'wît (Ojibwa) | Antifertility agent [Mi'kmaq: 61] and fever medicine [Ojibwa: 87].  **Leaves:** Dried and made into a tea given in kidney troubles. Steeped and given to prevent pregnancy [Malecite: 65]. Steeped in water with equal amount of leaves of *Nepeta cataria* and given in fever. Decoction taken in amenorrhoea [Ojibwa: 47].  **Roots:** Decoction put into ear for soreness, or used to gargle (or chewed) to treat sore throat [Ojibwa: 47]. |
| **Taraxacum* *officinale* F.H. Wigg.  Asteraceae, Herb | dandelion (Eng.); pissenlit (Fr.); meoskamewuskos (Cree); dado'cabodji'bĭk, wesa'usakwûnek (Ojibwa) | Juice used in cold, cough and skin diseases [55]. Plaster or poultice used for blood purification [Algonquin: 69].  **Leaves, stem and roots:** Hepatic, tonic, diuretic and slightly cholagogue [Cree: 74].  **Leaves and roots:** Taken to promote the flow of bile in liver disease, the milky latex used as a mosquito repellent [Metis: 13].  **Leaves:** Decoction drunk to purify blood, to treat anemia, jaundice and against nervousness [Metis: 13].  **Roots:** Used as a blood medicine [Ojibwa: 86]. Steeped in water with roots of *Cirsium sp*. and taken in confinement [Ojibwa: 47]. Tea given in heart burn [Ojibwa: 87]. Decoction drunk as a diuretic to clean blood stream [Cree: 13]. Boiled and taken in diabetes [Dene: 17]. |
| *Taxus canadensis* Marsh.  Taxaceae, Shrub | Canada yew, ground hemlock (Eng.); if du Canada, buis de sapin (Fr.); ne'bagandag' (Ojibwa); adskewacit (Atikamekw); al'nézité, sagaskôdagw (Abénaki); karkatiwahuk (Innu); tshîtshue ashtshîuâshîshk^u^ (Montagnais) | Medicinal [Innu: 72]. Used with *Lycopodium clavatum* as a brew for weakness and fever [Algonquians: 63]. Boiled and added to whiskey for bowels and internal troubles. Boiled and given with fresh milk to the mother after childbirth [Mi'kmaq: 43]. Used in toothache [Ojibwa: 89] or by women experiencing complications after childbirth [Algonquin: 69].  **Leaves:** Tea used to treat rheumatism, sometimes combined with *Prunus pensylvanica* [Abenaki: 67; Algonquin: 69]. Tea used in fever [Mi'kmaq: 62].  **Twigs:** Steeped to make a tea taken as an antiscorbutic or antipyretic [Mi'kmaq: 60], also used for colds [Algonquians: 63]. Boiled with *Juniperus virginiana* twigs and taken internally to treat rheumatism [Ojibwa: 47]. Brewed in a tea, alone or mixed with *Fraxinus pennsylvanica*, used for stomachache or menstrual disorders [Atikamekw: 73]. Boiled in water, mixed with grease and rolled in a towel to be applied locally to ease headache or eye problems [Montagnais: 71]. |
| *Thalictrum* *dasycarpum* Fisch. & Avé-Lall.  Ranunculaceae, Herb | meadow rue (Eng.); pigamon pourpré (Fr.) | **Roots:** Infusion used to reduce fever [Ojibwa: 87]. |
| *Thaspium* *barbinode* (Michx.) Nutt.  Apiaceae, Herb | bearded meadow parsnip (Eng.); panais à noeuds velus (Fr.); bûsidji'bĭkûgûk (Ojibwa) | **Roots:** Decoction used in colic [Ojibwa: 47]. |
| *Thuja occidentalis* L.  Cupressaceae, Tree | arbor vitae, white cedar (Eng.); thuya, cèdre (Fr.); mascakēs, masīkīsk, māsikīskāsiht (Cree); ka'gsgos (Malecite), masi'ck , mâshtshîshk (Montagnais); gi'jikan'dûg (Ojibwa); kizek, kiskens, kisgens, songup, kizigantic (Algonquin); kishig (Chippewa); malan'dak, môlôdagw (Abenaki) | Boiled and the steam inhaled for several hours under a tent to treat pleurisy [Algonquin: 68]. Used to treat swellings and to make medicinal pillows [67].  **Twigs:** Bruised and steeped to make a sweat drink [Algonquians: 63]. Boiled and used to stimulate synovia production and treat numb articulations [Algonquin: 68]. Burned as a disinfectant to fumigate a house where someone is sick of a contagious disease, such as smallpox [Chippewa: 85]. Used to make a steam bath to treat cold, fever, rheumatism, menstrual disorders and for women after childbirth. [Algonquin: 69]. Crushed and mixed with boiling water, the steam is beneficial for toothache [Algonquin: 69]. Decoction taken for pneumonia, powdered branches are used in various ailments. Used in a decoction or chewed to extract the juice and taken internally to treat urine retention, or a sore bladder [Cree: 95]. Steeped and taken for cough and cold. Tea used in consumption [Malecite: 65]. Mashed, steeped in boiling water and applied for heart pain [Montagnais: 70].  **Leaves:** Made into a poultice for swollen hands or feet [Algonquians: 63; Mi'kmaq: 60]. Fresh, mixed with bear fat and used as an ointment for rheumatism [66]. Tea used for cough, headache and as a blood purifier [Ojibwa: 87]. Crused, mixed with other plants in a decoction used as a wash and drunk to treat a facial paralysis caused by a stroke. Powdered with other plants and mixed with water to make a paste used as a poultice to treat facial paralysis caused by "bad medicine", or to treat general pain [Cree: 13] or rheumatism [Algonquin: 68]. Pulverized, heated, spread in a cloth and pressed on the chest to treat congestion and pain. Same preparation applied to treat skin infections, cuts and abdominal pain [Cree: 80]. Brewed as a wash to treat facial paralysis. Used to treat arthritis and muscular pain [Cree: 96].  **Cones:** Tea used to treat colic [Algonquin: 69].  **Wood:** Used for ear problems [Montagnais: 71]. Rotten wood powdered and used for rashes and skin irritations [Algonquin: 69].  **Charcoal:** Combined with bear gall, pricked into the temples with needles to treat convulsions [Chippewa: 47].  **Bark:** Pounded, mixed with grease and applied to burns [Malecite: 65].  **Gum:** Applied to ease toothache [Malecite: 65], also applied for chest or heart pain [Montagnais: 70]. |
| *Thuja* *plicata* Donn ex D. Don  Cupressaceae, Tree | western red cedar, gian arborvitae (Eng.); thuya géant, cèdre de l'ouest (Fr.) | **Leaves:** Diuretic [Cree: 44].  **Leaves and fruits:** Bruised and used as a headache remedy [Ojibwa: 44]. |
| *Tiarella cordifolia* L.  Saxifragaceae, Herb | false miterwort (Eng.); tiarelle cordifoliée (Fr.); siskwimi'nuk (Malecite) | **Roots:** Steeped and given in diarrhoea [Malecite: 65]. |
| *Tilia americana* L.  Tiliaceae, Tree | basswood, whitewood (Eng.); tilleul d'Amérique, bois blanc (Fr.); wikpi/mus (Malecite) | **Bark:** Used for suppurating wounds [Mi'kmaq: 60].  **Leaves:** Infusion used as an eye wash [Algonquin: 69].  **Roots:** Steeped and taken against worms [Malecite: 65]. |
| Toxicodendron pubescens Mill.  **Syn:** *Rhus toxicodendron* L.  Anacardiaceae, Shrub | poison ivy (Eng.); herbe à puce (Fr.); anîmîki'bûg (Ojibwa) | Used as a poultice for swellings [Ojibwa: 86]. |
| *Trametes suaveolens* (L. Ex Fries) Fries.  Polyporaceae, Fungi | diamond willow fungus (Eng.); tramète parfumée (Fr.); wiy(h)kimāsiygan (Cree) | Dried and powdered, used as part of a compound medicine [Dene: 13]. Dried fungus burned and inhaled as a medicine [Dene: 17].  **Fruting body:** Burned and the smoke inhaled to treat headache, or crumbled into an ear to treat earache [Dene: 13]. |
| *Trientalis* *borealis* Raf. ssp. *borealis*  Primulaceae, Herb | star anemone (Eng.); trientale boréale (Fr.) | Steeped and used for general sickness [Algonquians: 63]. |
| **Trifolium hybridum* L.  Fabaceae, Herb | alsike clover (Eng.); trèfle hybride, trèfle Alsike (Fr.); moostos mechewin (Cree); ligàz'à (Dene) | **Leaves:** Tea drunk as a blood purifier/thinner or to treat eczema or psoriasis [Metis: 13].  **Berries:** Tea drunk to cure respiratory problems [Dene: 101]. |
| **Trifolium pratense* L.  Fabaceae, Herb | red clover (Eng.); trèfle des prés, trèfle rouge (Fr.) | Tea used for whooping cough [Algonquin: 69]. |
| *Triglochin* *maritima* L.  Juncaginaceae, Herb | seaside arrow-grass (Eng.); troscart maritime, faux jonc (Fr.); minahikos (Cree) | **Whole plant:** Boiled and the decoction drunk to relieve bloody diarrhoea [Cree: 13]. |
| *Trillium erectum* L.  Liliaceae, Herb | bethroot, red trillium (Eng.); trille dressé, trille rouge (Fr.); d^z^id^z^iz (Abenaki) | **Rhizomes and roots:** Used for "women complaints" [44] and various child diseases [Abenaki: 67]. Freshly cut, and the acridity inhaled for nostril bleeding [44]. |
| *Trillium* *grandiflorum* (Michx.) Salisb.  Liliaceae, Herb | white trillium (Eng.); trille blanc, trille grandiflore (Fr.); inĭ'nĭwĭn'dĭbĭge'gûn (Ojibwa) | **Roots:** Infusion of inner bark of root dropped in the ear to treat soreness. Decoction taken in rheumatism [Ojibwa: 47]. |
| *Trillium undulatum* Willd.  Liliaceae, Herb | painted trillium (Eng.); trille ondulé (Fr.); penadamabaskw (Atikamekw) | **Flowers and leaves:** Crushed and the pulp eaten to accelerate childbirth [Atikamekw: 73]. |
| *Triosteum* *perfoliatum* L.  Caprifoliaceae, Herb | horse gentian (Eng.); trioste orangé (Fr.); moni^n^swa^n^ (Ojibwa) | **Roots:** Decoction used for urinary pain, as a diuretic, or as a laxative [Ojibwa: 19]. |
| *Tsuga canadensis* (L.) Carrière  Pinaceae, Tree | eastern hemlock (Eng.); prûche (Fr.); ksiusk (Malecite); gaga'gimĭc (Ojibwa); al'nézité (Abenaki) | Tea used for cold, boiled and applied externally for eczema and other skin infections. Used in the sudatory for women experiencing complications in childbirth [Algonquin: 69].  **Bark:** Tea used in cold, cough and grippe [Mi'kmaq: 43, 60]. Used to heal cuts and wounds [Ojibwa: 87, 91]. Tea used to relieve cold [Mi'kmaq: 62]. Mixed with buds or young cones of *Abies balsamea*, and bark of *Quercus rubra* and used for diarrhoea [Malecite: 65].  **Leaves:** Used for rheumatism, itching or scabies [Abenaki: 67].  **Roots:** Steeped with *Conioselium chinense, Chimaphila umbellate,* and *Rumex crispus* and given in bladder problems [Mi'kmaq: 62].  **Twigs:** Steeped to make a drink as a remedy for dysentery [Ojibwa: 85]. Tea, thick syrup or paste used as a poultice for arthritis. Crushed to make a poultice for the infected navel of an infant [Algonquin: 69].  **Inner bark:** Powdered and applied to wounds to stop bleeding [Ojibwa: 47]. Tea used for pain and cold [Mi'kmaq: 91].  **Wood:** Dried and used in prickly heat or chafed skin [Malecite: 65]. |
| *Typha angustifolia* L.  Typhaceae*,* Herb | cat-tail flag (Eng.); quenouille (Fr.); ba'ziask (Malecite) | **Roots:** Steeped and taken in gravel [Malecite: 65]. |
| *Typha latifolia* L.  Typhaceae, Herb | cattail (Eng.); massette, quenouille (Fr.); otawuskwa, ōtawaskwa, ā(h)towusk, wahōtāhuk, pāsīhkan (Cree); segidebigakde'gil (Malecite); tl'okàwhi (Dene); tlh'oghk'a (Chipewyan) | **Seeds:** Dried and used as a poultice for burns [Cree, Metis: 13]. Poultice used in skin disorders, cuts, burns and bee stings [Cree: 96].  **Leaves:** Greased and layed on a sore twice a day [Malecite: 65].  **Roots:** Used in diabetes [Cree: 83]. Crushed by pounding or chewing and applied as a poultice to sores [Ojibwa: 84]. Poultice applied to wounds and infections [Algonquin: 69]. Boiled and used to wash skin infections. Compress applied to treat sore throat [Cree: 80]. Medicinal [Chipewyan: 92].  **Stalk:** Burned and the ashes applied to a skin rash, towel soaked in tea and applied to cure urinary tract problems [Dene: 101]. |
| *Ulmus americana* L.  Ulmaceae, Tree | American elm (Eng.); orme d'Amérique (Fr.) | **Bark:** Steeped and drunk as a cure for lung bleeding [Algonquians: 63]. |
| *Ulmus* *rubra* Muhl.  **Syn.:** *U. fulva* Michx.  Ulmaceae, Tree | slippery elm (Eng.); orme rouge (Fr.) ah-nib, gawa'komĭc (Ojibwa) | Used in gonorrhoea [Ojibwa: 86].  **Bark:** Used for lung bleeding. Used to soothe irritated throat and lungs. Used for suppurating wounds [Mi'kmaq: 60]. Decoction used for infected kidneys or as a gargle to treat ulcerated throat [Ojibwa: 47, 91].  **Inner bark:** Chewed fresh for cough [44]. Used for sore throat [Ojibwa: 87].  **Roots:** Chewed dry to treat sore throat [Ojibwa: 47]. |
| *Umbilicaria* *mammulata* (Ach.) Tuck.  **Syn.:** *Gyrophora* *dillenii* (Tuck.) Müll. Arg.  Umbilicariaceae, Lichen | lichen (Eng.); tripe de roche (Fr.); asinewakunik (Atikamekw) | Softened in boiling water and applied to stomach of women having difficulty giving birth [Atikamekw: 73]. |
| *Umbilicaria* *muehlenbergii* (Ach.) Tuck.  **Syn.:** Actinogyra muehlenbergii (Ach.) Schol.  Umbilicariaceae, Lichen | rock tripe (Eng.); thets'í^n^. (Chipewyan); asinīwāhkona, wakoonak, asinīwākon (Cree); kwechį (Dene) | Chewed or dried, powdered, and boiled to make asyrup taken against tapeworms [Dene: 13; Chipewyan: 92]. Decoction used in stomachache [Cree: 13]. Soup eaten as a tonic and for breathing problems [Dene: 101]. |
| *Urtica* *dioica* L. ssp. *gracilis* (Aiton) Seland.  **Syn.:** *U. lyallii* S. Watson*; U. procera* Muhl. ex Willd  Urticaceae, Herb | nettle (Eng.); ortie élevée (Fr.); masan, ma'zana'tĭg, (Ojibwa); katsékô'sag (Abenaki) | Used for nosebleed [Abenaki: 67].  **Roots:** Decoction, alone or with *Athyrium filix-femina*, used to treat stoppage of urine. Steeped and taken in dysentry [Chippewa: 47]. Used as a wash in skin disorders, cuts, burns and bee stings [Cree: 96]. Herbal water taken for back paralysis, fever and urinary tract problems [Cree: 93].  **Leaves:** Soaked in warm water and used as a poultice for heat rashes [Ojibwa: 87].  **Leaves and stems:** Herbal water taken for anemia [Cree: 93]. |
| *Urtica dioica* L*.*  Urticaceae, Herb | stinging nettle (Eng.); ortie (Fr.); masān, musān, musanusk, assan, masānah (Cree) | Decoction used to keep blood flowing after childbirth [Cree: 95].  **Leaves:** Decoction used as a face wash to treat acne [Cree: 13] and drunk to treat diarrhoea or intestinal worms [Dene: 13].  **Roots:** Decoction used as a wash to relieve itching and inflammation caused by touching stinging leaves [Cree: 13], drunk as a blood purifier, to stop internal bleeding, to correct menstrual flow, or to clear phlegm from the lungs and throat [Dene: 13]. Boiled and the steam inhaled to treat asthma [Cree: 13]. |
| *Usnea hirta* (L.) F. H. Wigg.  Parmeliaceae, Lichen | miyapakwan, mithapakonuk (Cree) | Used to pack nostril to stop nosebleed [Dene: 13] |
| *Usnea* sp.  Parmeliaceae, Lichen | old man's beard moss (Eng.); mithāpākwan (Cree) | Inserted fresh into the nostril to stop nosebleed [Cree: 95]. |
| *Utricularia* *vulgaris* L.  Lentibulariaceae, Herb | bladderwort (Eng.); utriculaire commune (Fr.) | **Whole plant:** Tea used to treat kidney or bladder infection [Dene: 100]. Boiled and washed to treat sore legs [Dene: 98].  **Leaves:** Tea used to treat kidney or bladder infection [Dene: 100]. |
| *Uvularia grandiflora* Sm.  Liliaceae, Herb | large flowered bellwort (Eng.); uvulaire grandiflore (Fr.); wesawabi'kwonêk (Ojibwa) | **Roots:** Used for stomach troubles [Ojibwa: 87]. |
| *Vaccinium angustifolium* Aiton  **Syn.:** *V.pennsylvanicum* Lam.  Ericaceae, Shrub | late sweet blueberry, low-bush blueberry (Eng); airelle à feuilles étroites, bleuet (Fr.); mînûga'wunj, mĭna'aga'wûnj (Ojibwa) | Used to treat "crazyness" [Ojibwa: 47].  **Leaves:** Tea used as a blood purifier [Ojibwa: 87], to treat colic and given after miscarriage [Algonquin: 69].  **Roots:** Tea given to induce labor during childbirth [Algonquin: 69]. Used to treat urinary problems [Algonquin: 68]. Used in diabetes [Cree: 82, 83].  **Fruits:** Used in diabetes [Cree: 82, 83]. |
| *Vaccinium* *macrocarpon* Aiton  **Syn.:** Oxycoccus macrocarpus (Aiton) Pursh  Ericaceae, Shrub | cranberry (Eng.); airelle à gros fruits, gros atocas (Fr.) | **Twigs:** Steeped and used as a medicine for pleurisy [Algonquians: 63]. |
| *Vaccinium myrtilloides* Michx.  Ericaceae, Shrub | blueberry, bilberry, whortle berry, huckleberry (Eng.); airelle fausse-myrtille, bleuet (Fr.); inimena, īyinimin, iynimin, ithīnīmina, sīpīkōmin (Cree); ts'a^n^lhchoth (Chipewyan) | **Whole plant:** Component of an anticancer medicine [Metis: 13].  **Fruits:** Helps to reduce acne. Syrup used to stop vomiting [Metis: 13].  **Stem:** Boiled to make a drink taken to prevent pregnancy or in combination with other plants to prevent miscarriage, bring blood after childbirth, trigger menstruation, or slow excessive menstruation [Metis, Cree: 13].  **Leaves:** Decoction taken for headache [Dene: 13]. Steeped and applied for rheumatism [Mi'kmaq: 62].  **Roots:** Tea drunk for headache [Chhipewyan: 92]. Herbal water used to ease childbirth [Cree: 93]. |
| *Vaccinium oxycoccus* L.  Ericaceae, Shrub | cranberry (Eng.); canneberge, atocas (Fr.); mûcki'mûnj (Ojibwa) | Tea drunk to treat nausea [Ojibwa: 87]. |
| *Vaccinium* *uliginosum* L.  Ericaceae, Shrub | alpine bilberry, bog blueberry (Eng.); airelle des marécages (Fr.); jàk zheii (Dene) | **Stem and leaves:** Boiled to make a tea for cold [Dene: 99].  **Berries:** Eaten raw or made into a tea taken in diabetes or in heart problems [Dene: 100]. |
| *Vaccinium vitis-idaea* L.  Ericaceae, Shrub | bog cranberry, cowberry, lingonberry (Eng.); airelle vigne d'Ida, berris, graines rouges (Fr.); wesakemina, wīsaki(h)min, wiysukiymin (Cree); uîshatshiminânakashî (Montagnais); natl'at (Dene) ; na^n^tlh'ër (Chipewyan) | **Fruits:** Used for stomach cleansing [Cree: 13], fever [Dene: 13; Chipewyan: 92], diabetes [Cree: 82, 83], mouth infections in children, and throat pain [Montagnais: 71]. Cooked to make a jam used for diarrhoea. Juice used to dampen contractions during childbirth [Montagnais: 71]. Juice taken for kidney problems, cough and cold, indigestion, and to improve appetite [Dene: 99]. Chewed or boiled to ease cough and cold [Dene: 101].  **Roots and stems:** Boiled and the decoction drunk to treat bladder problems [Cree: 13].  **Whole plant and fruits:** Tea drunk to treat kidney or urinary tract problems [Dene: 100]. |
| *Valeriana dioica* L.  Valerianaceae, Herb | northern valerian (Eng.); valériane (Fr.); upistagiwasus, apiscisakōwaskwos, apiscakāwaskwos, apisichīsakwasosuk, apiscīsakwīwaskwos, āpisagiywuskos (Cree) | **Whole plant:** Dried, boiled and the decoction drunk to treat a sore aching body, cold, chill, and congestion, helping clear the air passages [Cree: 13].  **Leaves:** Decoction given to a child who has lost weight to help gain weight [Cree: 13]. Decoction given to prevent miscarriage or to ease labor pain [Cree: 13]. Dried, crushed, mixed with beaver fat and applied as an ointment on facial rashes [Cree: 13].  **Stem and leaves:** Boiled with another plant and drunk to treat cold or fever [Cree: 13].  **Roots:** Decoction from a plant that has not flowered is considered a very powerful tonic. If abused it will make user very sick [Cree: 13]. Chewed to treat severe heart trouble [Cree, Metis: 13], also helps to prevent aging and wrinkles and keep active [Cree: 13]. |
| *Valeriana* *dioica* L. var. *sylvatica* S. Watson  **Syn.:** *V. sylvatica* Sol. ex Richardson, non F.W. Schmidt V. septentrionalis Rydb.  Valerianaceae, Herb | northern valerian, wood valerian (Eng.); valériane nordique (Fr.); apiscīsakwīwaskwos (Cree) | **Roots:** Chewed, put into a cloth and placed in the ear to treat earache, and rubbed on the head and temples to treat headache. Applied externally in case of seizure. Powdered, used as an ingredient for many ailments and to treat menstrual troubles. Infusion used for all purposes. Decoction used for pneumonia and in a smoking mixture for colds [Cree: 95]. Chewed or prepared as a tea for heart problems. Tea taken as a tonic and blood cleanser [Cree: 96]. |
| *Veratrum viride* Aiton  **Syn.:** V. eschscholtzii A. Gray  Liliaceae, Herb | false or indian hellebore (Eng.); vérâtre vert, tabac du diable, hellébore (Fr.) | **Roots:** Febrifugal, depressant and stirnutatory [Cree: 74]. Used as an emetic [Dene: 98]. |
| **Verbascum thapsus* L.  Scrophulariaceae, Herb | mullein (Eng.); molène vulgaire, tabac du diable (Fr.); tcuglsia'sihonel (Malecite); mandando'hibag (Abenaki) | Necklace for teething babies [Abenaki: 67].  **Leaves:** Powdered and smoked to cure asthma [Algonquians: 63]. Applied to sores, cuts, and catarrh [Malecite: 65]. Steeped and smelled to relieve asthma [Mi'kmaq: 62].  **Roots:** Used as an expectorant [66]. |
| *Verbena* *hastata* L.  Verbenaceae, Herb | blue vervain (Eng.); verveine hastée (Fr.) | **Flowers:** Dried and snuffed to stop nostril bleeding [Ojibwa: 47]. |
| *Veronicastrum* *virginicum* (L.) Farw.  **Syn.:** *Leptandra* *virginica* (L.) Nutt. *V. virginica* L.  Scrophulariaceae, Herb | culver's root (Eng.); véronique de Virginie (Fr.); wi'sŏgedzhi'wik, wi'sûgidji'bĭk (Ojibwa) | **Roots:** Decoction taken as a purgative [Ojibwa: 44, 84] and a physic [Ojibwa: 47]. Decoction with bark of *Prunus virginiana* taken before breakfast to treat scrofula [Ojibwa: 47]. |
| *Viburnum acerifolium* L.  Caprifoliaceae, Shrub | mapleleaf viburnum (Eng.); viorne à feuilles d'érable (Fr.); anib' (Ojibwa) | **Inner bark:** Decoction to treat stomach cramps. Decoction, alone or with *Almus incana*, used as an emetic [Ojibwa: 47]. |
| *Viburnum* *edule* (Michx.) Raf.  **Syn.:** *V. pauciflorum* La Pylaie ex Torr. & A. Gray  Caprifoliaceae, Shrub | pembina, squashberry, mooseberry, low bush-cranberry, (Eng.); viorne comestible, pimbina (Fr.); moosomina, mōsomina, moosominahtik, mōsōminā(h)tik (Cree); deníjíé (Chipewyan) | Ingredient in a gargle for sore throat [Cree: 95]. Tea used as a blood purifier [Metis: 13].  **Twig:** Tips chewed to treat sore throat [Dene: 13, Cree: 95].  **Buds:** Unopened buds rubbed on sore lips to heal and dry [Cree: 95; Dene: 13].  **Roots:** Tea used to ease teething pain [Dene: 13, Cree: 95] and as a gargle for sore throat [Dene: 13]. Boiled to make an external wash or bath for sore body parts [Dene: 17]. Medicinal [Chipewyan: 92].  **Leaves and stem:** Infusion taken for sore throat [Cree: 95].  **Fruits:** Eaten to clean the blood. Boiled and drunk for cold [Dene: 17] or to cure urinary tract problems and constipation [Dene: 101]. Boiled and taken as a cough medicine [Dene: 98].  **Leaves:** Crushed and applied to bee stings and burns [Dene: 100]. |
| Viburnum lantanoides Michx.  **Syn.:** *V. alnifolium* Marsh.  Caprifoliaceae, Shrub | hobblebush (Eng.); viorne à feuilles d'aulne, bois d'orignal (Fr.); mistatiteminatuk (Atikamekw) | **Leaves:** Crushed and rubbed on head to fight headache [Atikamekw: 73]. |
| *Viburnum lentago* L.  Caprifoliaceae, Shrub | sheepberry, nannyberry, (Eng.); viorne lentago, alisier, bourdaine (Fr.); teta-minan, atîte' (Ojibwa); | **Leaves:** Steeped and drunk as a remedy for dysuria. Poultice bound on the abdomen, over the bladder [Ojibwa: 85].  **Roots:** Steeped and taken to trigger menstruation, sometimes mixed with Canada lily [Malecite: 65].  **Inner bark:** Tea used as a diuretic [Ojibwa: 87]. |
| *Viburnum opulus* L.  Caprifoliaceae, Shrub | cranberries, flat seed berries, moose berry (Eng.); viorne obier (Fr.) | **Fruits:** Steeped and drunk for swollen glands [Mi'kmaq: 60, Algonquians: 63] and mumps [Algonquians: 63]. Boiled and the mess rubbed in the eyes for sore eyes [Algonquians: 63].  **Bark:** Decoction used as a diuretic [66]. |
| *Viburnum* *opulus* L. var. *americanum* Aiton  Caprifoliaceae, Shrub | Bush cran berry (Eng.); viorne trilobée (Fr.); nepiminana, nīpiminān (Cree); a'nibîmî'nûga'wûck (Ojibwa) | **Roots:** Steeped and drunk as a remedy for pralapsus uteri. Part of the treatment consists of soaking a white cloth in an infusion and placing it in the vagina to put the uterus back into place [Ojibwa: 85].  **Bark:** Tea used as a diuretic, given to mothers after birth to prevent infection [Dene: 13], also given to treat insomnia [Dene, Metis: 13].  **Inner bark:** Tea used as a physic and to cure stomach cramps [Ojibwa: 87]. |
| *Viburnum prunifolium* L.  **Syn.:** *V. pomifolium*  Caprifoliaceae Shrub | stag-bush sloe (Eng.); viorne à feuilles de cerisier (Fr.) | Tea given to women before and after parturition [Mi'kmaq: 43]. |
| *Viola* *bicolor* Pursh  Violaceae, Herb | field pansy (Eng.); violette de Rafinesque (Fr.) | **Whole plant:** Infusion used to treat blood-shot eyes [Mi'kmaq: 62]. |
| *Viola canadensis* L.  Violaceae, Herb | Canada violet (Eng.); violette du Canada (Fr.); maskwī'widzhī'wiko'kŏk (Ojibwa) | **Roots:** Decoction taken for bladder pain [Ojibwa: 66, 84]. |
| *Viola* *labradorica* Schrank  **Syn.:** *V. conspersa* Rchb.  Violaceae, Herb | American dog violet, alpine violet (Eng.); violette du Labrador (Fr.); wewaîe'bûgûg (Ojibwa) | **Whole plant:** Tea given for heart trouble [Ojibwa: 87]. |
| *Viola pubescens* Aiton  Violaceae, Herb | downy yellow violet (Eng.); violette pubescente (Fr.); ogitē'wagu^n^s (Ojibwa) | **Roots:** Decoction taken in sore throat [Ojibwa: 66, 84]. |
| *Vitis* sp.  Vitaceae, Vine | wild grape (Eng.); vigne sauvage (Fr.); šiwi-min (Ojibwa) | **Roots and branches:** Steeped with roots of *Ceanothus* and *Hepatica*, bark of beech, and inner bark of sugar maple, black birch, and red-osier dogwood to make a remedy for pulmonary trouble [Ojibwa: 85].  **Sap:** Used as a tonic to wash hair [Ojibwa: 85]. |
| *Vitis* *vulpina* L**.**  **Syn.:** *V. cordifolia* Michx.  Vitaceae, Vine | frost grape, river-bank grape (Eng.); vigne des renards (Fr.); ci'wî mînûn, jo'mĭnaga'wûnj (Ojibwa) | **Twigs:** Tea used to clear up afterbirth and enable it to pass easily [Ojibwa: 87].  **Sap:** Used for stomach and bowel trouble [Ojibwa: 87].  **Roots:** Steeped and taken to treat rheumatism and diabetes [Ojibwa: 47]. |
| *Zanthoxylum americanum* Mill.  Rutaceae, Shrub | prickly ash (Eng.), clavalier d'Amérique, frêne épineux (Fr.); dagawak-mins, gawa'komĭc (Ojibwa) | **Bark:** Hot drink together with wild ginger root, bark of sassafras roots, and rhizomes of sweet flag, taken for cold, cough, and pulmonary troubles [Ojibwa: 85]. Tea drunk for worms [Mi'kmaq: 62]. Used to treat quinsy and sore throat [Ojibwa: 87].  **Roots:** Decoction used to treat sore throat [Ojibwa: 47].  **Fruits:** Tea drunk to treat sore throat, and as a spray on the chest to cool and relieve congestion in bronchitis [Ojibwa: 87]. |
| *Zizania aquatica* L.  Poaceae, Herb | wildrice (Eng.); zizanie aquatique, folle avoine, riz sauvage (Fr.); manomin (Ojibwa) | **Roots:** Used for urinary infection and diabetes [Ojibwa: 89]. |

*Introduced species.
