# Supplementary material for: Traditional use of medicinal plants in the boreal forest of Canada: review and perspectives
Source: J Ethnobiol Ethnomed. 2012 Jan 30;8:7. doi: 10.1186/1746-4269-8-7 (PMC3316145; doi:10.1186/1746-4269-8-7)
Supplement: Additional file 2 — Major disease categories and associated medicinal plants used by the Aboriginal people of boreal Canada. [file 1746-4269-8-7-S2.DOC]

**Additional file 2: Major disease categories and associated medicinal plants used by Aboriginal people of boreal Canada**

| ***Disease categories*** | ***Plants*** |
| --- | --- |
| Blood system disorders (purification, anaemia, coagulation, etc.) | *Alnus* sp*., Aralia nudicaulis, Arctium lappa, Arctostaphylos* *alpina,* Chamerion angustifolium ssp. angustifolium, *Chimaphila* *umbellata, Fragaria* *vesca* ssp. *bracteata, Gaultheria procumbens,* Heracleum maximum, *Larix* *laricina,* Melilotus officinalis, *Mentha arvensis, Nepeta cataria, Nuphar* *lutea* ssp. *polysepala, Oplopanax* *horridus, Pedicularis canadensis,* Picea glauca, *Picea* *mariana*, *Polygala senega, Populus alba, Populus balsamifera*, *Populus* *grandidentata, Populus tremuloides*, *Prunus pensylvanica, Prunus virginiana*, *Rhododendron groenlandicum, Rhus glabra, Rhus typhina, Salix* *nigra, Salix* sp.*, Sium suave, Sorbus americana, Taraxacum* *officinale, Thuja occidentalis*, *Trifolium hybridum, Urtica dioica, Urtica* *dioica* ssp. *gracilis,* *Valeriana* *dioica* var. *sylvatica*, *Viburnum* *edule* |
| Circulatory system disorders (heart problems, blood pressure, cholesterol, haemorrhoids, etc.) | *Abies balsamea, Acorus* *americanus*, *Acorus calamus, Alisma plantago-aquatica, Apocynum androsaemifolium, Aralia hispida, Aralia nudicaulis, Artemisia* *dracunculus, Betula papyrifera, Campanula rotundifolia,* Cardamine diphylla*,* Clintonia borealis, *Coptis* *trifolia, Corylus cornuta*, *Dalea purpurea*, *Dryopteris* sp., *Fragaria* *vesca* ssp. *bracteata*, Gaultheria hispidula, *Gaultheria procumbens*, *Inula helenium*, *Larix* *laricina, Lilium philadelphicum, Lonicera dioica, Matteuccia struthiopteris, Mentha arvensis, Mertensia* *paniculata, Nuphar lutea, Nuphar* *lutea* ssp. *polysepala, Picea* *mariana, Plantago major, Polygala senega, Polypodium virginianum, Populus balsamifera*, *Populus tremuloides, Prunus pensylvanica, Prunus* sp.*, Prunus virginiana*, *Quercus* *macrocarpa*, *Quercus rubra*, *Rubus idaeus, Rumex aquaticus, Sanguinaria canadensis, Scutellaria galericulata, Shepherdia canadensis, Sium suave, Solidago canadensis*, *Sorbus americana*, Symphyotrichum puniceum*, Thuja occidentalis, Vaccinium* *uliginosum*, *Valeriana dioica, Valeriana* *dioica* var. *sylvatica, Viola* *labradorica* |
| Cough, cold and sore throat | *Acer* *saccharum, Achillea millefolium*, *Achillea millefolium* ssp. *lanulosa* var. *lanulosa,* *Achillea sibirica, Acorus* *americanus*, *Acorus calamus*, *Agastache foeniculum*, *Allium stellatum, Alnus* *viridis* ssp. *crispa, Amelanchier alnifolia*, *Amelanchier* *bartramiana*, Anaphalis margaritacea*, Anemone canadensis, Aralia hispida, Aralia nudicaulis, Aralia racemosa, Arctium minus*, *Arctostaphylos uva-ursi, Artemisia campestris, Artemisia frigida*, *Artemisia* sp., *Artemisia* *tilesii*, *Asarum canadense, Asclepias tuberosa, Berberis vulgaris, Betula papyrifera, Caltha palustris, Campanula rotundifolia,* Cardamine diphylla, *Carum carvi,* Chamerion angustifolium ssp. angustifolium*, Chenopodium album, Coptis* *trifolia, Cornus alternifolia, Cornus canadensis*, *Cornus florida, Cornus sericea, Empetrum nigrum, Equisetum arvense, Erigeron* *philadelphicus*, *Eupatorium perfoliatum, Eupatorium purpureum, Gaultheria procumbens, Geum* *aleppicum, Geum rivale*, *Gnaphalium* sp., Heracleum maximum*, Hypericum perforatum, Iris versicolor, Juniperus communis, Juniperus horizontalis, Kalmia angustifolia, Larix* *laricina, Lilium philadelphicum, Maianthemum canadense, Mentha aquatica, Mentha arvensis, Monarda fistulosa, Myrica gale, Nuphar lutea, Nuphar* *lutea* ssp. *advena, Nymphaea* *odorata*, *Oplopanax* *horridus*, *Osmorhiza* *claytonii,* Osmorhiza longistylis, *Ostrya* *virginiana, Penstemon* *confertus*, *Phryma leptostachya,* Picea glauca*, Picea* *mariana, Picea* *rubens, Picea* *sitchensis, Pinus banksiana, Pinus* *contorta, Pinus* sp., *Pinus strobus, Platanthera* *dilatata* var. *leucostachys, Polygala* *polygama, Polygala senega, Polygonatum* *biflorum*, *Polyporus resinosus*, *Populus* sp., *Populus tremuloides,* Potentilla norvegica ssp. monspeliensis, *Prunella vulgaris, Prunus nigra, Prunus pensylvanica, Prunus serotina,* *Prunus virginiana, Rhexia virginica, Rhododendron* *groenlandicum, Rhododendron tomentosum, Rhus glabra, Rhus typhina,* Ribes hudsonianum*, Ribes lacustre, Rosa acicularis, Rosa* *woodsii*, *Rubus allegheniensis*, *Rubus chamaemorus, Salix* *candida, Salix discolor, Salix* sp., *Sanguinaria canadensis, Sanguisorba canadensis, Sarracenia purpurea, Sassafras albidum, Schoenoplectus* *acutus, Scirpus* *microcarpus, Shepherdia canadensis, Solidago canadensis, Solidago* *flexicaulis*, *Sorbus americana, Sorbus* *scopulina*, *Streptopus* *lanceolatus*, *Symplocarpus foetidus, Tanacetum vulgare, Taraxacum* *officinale*, *Taxus canadensis, Thuja occidentalis*, *Trifolium pratense, Tsuga canadensis, Ulmus* *rubra, Vaccinium* *uliginosum*, *Vaccinium vitis-idaea, Valeriana dioica, Valeriana* *dioica* var. *sylvatica, Viburnum* *edule, Viola pubescens, Zanthoxylum americanum* |
| Dermatological infections (boils, eczema, felons, itch, measles, running sore, scurvy, dropsy, irritant, small pox, chicken pox, skin problems, body wash, and used as baby powder, etc.) | *Abies balsamea, Acer saccharinum, Acer spicatum, Achillea millefolium, Achillea millefolium* ssp. *lanulosa* var. *lanulosa*, *Acorus* *americanus*, *Acorus calamus, Alnus* sp.*, Alnus* *viridis* ssp. *crispa,* Anaphalis margaritacea, *Anemone canadensis, Aralia nudicaulis, Aralia racemosa, Arisaema triphyllum, Arnica* *angustifolia*, *Artemisia dracunculus, Artemisia frigida, Baptisia tinctoria, Betula papyrifera, Betula pubescens*, *Carum carvi, Caulophyllum thalictroides, Celastrus scandens,* Chamerion angustifolium ssp. angustifolium*, Chimaphila umbellata, Cirsium arvense*, Clintonia borealis*, Comptonia peregrina, Coptis* *trifolia*, *Cornus sericea, Cypripedium acaule*, *Dryopteris carthusiana, Empetrum nigrum, Equisetum arvense, Equisetum sylvaticum, Erysimum cheiranthoides, Galium aparine, Galium* *trifidum*, *Hamamelis virginiana*, *Heracleum* maximum, *Hylotelephium telephium,* Impatiens capensis, *Impatiens pallida, Juniperus communis, Lactuca canadensis, Larix laricina, Lycoperdon perlatum, Lycoperdon* sp., *Maianthemum racemosum* ssp*. racemosum, Myrica gale, Nuphar lutea*, *Osmorhiza* *claytonii*, *Petasites frigidus* var*. sagittatus*, Picea glauca*, Picea mariana, Picea* sp., *Picea sitchensis, Pinus banksiana, Pinus strobus, Plantago major, Platanthera orbiculata,* *Polygonatum biflorum* var*. commutatum, Polygonum hydropiper, Populus balsamifera, Populus* sp., *Populus tremuloides, Prunus pensylvanica, Prunus serotina*, *Prunus* sp., *Pyrola* *asarifolia* ssp. *asarifolia, Rhododendron groenlandicum, Ribes* *triste*, *Rosa blanda, Rubus chamaemorus, Rubus idaeus, Rubus idaeus* ssp*. strigosus, Rumex aquaticus, Rumex* *arcticus*, *Rumex crispus, Sagittaria cuneata, Salix discolor, Sanguinaria canadensis, Sarracenia purpurea, Scirpus microcarpus*, *Shepherdia canadensis, Solidago* *altissima*, *Sorbus americana*, Sphagnum capillifolium*, Sphagnum fuscum, Stellaria media*, *Taraxacum officinale, Taxus canadensis, Thuja occidentalis, Trifolium hybridum, Tsuga canadensis, Urtica dioica, Urtica* *dioica* ssp. *gracilis, Vaccinium myrtilloides, Valeriana dioica, Verbascum thapsus* |
| Diabetes | *Abies balsamea, Achillea millefolium*, *Acorus* *americanus, Acorus calamus, Andromeda* *polifolia* var. *latifolia*, *Aralia racemosa, Arctostaphylos uva-ursi, Cladina* *rangiferina*, *Euphorbia* sp., Gaultheria *hispidula*, *Juniperus communis, Kalmia angustifolia, Larix* *laricina, Lycopodium clavatum*, Lysichiton *americanus, Mentha arvensis*, *Nuphar lutea*, *Oplopanax* *horridus*, Picea glauca*, Picea* *mariana, Pinus banksiana, Populus balsamifera, Populus* sp., *Populus tremuloides, Rhododendron* *groenlandicum, Rhododendron tomentosum, Ribes* *americanum*, *Salix* *planifolia, Salix* sp., *Sarracenia purpurea, Shepherdia canadensis, Sorbus* *decora*, *Sorbus* *scopulina*, *Sphagnum fuscum, Stereocaulon* *paschale*, *Taraxacum* *officinale*, *Typha latifolia, Vaccinium angustifolium, Vaccinium* *uliginosum*, *Vaccinium vitis-idaea, Vitis* *vulpina*, *Zizania aquatica* |
| Earache, deafness, ear irritation | *Acorus calamus, Apocynum androsaemifolium, Aralia nudicaulis, Aralia racemosa*, *Campanula rotundifolia, Fraxinus americana, Fraxinus* sp., *Larix* *laricina, Lobelia inflata, Mitella nuda, Nicotiana tabacum, Oclemena* *nemoralis, Picea* *mariana, Plantago major, Rhus glabra, Tanacetum vulgare, Thuja occidentalis*, *Trametes suaveolens*, *Trillium* *grandiflorum*, *Valeriana* *dioica* var. *sylvatica* |
| Fainting and fits | *Adiantum pedatum, Alnus* sp*., Aralia nudicaulis, Arctostaphylos uva-ursi, Aristolochia serpentaria*, *Cornus canadensis, Heracleum* maximum*, Nicotiana tabacum*, *Picea* glauca*, Picea mariana, Pinus banksiana, Pinus strobus, Plantago major*, *Polygonum pensylvanicum, Populus balsamifera, Quercus rubra, Rhinanthus minor, Salix discolor* |
| Fever | *Achillea millefolium, Acorus* *americanus*, *Acorus calamus, Alnus* sp*., Amelanchier alnifolia, Arctium minus, Arctostaphylos uva-ursi, Artemisia absinthium, Artemisia frigida, Asarum canadense, Campanula rotundifolia*, Cardamine diphylla, Comptonia peregrina, *Cornus alternifolia, Cornus florida, Cornus sericea, Dasiphora* *fruticosa* ssp. *floribunda*, *Erigeron* *philadelphicus*, *Eupatorium perfoliatum, Eryngium aquaticum,* Gaultheria hispidula, *Iris versicolor, Juniperus communis, Lilium philadelphicum, Lycopodium clavatum, Lycopodium* sp. *, Mentha arvensis, Mitchella repens, Monarda fistulosa, Nepeta cataria,* Picea glauca, *Populus tremuloides, Prunella vulgaris, Prunus pensylvanica, Prunus virginiana, Pycnanthemum* *virginianum*, *Rhododendron groenlandicum, Rhus typhina, Rosa acicularis, Rubus allegheniensis, Rubus chamaemorus, Rubus idaeus, Sambucus nigra* ssp*. canadensis, Sanicula marilandica, Sarracenia purpurea, Schoenoplectus acutus*, Scutellaria *galericulata, Shepherdia canadensis, Sium suave, Solidago canadensis*, *Solidago* *speciosa* var. *rigidiuscula*, Sphagnum capillifolium, Symphyotrichum laeve*,* Symphyotrichum puniceum, *Tanacetum vulgare, Taxus canadensis, Thalictrum dasycarpum, Thuja occidentalis, Urtica* *dioica* ssp. *gracilis,* *Vaccinium vitis-idaea, Valeriana dioica, Veratrum viride* |
| Gastro-intestinal disorders (biliousness, cholera, colic, constipation, indigestion, diarrhoea, dysentery, emetic, laxative, liver disorders, piles, physic, purgative, cathartic, intestinal worms, nausea, vomiting, etc.) | *Abies balsamea, Acer negundo, Acer nigrum, Acer pensylvanicum, Acer saccharinum, Achillea millefolium, Achillea sibirica, Acorus calamus, Actaea rubra, Agastache foeniculum, Aletris farinosa, Alisma plantago-aquatica, Allium tricoccum,* Alnus incana ssp*.* rugosa*, Alnus rubra, Alnus* sp., *Alnus viridis, Alnus* *viridis* ssp. *crispa, Amelanchier alnifolia, Amelanchier* *canadensis*, *Amorpha canescens, Amphicarpaea* *bracteata* var. *comosa, Andromeda* *polifolia* var. *latifolia*, *Andropogon gerardii, Antennaria* *plantaginifolia*, *Apocynum cannabinum, Aquilegia* *canadensis*, *Aralia nudicaulis, Aralia racemosa, Arctium minus,* *Arctostaphylos uva-ursi, Arisaema triphyllum, Artemisia absinthium, Artemisia campestris, Artemisia* *dracunculus*, *Artemisia frigida, Artemisia* sp., *Asarum canadense, Aspidium cristatum, Astragalus americanus, Baptisia tinctoria,* Betula alleghaniensis*, Betula* *glandulosa*, *Betula lenta, Betula* *nigra*, *Betula papyrifera, Betula* *pubescens*, *Boschniakia* *rossica*, *Botrychium virginianum, Capsella bursa-pastoris, Carex aquatilis, Carum carvi, Caulophyllum thalictroides, Ceanothus americanus, Ceanothus herbaceus, Celastrus scandens,* Chamerion angustifolium ssp*.* angustifolium, *Chimaphila umbellata, Cirsium arvense, Cirsium* *vulgare*, *Cladina stellaris, Comarum* *palustre*, *Comptonia* *peregrine*, *Coptis trifolia, Cornus alternifolia, Cornus canadensis, Cornus sericea, Corylus cornuta*, *Cypripedium acaule*, *Diervilla lonicera, Dirca palustris, Dryopteris* *marginalis*, *Echinocystis* *lobata*, *Echinodontium tinctorium, Empetrum nigrum, Equisetum arvense, Equisetum palustre, Equisetum pratense, Erigeron canadensis, Erigeron* sp.*, Eriophorum* sp., *Fagus grandifolia, Fomes pinicola, Fragaria vesca ssp. bracteata, Fragaria virginiana, Galeopsis tetrahit, Galium boreale*, *Geranium maculatum, Geum canadense, Geum rivale, Geum* *triflorum*, Gymnocarpium disjunctum*, Hamamelis virginiana, Hedysarum* *alpinum*, *Hepatica triloba,* Heracleum maximum*, Heuchera richardsonii, Hydrophyllum virginianum*, Ilex verticillata, Impatiens capensis, *Iris versicolor, Juglans cinerea, Juniperus communis, Kalmia angustifolia, Kalmia latifolia, Kalmia* *polifolia*, *Larix laricina, Lathyrus ochroleucus, Lilium canadense, Lilium philadelphicum, Lobelia inflata, Lobelia kalmia, Lonicera dioica, Lycopodium dendroideum, Lycopodium* sp., *Maianthemum racemosum* ssp. *racemosum, Matteuccia struthiopteris, Medicago sativa, Mentha arvensis, Mentha spicata, Monarda fistulosa, Monarda punctata, Nuphar* *lutea*, *Nuphar* *lutea* ssp. *polysepala, Oenothera biennis, Penstemon confertus, Physocarpus opulifolius, Phytolacca americana,* Picea glauca, *Picea mariana, Plantago major, Podophyllum peltatum, Polygonum muhlenbergii, Polygonum persicaria, Polygonum punctatum, Polypodium virginianum, Populus* sp.*, Populus tremuloides, Potentilla arguta, Prunella vulgaris*, *Prunus americana, Prunus pensylvanica, Prunus virginiana, Prunus* *virginiana* var. *demissa*, *Pteris aquilina, Quercus alba, Quercus macrocarpa, Quercus rubra,* *Rhododendron groenlandicum, Rhododendron* *tomentosum*, *Rhus aromatica, Rhus copallinum,* *Rhus glabra, Rhus typhina, Ribes lacustre, Ribes oxyacanthoides, Ribes* *triste*, *Rosa acicularis, Rosa* *blanda*, *Rosa* sp., *Rubus allegheniensis, Rubus chamaemorus, Rubus idaeus, Rubus idaeus* ssp. *strigosus, Rubus occidentalis, Rubus* sp.*, Rudbeckia laciniata, Rumex altissimus, Rumex crispus, Rumex* sp.*, Sagittaria cuneata, Sagittaria latifolia, Salix bebbiana, Salix discolor, Salix lucida, Salix* *pedicellaris*, *Salix* sp., *Sambucus nigra* ssp*. canadensis, Sambucus racemosa, Sanguinaria canadensis, Sarracenia purpurea, Saururus cernuus, Scutellaria lateriflora, Shepherdia canadensis, Silphium perfoliatum, Solanum dulcamara, Solidago canadensis, Sorbus americana, Sporobolus heterolepis, Stachys palustris,* Symphyotrichum puniceum*, Taraxacum officinale, Taxus canadensis, Thaspium barbinode, Thuja occidentalis, Tiarella cordifolia, Tilia americana, Triglochin maritima, Triosteum perfoliatum, Tsuga canadensis, Umbilicaria muehlenbergii, Urtica dioica, Urtica* *dioica* ssp. *gracilis, Uvularia grandiflora, Vaccinium angustifolium, Vaccinium myrtilloides, Vaccinium oxycoccus, Vaccinium vitis-idaea, Veratrum viride, Veronicastrum virginicum, Viburnum acerifolium, Viburnum* *edule*, *Viburnum* *opulus* var. *americanum,* *Vitis* *vulpina* |
| General health (no description or just mentioned as a medicinal plant) | *Abies balsamea, Acer pensylvanicum, Acorus calamus*, *Alisma plantago-aquatica, Amelanchier alnifolia*, Anemone multifida, *Apocynum* *cannabinum, Aralia nudicaulis, Aralia racemosa, Arctium minus, Arctostaphylos alpina, Arisaema triphyllum*, *Artemisia* sp., *Asarum canadense, Baptisia tinctoria*, *Betula lenta, Betula papyrifera, Boschniakia* *rossica*, *Celastrus scandens*, *Chelone glabra, Cicuta macualta, Clintonia* *uniflora, Cornus alternifolia, Cornus canadensis, Cornus sericea*, *Crataegus* sp., *Eupatorium perfoliatum, Fragaria* *vesca* ssp. *bracteata*, *Fragaria virginiana, Gaultheria procumbens, Geum macrophyllum,* Heliopsis helianthoides var. scabra, Heracleum maximum, *Iris versicolor, Juniperus communis, Juniperus horizontalis,* *Larix* *laricina*, *Lepidium virginicum*, *Lobelia inflata, Lycopodium* *dendroideum,* Lysichiton americanus, *Maianthemum* *stellatum*, *Matricaria discoidea, Mentha arvensis, Menyanthes* *trifoliata*, *Nuphar lutea, Oplopanax* *horridus, Ostrya* *virginiana,* Picea glauca*, Picea* *mariana, Plantago major, Polygala senega*, *Polygonum amphibium*, *Pontederia cordata, Populus balsamifera, Populus tremuloides, Pulsatilla* *patens* ssp. *multifida, Pyrola* *asarifolia* ssp. *asarifolia, Quercus rubra*, *Rhododendron* *groenlandicum, Rhododendron tomentosum,* *Rhus aromatica*, *Rhus glabra*, Ribes hudsonianum, *Rosa* *virginiana, Rumex* *altissimus, Rumex crispus, Sagittaria cuneata, Salix* sp., *Sambucus racemosa*, *Sanicula canadensis, Shepherdia canadensis, Solanum nigrum*, *Sorbus americana, Spiraea* *alba, Spiraea tomentosa, Streptopus amplexifolius, Symplocarpus foetidus, Taxus canadensis, Thuja occidentalis*, *Trametes suaveolens, Trientalis* *borealis* ssp. *borealis, Trillium erectum, Typha latifolia, Umbilicaria* *muehlenbergii*, *Valeriana* *dioica* var. *sylvatica, Veronicastrum* *virginicum, Viburnum* *edule*, *Zanthoxylum americanum* |
| Gynaecological problems (menstrual disorders, pain, emmenagogue, etc.) | *Abies balsamea, Achillea millefolium, Acorus calamus*, *Actaea* *pachypoda*, *Actaea rubra, Alnus* *viridis, Alnus* *viridis* ssp. *crispa, Aletris* *farinosa, Amelanchier* *canadensis*, *Aralia nudicaulis, Aralia racemosa*, *Arctostaphylos uva-ursi, Artemisia* *dracunculus*, *Asclepias syriaca, Betula papyrifera*, *Betula pumila* var. *glandulifera, Carex* *aquatilis, Castilleja* *coccinea, Caulophyllum* *thalictroides*, Chamerion angustifolium ssp. angustifolium, *Chenopodium ambrosioides, Cirsium* sp., *Cornus canadensis*, *Crataegus* sp., *Cypripedium acaule, Cypripedium* *parviflorum*, *Empetrum nigrum, Erigeron canadensis*, *Eupatorium maculatum, Eupatorium perfoliatum, Eupatorium purpureum, Fragaria virginiana, Fraxinus americana, Geum* *canadense, Geum macrophyllum***,** *Glyceria* *canadensis*, *Hepatica triloba*, *Hudsonia tomentosa, Juniperus communis*, *Lilium canadense*, *Linnaea borealis, Lonicera dioica, Maianthemum* *racemosum* ssp. *racemosum*, *Mentha arvensis*, *Monarda fistulosa, Myriosclerotinia caricis-ampullaceae, Nuphar lutea, Nymphaea* *odorata,* Osmorhiza longistylis*, Pastinaca* *sativa*, *Polyporus* sp., *Populus balsamifera*, *Populus* *grandidentata, Prenanthes* *alba, Prunella vulgaris*, *Pycnanthemum* *virginianum*, *Ribes* *americanum*, *Ribes* *glandulosum*, *Ribes oxyacanthoides, Ribes* *triste*, *Rosa acicularis, Rubus* *frondosus*, *Rubus idaeus, Rubus occidentalis, Rubus* *pubescens* var. *pubescens*, *Sanicula canadensis, Sanicula marilandica*, *Sanicula* *odorata*, *Sarracenia purpurea, Scutellaria* *lateriflora, Silphium perfoliatum, Solidago* *flexicaulis, Solidago juncea,* Symphyotrichum puniceum, *Tanacetum vulgare, Taraxacum* *officinale, Taxus canadensis, Thuja occidentalis, Trillium erectum, Urtica dioica, Vaccinium angustifolium, Vaccinium myrtilloides, Valeriana* *dioica* var. *sylvatica, Viburnum lentago*, *Viburnum* *opulus* var. *americanum* |
| Haemorrhages (internal bleeding, blood spitting, nasal haemorrhage, etc.) | *Acer pensylvanicum, Achillea millefolium*, *Achillea millefolium* ssp. *lanulosa* var. *lanulosa*, *Actaea rubra, Anemone canadensis, Apocynum androsaemifolium, Aralia nudicaulis, Aralia racemosa, Baptisia tinctoria, Calla palustris, Calvatia craniiformis, Eupatorium perfoliatum, Galium aperina, Hamamelis virginiana,* *Lycoperdon perlatum, Lycoperdon* sp., *Maianthemum* *racemosum* ssp. *racemosum,* *Mentha arvensis, Packera* *aurea, Plantago major, Populus balsamifera, Populus tremuloides, Polygonatum pubescens*, *Potentilla anserina*, *Pyrola* *asarifolia* ssp. *asarifolia, Rhododendron* *groenlandicum, Rhus typhina, Rubus* *idaeus* ssp. *strigosus, Sarracenia purpurea, Silphium perfoliatum, Sorbus americana, Streptopus amplexifolius, Trillium erectum, Urtica dioica, Urtica* *dioica* ssp. *gracilis*, *Usnea hirta, Usnea* sp., *Verbena* *hastata* |
| Hair care (prevent hair loss, scalp problems, lice) | *Abies balsamea, Androsace* *septentrionalis*, *Artemisia* *dracunculus*, *Delphinium* *glaucum*, *Dirca palustris,* Heracleum maximum, *Juniperus communis, Lonicera dioica,* *Poa* palustris, *Prunus virginiana*, *Rhododendron* *groenlandicum, Solidago* *speciosa* var. *rigidiuscula*, *Vitis* sp. |
| Headache | *Achillea millefolium*, *Achillea millefolium* ssp. *lanulosa* var. *lanulosa, Acorus* *americanus*, *Acorus calamus, Alisma plantago-aquatica,* *Amelanchier alnifolia,* Anemone multifida, *Anemone* sp., *Apocynum androsaemifolium, Aralia recemosa*, *Arctium minus*, *Arctostaphylos uva-ursi, Artemisia frigida, Castilleja miniata, Chamaecyparis* *thyoides*, *Chamaedaphne calyculata, Cicuta* *douglasii*, *Comptonia* *peregrine, Cornus sericea, Corydalis aurea*, *Cynoglossum* *virginianum* var. *boreale, Erigeron* *strigosus*, *Eurybia* *macrophylla*, *Fomes pinicola, Gaultheria procumbens, Grindelia squarrosa, Helenium autumnale*, Heracleum maximum, *Hymenoxys richardsonii,* Impatiens capensis, *Inula helenium, Juniperus communis, Juniperus virginiana, Kalmia angustifolia*, *Lappula* *squarrosa*, *Larix* *laricina, Maianthemum canadense, Maianthemum* *racemosum* ssp. *racemosum, Mentha* *aquatica, Monarda fistulosa, Nuphar lutea*, *Nuphar variegatum, Pedicularis* *lanata*, *Plantago major,* Picea glauca, *Picea* *mariana, Pinus resinosa*, *Pinus strobus, Polygonatum* *biflorum* var. *commutatum*, *Pteris aquilina, Pulsatilla* *patens* ssp. *multifida, Ranunculus acris, Rhododendron* *groenlandicum, Rhododendron tomentosum, Salix lucida*, *Salix* sp., *Sarracenia purpurea, Sium suave, Sorbus americana,* Symphyotrichum puniceum*, Symplocarpus foetidus, Tanacetum vulgare,* *Taxus canadensis,* *Thuja occidentalis*, *Thuja* *plicata*, *Trametes suaveolens*, *Vaccinium myrtilloides, Valeriana* *dioica* var. *sylvatica,* Viburnum lantanoides |
| Injuries (cuts and wounds, bruises, burns and scalds) | *Abies balsamea, Acer saccharinum, Acer spicatum, Aconogonon alaskanum*, *Acorus* *americanus, Acorus calamus*, *Achillea millefolium, Achillea* *millefolium* var. *occidentalis, Actaea rubra, Agastache foeniculum, Alnus viridis, Amelanchier* *bartramiana*, *Anemone canadensis, Aralia racemosa, Arctium lappa, Arctium minus,* *Artemisia* *dracunculus*, *Artemisia frigida, Asarum canadense, Asclepias tuberosa, Astragalus crassicarpus*, *Betula* *glandulosa*, *Betula nana,* *Betula papyrifera, Betula pumila* var. *glandulifera*, *Botrychium virginianum*, *Calla palustris*, Chamerion angustifolium ssp. angustifolium, *Chimaphila* *umbellate*, *Cicuta maculata, Cirsium arvense, Cirsium* *discolor*, Clintonia borealis, *Corylus americana, Cornus florida, Cornus sericea, Echinodontium tinctorium*, *Fagus grandifolia*, *Fomes pinicola, Fragaria virginiana*, *Fragaria* *virginiana* ssp. *glauca*, Gaultheria hispidula, *Hamamelis virginiana, Helianthus* *occidentalis*, *Hepatica triloba,* Heracleum maximum, *Iris versicolor, Juniperus communis*, *Kalmia angustifolia*, *Larix* *laricina, Lathyrus* *venosus*, *Lilium philadelphicum, Lycoperdon perlatum, Maianthemum canadense, Maianthemum* *racemosum* ssp. *racemosum*, *Monarda fistulosa, Nicotiana tabacum, Nuphar lutea, Nuphar* *lutea* ssp. *advena, Nuphar* *lutea* ssp. *polysepala,* *Oenothera biennis, Oligoneuron* *rigidum* var. *rigidum, Orthilia* *secunda*, *Packera* *aurea, Panax trifolius, Petasites frigidus, Phytolacca americana,* Picea glauca*, Picea* *mariana, Picea* *sitchensis, Pinus banksiana, Pinus* *contorta, Pinus* sp., *Pinus strobus*, *Plantago major,* *Polygala senega, Populus balsamifera, Populus* *deltoides, Populus tremuloides, Potentilla anserina*, *Potentilla arguta*, *Prunus* *americana*, *Prunus pensylvanica, Prunus serotina, Prunus virginiana, Pyrola asarifolia, Pyrola* *grandiflora*, *Quercus alba, Quercus* *macrocarpa*, *Rhododendron* *groenlandicum, Rhus glabra, Ribes lacustre, Rosa acicularis, Rubus chamaemorus, Rudbeckia laciniata*, *Rumex aquaticus, Rumex crispus, Rumex* *obtusifolius*, *Sagittaria cuneata, Sanguinaria canadensis, Salix bebbiana, Salix* *fragilis*, *Salix lucida*, *Salix* *nigra, Salix* sp., *Shepherdia canadensis, Silphium perfoliatum, Solidago* *altissima*, *Solidago* *speciosa* var. *rigidiuscula, Stachys palustris, Symphyotrichum* *ciliolatum, Taraxacum* *officinale, Thuja occidentalis, Tilia americana, Tsuga canadensis, Typha latifolia, Ulmus rubra, Urtica* *dioica* ssp. *gracilis, Verbascum thapsus* |
| Mental disorders (anxiety, depression, hallucination, stress, insanity, insomnia, nervousness, seizures, etc.) | *Caulophyllum* *thalictroides, Cypripedium acaule,* *Cypripedium* *parviflorum, Cypripedium* *reginae,* *Dryopteris carthusiana, Larix* *laricina, Matricaria discoidea, Mentha arvensis, Prunus serotina, Pulsatilla* *patens* ssp. *multifida, Rhododendron* *groenlandicum, Salix discolor, Sanguinaria canadensis,* Symphyotrichum puniceum, *Taraxacum* *officinale, Valeriana* *dioica* var. *sylvatica,* *Veratrum viride, Viburnum* *opulus* var. *americanum* |
| Metabolic system disorders (chill, refrigerant, prickly heat, sudorific) | *Abies balsamea, Apocynum androsaemifolium, Aralia nudicaulis, Aralia racemosa, Arctium minus, Artemisia* *dracunculus*, *Asclepias tuberosa, Chimaphila* *umbellata, Geum aleppicum, Mentha arvensis, Nuphar lutea, Prunus pensylvanica, Quercus alba*, *Rhododendron* *groenlandicum, Sambucus* *nigra* ssp. *canadensis, Streptopus* *lanceolatus* var. *roseus,* Symphyotrichum puniceum, *Tanacetum vulgare, Tsuga canadensis*, *Valeriana dioica* |
| Musculoskeletal disorders (analgesic, arthritis, rheumatism, body pain, joint pain, sprains, swellings, cramps, retching, muscle relaxant, etc.) | *Abies balsamea, Abies* *lasiocarpa*, *Acer pensylvanicum, Achillea millefolium*, *Achillea* *millefolium* var. *occidentalis, Acorus* *americanus*, *Acorus calamus*, *Alnus* sp., *Alnus* *viridis* ssp. *crispa,* *Amelanchier alnifolia,* Anaphalis margaritacea, *Anemone canadensis, Aralia racemosa*, *Arctium lappa*, *Arctium minus, Arctostaphylos* *alpina, Arctostaphylos uva-ursi, Arnica* *angustifolia*, *Artemisia absinthium, Artemisia frigida, Artemisia* *norvegica* ssp. *saxatilis, Asarum canadense,* Betula alleghaniensis, *Betula papyrifera, Brassica* sp., *Calla palustris*, *Caltha palustris, Carum carvi, Carya* *cordiformis*, *Carya* *laciniosa*, *Carya* *ovata*, *Caulophyllum* *thalictroides, Celastrus scandens, Chamaecyparis* *thyoides*, Chamerion angustifolium ssp. angustifolium, *Chenopodium album, Chimaphila* *umbellata,* Clintonia borealis, *Cicuta maculata, Cirsium arvense,* Comptonia peregrina, *Cornus canadensis, Cornus sericea, Cornus* sp., *Cypripedium* *parviflorum, Dryopteris* sp., *Eupatorium maculatum, Eupatorium perfoliatum, Fagus grandifolia, Fomes fomentarius, Gaultheria procumbens,* Heracleum maximum, *Hydrophyllum virginianum*, *Inonotus obliquus, Iris versicolor, Juniperus communis*, *Juniperus horizontalis, Juniperus virginiana, Kalmia angustifolia, Larix* *laricina, Lilium philadelphicum, Linnaea borealis, Lycopodium* *obscurum, Maianthemum canadense, Maianthemum* *racemosum* ssp. *racemosum, Matteuccia struthiopteris, Medicago sativa, Mentha arvensis, Mirabilis* *nyctaginea*, *Mitchella repens, Monarda fistulosa, Morella* *pensylvanica, Nuphar lutea, Nuphar* *lutea* ssp. *advena, Nuphar* *lutea* ssp. *polysepala, Nuphar variegatum*, *Nymphaea* *odorata, Oplopanax* *horridus*, *Ostrya* *virginiana, Petasites* *frigidus* var. *sagittatus*, *Phryma leptostachya*, *Phytolacca* *americana,* Picea glauca*, Picea* *mariana*, *Picea* *sitchensis, Picea* sp., *Pinus banksiana, Pinus resinosa, Pinus strobus, Plantago major, Platanthera* *dilatata* var. *leucostachys, Polystichum* *acrostichoides*, *Populus balsamifera, Populus tremuloides, Prunus pensylvanica, Prunus serotina*, *Pulsatilla* *patens* ssp. *multifida*, *Pyrola* *grandiflora*, *Quercus* *macrocarpa*, *Rhododendron* *groenlandicum, Rhus typhina, Ribes oxyacanthoides, Rubus occidentalis, Rumex aquaticus, Rumex crispus, Rumex orbiculatus, Rumex* *salicifolius* var. *mexicanus, Salix* *nigra*, *Salix* sp., *Sanicula* *odorata*, *Sarracenia purpurea, Shepherdia canadensis, Silphium perfoliatum, Solidago* *speciosa* var. *rigidiuscula, Sorbus americana, Sorbus* *decora, Sorbus* *scopulina*, *Sorbus* *sitchensis*, Sphagnum capillifolium, *Sporobolus* *heterolepis*, *Symplocarpus foetidus, Taraxacum officinale, Taxus canadensis, Thuja occidentalis,* Toxicodendron pubescens, *Trillium* *grandiflorum*, *Tsuga canadensis, Typha latifolia*, *Urtica* *dioica* ssp. *gracilis*, *Utricularia* *vulgaris*, *Vaccinium myrtilloides, Valeriana* *dioica* var. *sylvatica, Valeriana dioica, Vitis* *vulpina* |
| Nervous system disorders (convulsions, paralysis, hyperactivity, etc.) | *Abies balsamea, Acorus calamus*, *Actaea* *pachypoda*, Anaphalis margaritacea, *Artemisia frigida, Asarum canadense, Astragalus crassicarpus*, *Botrychium virginianum, Carya* *ovata,* *Castilleja miniata, Caulophyllum* *thalictroides, Cornus canadensis, Cornus sericea*, *Corylus americana, Fagus grandifolia, Galeopsis tetrahit, Hepatica* *nobilis* var. *obtusa*, Heracleum maximum, *Lathyrus* *venosus*, Leucanthemum vulgare, *Moneses* *uniflora, Polypodium* *virginianum*, *Potentilla arguta*, *Pulsatilla* *patens* ssp. *multifida, Ribes* sp., *Rosa arkansana*, *Rosa* sp., *Rubus* sp., *Solidago juncea,* Symphyotrichum puniceum, *Thuja occidentalis* |
| Nutritional disorders (weight loss, weight gain, tonic, immune stimulant, appetizers, etc.) | *Abies balsamea, Acer saccharinum, Acer* *saccharum, Achillea millefolium*, *Acorus calamus, Aletris* *farinosa, Alnus* sp., *Aralia nudicaulis, Aralia racemosa, Arctium minus*, *Arctostaphylos uva-ursi, Artemisia absinthium, Artemisia frigida, Astragalus crassicarpus*, *Betula lenta, Betula papyrifera, Boschniakia* *rossica*, Cardamine diphylla, *Chaenactis* *douglasii*, *Chimaphila* *umbellata*, *Cirsium arvense, Comptonia* *peregrina, Coptis* *trifolia*, *Cornus sericea, Dryopteris* *expansa*, *Echinocystis* *lobata*, *Fagus grandifolia, Fraxinus* *pennsylvanica*, *Fraxinus* sp., *Galeopsis tetrahit,* Gaultheria hispidula, *Gaultheria procumbens, Geum* *triflorum, Hedysarum* *alpinum*, Heliopsis helianthoides var. scabra, *Inonotus obliquus, Juniperus communis, Kalmia angustifolia, Lathyrus* *venosus*, *Lycopodium clavatum, Maianthemum* *stellatum*, *Matteuccia struthiopteris,* Melilotus officinalis, *Mentha arvensis, Polygala senega, Populus* *grandidentata, Populus tremuloides,* Prosartes trachycarpa, *Prunella vulgaris, Prunus serotina, Pteris* sp., *Pycnanthemum* *virginianum, Pyrola* *elliptica, Rhododendron* *groenlandicum, Rhus typhina, Rosa acicularis, Rosa arkansana, Rubus idaeus, Salix bebbiana, Salix cordata, Salix* *nigra, Sanguinaria canadensis, Sium suave, Smilax herbacea, Solidago multiradiata, Solidago* *speciosa* var. *rigidiuscula, Sorbus americana, Taraxacum* *officinale, Taxus canadensis, Valeriana dioica, Valeriana* *dioica* var. *sylvatica* |
| Odontological problems (teething sicknesses, tooth ache, gum problems, decayed teeth) | *Achillea* *millefolium* var. *occidentalis, Achillea sibirica, Acorus calamus*, *Alnus* incana ssp. rugosa*, Amelanchier alnifolia, Aralia nudicaulis, Berberis vulgaris*, *Betula papyrifera, Coptis* *trifolia, Cypripedium acaule, Equisetum arvense, Fragaria* *vesca* ssp. *bracteata*, Gaultheria hispidula, *Geum aleppicum,* *Geum macrophyllum,* Heracleum maximum*, Juniperus communis, Juniperus horizontalis, Lilium philadelphicum, Mentha arvensis, Monotropa uniflora, Nicotiana tabacum*, *Orthilia* *secunda*, *Parmelia sulcata, Plantago major,* Picea glauca, *Picea* *mariana, Polygala senega*, *Populus balsamifera, Populus tremuloides, Pyrola asarifolia*, *Rhododendron* *groenlandicum, Rubus idaeus, Salix bebbiana*, *Salix* sp., *Sambucus racemosa, Sorbus americana*, Sphagnum capillifolium, Symphyotrichum laeve, Symphyotrichum puniceum*, Taxus canadensis*, *Thuja occidentalis*, *Verbascum thapsus, Viburnum* *edule* |
| Ophthalmological disorders (eye wash, sore eyes, infection, snow blindness) | *Abies balsamea, Acer rubrum, Acer spicatum*, *Achillea millefolium*, *Acorus calamus,* Alnus incana ssp. rugosa, *Alnus* sp., *Amanita muscaria, Apocynum androsaemifolium, Arisaema triphyllum, Betula papyrifera, Chimaphila* *umbellata, Coptis* *trifolia, Cornus alternifolia*, *Cornus sericea*, *Diervilla lonicera, Evernia mesomorpha, Fraxinus nigra*, *Hamamelis virginiana, Hedysarum* *alpinum*, *Heuchera richardsonii, Hordeum jubatum, Larix* *laricina, Matricaria discoidea*, *Melampyrum* *lineare*, *Monotropa uniflora, Myrica gale,* Picea glauca, *Picea* *mariana, Picea* *sitchensis*, *Pinus banksiana, Populus* sp., *Prunus pensylvanica, Pyrola asarifolia*, *Rhododendron* *groenlandicum, Rhus glabra, Ribes oxyacanthoides, Rosa acicularis, Rosa* *blanda, Rosa* sp., *Rosa* *virginiana, Rubus chamaemorus, Rubus idaeus, Rubus* *idaeus* ssp. *strigosus, Rubus occidentalis*, *Salix lucida*, *Salix* sp., *Stellaria* *media, Streptopus* *lanceolatus, Symphoricarpos* *orbiculatus*, *Symphyotrichum* *ciliolatum, Taxus canadensis, Tilia americana, Usnea* sp., *Viburnum edule, Viburnum opulus*, *Viola* *bicolor* |
| Poisoning (insect bites, snake bites, bee stings, food intoxication ) | *Abies balsamea, Achillea millefolium, Achillea* *millefolium* var. *occidentalis, Acorus* *americanus*, *Aralia nudicaulis, Arctostaphylos uva-ursi, Artemisia frigida, Betula* *glandulosa*, *Betula* *neoalaskana*, *Betula papyrifera, Botrychium virginianum,* Clintonia borealis, Chamerion angustifolium ssp. angustifolium, *Chamerion* *latifolium,* Comptonia peregrina, *Cornus sericea, Eupatorium perfoliatum, Eryngium* *aquaticum, Gymnocarpium dryopteris, Lilium canadense, Lilium philadelphicum, Picea glauca, Pinus strobus, Plantago major, Polygala senega, Populus balsamifera*, *Populus tremuloides, Prunus pensylvanica, Prunus virginiana, Rosa acicularis, Rosa* *woodsii*, *Rubus chamaemorus, Salix* sp., *Sagittaria cuneata, Sanicula* *odorata*, *Sanicula marilandica*, *Sarracenia purpurea, Taraxacum* *officinale, Typha latifolia, Urtica* *dioica* ssp. *gracilis,* *Viburnum* *edule* |
| Pregnancy, child birth, puerperium (labour induction, labour pain, after child birth, miscarriages, abortion, lactation stimulant, pregnancy prevention) | *Abies balsamea, Acorus calamus, Achillea millefolium,* *Actaea rubra, Alisma plantago-aquatica, Amelanchier* *laevis*, *Antennaria* *howellii* ssp. *neodioica*, Antennaria neglecta, *Apocynum androsaemifolium, Aralia nudicaulis, Arctostaphylos uva-ursi*, *Artemisia* *dracunculus, Asclepias syriaca, Betula papyrifera, Caltha palustris*, *Caulophyllum* *thalictroides, Chelone glabra,* Clintonia borealis, *Diervilla lonicera*, *Empetrum nigrum, Eupatorium maculatum, Fraxinus americana, Geum rivale, Grindelia squarrosa, Hierochloe odorata, Juniperus communis*, *Juniperus horizontalis, Lactuca* *biennis*, *Lonicera dioica, Lycopodium* sp., *Maianthemum canadense, Matteuccia struthiopteris, Matricaria discoidea, Monarda fistulosa, Myriosclerotinia caricis-ampullaceae, Nuphar lutea, Onoclea* *sensibilis, Oplopanax* *horridus,* Osmorhiza longistylis, *Packera* *aurea, Panax* *quinquefolius*, *Petasites* *frigidus* var. *sagittatus*, Picea glauca*, Picea* *mariana, Pontederia cordata, Populus* *grandidentata, Populus tremuloides, Prunus virginiana*, *Rhododendron* *groenlandicum, Ribes* *glandulosum,* Ribes hudsonianum*, Ribes lacustre, Ribes oxyacanthoides, Rubus allegheniensis, Rubus idaeus, Salix discolor, Sanguinaria canadensis, Sanicula canadensis*, *Sanicula* *odorata,* *Sarracenia purpurea, Shepherdia canadensis*, *Solidago* *speciosa* var. *rigidiuscula, Sorbus americana*, Sphagnum capillifolium, *Sphagnum fuscum, Spiraea tomentosa,* Symphyotrichum leave, Symphyotrichum puniceum, Symphoricarpos albus var. albus, *Tanacetum vulgare, Taxus canadensis, Thuja occidentalis, Trillium undulatum*, *Tsuga canadensis, Umbilicaria* *mammulata*, *Urtica dioica, Vaccinium angustifolium, Vaccinium myrtilloides, Vaccinium vitis-idaea, Valeriana dioica, Viburnum* *opulus* var. *americanum, Viburnum prunifolium, Vitis* *vulpina* |
| Respiratory system disorders (asthma, catarrh, diptheria, expectorant, hiccups, hoarseness, pleurisy, pneumonia, tuberculosis, consumption, coughing up blood, scrofula) | *Abies balsamea, Acer pensylvanicum, Acer saccharinum, Achillea millefolium, Acorus calamus, Agastache foeniculum, Alnus* sp., *Amelanchier alnifolia,* Anaphalis margaritacea, *Anemone* *cylindrica,* *Antennaria* *plantaginifolia*, *Apocynum androsaemifolium, Aralia racemosa, Arisaema triphyllum*, *Artemisia* *tilesii*, *Asclepias tuberosa, Asarum canadense*, *Betula lenta, Betula papyrifera, Betula* *pubescens, Betula pumila* var. *glandulifera*, *Botrychium virginianum, Caltha palustris, Campanula rotundifolia,* Cardamine diphylla*,* *Caulophyllum* *thalictroides, Ceanothus americanus*, *Ceanothus* *herbaceous*, *Cetraria* *islandica*, *Chimaphila* *umbellata,* Clintonia borealis, *Cirsium arvense,* Comptonia peregrine, *Cornus sericea, Dirca palustris*, *Equisetum arvense, Euthamia* *graminifolia*, *Fagus grandifolia, Galeopsis tetrahit, Galium* *tinctorium*, Gaultheria hispidula, *Gaultheria procumbens, Geum* *aleppicum*, *Gnaphalium* sp., *Heracleum maximum*, *Iris versicolor, Juniperus communis, Larix* *laricina*, *Lilium philadelphicum*, *Limonium* *carolinianum, Linaria* *vulgaris*, *Linnaea borealis, Lonicera dioica, Maianthemum* *racemosum* ssp. *racemosum*, *Menispermum canadense, Mentha arvensis, Monarda fistulosa, Myrica gale, Nuphar lutea, Nuphar* *lutea* ssp. *polysepala*, *Nymphaea* *odorata*, *Oplopanax* *horridus*, *Ostrya* *virginiana, Petasites* *frigidus* var. *palmatus*, *Petasites* *frigidus* var. *sagittatus*, Picea glauca, *Picea* *mariana*, *Picea* *rubens, Picea* *sitchensis, Picea* sp., *Pinus banksiana, Pinus strobus, Polypodium* *virginianum, Polystichum* *acrostichoides, Populus balsamifera, Populus tremuloides, Prunus pensylvanica, Prunus serotina, Prunus virginiana*, *Pyrola asarifolia, Quercus* *macrocarpa, Quercus rubra*, *Rhododendron* *groenlandicum, Rhododendron tomentosum*, *Rhus glabra*, *Rhus typhina*, *Ribes oxyacanthoides, Rubus allegheniensis, Rubus* *frondosus*, *Rubus idaeus, Rubus* sp., *Sagittaria cuneata, Salix lucida*, *Sanguinaria canadensis, Sarracenia purpurea, Sassafras albidum, Shepherdia canadensis, Silphium perfoliatum, Sium suave, Smilax herbacea, Solidago* *speciosa* var. *rigidiuscula,* *Sorbus americana, Sorbus* *scopulina*, *Sorbus* *sitchensis*, Symphyotrichum puniceum*, Symplocarpus foetidus, Thuja occidentalis*, *Ulmus americana*, *Ulmus rubra, Umbilicaria* *muehlenbergii*, *Urtica dioica, Vaccinium* *macrocarpon, Valeriana dioica, Valeriana* *dioica* var. *sylvatica, Verbascum thapsus, Veronicastrum virginicum*, *Vitis* sp., *Zanthoxylum americanum* |
| Sexual dysfunction | *Dactylorhiza* *viridis*, *Oxalis* *montana*, *Pedicularis canadensis* |
| Urinary system disorders (kidney, bladder, gravel, urination, urinary tract infection, etc.) | *Abies balsamea, Acer pensylvanicum, Acer saccharinum,* *Achillea millefolium, Actaea* *racemosa* var. *racemosa, Agrimonia* *gryposepala*, *Alnus* sp., *Apocynum androsaemifolium, Aralia nudicaulis, Aralia racemosa, Arctium minus, Arctostaphylos uva-ursi, Artemisia frigida, Athyrium* *filix-femina, Baptisia tinctoria, Betula* *nana* ssp. *exilis, Caltha palustris, Caulophyllum* *thalictroides, Celastrus scandens*, *Chimaphila* *umbellate, Clintonia* *uniflora*, *Cirsium arvense, Cornus canadensis*, *Cornus sericea*, *Cucurbita* *maxima*, *Cypripedium acaule*, *Diervilla lonicera*, *Dirca palustris*, *Dryopteris carthusiana, Empetrum nigrum, Epigaea repens, Equisetum arvense, Equisetum pratense, Equisetum sylvaticum, Eryngium* *aquaticum, Eupatorium perfoliatum, Fagus grandifolia, Fragaria virginiana, Galium aparina*, *Galium boreale, Gaultheria procumbens, Humulus* *lupulus*, *Iris versicolor, Juniperus communis*, *Juniperus horizontalis, Juniperus virginiana, Laportea* *canadensis*, *Larix* *laricina, Lasallia* *papulosa, Lonicera* *canadensis, Lonicera dioica*, *Lycopodium* *dendroideum*, *Lycopodium* sp., *Malaxis* *unifolia*, *Maianthemum* *racemosum* ssp. *racemosum*, *Matricaria discoidea, Mentha* *aquatica, Morella* *pensylvanica, Nuphar lutea, Nuphar* *lutea* ssp. *polysepala, Oligoneuron* *rigidum* var. *rigidum, Oplopanax* *horridus, Ostrya* *virginiana,* *Platanthera* *dilatata*, Picea glauca, *Pinus strobus, Polypodium* *virginianum*, *Populus* sp., *Pyrola asarifolia, Pyrola* *asarifolia* ssp. *asarifolia, Rhododendron* *groenlandicum, Ribes* sp., *Ribes* *triste*, *Rubus allegheniensis, Rubus idaeus, Rumex crispus, Salix discolor*, *Salix* *nigra, Salix* sp*., Sambucus racemosa, Sanicula* *odorata, Sarracenia purpurea, Smilax herbacea, Solidago canadensis, Sorbus americana,* Sphagnum capillifolium, *Streptopus amplexifolius, Symphoricarpos* *occidentalis*, Symphyotrichum puniceum*, Tanacetum vulgare, Taraxacum* *officinale,* *Thuja occidentalis, Thuja* *plicata*, *Trifolium hybridum, Triosteum* *perfoliatum*, *Tsuga canadensis, Typha angustifolia, Typha latifolia, Ulmus* *rubra*, *Urtica* *dioica* ssp. *gracilis, Utricularia* *vulgaris*, *Vaccinium angustifolium, Vaccinium vitis-idaea, Viburnum* *edule*, *Viburnum lentago, Viburnum opulus, Viburnum* *opulus* var. *americanum*, *Viola canadensis, Zizania aquatica* |
| Venereal diseases (gonorrhea, syphilis, etc.) | *Abies balsamea, Acer pensylvanicum, Acorus calamus, Alnus viridis, Alnus* *viridis* ssp. *crispa,* *Aralia racemosa, Arctium lappa, Baptisia tinctoria, Betula pubescens, Chimaphila umbellata, Cypripedium acaule, Eupatorium perfoliatum, Eupatorium maculatum, Fragaria vesca* ssp*. bracteata, Galium aparina, Grindelia squarrosa,* Heracleum maximum, *Larix laricina, Lepargyrea canadensis, Lonicera dioica, Lonicera involucrata, Picea mariana, Pyrola asarifolia* ssp*. asarifolia, Rhus glabra, Sarracenia purpurea, Shepherdia canadensis, Streptopus amplexifolius,* *Ulmus rubra* |
